# Genome analysis of Neisseria gonorrhoeae in Norway, 2016–2023, reveals shifting epidemiology in the wake of the COVID-19 pandemic

**DOI:** 10.1099/mgen.0.001479

**Published:** 2025-09-11

**Authors:** Kristian Alfsnes, Magnus N. Osnes, Hanne Margrethe Gilboe, Vegard Eldholm, Dominique A. Caugant

**Affiliations:** 1 Division for Infection Control, Norwegian Institute of Public Health, 0213 Oslo, Norway; 2 Norwegian Veterinary Institute, 1433 Ås, Norway; 3 Department of Microbiology, Oslo University Hospital, Oslo, Norway; 4 Department of Community Medicine and Global Health, Faculty of Medicine, University of Oslo, Blindern, 0316 Oslo, Norway

**Keywords:** antimicrobial resistance, molecular epidemiology, phylogenetics, ST (sequence type)-1580 genomics, surveillance, whole-genome sequencing

## Abstract

The incidence of gonorrhoea has increased significantly in Norway over the past 10 years. The emergence of antimicrobial resistance and potential for increased rates of future treatment failure make epidemiological surveillance of *Neisseria gonorrhoeae* a top priority. Here, we combine epidemiological data and genomic analyses of isolates from cases in Norway between January 2016 and May 2023 to identify genomic clusters and antimicrobial resistance patterns. We describe how the epidemiology of *N. gonorrhoeae* has changed before and after the COVID-19 pandemic and the lockdown restrictions of the Norwegian society. A total of 4,096 isolates, representing 39% of all the cases recorded by the Norwegian Surveillance System for Communicable Diseases, were analysed by whole-genome sequencing. Forty-one clusters, 12 of which encompassed more than 100 isolates, were identified. As seen in other countries, the lockdown of society in 2020–2021 led to a dramatic reduction in the cases of gonorrhoea. With the lifting of the restrictions, one cluster, dominated by sequence type 1580, rapidly expanded among young, heterosexual men and women. A significant increase in the fraction of female patients was observed through the study period. An increase in isolates resistant to azithromycin was observed throughout the study period, while the proportion of ciprofloxacin and cefixime resistance remained stable. A single ceftriaxone-resistant isolate was identified.

## Data Summary

Sequence data have been deposited in the European Nucleotide Archive under accession numbers PRJEB32435 and PRJEB83897. All supporting data, code and protocols have been provided within the article.

**Impact Statement**

The gonorrhoea epidemic in Norway has increased sharply in the years before the COVID-19 pandemic, mainly because of transmission among men who have sex with men. As in other countries, the lockdown imposed to stop the spread of COVID-19 resulted in a dramatic reduction of gonorrhoea cases. Following the lifting of the restrictions, preexisting clusters disseminated in the population. This study reports the changes in epidemiology and in the phylogenetic clusters of *Neisseria gonorrhoeae* in Norway before, during and after the COVID-19 pandemic. Our findings of the emergence of new clusters and antimicrobial resistance are of interest for public health surveillance in other European countries and globally.

## Introduction

In Norway, the incidence of gonorrhoea, caused by the sexually transmitted Gram-negative bacterium *Neisseria gonorrhoeae*, increased sharply from around 2010 until the onset of the COVID-19 pandemic [1]. This epidemic surge was driven mainly by men who have sex with men (MSM) [2,3]. However, non-pharmaceutical interventions imposed during the COVID-19 pandemic, such as lockdowns and border restrictions, not only reduced transmission of SARS-CoV-2 but also led to a reduction in cases of invasive infectious diseases [4] and sexually transmitted infections [5]. On 12 March 2020, public health measures were put in place in Norway to limit the spread of SARS-CoV-2, including limits on gatherings and implementation of border restrictions. These measures were strictly observed by the Norwegian population. The society reopened during the summer of 2020, and travel restrictions were lifted. Following an increase in SARS-CoV-2 cases in July–August 2020 and the occurrence of several local outbreaks, a new lockdown was imposed on September 2020 until the spring 2021, when a large proportion of the most vulnerable population had been vaccinated.

Travel is a key driver of gonococcal infections [6] and represents a significant risk for transmission of antibiotic-resistant strains [7]. A recent example was the introduction into the Norwegian population of *N. gonorrhoeae* sequence type (ST) 7827, which harboured resistance to fluoroquinolones and reduced susceptibility to both azithromycin (AZM) and the extended-spectrum cephalosporins, cefixime (CFM) and ceftriaxone (CRO) [8]. The lineage likely originated in East Asia and spread to several European countries, the USA and Australia, resulting in outbreaks nearly exclusively affecting men [8,10].

The objectives of this study were (1) to describe the demographic changes in the population affected by *N. gonorrhoeae* during and after the COVID-19 pandemic, (2) to investigate the impact of non-pharmaceutical interventions on the population structure of the pathogen and (3) to assess whether new lineages with reduced susceptibility to antibiotics have emerged in Norway following the lift of all COVID-19 preventive measures.

## Methods

The Norwegian Institute of Public Health (NIPH) hosts the National Reference Laboratory for Gonococci and receives all gonococcal isolates cultured by the medical laboratories in Norway. In this study, we included all isolates collected between January 2016 and May 2023, a total of 4,431 isolates. From these, 4,111 isolates had been submitted to whole-genome sequencing. From some patients, several isolates were received from several clinical sites (e.g. from throat, genitalia and rectum) at a single infection episode. In cases where these were found genetically identical (same multilocus ST), a single isolate, randomly selected, was kept in the analyses. If the patients harboured different genotypes during a single episode of infection, the distinct isolates (2 or 3) were included in the analyses. Thus, the total number of included isolates was 4,096 (Table S1, available in the online Supplementary Material).

Upon receipt, the isolates were grown overnight on chocolate blood agar for DNA extraction, followed by whole-genome sequencing (see below). Antibiotic susceptibility testing was performed on a GC agar base supplemented with 1% isovitalex and 1% haemoglobin. Beta-lactamase production was detected using the nitrocefin test. MICs of antibiotics were determined using E-test (bioMérieux, Marcy-l’Etoile, France). The antibiotics tested were AZM, ciprofloxacin (CIP), CRO, CFM, penicillin G, spectinomycin and tetracycline. Interpretation of results (susceptible/intermediate/resistant) was according to clinical breakpoints set by the European Committee for Antimicrobial Susceptibility Testing (EUCAST) for 2024 (version 14.0).

Date of sample collection, age and sex of the pseudonymized patients were obtained from NIPH laboratory management software. Data on all notified cases of gonorrhoea during the study period 2016–2023 were obtained from the Norwegian Surveillance System for Communicable Diseases (MSIS). To determine the effect of the Norwegian government-imposed lockdowns on the epidemiology of *N. gonorrhoeae*, we defined the period in the dataset corresponding to the lockdown from March 2020 to June 2021. A more detailed description of the timeline of imposed regulations/interventions can be found online [11].

DNA was extracted using MagNA Pure 96 (Roche Life Science), and DNA sequencing libraries were prepared from the extracted DNA using KAPA HyperPlus kits (Roche Life Science) with NEXTflex DNA barcodes (Bioo Scientific) following the manufacturer’s instructions. The DNA libraries were sequenced on a MiSeq or NextSeq platform (Illumina) using the v2 500-cycle or the v3 600-cycle reagent kits (Illumina) following the manufacturer’s instructions.

Illumina reads were trimmed using Trimmomatic v0.39 [12] and assembled *de novo* using SPAdes v3.14.1 [13]. QUAST v5.2 [14] was run on assemblies to assess and confirm the quality of the sequences. Contigs smaller than 2 Mbp and larger than 2.5 Mbp were removed. The ST of the isolates was determined using the PubMLST typing scheme [15], mlst v2.23 (https://github. ‌com/tseemann/mlst). Sequences were further analysed using PathogenWatch 22.3.8 (https://pathogen.watch/) to identify alleles of genes linked to antimicrobial resistance (AMR). In addition to the *mtrD* mosaic detection in PathogenWatch, we established a separate analysis to calculate the allelic distance (as a proxy for mosaicism) of the *mtrD* genes of the isolates to the reference sequence FA 1090. Briefly, assemblies were queried against an *mtrD* (NEIS1633) allele database downloaded from PubMLST (https://pubmlst.org/), using the blastn option in blast 2.5.0+ [16], selecting the best hit. Furthermore, a multifasta alignment was generated from the identified *mtrD* allele sequences of the isolates by aligning them to the reference sequence FA 1090 (GCF_000006845.1) *mtrD* using Aliview 1.28 [17]. The SNP distance to the WT FA 1090 *mtrD* allele was then estimated for each allele using snp-dists v0.8.2 (https://github.com/tseemann/snp-dists). The 23S alleles, A2048G and C2600T, were characterized by querying raw reads against the WT FA 1090 gene sequence using bwa v0.7.19 [18]. Copy number of the 23S alleles was calculated by the relative distribution of reads, where >13%, >38%, >63% and >88% corresponded to 1, 2, 3 or 4 copies, respectively. Similarly, *penA* alleles were identified and defined as mosaic or non-mosaic, by querying assemblies against the *penA* allele database (https:// ‌ngstar.canada.ca/alleles/penA) available from NG-STAR.

Assemblies were aligned using Parsnp v1.7.4 [19] using FA 1090 as the reference sequence. The resulting core genome alignment was converted to a full genome multifasta using parsnp2fasta.sh (https://github.com/krepsen). Homologous recombination was removed using Gubbins v3.3.1 [20] (using default settings, tree-building algorithm RAxML). BAPS clusters were defined using fastBAPS v1.0.8 [21] (using the phylogeny from Gubbins as input and setting the Dirichlet prior hyperparameter to the second most conservative option ‘baps’). BAPS level 1 was used to define the clusters. BAPS clusters comprising more than 100 isolates were uniquely labelled, while clusters with fewer isolates were pooled together and labelled as ‘Other’.

Sequence assemblies identified as ST-1580, an ST that re-emerged in Norway after the pandemic (see results), were downloaded from PathogenWatch 22.3.8 (https://pathogen.watch/). A separate alignment was performed for the ST-1580 sequences in our dataset together with those from PathogenWatch with country of origin and date of isolation (Table S2), the sequences were aligned using Parsnp v1.7.4, and homologous recombination was removed using Gubbins v3.3.1 as described above.

To reconstruct the population dynamics of the Norwegian ST-1580, a higher sensitivity protocol was used; the sequences of all Norwegian ST-1580 were aligned to the reference sequence FA 1090 using Snippy v.4.6.0 (github.com/tseemann/snippy), followed by detection and removal of homologous recombination using Gubbins v3.3.1 (tree-building algorithm IQTree). A time-scaled phylogeny was inferred from the resulting alignment using BactDating v1.1.1 [22] incorporating the estimated fractions of recombination on each branch from Gubbins. Temporal signal was assessed using root-to-tip regression analysis in BactDating. Five molecular clock models were evaluated: a strict clock [23], an uncorrelated relaxed clock (RC) [24], an additive RC (ARC) [25], a continuous additive RC (cARC) [25] and a relaxed negative binomial clock [26] as implemented in BactDating [22]. The optimal molecular clock model was selected based on the Deviance Information Criterion. Markov Chain Monte Carlo (MCMC) chains were run for 10^7^ iterations, discarding the first half as burn-in, with the remaining samples thinned to retain 10,000 posterior samples. Three MCMC chains were run and checked for convergence by ensuring that the effective sample size was above 200 and that the Gelman–Rubin criterion was less than 1.01 for all the monitored parameters. Node dates were summarized as the median posterior estimate, with 95% credibility intervals determined by the 2.5% and 97.5% percentiles. Finally, the effective population sizes of the Norwegian ST-1580 lineage BAPS 7 and BAPS 1 were estimated using Skygrowth v0.3.1 [27] using maximum posterior estimates.

All statistical analyses were done in R v4.2.1, with the following additional packages: ggplot2 v3.3.5, ggtree v3.4.2, phytools v1.2.0, ape v5.4.1 and pegas v1.3. Nucleotide diversity was measured as average pairwise differences per site, π [28], and the Watterson estimator, θ [29], using the nuc.div and theta.s commands in pegas v1.3. Nucleotide diversity measurements were done on subsets of the full genome fasta alignment (described above), where the sequences were selected by their respective months of isolation.

## Results

**Fig. 1.**
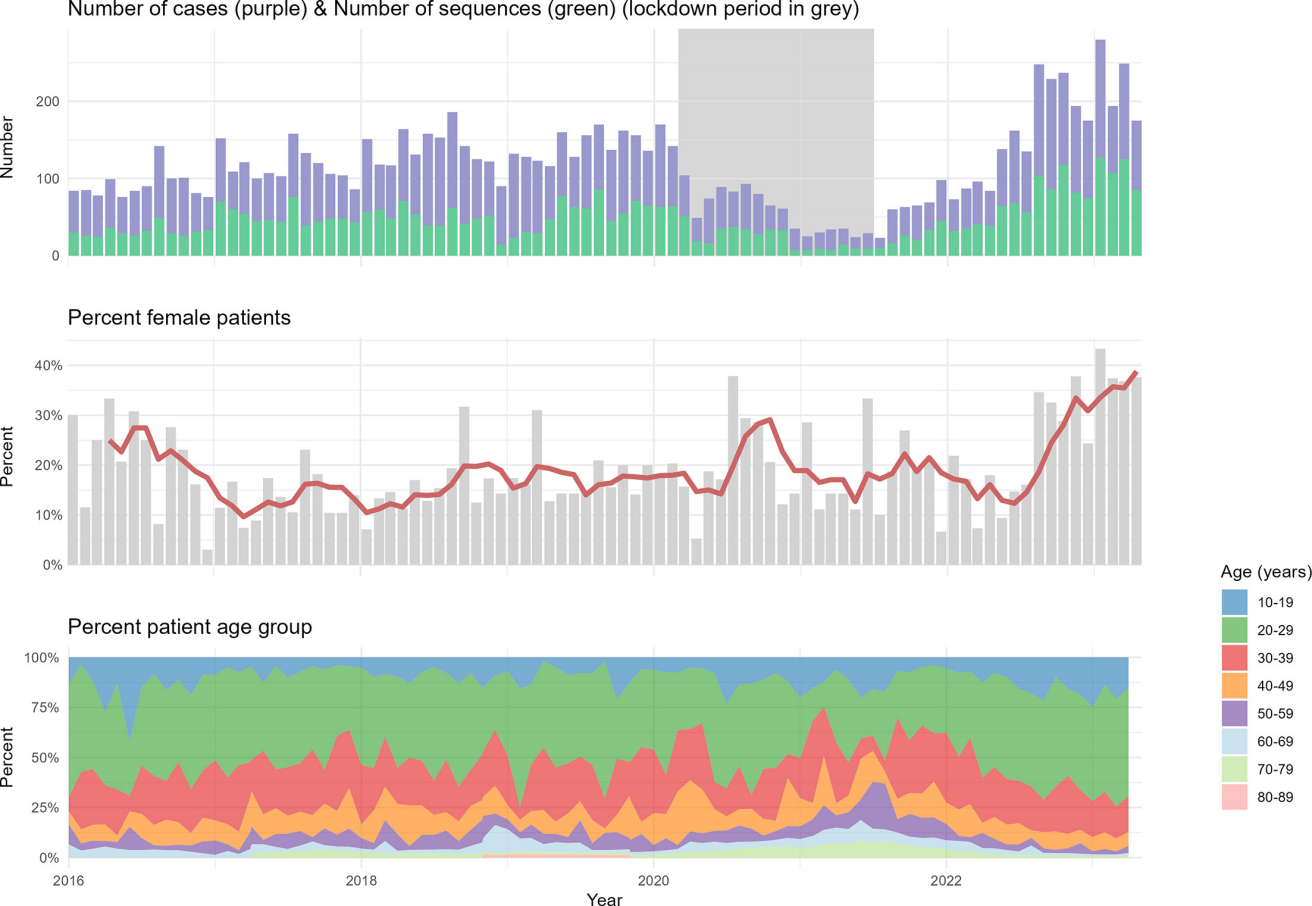
Number of gonorrhoea cases (MSIS database) and number of sequences processed at NIPH (grey square indicates period of lockdown), per cent female patients per month (with rolling mean in red, window size=4 months) (MSIS database) and patient age in brackets of 10 years.

The 4,096 *N*. *gonorrhoeae* whole-genome sequences represented 39% of the cases of gonorrhoea registered in the MSIS during the period and included 92% of all culture-positive cases submitted to NIPH. The number of registered cases and the number of sequenced isolates per month of isolation are shown in Fig. 1. A slight increase over the years was observed in the number of cases reported to MSIS from 2016 until the onset of the COVID-19-associated lockdown in March 2020. The incidence was drastically reduced during the period of non-pharmaceutical interventions targeting SARS-CoV-2 transmission, but following the opening of the Norwegian society around July 2021, the incidence rebounded to a level well above pre-lockdown levels (Fig. 1).

The proportion of female cases was relatively low (average 21%) for the whole study period. However, a significant increase in the proportion of female cases was observed throughout the study period (R^2^=0.09, *P*<0.01; Figs 1 and S1). The median age of the patients was 29 years, and it remained relatively unchanged through the period. A shift towards a larger proportion of older patients (over 50 years) was observed during the lockdown, while after the reopening of the society, there was a trend towards younger patients (median 27 years of age) (Fig. 1). The majority (60.2%) of the isolates were sampled from the urethra, vagina or cervix uteri, a smaller proportion from the anus (23.5%) and pharynx (11.9%), and the remaining isolates (4.5%) were sampled from other locations (such as eye and knee effusions) or unspecified.

**Fig. 2.**
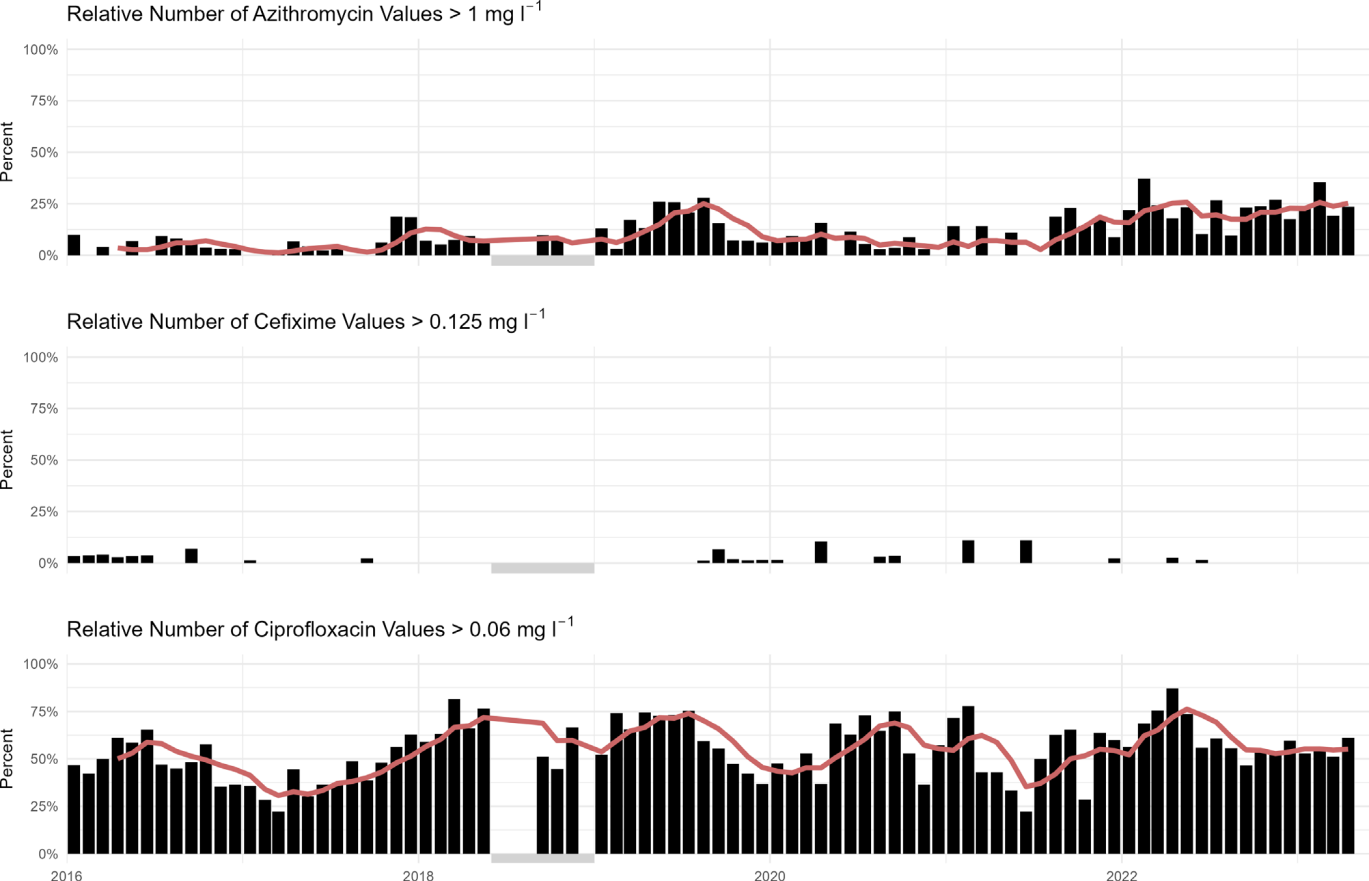
Fraction of cases resistant to AZM, CFM and CIP by months (with rolling mean in red, window size=4 months). A grey square indicates a period with lacking/inconsistently sampled phenotypic data.

Except for a short period in 2018, the isolates underwent phenotypic antibiotic susceptibility testing. Overall, 13% of the tested isolates were resistant to AZM, 1% to CFM and 54% to CIF (Fig. 2). A significant trend towards increased AZM resistance over time was observed (R^2^=0.32, *P*<0.01; Fig. S2). A single isolate resistant to CRO (MIC=0.25 mg l^−1^) was observed in 2019.

**Fig. 3.**
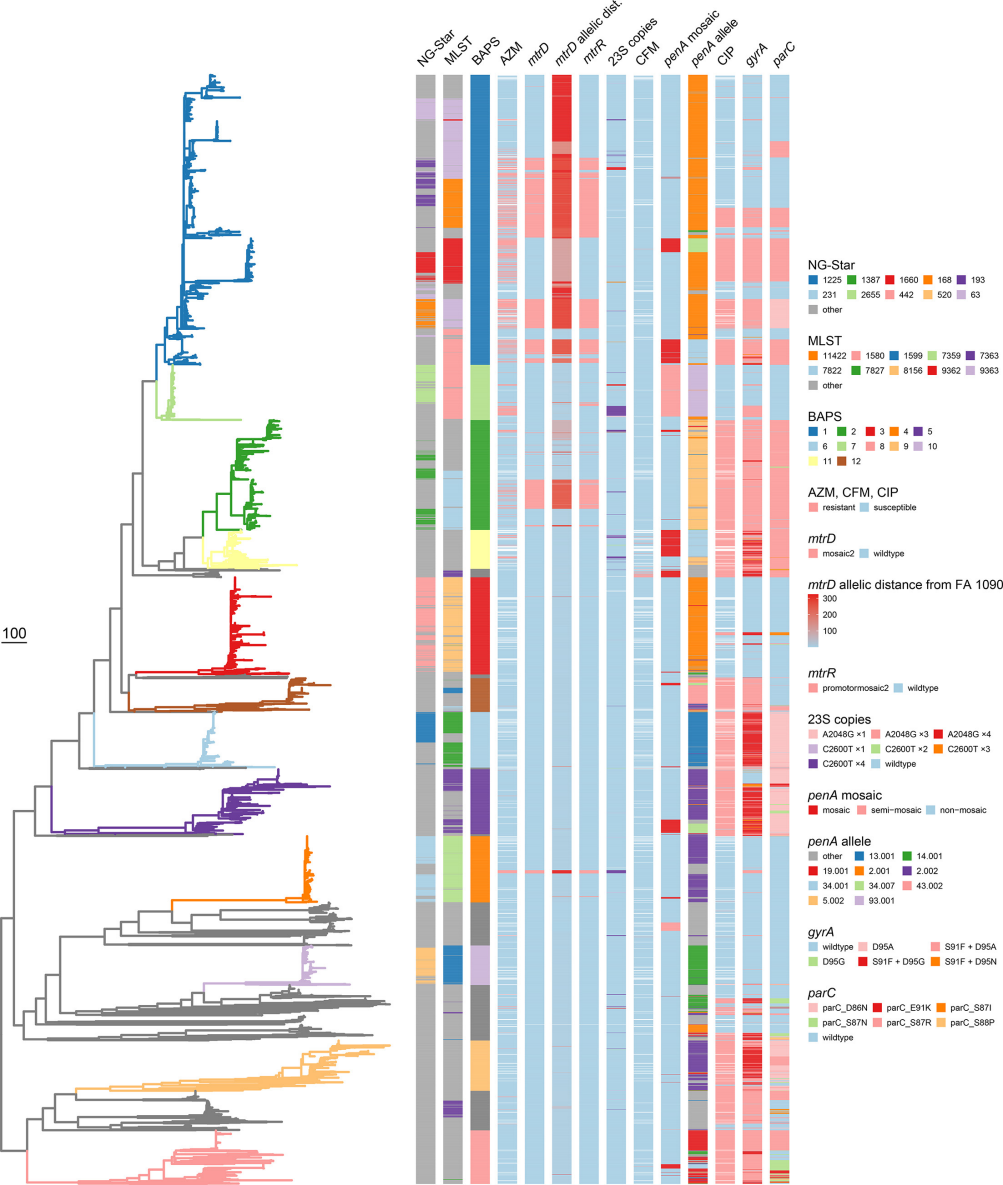
Recombination-corrected maximum-likelihood phylogeny. Branch colour defined by BAPS clusters. The 10 most abundant NG-Star and MLST types are shown, and only the BAPS cluster with ≥10 isolates is shown. Grey bars indicate ‘other’ (NG-Star, MLST and BAPS). Phenotypic AMR using the EUCAST breakpoints is shown for AZM, CFM and CIP. Missing phenotypic AMR data are indicated with white space. AMR determinants are shown with presence or absence (‘wildtype’), and the *mtrD* analysis shows the allelic distance to the reference FA 1090 (see methods for details). The ten most abundant *penA* alleles are shown, and the remaining are shown as ‘other’. Branch lengths show the number of point mutations (SNPs).

To delineate phylogenetically relevant clusters among the 4,096 *N*. *gonorrhoeae* sequences, we identified genetic clusters in the whole-genome SNP-based phylogeny using fastBAPS [21] (referred to as ‘BAPS clusters’). A total of 41 BAPS clusters were identified. Twelve BAPS clusters that consisted of more than 100 isolates and comprised a total of 86% of the 4,096 isolates were numbered consecutively from the largest to the smallest. BAPS 1, the largest cluster, comprised 26% of the isolates (Fig. 3). The 29 BAPS clusters, each comprising fewer than 100 isolates and including a total of 568 isolates, were pooled together and labelled ‘Other’. Among these 29 BAPS clusters, 15 comprised less than 10 isolates, and 5 comprised only a single isolate.

**Fig. 4.**
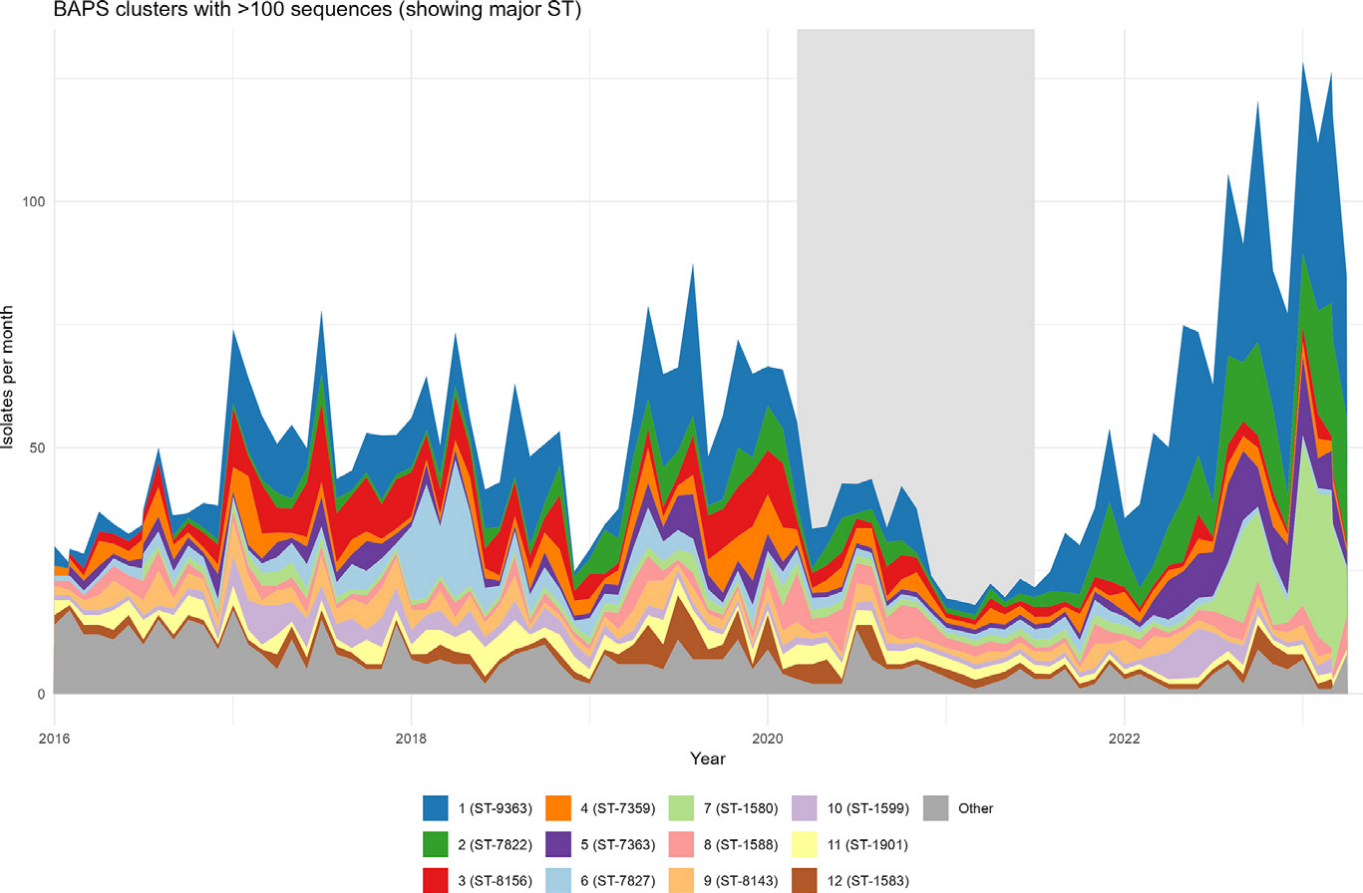
BAPS clusters with >100 sequences, with major STs within each cluster in parentheses. See also Table 1.

The delineation of some BAPS clusters was almost perfectly mirrored by the MLST assignment, whereas other clusters contained several STs at relatively high proportions (Table 1). BAPS 1 included ST-9363 (36%), ST-11422 (17%), ST-9362 (16%) and ST-1580 (11%); BAPS 2 included ST-7822 (51%) and ST-10314 (24%); and BAPS 5 was made up of ST-7363 (49%) and ST-1587 (35%) (Table 1 and Fig. 3). Isolates of the ten most numerous STs were usually assigned to separate BAPS clusters, except for ST-1580 isolates which were split between BAPS 1 (*n*=118) and BAPS 7 (*n*=196), and ST-7363 isolates, which were predominantly in BAPS 5 but with some isolates assigned to other small BAPS clusters. While the top 12 BAPS clusters were seen continuously throughout the study period (Fig. 4), their relative frequency varied. BAPS 1, which dominated overall, was found at relatively low frequency during the lockdown period. The lockdown period was characterized by few, but genetically diverse, isolates. BAPS 6, which mainly comprised ST-7827 isolates (78.0%), was the dominant cluster in the first quarter of 2018 but greatly reduced after 2020. Following the end of the lockdown, BAPS 1, 2, 5 and 7 expanded notably, whereas BAPS 3 and 4 remained at very low levels (Fig. 4).

**Table 1.** BAPS clusters with more >100 isolates and corresponding metadata

BAPS clusters (level 1)	Total no. of sequences in BAPS cluster	% Female	% <30 years	% Resistance AZM	% Resistance CFM	% Resistance CIP	Main ST(s)	No. of isolates (% in BAPS cluster)
1	1,071	13	50	37.1	<0.1	42.5	ST-9363 ST-1422 ST-9362 ST-1580	388 (36) 178 (17) 169 (16) 118 (11)
2	406	13	51	12.3	0	99.0	ST-7822 ST-10314	208 (51) 96 (24)
3	359	7	44	0	0	5.4	ST-8156	316 (88)
4	245	45	67	4.9	0	0.4	ST-7359	238 (97)
5	240	25	55	0.9	0	97.9	ST-7363 ST-1587	117 (49) 85 (35)
6	205	8	50	0	0	99.4	ST-7827	160 (78)
7	204	69	96	20.3	0	0.5	ST-1580	196 (96)
8	198	14	42	0	0.5	100	ST-1588	128 (65)
9	186	13	44	0.6	0	97.1	ST-8143	84 (45)
10	146	8	49	0	0	1.6	ST-1599	141 (97)
11	144	41	60	15.2	5.4	100	ST-1901	109 (76)
12	124	8	38	0.9	0	96.6	ST-1583	73 (59)
Other (29 clusters)	568	30	60	2.3	3.6	44.3	ST-7363	84 (15)
Total	4096	21	54	12.5	0.7	54.1	ST-9363	388 (10)

Average pairwise nucleotide differences per site, π [28], per month, were found to fluctuate throughout the study period (Fig. S3). π did not significantly increase during the period of lockdown restrictions (Kruskal–Wallis Bonferroni method, *P*>0.05) but significantly decreased as the society reopened and the restrictions were lifted (*P*<0.001, Fig. S4). Similarly, the Watterson estimator, θ [29], which is a measure of segregating sites corrected for the number of sequences, was calculated per month. Relatively stable fractions of polymorphic sites were seen from 2016 to early 2022, but a notable (but not statistically significant) increase occurred in the second half of 2022 (Fig. S3). The number of different BAPS clusters per month decreased in March 2020 to June 2021 compared to levels before (Kruskal–Wallis Bonferroni method, *P*<0.001) and after (*P*<0.05, Fig. S4) the lockdown.

Three BAPS clusters included isolates from a higher proportion of female patients than the overall average (21%): BAPS 4 (45%), 7 (69%) and 11 (41%) (Table 1). Isolates belonging to these BAPS clusters were also predominantly affecting young patients (<30 years): BAPS 4 (67%), 7 (96%) and 11 (60%) (Table 1). BAPS 3, 6, 10 and 12 clusters, in contrast, were rarely found to be affecting females (7%, 8%, 8% and 8%, respectively) (Table 1). Isolates of BAPS 12 were found in relatively older patients; 62% were older than 30 years old, compared to 54% overall (Table 1).

BAPS 7, which was disproportionately identified in young women, is also relatively new in Norway – with peak number of cases in January 2023 (Fig. S5). BAPS 7 (*n*=204) was almost exclusively made up by isolates identified as ST-1580 (96%). Additional ST-1580 isolates (*n*=118) were found in BAPS 1 (Table 1), a phylogenetically closely related BAPS cluster (Figs.3  5). We evaluated whether the ST-1580 belonging to BAPS 7 formed an exclusive Norwegian cluster by including all ST-1580 available on PathogenWatch (as of 27 May 2024) containing country of origin and date of sample. Isolates from the global collection were interspersed among Norwegian isolates, demonstrating multiple imports of ST-1580 belonging to both BAPS 1 and BAPS 7 (Fig. S6).

**Fig. 5.**
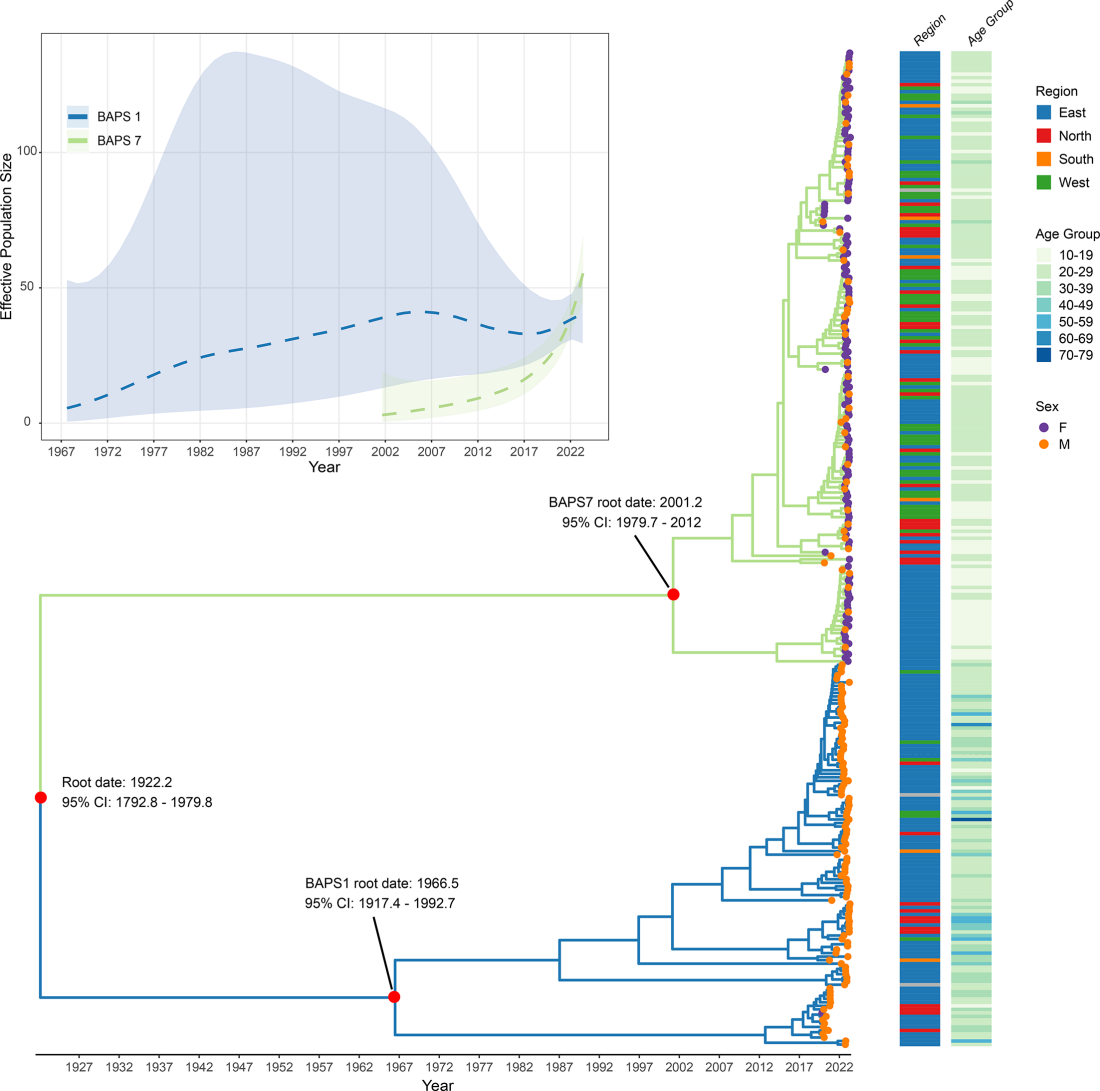
Time-scaled phylogeny of all Norwegian ST-1580 isolates and the estimated effective population size (inset). Tip label colours correspond to patient sex (female/male), Norwegian region of admitting hospital/clinic and age groups represented for each isolate. The delimitation between BAPS cluster 1 (blue) and 7 (green) is indicated.

In the Norwegian ST-1580, Gubbins identified 89.2% of SNPs to be inside of recombination tracts, representing a recombination-to-mutation ratio of 8.25. The recombination-filtered phylogeny of the ST-1580 sequences from Norway exhibited a clear temporal signal; when the tree was rooted based on the best root-to-tip correlation, the R^2^ value was 0.35, with a corresponding root-to-tip distance and date correction of 0.59 (Fig. S9). The relaxed negative binomial clock was the best-fitting molecular clock model, and the estimated mutation rate was 5.38 (95% CI: 4.19–6.76) mutations per genome per year, which translates to 2.49×10⁻⁶ (95% CI: 1.94×10⁻⁶–3.13×10⁻⁶) mutations per site per year, and overlaps with other estimates from gonococci [30]. The estimated root date of the Norwegian ST-1580 phylogeny was 1922 with a credibility interval (95% CI: 1793–1980) (Fig. 5). In comparison to the older clade BAPS 1 (estimated root 1967, 95% CI: 1917–1993), BAPS 7 is estimated to be relatively young, 2001.2 (95% CI: 1980–2012) (Fig. 5). BAPS 1 and BAPS 7 delineate two closely related clades in the phylogeny associated with different demographic groups. Using the midpoint of reported age groups (10–20, 20–30, … 70–79), the average age in BAPS 7 was 31.9 years and in BAPS 1 was 21.3 years. BAPS 7 was associated with a higher fraction of isolates from the West of the country (25.5% vs. 5.1%), a lower fraction from the East (54.6% vs. 78.0%) and a modestly higher contribution from the North (14.8% vs. 11.0%) (Fig. 5). The effective population size of BAPS 7 supports a relatively recent population expansion, while BAPS 1’s effective population size increased through 1970–1990 and has remained relatively constant since (although the 95% credibility intervals indicate uncertainty with respect to the trajectory of BAPS 1) (Fig. 5).

The percentage of AZM resistance varied between BAPS clusters, from 0% in BAPS 3, 6, 8 and 9 up to 37.1% in BAPS 1 (Table 1). The increase of AZM resistance in Norway can possibly be attributed to the rise of BAPS 1 in the period when CIP resistance was rare in BAPS 4 (0.4%), BAPS 7 (0.5%) and BAPS 10 (1.4%), while it was over 90% in BAPS 2, 5, 8, 9 and 12. BAPS 1 was found with an intermediate level of CIP resistance, 42.5% (Table 1). A few isolates were CFM resistant, 1 isolate each in BAPS 1 and 8, 6 isolates in BAPS 11 (4.2%) and 19 isolates in different smaller BAPS clusters labelled ‘Other’ (13 isolates typed as ST-7363) (Table 1). The single CRO-resistant isolate belonged to a smaller BAPS cluster labelled ‘Other’ (ST-7363).

The AMR status of the isolates was predicted *in silico* from the sequences using PathogenWatch. High sensitivity and specificity were observed for CFM (93% and 92%) and CIP (99% and 95%) predictions, while for AZM, the specificity was high (91%), but the sensitivity was lower (77%). The CIP *in silico* predictions and observations were largely overlapping (except for a small cluster in BAPS 1), whereas the *in silico* predictions failed to identify all the observed AZM-resistant isolates (Table S1). Specific AMR determinants were also characterized independently of PathogenWatch by blasting assemblies against suitable allele databases (see methods). Among 511 isolates phenotypically resistant to AZM, 395 (77.3%) harboured a 23S mutation (A2059G, C261T) (median MIC=16 mg l^−1^), *mtrD* mosaic2 (median MIC=2 mg l^−1^) or *mtrR* promoter mosaic2 (median MIC=2 mg l^−1^) predicted by PathogenWatch (Fig. 3). Of the 3,297 phenotypically susceptible AZM isolates, PathogenWatch predicted 282 isolates (8.6%) with a 23S rDNA mutation (A2059G, C261T) (median MIC=1 mg l^−1^), *mtrD* mosaic2 (median MIC=1 mg l^−1^) or *mtrR* promoter mosaic2 (median MIC=1 mg l^−1^), and the median MIC values of these isolates are close to the EUCAST breakpoint >1 mg l^−1^. Comparing the SNP distances of *mtrD* alleles to the FA 1090 reference could highlight additional isolates that could harbour mosaic *mtrD* undetected with PathogenWatch (Fig. S7). Identified *mtrD* mosaic2 isolates with PathogenWatch corresponded to isolates with SNP distances in the range of 137–322 relative to FA 1090 (Table S1); henceforth, 137 SNPs were used as a cut-off for calling *mtrD* mosaicism. Following this cut-off, most of the SNP-inferred mosaic isolates corresponded to those identified with PathogenWatch (Fig. S7). Other isolates with a high number of SNPs relative to FA 1090 were not identified as mosaic with PathogenWatch, such as 46 and 295 isolates with an SNP distance of 169 and 323; these isolates were found to be susceptible to AZM (median MIC=1 mg l^−1^) (Fig. S7). Furthermore, the reference-based alignment analysis identified 148 isolates with 23S mutations A2048G or C2600T compared to the 122 isolates predicted with PathogenWatch. From the new analysis, 23 isolates were found with <3 allelic copies and AZM susceptibility (median MIC=1 mg l^−1^), and 124 isolates were found with ≥3 allelic copies and AZM resistance (median MIC=16 mg l^−1^) (Fig. S7).

In BAPS 1, the majority of the AZM-resistant isolates carried the *mtrD mosaic2* and *mtrR*-promoter mosaic (262 out of 378), indicating mosaicism across the *mtrRCDE* region. A small subset (13 out of 1,071) of the BAPS 1 isolates exhibited very high MIC (256 mg l^−1^), all of which carried both *mtrD* mosaic2 and *mtrR-*promoter mosaic2, as well as 23S rDNA mutations (Table S1). BAPS 7 (also including ST-1580 isolates) carried no *mtrD* mosaic2, and only a few *mtrR*-promoter mosaic2, most of which did not correspond to observed AZM resistance (Table S1). Most of the AZM-resistant isolates in BAPS 7 carried 23S rDNA mutations (38 out of 41) (Table S1).

The analysis of *penA* alleles revealed all CFM-resistant isolates as variants 10.001, 34.001, 34.007, 101.001 and 22.001. CFM resistance could only be observed in a fraction of the isolates in the variants 10.001 (18 out of 38), 34.001 (6 out of 171), 34.007 (1 out of 92) and 22.001 (1 out of 46) (Table S1 and Fig. S8). CIP resistance determination by PathogenWatch is based on mutations in *gyrA* and *parC*, comparing that the individual mutations for this dataset revealed that *gyrA* mutations alone would have greatly improved the specificity (100%) and sensitivity (99%) of the phenotypic CIP resistance (Fig. 3 and Table S1).

## Discussion

In this study, we analysed the gonococcal epidemiological landscape in Norway over an 8-year period spanning the COVID-19 pandemic, using whole-genome sequencing of the isolates from nearly 40% of the reported cases. The constant rise in case numbers in the pre-pandemic years was abruptly interrupted by the lockdown and travel restrictions, but a rebound occurred after reopening of the society to an incidence level surpassing that in 2019. The total number of reported cases in 2023 was 75% higher than that in 2019 (MSIS). Similar post-pandemic rebounds of gonorrhoea case rates have been observed in several countries [31,32].

We used Bayesian analysis of population structure to group the 4,096 isolates into 41 clusters, of which the 12 largest contained 86% of the isolate collection. We observed clear changes in the distribution of the BAPS clusters over time, most significantly the near disappearance post-pandemic of BAPS 6 (ST-7827) which was responsible for an outbreak in 2018 [8] and the dramatic increase of BAPS 7 (ST-1580) among young women. Interestingly, gonorrhoea cases caused by the predominant BAPS cluster, BAPS 1, which included numerous STs, also increased post-pandemic, with ST-1580 as a major contributor. The expansion of ST-1580 in post-pandemic Norway (Fig. 5), following multiple importation events (belonging to both BAPS 1 and 7, see Fig. S6), may also have been driven by transmission of BAPS 7 among young adults of both sexes. In addition to the expansion of the polyphyletic ST-1580 (BAPS 1 and 7), increased incidence of BAPS 2 and 5 was found following the lift of the restrictions. These two clusters, corresponding to the globally abundant ST-7822 and ST-7363, exhibited less AZM resistance (12.3 and 0.9%, respectively), and with patient characteristics suggesting MSM dissemination.

Towards the end of the study period, we observed an increased proportion of younger patients (10–29 years old) and, from the summer of 2022, a sharp increase in the proportion of female patients. This shift was driven in large part by the spread of BAPS 7 in the heterosexual population. In the Netherlands, the COVID-19 pandemic was associated with a slight reduction in the ST diversity and a shift in the dominant ST, ST-8156, to ST-9362 during the lockdown [33]. In contrast, we observed an increase in ST diversity during the lockdown, followed by a sharp decrease in the months following the reopening of the society. In Australia, large core genome MLST clusters persisted through the pandemic, while in the post-pandemic period, some clusters were observed to expand, especially in heterosexual patients [34]. Sporadic import cases during the period of lockdown restrictions may have ensured a relatively high nucleotide diversity (π) throughout the pandemic, whereas the expansion of a restricted number of BAPS clusters, possibly driven by local transmission, likely drove the reduction in π following the pandemic (Figs S3 and S4). In parallel, this expansion, representing a post-bottleneck recovery [35,37], allowed for the accumulation of diversity, likely explaining the increase in the number of segregating sites (θ) observed since the beginning of 2022 (Figs S3 and S4). Evolutionary bottlenecks have been observed in other places; for example, in Australia, both decreased diversity and transmission were observed during the pandemic [34]. A comprehensive retrospective study in Europe revealed overall reduced diversity across the region during the pandemic, with a subsequent increase in clonality [38]. Reduced importation of novel strains was suggested as a possible driver of these observations [38].

Though our data do not contain information on the transmission routes, we find some BAPS clusters (i.e. 3, 6, 10 and 12) that conform to traditional demographics corresponding to MSM transmission, namely a low proportion of young and female patients. When social distancing measures were lifted, new patterns of transmission occurred, with a sharp increase in the proportion of cases among young women, especially associated with a particular genotype. This trend continued in 2023, with over a doubling of the number of cases in women compared to 2022, reaching nearly the number of cases in MSM [39]. AMR patterns of the isolates exhibited a clear link to the phylogenetic structure of the population (Fig. 3). Whether resistance may act as a potential driver for the spread of certain BAPS clusters remains undetermined, and some BAPS clusters (e.g. BAPS 7) expanding post-pandemic did not exhibit elevated AMR markers, whereas others (e.g. BAPS 2 and 5) exhibited AMR resistance (e.g. CIP). The general increasing trend in AZM resistance throughout the period mirrors patterns observed in other countries [38,40]. Compared to PathogenWatch, the analysis of allelic distance of *mtrD* alleles relative to FA 1090 did not identify additional mosaic alleles associated with AZM resistance (Figs 3 and S7). Furthermore, as 23S rDNA exists in multiple copies across individual genomes, PathogenWatch may not identify 23S mutations when these are present in a minority of alleles. We identified a minority of allele mutations (<3 copies) in 23 isolates (not detected with PathogenWatch), and 33% of these minority allele mutations were AZM resistant (Fig. S7). Most CFM-resistant isolates carried the *penA* alleles 10.001 or 34.001 (Fig. S8). In the case of CIP resistance determination, the specificity and sensitivity were greatly improved if only accounting for *gyrA* mutations.

We observed a clear impact of the COVID-19 pandemic on the epidemiology of *N. gonorrhoeae* in Norway. The non-pharmaceutical interventions imposed by the Norwegian government to stop the spread of SARS-CoV-2 dramatically reduced the number of cases of gonorrhoea. As restrictions were lifted, social interactions and travelling resumed, increasing gonorrhoea transmission and likely also the import of new strains. New transmission patterns emerged, requiring intervention and information from the health authorities to attempt to control the gonococcal epidemic. The emergence of AMR and the potential for treatment failure make continuous epidemiological surveillance of *N. gonorrhoeae* a top priority, particularly in the post-pandemic society.

## Supplementary material

10.1099/mgen.0.001479 Uncited Supplementary Material 1.

10.1099/mgen.0.001479 Uncited Table S1.

10.1099/mgen.0.001479 Uncited Table S2.

## Abbreviations

AMR: antimicrobial resistance
ARC: additive RC
AZM: azithromycin
cARC: continuous additive RC
CFM: cefixime
CIP: ciprofloxacin
CRO: ceftriaxone
EUCAST: European Committee for Antimicrobial Susceptibility Testing
MCMC: Markov Chain Monte Carlo
MSIS: Norwegian Surveillance System for Communicable Diseases
MSM: men who have sex with men
NIPH: Norwegian Institute of Public Health
RC: relaxed clock
ST: sequence type
