# Supplementary material for: Genome analysis of Neisseria gonorrhoeae in Norway, 2016–2023, reveals shifting epidemiology in the wake of the COVID-19 pandemic
Source: Microb Genom. 2025 Sep 11;11(9):001479. doi: 10.1099/mgen.0.001479 (PMC12426203; doi:10.1099/mgen.0.001479)
Supplement: Uncited Supplementary Material 1. [file mgen-11-01479-s001.pdf]

Supplementary material

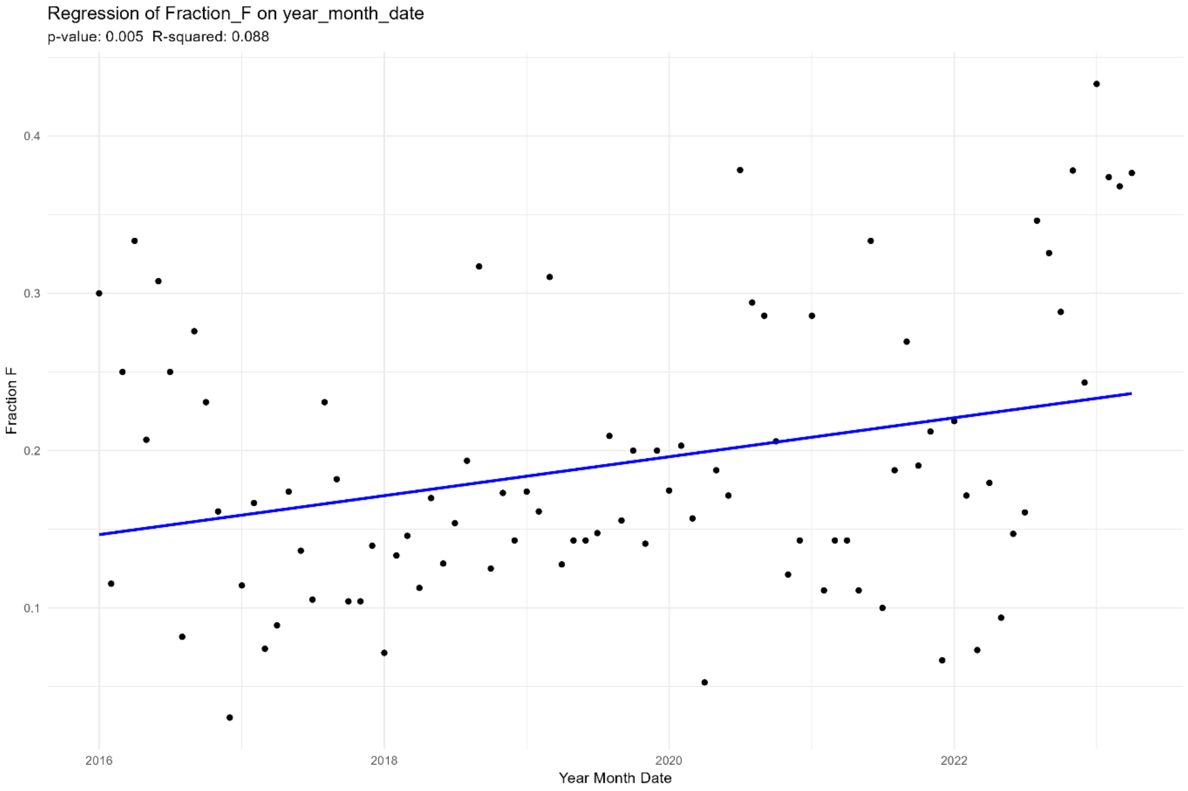

Fig. S1 – Regression analysis of female fraction by date

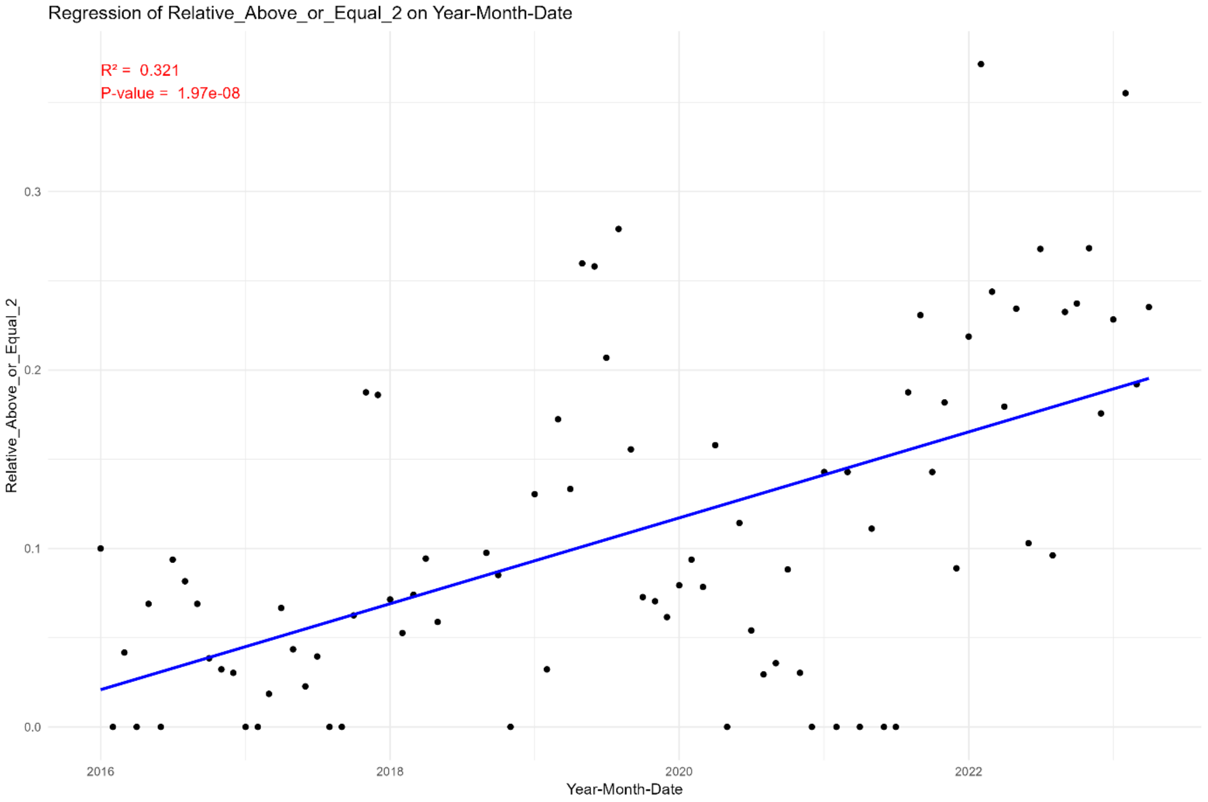

Fig. S2 – Regression analysis of azithromycin resistance by date

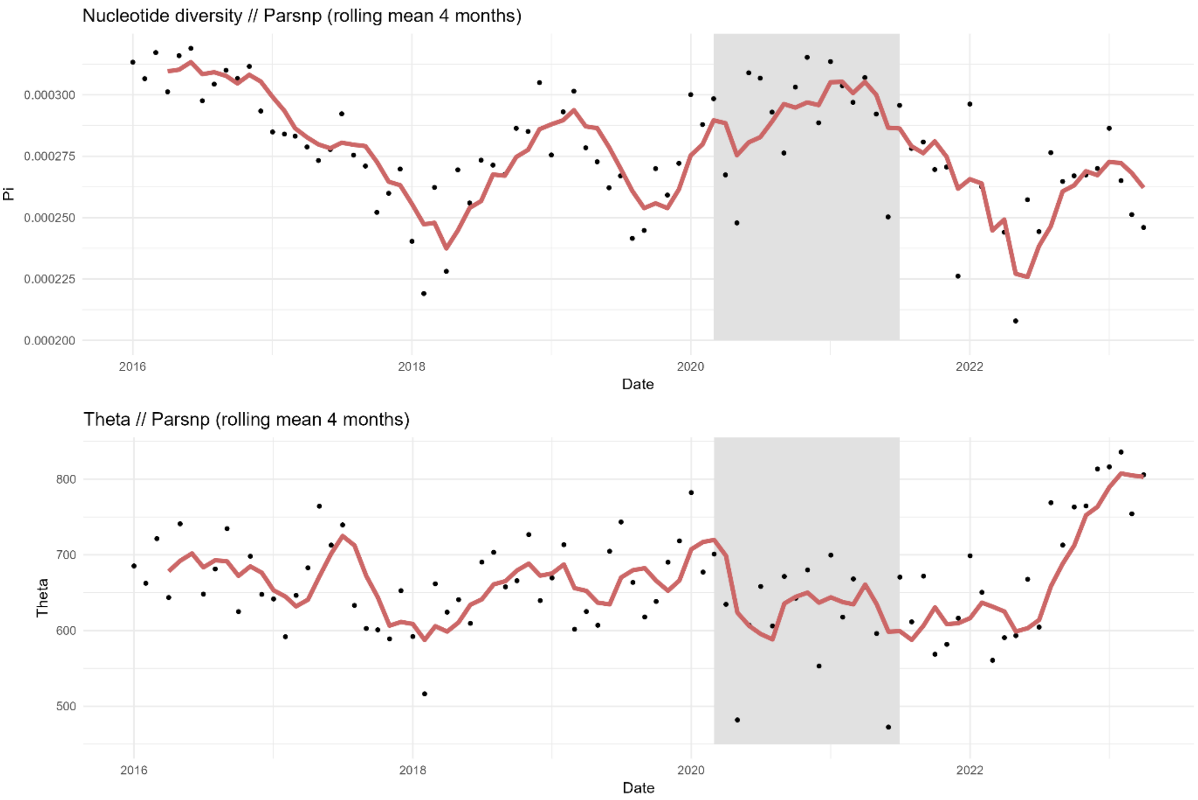

Fig. S3 – Average nucleotide diversity (π) and the number of segregating sites (θ) per month

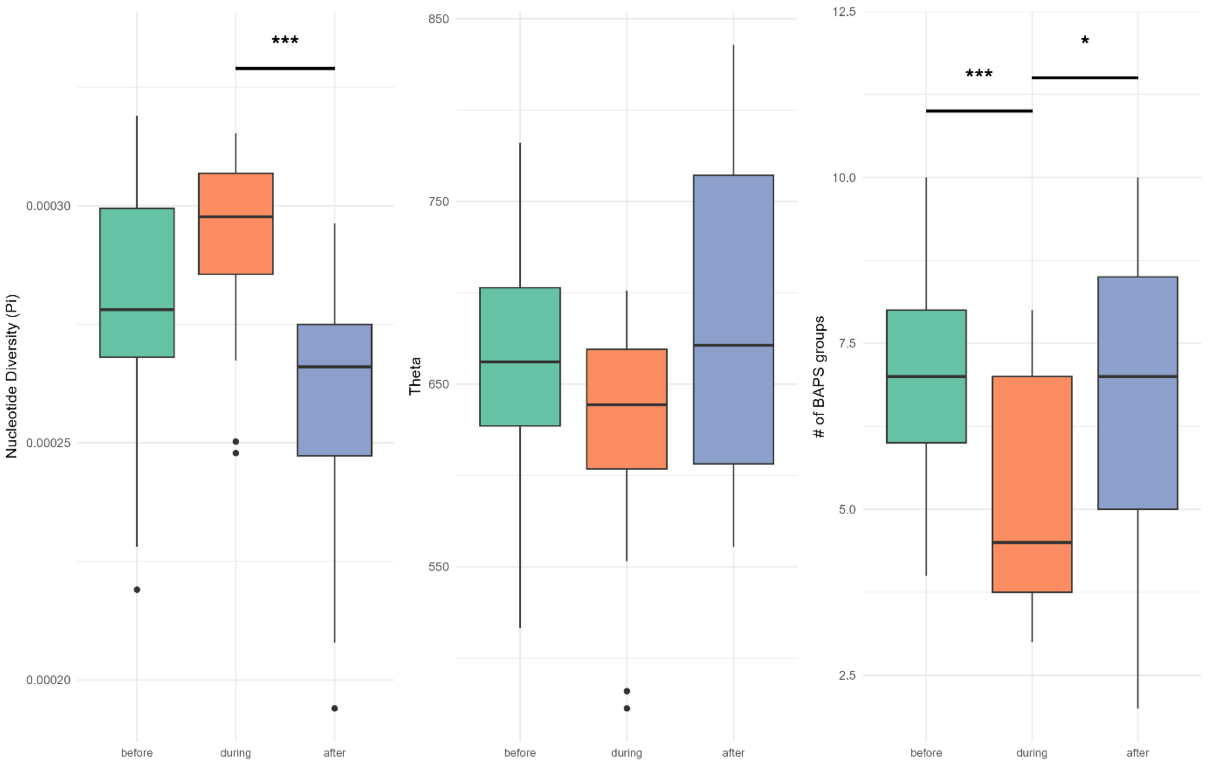

Fig. S4 – Boxplots of the periods before, during (March 2020 – June 2021) and after lockdown restrictions, average nucleotide diversity by month (π) (left) and the Watterson estimator by month (θ) (middle) and number of BAPS groups by month (right). Statistically significant pairwise comparison using Dunn (1964) Kruskal-Wallis multiple comparison adjusted p-values with the Bonferroni method shown as * $p<0.05$, *** $p<0.001$

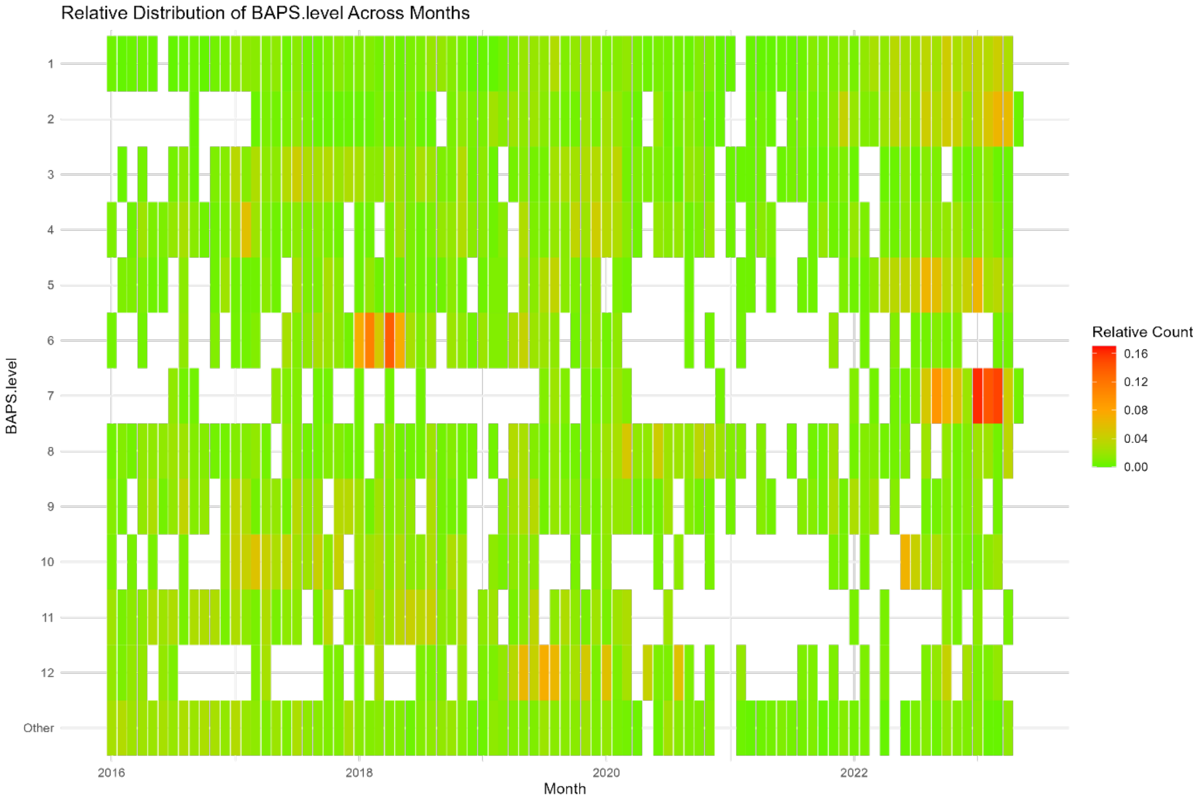

Fig S5 – Tiled heat map, showing the relative proportion of each BAPS cluster throughout the study period.

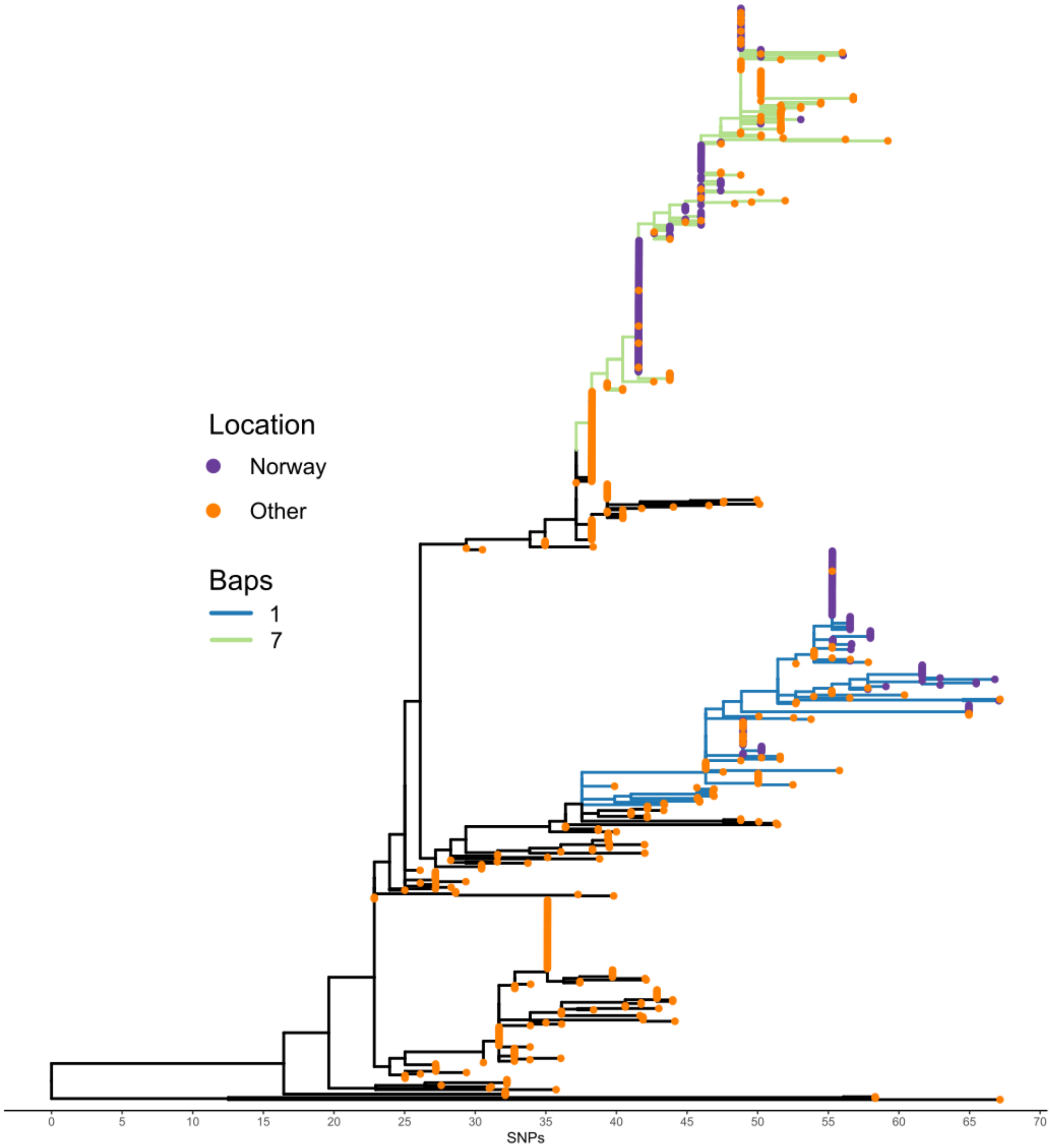

Fig S6 - Phylogeny of ST-1580. Collection and comparison of ST-1580 from PathogenWatch (criteria: known year and known Country, accessed 27th May 2024). Alignment obtained with ParSNP + Gubbins. Branch lengths show number of point mutations (SNPs) – scale bar shows 10 SNPs. Purple tips indicate Norwegian isolates, orange tips indicate non-Norwegian isolates. Green branches indicate isolates in the BAPS 1 cluster, blue branches indicate isolates in the BAPS 7 cluster.

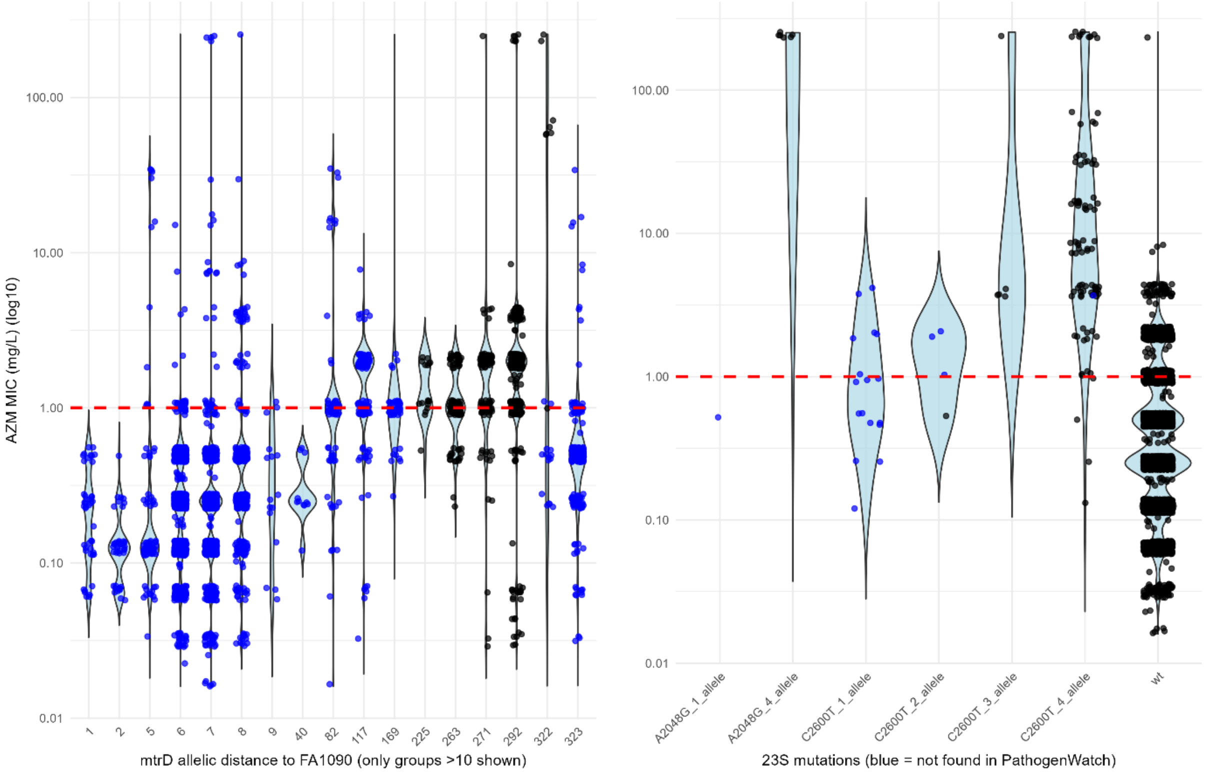

Fig. S7 – Azithromycin (AZM) MIC shown for different analyses, allelic distance of alignment of *mtrD* to the reference sequence FA1090 (only allelic distance with >10 cases are shown, blue dots indicate assemblies not called as *mtrD* mosaic with PathogenWatch) (left), and 23S rDNA mutations and copy number (blue dots indicate 23S rDNA mutations not found with PathogenWatch) (right). Red dashed line indicates the ECOFF 1 mg/L.

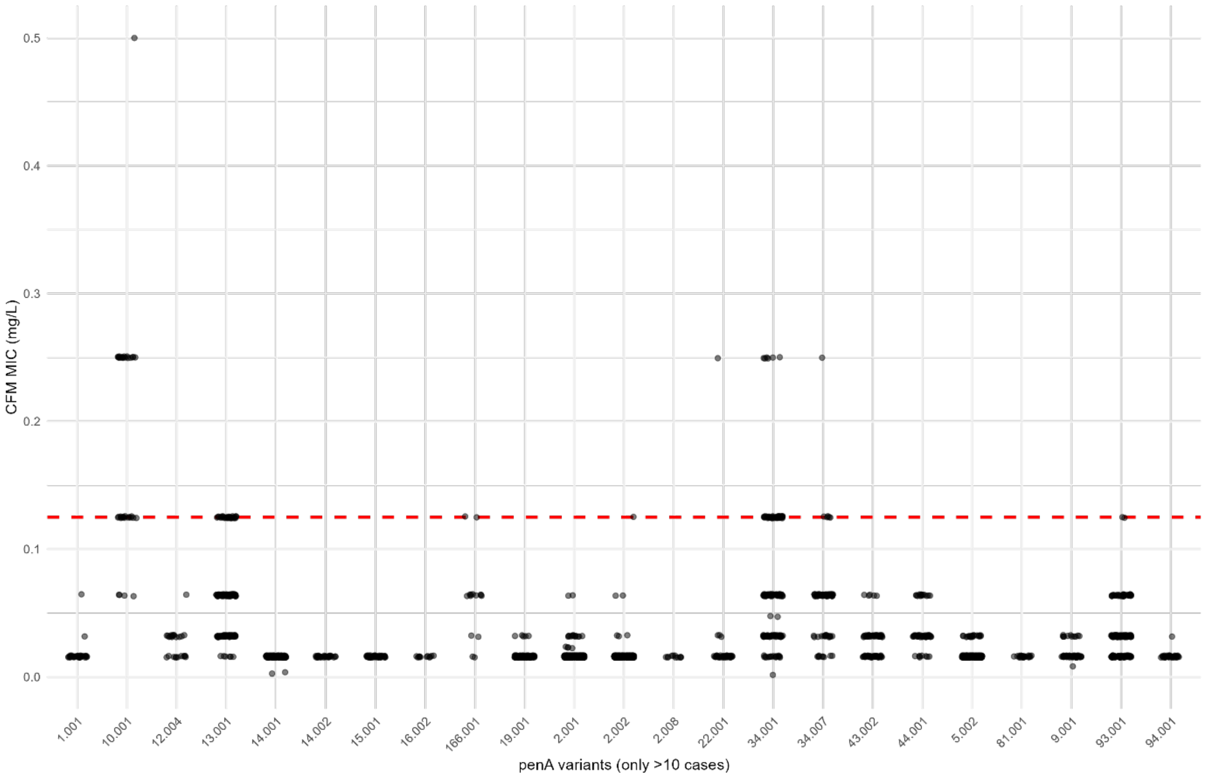

Fig. S8 – Cefixime (CFM) MIC shown penA variants (only variants >10 cases are shown). Red dashed line indicates MIC breakpoint R>0.125 mg/L.

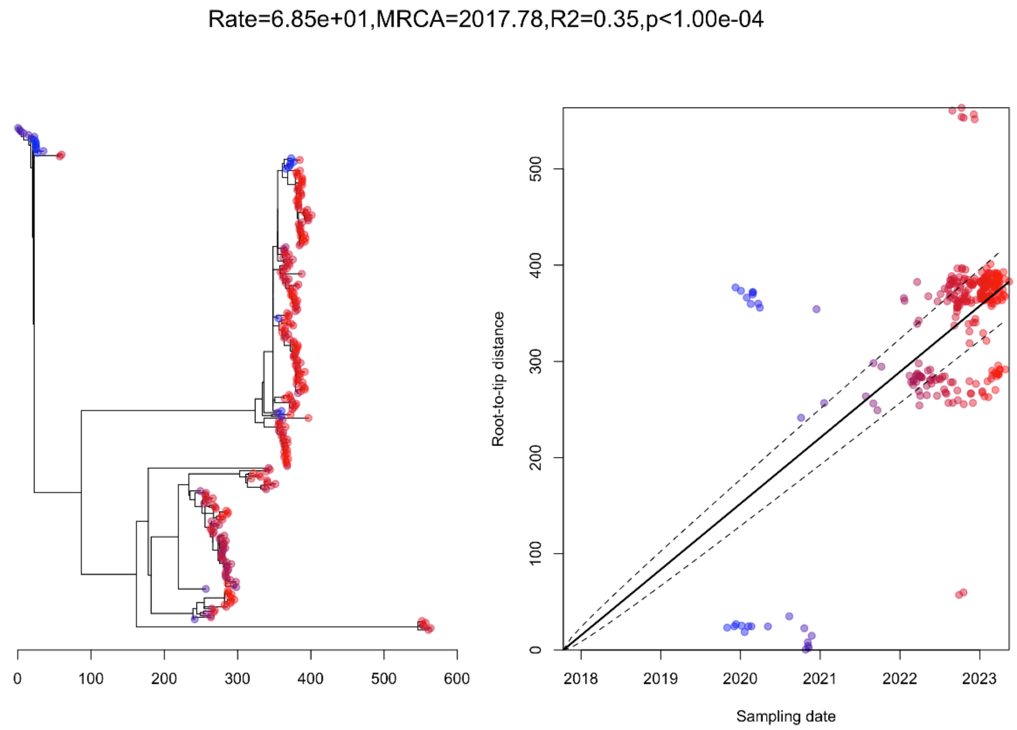

Fig. S9 – Temporal signal of the Norwegian ST-1580 alignment using Snippy and Gubbins (with treebuilder IQTree) assessed using root-to-tip regression analysis in BactDating.
