# Supplementary material for: Genome analysis of Neisseria gonorrhoeae in Norway, 2016–2023, reveals shifting epidemiology in the wake of the COVID-19 pandemic
Source: Microb Genom. 2025 Sep 11;11(9):001479. doi: 10.1099/mgen.0.001479 (PMC12426203; doi:10.1099/mgen.0.001479)
Supplement: Uncited Table S1. [file mgen-11-01479-s002.pdf]

MatchingEntry	MatchingEntryPrefix	SampleAccession	StudyAccession	Age	Sex	Region	Region	Material	Location	Sample_Date	Azitromycin	Cefixime	Ceftriakson	Ciprofloxacin	Penicillin.G	Spectinomycin	Tetracyklin	Gentamicin	Beta-laktamase.prod	MLST	BAPS.level.1	Level.1	NGstar_ST	Azithromycin	Ceftriaxone	Cefixime	Ciprofloxacin	Penicillin	Sulfonamides	Spectinomycin	Tetracycline	Variants	Acquired		Mtr_allele_dist_from_FA1090
518613.fasta	518613	SAMEA5608227	PRJEB32435	28	M	Trøndelag	North	Sekret	Urethra	2016-01-29	4	0,016	0,004	0,004	0,125	16	0,5	NA	NA	9362	1	18	64	RESISTANT	NOT_FOUND	NOT_FOUND	NOT_FOUND	RESISTANT	RESISTANT	NOT_FOUND	INTERMEDIATE	23S_rDNA_c2597tfolP_R228SmtrR_A39TpenA_ins346DrpsJ_V57M	blaTEM		323
22GB001231.fasta	22GB001231	SAMEA117567711	PRJEB83897	30	F	Trøndelag	North	Sekret	Urethra	2022-09-26	16	0,016	0,016	0,004	0,125	16	1	8	Negativ	9363	1	18	1964	RESISTANT	NOT_FOUND	NOT_FOUND	RESISTANT	INTERMEDIATE	RESISTANT	NOT_FOUND	NOT_FOUND	23S_rDNA_c2597tfolP_R228SmtrR_A39TparC_S87RpenA_ins346D		0	323
22GB001450.fasta	22GB001450	SAMEA117567831	PRJEB83897	22	F	Agder	South	Sekret	Vagina	2022-11-01	16	0,016	0,016	0,004	0,125	16	0,5	8	Negativ	9363	1	18	1964	RESISTANT	NOT_FOUND	NOT_FOUND	RESISTANT	INTERMEDIATE	RESISTANT	NOT_FOUND	INTERMEDIATE	23S_rDNA_c2597tfolP_R228SmtrR_A39TparC_S87RpenA_ins346DrpsJ_V57M		0	323
628731.fasta	628731	SAMEA6533581	PRJEB32435	19	M	NA	NA	SEKRET	URETRA	2018-05-09	NA	NA	NA	NA	NA	NA	NA	NA	NA	9363	1	18	1964	RESISTANT	NOT_FOUND	NOT_FOUND	RESISTANT	INTERMEDIATE	RESISTANT	NOT_FOUND	INTERMEDIATE	23S_rDNA_c2597tfolP_R228SmtrR_A39TparC_S87RpenA_ins346DrpsJ_V57M		0	323
20GB001016.fasta	20GB001016	SAMEA117566797	PRJEB83897	21	M	Nordland	North	Sekret	Urethra	2020-06-16	8	0,016	0,004	0,004	0,125	8	1	NA	Ikke påvist	9362	1	18	64	RESISTANT	NOT_FOUND	NOT_FOUND	NOT_FOUND	INTERMEDIATE	RESISTANT	NOT_FOUND	INTERMEDIATE	23S_rDNA_c2597tfolP_R228SmtrR_A39TpenA_ins346DrpsJ_V57M		0	323
20GB001036.fasta	20GB001036	SAMEA117566807	PRJEB83897	19	F	Vestland	West	Sekret	Cervix uter	2020-06-23	16	0,016	0,016	0,004	0,125	16	1	NA	Ikke påvist	9362	1	18	64	RESISTANT	NOT_FOUND	NOT_FOUND	NOT_FOUND	INTERMEDIATE	RESISTANT	NOT_FOUND	INTERMEDIATE	23S_rDNA_c2597tfolP_R228SmtrR_A39TpenA_ins346DrpsJ_V57M		0	323
20GB001078.fasta	20GB001078	SAMEA117566825	PRJEB83897	19	M	Oslo	East	Sekret	Urethra	2020-07-13	32	0,016	0,004	0,004	0,064	16	0,25	NA	Ikke påvist	9362	1	18	64	RESISTANT	NOT_FOUND	NOT_FOUND	NOT_FOUND	INTERMEDIATE	RESISTANT	NOT_FOUND	INTERMEDIATE	23S_rDNA_c2597tfolP_R228SmtrR_A39TpenA_ins346DrpsJ_V57M		0	323
20GB001185.fasta	20GB001185	SAMEA117566877	PRJEB83897	19	F	Viken	East	Sekret	Cervix uter	2020-08-24	8	0,016	0,016	0,004	0,125	16	1	NA	Ikke påvist	9362	1	18	64	RESISTANT	NOT_FOUND	NOT_FOUND	NOT_FOUND	INTERMEDIATE	RESISTANT	NOT_FOUND	INTERMEDIATE	23S_rDNA_c2597tfolP_R228SmtrR_A39TpenA_ins346DrpsJ_V57M		0	323
517960.fasta	517960	SAMEA5608223	PRJEB32435	26	F	Vestfold og Telemark	East	Sekret	Cervix uter	2016-01-05	4	0,032	0,008	0,004	0,125	16	0,5	NA	NA	9362	1	18	64	RESISTANT	NOT_FOUND	NOT_FOUND	NOT_FOUND	INTERMEDIATE	RESISTANT	NOT_FOUND	INTERMEDIATE	23S_rDNA_c2597tfolP_R228SmtrR_A39TpenA_ins346DrpsJ_V57M		0	323
518630.fasta	518630	SAMEA5608228	PRJEB32435	28	F	Vestfold og Telemark	East	Sekret	Vagina	2016-01-27	4	0,016	0,004	0,004	0,125	16	0,5	NA	NA	9362	1	18	64	RESISTANT	NOT_FOUND	NOT_FOUND	NOT_FOUND	INTERMEDIATE	RESISTANT	NOT_FOUND	INTERMEDIATE	23S_rDNA_c2597tfolP_R228SmtrR_A39TpenA_ins346DrpsJ_V57M		0	323
20GB000679.fasta	20GB000679	SAMEA117566703	PRJEB83897	29	M	Oslo	East	Sekret	Urethra	2020-03-05	1	0,016	0,002	0,004	0,125	8	0,125	NA	Ikke påvist	7359	4	5	85d489d232e	RESISTANT	NOT_FOUND	NOT_FOUND	NOT_FOUND	NOT_FOUND	NOT_FOUND	NOT_FOUND	NOT_FOUND	0	mtrD_mosaic_2mtrR_promoter_mosaic_2		322
22GB000604.fasta	22GB000604	SAMEA117567377	PRJEB83897	25	F	Oslo	East	Ascites	Abdomen	2022-05-18	64	0,016	0,016	0,008	0,25	8	0,25	4	Negativ	7359	4	5	5478	RESISTANT	NOT_FOUND	NOT_FOUND	NOT_FOUND	NOT_FOUND	NOT_FOUND	NOT_FOUND	NOT_FOUND	23S_rDNA_c2597t	mtrD_mosaic_2mtrR_promoter_mosaic_2		322
22GB000769.fasta	22GB000769	SAMEA117567452	PRJEB83897	25	M	Oslo	East	Sekret	Urethra	2022-06-17	256	0,016	0,002	0,004	0,125	4	0,125	8	Negativ	7359	4	5	5478	RESISTANT	NOT_FOUND	NOT_FOUND	NOT_FOUND	NOT_FOUND	NOT_FOUND	NOT_FOUND	NOT_FOUND	23S_rDNA_c2597t	mtrD_mosaic_2mtrR_promoter_mosaic_2		322
22GB001575.fasta	22GB001575	SAMEA117567889	PRJEB83897	21	F	Viken	East	Sekret	Vagina	2022-11-22	64	0,016	0,016	0,008	0,25	8	0,25	8	Negativ	7359	4	5	5478	RESISTANT	NOT_FOUND	NOT_FOUND	NOT_FOUND	NOT_FOUND	NOT_FOUND	NOT_FOUND	NOT_FOUND	23S_rDNA_c2597t	mtrD_mosaic_2mtrR_promoter_mosaic_2		322
23GB000215.fasta	23GB000215	SAMEA117568049	PRJEB83897	35	F	Oslo	East	Sekret	Cervix uter	2023-01-12	256	0,016	0,002	0,002	0,25	8	0,125	NA	Negativ	7359	4	5	5478	RESISTANT	NOT_FOUND	NOT_FOUND	NOT_FOUND	NOT_FOUND	NOT_FOUND	NOT_FOUND	NOT_FOUND	23S_rDNA_c2597t	mtrD_mosaic_2mtrR_promoter_mosaic_2		322
23GB000335.fasta	23GB000335	SAMEA117568110	PRJEB83897	42	M	Oslo	East	Sekret	Urethra	2023-01-30	256	0,016	0,002	0,008	0,25	16	0,125	NA	Negativ	7359	4	5	5478	RESISTANT	NOT_FOUND	NOT_FOUND	NOT_FOUND	NOT_FOUND	NOT_FOUND	NOT_FOUND	NOT_FOUND	23S_rDNA_c2597t	mtrD_mosaic_2mtrR_promoter_mosaic_2		322
23GB000336.fasta	23GB000336	SAMEA117568111	PRJEB83897	44	F	Oslo	East	Sekret	Cervix uter	2023-01-30	256	0,016	0,002	0,004	0,125	16	0,064	NA	Negativ	7359	4	5	5478	RESISTANT	NOT_FOUND	NOT_FOUND	NOT_FOUND	NOT_FOUND	NOT_FOUND	NOT_FOUND	NOT_FOUND	23S_rDNA_c2597t	mtrD_mosaic_2mtrR_promoter_mosaic_2		322
23GB000342.fasta	23GB000342	SAMEA117568114	PRJEB83897	38	F	Oslo	East	Sekret	Cervix uter	2023-02-02	256	0,016	0,002	0,004	0,125	8	0,125	NA	Negativ	7359	4	5	5478	RESISTANT	NOT_FOUND	NOT_FOUND	NOT_FOUND	NOT_FOUND	NOT_FOUND	NOT_FOUND	NOT_FOUND	23S_rDNA_c2597t	mtrD_mosaic_2mtrR_promoter_mosaic_2		322
23GB000357.fasta	23GB000357	SAMEA117568125	PRJEB83897	38	F	Oslo	East	Sekret	Cervix uter	2023-02-01	64	0,016	0,016	0,008	0,25	8	0,5	NA	Negativ	7359	4	5	5478	RESISTANT	NOT_FOUND	NOT_FOUND	NOT_FOUND	NOT_FOUND	NOT_FOUND	NOT_FOUND	NOT_FOUND	23S_rDNA_c2597t	mtrD_mosaic_2mtrR_promoter_mosaic_2		322
23GB000502.fasta	23GB000502	SAMEA117568192	PRJEB83897	36	F	Oslo	East	Sekret	Cervix uter	2023-02-20	256	0,016	0,002	0,008	0,25	32	0,25	NA	Negativ	7359	4	5	5478	RESISTANT	NOT_FOUND	NOT_FOUND	NOT_FOUND	NOT_FOUND	NOT_FOUND	NOT_FOUND	NOT_FOUND	23S_rDNA_c2597t	mtrD_mosaic_2mtrR_promoter_mosaic_2		322
23GB000766.fasta	23GB000766	SAMEA117568313	PRJEB83897	45	M	Vestfold og Telemark	East	Sekret	Urethra	2023-03-24	64	0,016	0,016	0,008	0,25	16	0,25	NA	Negativ	7359	4	5	5478	RESISTANT	NOT_FOUND	NOT_FOUND	NOT_FOUND	NOT_FOUND	NOT_FOUND	NOT_FOUND	NOT_FOUND	23S_rDNA_c2597t	mtrD_mosaic_2mtrR_promoter_mosaic_2		322
23GB000867.fasta	23GB000867	SAMEA117568363	PRJEB83897	50	F	Vestfold og Telemark	East	Sekret	Vagina	2023-03-31	64	0,016	0,016	0,008	0,25	16	0,25	NA	Negativ	7359	4	5	5478	RESISTANT	NOT_FOUND	NOT_FOUND	NOT_FOUND	NOT_FOUND	NOT_FOUND	NOT_FOUND	NOT_FOUND	23S_rDNA_c2597t	mtrD_mosaic_2mtrR_promoter_mosaic_2		322
23GB000739.fasta	23GB000739	SAMEA117568298	PRJEB83897	22	M	Viken	East	Sekret	Urethra	2023-03-23	2	0,016	0,016	16	0,5	16	2	NA	Negativ	9363	1	18	168	RESISTANT	NOT_FOUND	NOT_FOUND	RESISTANT	INTERMEDIATE	RESISTANT	NOT_FOUND	INTERMEDIATE	folP_R228SgyrA_D95AgyrA_S91FparC_D86NpenA_ins346DporB1b_A121NporB1b_G120KrpsJ_V57M	mtrD_mosaic_2mtrR_promoter_mosaic_2		302
23GB000743.fasta	23GB000743	SAMEA117568299	PRJEB83897	23	F	Viken	East	Sekret	ukjent	2023-03-23	2	0,016	0,016	16	0,5	16	2	NA	Negativ	9363	1	18	168	RESISTANT	NOT_FOUND	NOT_FOUND	RESISTANT	INTERMEDIATE	RESISTANT	NOT_FOUND	INTERMEDIATE	folP_R228SgyrA_D95AgyrA_S91FparC_D86NpenA_ins346DporB1b_A121NporB1b_G120KrpsJ_V57M	mtrD_mosaic_2mtrR_promoter_mosaic_2		302
23GB000832.fasta	23GB000832	SAMEA117568344	PRJEB83897	24	M	Viken	East	Sekret	Urethra	2023-03-30	4	0,016	0,004	8	1	32	2	NA	Negativ	9363	1	18	168	RESISTANT	NOT_FOUND	NOT_FOUND	RESISTANT	INTERMEDIATE	RESISTANT	NOT_FOUND	INTERMEDIATE	folP_R228SgyrA_D95AgyrA_S91FparC_D86NpenA_ins346DporB1b_A121NporB1b_G120KrpsJ_V57M	mtrD_mosaic_2mtrR_promoter_mosaic_2		302
23GB001025.fasta	23GB001025	SAMEA117568430	PRJEB83897	30	F	Oslo	East	Sekret	Vagina	2023-04-28	0,125	0,016	0,002	1	0,25	16	0,5	NA	Negativ	9363	1	18	168	RESISTANT	NOT_FOUND	NOT_FOUND	RESISTANT	INTERMEDIATE	RESISTANT	NOT_FOUND	INTERMEDIATE	folP_R228SgyrA_D95AgyrA_S91FparC_D86NpenA_ins346DporB1b_A121NporB1b_G120KrpsJ_V57M	mtrD_mosaic_2mtrR_promoter_mosaic_2		302
603457.fasta	603457	SAMEA5609120	PRJEB32435	44	M	Oslo	East	Sekret	Urethra	2017-10-25	256	0,016	0,004	0,032	0,5	16	1	NA	NA	9363	1	18	1993	RESISTANT	NOT_FOUND	NOT_FOUND	NOT_FOUND	INTERMEDIATE	RESISTANT	NOT_FOUND	INTERMEDIATE	23S_rDNA_a2045gfolP_R228SpenA_ins346DporB1b_A121NporB1b_G120KrpsJ_V57M	mtrD_mosaic_2mtrR_promoter_mosaic_2		294
[illegible]	[illegible]	[illegible]	[illegible]	[illegible]	[illegible]	[illegible]	[illegible]	[illegible]	[illegible]	[illegible]	[illegible]	[illegible]	[illegible]	[illegible]	[illegible]	[illegible]	[illegible]	[illegible]	[illegible]	[illegible]	[illegible]	[illegible]	[illegible]	[illegible]	[illegible]	[illegible]	[illegible]	[illegible]	[illegible]	[illegible]	[illegible]	[illegible]	[illegible]	[illegible]	[illegible]

671189.fasta	671189	SAMEA6534555	PRJEB32435	37	M	Oslo	East	Sekret	Urethra	2019-08-26	2	0,016	0,008	0,016	0,5	0,016	1	4	Ikke påvist	11422	1	18	193	RESISTANT	NOT_FOUND	NOT_FOUND	NOT_FOUND	INTERMEDIATE	RESISTANT	NOT_FOUND	INTERMEDIATE	folP_R228SpenA_ins346DporB1b_A121NporB1b_G120KrpsJ_V57M	mtrD_mosaic_2mtrR_promoter_mosaic_2	292
671191.fasta	671191	SAMEA6534556	PRJEB32435	56	M	Innlandet	East	Sekret	Hals	2019-08-26	4	0,016	0,004	0,016	0,5	16	1	4	Ikke påvist	11422	1	18	193	RESISTANT	NOT_FOUND	NOT_FOUND	NOT_FOUND	INTERMEDIATE	RESISTANT	NOT_FOUND	INTERMEDIATE	folP_R228SpenA_ins346DporB1b_A121NporB1b_G120KrpsJ_V57M	mtrD_mosaic_2mtrR_promoter_mosaic_2	292
671559.fasta	671559	SAMEA6534580	PRJEB32435	28	M	Rogaland	West	Sekret	Urethra	2019-09-01	1	0,016	0,016	0,008	1	16	1	4	Ikke påvist	9363	1	18	193	RESISTANT	NOT_FOUND	NOT_FOUND	NOT_FOUND	INTERMEDIATE	RESISTANT	NOT_FOUND	INTERMEDIATE	folP_R228SpenA_ins346DporB1b_A121NporB1b_G120KrpsJ_V57M	mtrD_mosaic_2mtrR_promoter_mosaic_2	292
673447.fasta	673447	SAMEA6534607	PRJEB32435	57	F	Vestfold og Telemark	East	Sekret	Vagina	2019-09-27	2	0,016	0,016	0,032	0,5	16	1	8	Ikke påvist	11422	1	18	193	RESISTANT	NOT_FOUND	NOT_FOUND	NOT_FOUND	INTERMEDIATE	RESISTANT	NOT_FOUND	INTERMEDIATE	folP_R228SpenA_ins346DporB1b_A121NporB1b_G120KrpsJ_V57M	mtrD_mosaic_2mtrR_promoter_mosaic_2	292
673528.fasta	673528	SAMEA6534613	PRJEB32435	28	M	Oslo	East	Sekret	Anus	2019-09-26	2	0,016	0,008	0,016	16	16	1	4	Ikke påvist	9363	1	18	193	RESISTANT	NOT_FOUND	NOT_FOUND	NOT_FOUND	INTERMEDIATE	RESISTANT	NOT_FOUND	INTERMEDIATE	folP_R228SpenA_ins346DporB1b_A121NporB1b_G120KrpsJ_V57M	mtrD_mosaic_2mtrR_promoter_mosaic_2	292
676269.fasta	676269	SAMEA6534669	PRJEB32435	44	M	Oslo	East	Sekret	Anus	2019-11-01	0,5	0,016	0,016	0,032	0,125	16	1	4	Ikke påvist	11422	1	18	193	RESISTANT	NOT_FOUND	NOT_FOUND	NOT_FOUND	INTERMEDIATE	RESISTANT	NOT_FOUND	INTERMEDIATE	folP_R228SpenA_ins346DporB1b_A121NporB1b_G120KrpsJ_V57M	mtrD_mosaic_2mtrR_promoter_mosaic_2	292
676288.fasta	676288	SAMEA6534670	PRJEB32435	34	M	Vestland	West	Sekret	Urethra	2019-11-01	1	0,016	0,016	0,016	0,5	8	1	4	Ikke påvist	9363	1	18	193	RESISTANT	NOT_FOUND	NOT_FOUND	NOT_FOUND	INTERMEDIATE	RESISTANT	NOT_FOUND	INTERMEDIATE	folP_R228SpenA_ins346DporB1b_A121NporB1b_G120KrpsJ_V57M	mtrD_mosaic_2mtrR_promoter_mosaic_2	292
679282.fasta	679282	SAMEA6534743	PRJEB32435	53	M	Oslo	East	Sekret	Anus	2019-12-03	1	0,016	0,032	0,032	0,5	16	0,5	8	Ikke påvist	11422	1	18	193	RESISTANT	NOT_FOUND	NOT_FOUND	NOT_FOUND	INTERMEDIATE	RESISTANT	NOT_FOUND	INTERMEDIATE	folP_R228SpenA_ins346DporB1b_A121NporB1b_G120KrpsJ_V57M	mtrD_mosaic_2mtrR_promoter_mosaic_2	292
679284.fasta	679284	SAMEA6534744	PRJEB32435	29	M	Oslo	East	Sekret	Anus	2019-12-03	2	0,016	0,016	0,032	0,5	16	0,5	8	Ikke påvist	11422	1	18	193	RESISTANT	NOT_FOUND	NOT_FOUND	NOT_FOUND	INTERMEDIATE	RESISTANT	NOT_FOUND	INTERMEDIATE	folP_R228SpenA_ins346DporB1b_A121NporB1b_G120KrpsJ_V57M	mtrD_mosaic_2mtrR_promoter_mosaic_2	292
679646.fasta	679646	SAMEA6534750	PRJEB32435	30	M	Oslo	East	Sekret	Anus	2019-12-06	0,5	0,016	0,016	0,016	0,5	8	0,5	4	Ikke påvist	11422	1	18	193	RESISTANT	NOT_FOUND	NOT_FOUND	NOT_FOUND	INTERMEDIATE	RESISTANT	NOT_FOUND	INTERMEDIATE	folP_R228SpenA_ins346DporB1b_A121NporB1b_G120KrpsJ_V57M	mtrD_mosaic_2mtrR_promoter_mosaic_2	292
20GB000980.fasta	20GB000980	SAMEA117566786	PRJEB83897	22	M	Viken	East	Sekret	Anus	2020-06-02	256	0,016	0,002	0,008	0,5	16	0,5	NA	Ikke påvist	11422	1	18	933dd46ed1d	RESISTANT	NOT_FOUND	NOT_FOUND	NOT_FOUND	INTERMEDIATE	RESISTANT	NOT_FOUND	INTERMEDIATE	folP_R228SpenA_ins346DrpsJ_V57M	mtrD_mosaic_2mtrR_promoter_mosaic_2	292
21GB000126.fasta	21GB000126	SAMEA117567008	PRJEB83897	40	M	Rogaland	West	Sekret	Hals	2021-03-08	2	0,016	0,016	0,008	0,25	16	1	NA	Ikke påvist	15248	1	18	2885	RESISTANT	NOT_FOUND	NOT_FOUND	NOT_FOUND	INTERMEDIATE	RESISTANT	NOT_FOUND	INTERMEDIATE	folP_R228SpenA_ins346DrpsJ_V57M	mtrD_mosaic_2mtrR_promoter_mosaic_2	292
21GB000416.fasta	21GB000416	SAMEA117567056	PRJEB83897	24	M	Agder	South	Sekret	Urethra	2021-08-11	2	0,016	0,016	0,008	0,25	8	1	NA	Ikke påvist	11422	1	18	213	RESISTANT	NOT_FOUND	NOT_FOUND	NOT_FOUND	INTERMEDIATE	RESISTANT	NOT_FOUND	INTERMEDIATE	folP_R228SpenA_ins346DrpsJ_V57M	mtrD_mosaic_2mtrR_promoter_mosaic_2	292
21GB000591.fasta	21GB000591	SAMEA117567097	PRJEB83897	20	M	Viken	East	Sekret	Anus	2021-10-04	1	0,016	0,002	0,004	0,25	4	0,25	NA	Ikke påvist	11422	1	18	2885	RESISTANT	NOT_FOUND	NOT_FOUND	NOT_FOUND	INTERMEDIATE	RESISTANT	NOT_FOUND	INTERMEDIATE	folP_R228SpenA_ins346DrpsJ_V57M	mtrD_mosaic_2mtrR_promoter_mosaic_2	292
21GB000608.fasta	21GB000608	SAMEA117567106	PRJEB83897	31	M	Oslo	East	Sekret	Urethra	2021-10-11	2	0,016	0,002	0,004	0,25	8	0,25	NA	Ikke påvist	11422	1	18	2885	RESISTANT	NOT_FOUND	NOT_FOUND	NOT_FOUND	INTERMEDIATE	RESISTANT	NOT_FOUND	INTERMEDIATE	folP_R228SpenA_ins346DrpsJ_V57M	mtrD_mosaic_2mtrR_promoter_mosaic_2	292
21GB000621.fasta	21GB000621	SAMEA117567108	PRJEB83897	39	M	Oslo	East	Sekret	Hals	2021-10-15	2	0,016	0,002	0,002	0,25	8	0,25	NA	Ikke påvist	11422	1	18	213	RESISTANT	NOT_FOUND	NOT_FOUND	NOT_FOUND	INTERMEDIATE	RESISTANT	NOT_FOUND	INTERMEDIATE	folP_R228SpenA_ins346DrpsJ_V57M	mtrD_mosaic_2mtrR_promoter_mosaic_2	292
21GB000644.fasta	21GB000644	SAMEA117567114	PRJEB83897	31	M	Oslo	East	Sekret	Anus	2021-10-25	2	0,016	0,004	0,002	1	8	0,5	NA	Ikke påvist	13527	1	18	2958	RESISTANT	NOT_FOUND	NOT_FOUND	NOT_FOUND	INTERMEDIATE	RESISTANT	NOT_FOUND	INTERMEDIATE	folP_R228SpenA_ins346DrpsJ_V57M	mtrD_mosaic_2mtrR_promoter_mosaic_2	292
21GB000778.fasta	21GB000778	SAMEA117567147	PRJEB83897	33	M	Rogaland	West	Sekret	Anus	2021-11-23	2	0,016	0,016	0,008	0,25	16	1	NA	Ikke påvist	11422	1	18	2885	RESISTANT	NOT_FOUND	NOT_FOUND	NOT_FOUND	INTERMEDIATE	RESISTANT	NOT_FOUND	INTERMEDIATE	folP_R228SpenA_ins346DrpsJ_V57M	mtrD_mosaic_2mtrR_promoter_mosaic_2	292
21GB000800.fasta	21GB000800	SAMEA117567154	PRJEB83897	22	M	Oslo	East	Sekret	Anus	2021-12-02	1	0,016	0,002	0,004	0,25	16	0,25	NA	Ikke påvist	11422	1	18	2885	RESISTANT	NOT_FOUND	NOT_FOUND	NOT_FOUND	INTERMEDIATE	RESISTANT	NOT_FOUND	INTERMEDIATE	folP_R228SpenA_ins346DrpsJ_V57M	mtrD_mosaic_2mtrR_promoter_mosaic_2	292
21GB000803.fasta	21GB000803	SAMEA117567157	PRJEB83897	47	M	Oslo	East	Sekret	Anus	2021-12-02	0,5	0,016	0,002	0,004	0,25	16	0,125	NA	Ikke påvist	11422	1	18	2885	RESISTANT	NOT_FOUND	NOT_FOUND	NOT_FOUND	INTERMEDIATE	RESISTANT	NOT_FOUND	INTERMEDIATE	folP_R228SpenA_ins346DrpsJ_V57M	mtrD_mosaic_2mtrR_promoter_mosaic_2	292
22GB000104.fasta	22GB000104	SAMEA117567221	PRJEB83897	42	M	Agder	South	Sekret	Kjønnsorga	2022-01-18	2	0,016	0,016	0,004	0,25	16	1	NA	Ikke påvist	9363	1	18	213	RESISTANT	NOT_FOUND	NOT_FOUND	NOT_FOUND	INTERMEDIATE	RESISTANT	NOT_FOUND	INTERMEDIATE	folP_R228SpenA_ins346DrpsJ_V57M	mtrD_mosaic_2mtrR_promoter_mosaic_2	292
22GB000109.fasta	22GB000109	SAMEA117567224	PRJEB83897	24	M	Viken	East	Sekret	Cervix uter	2022-01-31	2	0,016	0,002	0,004	0,25	16	0,25	NA	Ikke påvist	9363	1	18	213	RESISTANT	NOT_FOUND	NOT_FOUND	NOT_FOUND	INTERMEDIATE	RESISTANT	NOT_FOUND	INTERMEDIATE	folP_R228SpenA_ins346DrpsJ_V57M	mtrD_mosaic_2mtrR_promoter_mosaic_2	292
22GB000194.fasta	22GB000194	SAMEA117567265	PRJEB83897	41	M	Trøndelag	North	Sekret	Anus	2022-02-28	2	0,016	0,016	0,004	0,25	8	1	NA	Ikke påvist	11422	1	18	2885	RESISTANT	NOT_FOUND	NOT_FOUND	NOT_FOUND	INTERMEDIATE	RESISTANT	NOT_FOUND	INTERMEDIATE	folP_R228SpenA_ins346DrpsJ_V57M	mtrD_mosaic_2mtrR_promoter_mosaic_2	292
22GB000257.fasta	22GB000257	SAMEA117567285	PRJEB83897	19	M	Viken	East	Sekret	Anus	2022-03-17	1	0,016	0,002	0,004	0,25	8	0,5	NA	Ikke påvist	11422	1	18	2885	RESISTANT	NOT_FOUND	NOT_FOUND	NOT_FOUND	INTERMEDIATE	RESISTANT	NOT_FOUND	INTERMEDIATE	folP_R228SpenA_ins346DrpsJ_V57M	mtrD_mosaic_2mtrR_promoter_mosaic_2	292
22GB000279.fasta	22GB000279	SAMEA117567291	PRJEB83897	27	M	Oslo	East	Sekret	Anus	2022-03-22	1	0,016	0,002	0,008	0,25	16	0,5	NA	Ikke påvist	11422	1	18	211	RESISTANT	NOT_FOUND	NOT_FOUND	NOT_FOUND	INTERMEDIATE	RESISTANT	NOT_FOUND	INTERMEDIATE	folP_R228SpenA_ins346DrpsJ_V57M	mtrD_mosaic_2mtrR_promoter_mosaic_2	292
22GB000781.fasta	22GB000781	SAMEA117567458	PRJEB83897	26	M	Oslo	East	Sekret	Anus	2022-06-28	1	0,016	0,004	0,004	0,25	16	0,25	8	Negativ	11422	1	18	211	RESISTANT	NOT_FOUND	NOT_FOUND	NOT_FOUND	INTERMEDIATE	RESISTANT	NOT_FOUND	INTERMEDIATE	folP_R228SpenA_ins346DrpsJ_V57M	mtrD_mosaic_2mtrR_promoter_mosaic_2	292
22GB000830.fasta	22GB000830	SAMEA117567480	PRJEB83897	42	M	Oslo	East	Sekret	Hals	2022-07-04	1	0,016	0,004	0,004	0,25	16	0,25	8	Negativ	11422	1	18	213	RESISTANT	NOT_FOUND	NOT_FOUND	NOT_FOUND	INTERMEDIATE	RESISTANT	NOT_FOUND	INTERMEDIATE	folP_R228SpenA_ins346DrpsJ_V57M	mtrD_mosaic_2mtrR_promoter_mosaic_2	292
22GB001031.fasta	22GB001031	SAMEA117567600	PRJEB83897	25	M	Vestfold og Telemark	East	Sekret	Ureter	2022-08-25	1	0,016	0,004	0,004	0,125	8	0,125	8	Negativ	11422	1	18	211	RESISTANT	NOT_FOUND	NOT_FOUND	NOT_FOUND	INTERMEDIATE	RESISTANT	NOT_FOUND	INTERMEDIATE	folP_R228SpenA_ins346DrpsJ_V57M	mtrD_mosaic_2mtrR_promoter_mosaic_2	292
22GB001142.fasta	22GB001142	SAMEA117567665	PRJEB83897	36	M	Oslo	East	Sekret	Hals	2022-09-12	2	0,016	0,016	0,004	0,25	16	0,5	8	Negativ	11422	1	18	213	RESISTANT	NOT_FOUND	NOT_FOUND	NOT_FOUND	INTERMEDIATE	RESISTANT	NOT_FOUND	INTERMEDIATE	folP_R228SpenA_ins346DrpsJ_V57M	mtrD_mosaic_2mtrR_promoter_mosaic_2	292
22GB001277.fasta	22GB001277	SAMEA117567734	PRJEB83897	30	M	Rogaland	West	Sekret	Anus	2022-10-05	2	0,016	0,016	0,008	0,5	16	1	8	Negativ	11422	1	18	211	RESISTANT	NOT_FOUND	NOT_FOUND	NOT_FOUND	INTERMEDIATE	RESISTANT	NOT_FOUND	INTERMEDIATE	folP_R228SpenA_ins346DrpsJ_V57M	mtrD_mosaic_2mtrR_promoter_mosaic_2	292
22GB001423.fasta	22GB001423	SAMEA117567817	PRJEB83897	28	M	Vestland	West	Sekret	Urethra	2022-10-27	1	0,016	0,004	0,002	0,25	16	0,25	8	Negativ	8134	1	18	2885	RESISTANT	NOT_FOUND	NOT_FOUND	NOT_FOUND	INTERMEDIATE	RESISTANT	NOT_FOUND	INTERMEDIATE	folP_R228SpenA_ins346DrpsJ_V57M	mtrD_mosaic_2mtrR_promoter_mosaic_2	292
22GB001429.fasta	22GB001429	SAMEA117567822	PRJEB83897	28	M	Trøndelag	North	Sekret	Hals	2022-10-26	2	0,016	0,016	0,004	0,25	16	1	8	Negativ	11422	1	18	213	RESISTANT	NOT_FOUND	NOT_FOUND	NOT_FOUND	INTERMEDIATE	RESISTANT	NOT_FOUND	INTERMEDIATE	folP_R228SpenA_ins346DrpsJ_V57M	mtrD_mosaic_2mtrR_promoter_mosaic_2	292
22GB001457.fasta	22GB001457	SAMEA117567834	PRJEB83897	34	M	Oslo	East	Sekret	Anus	2022-10-31	2	0,016	0,004	0,004	0,25	32	1	NA	Negativ	8134	1	18	2885	RESISTANT	NOT_FOUND	NOT_FOUND	NOT_FOUND	INTERMEDIATE	RESISTANT	NOT_FOUND	INTERMEDIATE	folP_R228SpenA_ins346DrpsJ_V57M	mtrD_mosaic_2mtrR_promoter_mosaic_2	292
22GB001499.fasta	22GB001499	SAMEA117567855	PRJEB83897	19	F	Vestland	West	Sekret	Cervix uter	2022-11-11	1	0,016	0,016	0,008	0,25	8	0,5	8	Negativ	11422	1	18	2885	RESISTANT	NOT_FOUND	NOT_FOUND	NOT_FOUND	INTERMEDIATE	RESISTANT	NOT_FOUND	INTERMEDIATE	folP_R228SpenA_ins346DrpsJ_V57M	mtrD_mosaic_2mtrR_promoter_mosaic_2	292
22GB001524.fasta	22GB001524	SAMEA117567868	PRJEB83897	25	M	Oslo	East	Halssekret	Hals	2022-11-11	1	0,016	0,008	0,008	0,5	16	0,25	8	Negativ	11422	1	18	2885	RESISTANT	NOT_FOUND	NOT_FOUND	NOT_FOUND	INTERMEDIATE	RESISTANT	NOT_FOUND	INTERMEDIATE	folP_R228SpenA_ins346DrpsJ_V57M	mtrD_mosaic_2mtrR_promoter_mosaic_2	292
23GB000425.fasta	23GB000425	SAMEA117568159	PRJEB83897	30	M	Oslo	East	Sekret	Anus	2023-02-08	2	0,016	0,004	0,004	0,25	16	0,25	NA	Negativ	11422	1	18	2885	RESISTANT	NOT_FOUND	NOT_FOUND	NOT_FOUND	INTERMEDIATE	RESISTANT	NOT_FOUND	INTERMEDIATE	folP_R228SpenA_ins346DrpsJ_V57M	mtrD_mosaic_2mtrR_promoter_mosaic_2	292
23GB000912.fasta	23GB000912	SAMEA117568373	PRJEB83897	36	M	Oslo	East	Sekret	Anus	2023-04-13	1	0,016	0,002	0,004	0,125	16	0,5	NA	Negativ	11422	1	18	213	RESISTANT	NOT_FOUND	NOT_FOUND	NOT_FOUND	INTERMEDIATE	RESISTANT	NOT_FOUND	INTERMEDIATE	folP_R228SpenA_ins346DrpsJ_V57M	mtrD_mosaic_2mtrR_promoter_mosaic_2	292
594376.fasta	594376	SAMEA5608972	PRJEB32435	69	M	NA	NA	Sekret	Hals	2017-07-28	2	0,016	0,002	0,002	0,125	16	1	NA	NA	9363	1	18	213	RESISTANT	NOT_FOUND	NOT_FOUND	NOT_FOUND	INTERMEDIATE	RESISTANT	NOT_FOUND	INTERMEDIATE	folP_R228SpenA_ins346DrpsJ_V57M	mtrD_mosaic_2mtrR_promoter_mosaic_2	292
641085.fasta	641085	SAMEA6533862	PRJEB32435	32	M	NA	NA	SEKRET	URETRA	2018-10-02	1,5	0,016	0,016	0,002	NA	NA	NA	NA	NA	9363	1	18	1252	RESISTANT	NOT_FOUND	NOT_FOUND	NOT_FOUND	INTERMEDIATE	RESISTANT	NOT_FOUND	INTERMEDIATE	folP_R228SpenA_ins346DrpsJ_V57M	mtrD_mosaic_2mtrR_promoter_mosaic_2	292
641181.fasta	641181	SAMEA6533872	PRJEB32435	29	M	NA	NA	SEKRET	HALS	2018-10-01	2	0,016	0,016	0,002	NA	NA	NA	NA	NA	11422	1	18	213	RESISTANT	NOT_FOUND	NOT_FOUND	NOT_FOUND	INTERMEDIATE	RESISTANT	NOT_FOUND	INTERMEDIATE	folP_R228SpenA_ins346DrpsJ_V57M	mtrD_mosaic_2mtrR_promoter_mosaic_2	292
651461.fasta	651461	SAMEA6534034	PRJEB32435	24	M	Viken	East	Sekret	Urethra	2019-01-22	2	0,016	0,008	0,004	0,5	32	1	NA	NA	9363	1	18	213	RESISTANT	NOT_FOUND	NOT_FOUND	NOT_FOUND	INTERMEDIATE	RESISTANT	NOT_FOUND	INTERMEDIATE	folP_R228SpenA_ins346DrpsJ_V57M	mtrD_mosaic_2mtrR_promoter_mosaic_2	292
664754.fasta	664754	SAMEA6534271	PRJEB32435	42	M	Oslo	East	Sekret	Anus	2019-05-29	2	0,016	0,004	0,004	0,25	16	0,5	4	Ikke påvist	9363	1	18	213	RESISTANT	NOT_FOUND	NOT_FOUND	NOT_FOUND	INTERMEDIATE	RESISTANT	NOT_FOUND	INTERMEDIATE	folP_R228SpenA_ins346DrpsJ_V57M	mtrD_mosaic_2mtrR_promoter_mosaic_2	292
669197.fasta	669197	SAMEA6534493	PRJEB32435	22	M	Oslo	East	Sekret	Hals	2019-08-01	1	0,016	0,004	0,004	0,5	16	0,5	4	Ikke påvist	11422	1	18	2885	RESISTANT	NOT_FOUND	NOT_FOUND	NOT_FOUND	INTERMEDIATE	RESISTANT	NOT_FOUND	INTERMEDIATE	folP_R228SpenA_ins346DrpsJ_V57M	mtrD_mosaic_2mtrR_promoter_mosaic_2	292
22GB001090.fasta	22GB001090	SAMEA117567640	PRJEB83897	28	M	Viken	East	Sekret	Urethra	2022-09-02	2	0,016	0,002	0,016	0,5	32	1	8	Negativ	9363	1	18	5383	RESISTANT	NOT_FOUND	NOT_FOUND	NOT_FOUND	INTERMEDIATE	RESISTANT	NOT_FOUND	INTERMEDIATE	folP_R228SporB1b_A121NporB1b_G120KrpsJ_V57M	mtrD_mosaic_2mtrR_promoter_mosaic_2	292
22GB001112.fasta	22GB001112	SAMEA117567652	PRJEB83897	27	M	Oslo	East	Sekret	Hals	2022-09-05	2	0,016	0,002	0,016	0,5	16	0,5	8	Negativ	9363	1	18	5383	RESISTANT	NOT_FOUND	NOT_FOUND	NOT_FOUND	INTERMEDIATE	RESISTANT	NOT_FOUND	INTERMEDIATE	folP_R228SporB1b_A121NporB1b_G120KrpsJ_V57M	mtrD_mosaic_2mtrR_promoter_mosaic_2	292
613847.fasta	613847	SAMEA6533442	PRJEB32435	38	M	Oslo	East	Sekret	Anus	2018-01-15	1	0,016	0,008	8	0,5	16	1	NA	NA	13292	1	18	30b21ceaacc3	RESISTANT	NOT_FOUND	NOT_FOUND	NOT_FOUND	INTERMEDIATE	RESISTANT	NOT_FOUND	INTERMEDIATE	folP_R228SmtrR_A39TpenA_ins346DporB1b_A121NporB1b_G120KrpsJ_V57M	mtrD_mosaic_2	292
637779.fasta	637779	SAMEA6533791	PRJEB32435	38	M	NA	NA	SEKRET	ANUS	2018-08-31	NA	NA	NA	NA	NA	NA	NA	NA	NA	11422	1	18	193	RESISTANT	NOT_FOUND	NOT_FOUND	NOT_FOUND	RESISTANT	RESISTANT	NOT_FOUND	INTERMEDIATE	folP_R228SpenA_ins346DporB1b_A121NporB1b_G120KrpsJ_V57M	blaTEMmtrD_mosaic_2mtrR_promoter_mosaic_2	292
22GB000803.fasta	22GB000803	SAMEA117567471	PRJEB83897	43	M	Vestfold og Telemark	East	Sekret	Hals	2022-06-30	1	0,016	0,016	0,004	0,5	8	16	8	Negativ	16655	1	18	0e5918c55b1	RESISTANT	NOT_FOUND	NOT_FOUND	NOT_FOUND	INTERMEDIATE	RESISTANT	NOT_FOUND	RESISTANT	folP_R228SmtrR_A39TpenA_ins346DrpsJ_V57M	mtrD_mosaic_2mtrR_promoter_mosaic_2tetM	271
22GB000274.fasta	22GB000274	SAMEA117567286	PRJEB83897	43	M	Oslo	East	Sekret	Urethra	2022-03-22	256	0,032	0,008	4	0,25	8	0,5	NA	Ikke påvist	1580	1	18	83396ab896a	RESISTANT	NOT_FOUND	RESISTANT	RESISTANT	INTERMEDIATE	RESISTANT	NOT_FOUND	INTERMEDIATE	23S_rDNA_c2597tfolP_R228SgyrA_D95AgyrA_S91FparC_S87RpenA_G545SpenA_I312MpenA_V316TrpsJ_V57M	mtrD_mosaic_2mtrR_promoter_mosaic_2	271
21GB000489.fasta	21GB000489	SAMEA117567068	PRJEB83897	24	M	Viken	East	Sekret	Urethra	2021-09-02	0,5	0,032	0,004	0,5	0,125	8	0,25	NA	Ikke påvist	1580	1	18	4457	RESISTANT	NOT_FOUND	RESISTANT	RESISTANT	INTERMEDIATE	RESISTANT	NOT_FOUND	INTERMEDIATE	folP_R228SgyrA_D95AgyrA_S91FparC_S87RpenA_G545SpenA_I312MpenA_V316TrpsJ_V57M	mtrD_mosaic_2mtrR_promoter_mosaic_2	271
21GB000597.fasta	21GB000597	SAMEA117567101	PRJEB83897	29	M	Oslo	East	Sekret	Hals	2021-10-08	1	0,016	0,004	2	0,25	8	0,125	NA	Ikke påvist	1580	1	18	4457	RESISTANT	NOT_FOUND	RESISTANT	RESISTANT	INTERMEDIATE	RESISTANT	NOT_FOUND	INTERMEDIATE	folP_R228SgyrA_D95AgyrA_S91FparC_S87RpenA_G545SpenA_I312MpenA_V316TrpsJ_V57M	mtrD_mosaic_2mtrR_promoter_mosaic_2	271
22GB000150.fasta	22GB000150	SAMEA117567247	PRJEB83897	40	M	Oslo	East	Sekret	Urethra	2022-02-14	2	0,032	0,004	2	0,25	16	0,25	NA	Ikke påvist	1580	1	18	4457	RESISTANT	NOT_FOUND	RESISTANT	RESISTANT	INTERMEDIATE	RESISTANT	NOT_FOUND	INTERMEDIATE	folP_R228SgyrA_D95AgyrA_S91FparC_S87RpenA_G545SpenA_I312MpenA_V316TrpsJ_V57M	mtrD_mosaic_2mtrR_promoter_mosaic_2	271
22GB000174.fasta	22GB000174	SAMEA117567257	PRJEB83897	23	M	Oslo	East	Sekret	Urethra	2022-02-21	2	0,032	0,008	2	0,25	16	0,5	NA	Ikke påvist	1580	1	18	4457	RESISTANT	NOT_FOUND	RESISTANT	RESISTANT	INTERMEDIATE	RESISTANT	NOT_FOUND	INTERMEDIATE	folP_R228SgyrA_D95AgyrA_S91FparC_S87RpenA_G545SpenA_I312MpenA_V316TrpsJ_V57M	mtrD_mosaic_2mtrR_promoter_mosaic_2	271
22GB000198.fasta	22GB000198	SAMEA117567269	PRJEB83897	32	M	Oslo	East	Sekret	Anus	2022-03-02	1	0,016	0,004	2	0,25	16	0,5	NA	Ikke påvist	1580	1	18	4457	RESISTANT	NOT_FOUND	RESISTANT	RESISTANT	INTERMEDIATE	RESISTANT	NOT_FOUND	INTERMEDIATE	folP_R228SgyrA_D95AgyrA_S91FparC_S87RpenA_G545SpenA_I312MpenA_V316TrpsJ_V57M	mtrD_mosaic_2mtrR_promoter_mosaic_2	271
22GB000201.fasta	22GB000201	SAMEA117567270	PRJEB83897	47	M	Møre og Romsdal	West	Sekret		2022-03-02	2	0,064	0,016	4	0,25	8	1	NA	Ikke påvist	1580	1	18	4457	RESISTANT	NOT_FOUND	RESISTANT	RESISTANT	INTERMEDIATE	RESISTANT	NOT_FOUND	INTERMEDIATE	folP_R228SgyrA_D95AgyrA_S91FparC_S87RpenA_G545SpenA_I312MpenA_V316TrpsJ_V57M	mtrD_mosaic_2mtrR_promoter_mosaic_2	271
22GB000218.fasta	22GB000218	SAMEA117567271	PRJEB83897	35	M	Rogaland	West	Sekret	Urethra	2022-03-07	2	0,032	0,008	4	0,5	16	0,5	NA	Ikke påvist	1580	1	18	4457	RESISTANT	NOT_FOUND	RESISTANT	RESISTANT	INTERMEDIATE	RESISTANT	NOT_FOUND	INTERMEDIATE	folP_R228SgyrA_D95AgyrA_S91FparC_S87RpenA_G545SpenA_I312MpenA_V316TrpsJ_V57M	mtrD_mosaic_2mtrR_promoter_mosaic_2	271
22GB000255.fasta	22GB000255	SAMEA117567283	PRJEB83897	54	M	Oslo	East	Sekret	Urethra	2022-03-15	0,5	0,032	0,002	2	0,064	8	0,25	NA	Ikke påvist	1580	1	18	4457	RESISTANT	NOT_FOUND	RESISTANT	RESISTANT	INTERMEDIATE	RESISTANT	NOT_FOUND	INTERMEDIATE	folP_R228SgyrA_D95AgyrA_S91FparC_S87RpenA_G545SpenA_I312MpenA_V316TrpsJ_V57M	mtrD_mosaic_2mtrR_promoter_mosaic_2	271
22GB000256.fasta	22GB000256	SAMEA117567284	PRJEB83897	39	M	Viken	East	Sekret	Anus	2022-03-17	0,5	0,016	0,004	2	0,032	8	0,25	NA	Ikke påvist	1580	1	18	4457	RESISTANT	NOT_FOUND	RESISTANT	RESISTANT	INTERMEDIATE	RESISTANT	NOT_FOUND	INTERMEDIATE	folP_R228SgyrA_D95AgyrA_S91FparC_S87RpenA_G545SpenA_I312MpenA_V316TrpsJ_V57M	mtrD_mosaic_2mtrR_promoter_mosaic_2	271
22GB000277.fasta	22GB000277	SAMEA117567289	PRJEB83897	31	M	Oslo	East	Sekret	Anus	2022-03-21	2	0,032	0,004	4	0,25	16	0,5	NA	Ikke påvist	1580	1	18	4457	RESISTANT	NOT_FOUND	RESISTANT	RESISTANT	INTERMEDIATE	RESISTANT	NOT_FOUND	INTERMEDIATE	folP_R228SgyrA_D95AgyrA_S91FparC_S87RpenA_G545SpenA_I312MpenA_V316TrpsJ_V57M	mtrD_mosaic_2mtrR_promoter_mosaic_2	271
22GB000319.fasta	22GB000319	SAMEA117567295	PRJEB83897	29	M	Oslo	East	Sekret	Urethra	2022-03-28	1	0,016	0,004	2	0,125	8	0,5	NA	Ikke påvist	1580	1	18	4457	RESISTANT	NOT_FOUND	RESISTANT	RESISTANT	INTERMEDIATE	RESISTANT	NOT_FOUND	INTERMEDIATE	folP_R228SgyrA_D95AgyrA_S91FparC_S87RpenA_G545SpenA_I312MpenA_V316TrpsJ_V57M	mtrD_mosaic_2mtrR_promoter_mosaic_2	271
22GB000323.fasta	22GB000323	SAMEA117567299	PRJEB83897	41	M	Viken	East	Sekret	Urethra	2022-03-28	0,5	0,016	0,008	1	0,25	8	0,25	NA	Ikke påvist	1580	1	18	4457	RESISTANT	NOT_FOUND	RESISTANT	RESISTANT	INTERMEDIATE	RESISTANT	NOT_FOUND	INTERMEDIATE	folP_R228SgyrA_D95AgyrA_S91FparC_S87RpenA_G545SpenA_I312MpenA_V316TrpsJ_V57M	mtrD_mosaic_2mtrR_promoter_mosaic_2	271
22GB000324.fasta	22GB000324	SAMEA117567300	PRJEB83897	25	M	Vestland	West	Sekret	Urethra	2022-04-01	1	0,016	0,004	2	0,125	8	0,25	NA	Ikke påvist	1580	1	18	4457	RESISTANT	NOT_FOUND	RESISTANT	RESISTANT	INTERMEDIATE	RESISTANT	NOT_FOUND	INTERMEDIATE	folP_R228SgyrA_D95AgyrA_S91FparC_S87RpenA_G545SpenA_I312MpenA_V316TrpsJ_V57M	mtrD_mosaic_2mtrR_promoter_mosaic_2	271
22GB000325.fasta	22GB000325	SAMEA117567301	PRJEB83897	28	M	Oslo	East	Sekret	Urethra	2022-03-31	2	0,032	0,004	2	0,25	16	0,25	NA	Ikke påvist	1580	1	18	4457	RESISTANT	NOT_FOUND	RESISTANT	RESISTANT	INTERMEDIATE	RESISTANT	NOT_FOUND	INTERMEDIATE	folP_R228SgyrA_D95AgyrA_S91FparC_S87RpenA_G545SpenA_I312MpenA_V316TrpsJ_V57M	mtrD_mosaic_2mtrR_promoter_mosaic_2	271
22GB000349.fasta	22GB000349	SAMEA117567307	PRJEB83897	25	M	NA	NA	Sekret	Urethra	2022-04-04	2	0,032	0,004	2	0,25	16	0,25	8	Negativ	1580	1	18	4457	RESISTANT	NOT_FOUND	RESISTANT	RESISTANT	INTERMEDIATE	RESISTANT	NOT_FOUND	INTERMEDIATE	folP_R228SgyrA_D95AgyrA_S91FparC_S87RpenA_G545SpenA_I312MpenA_V316TrpsJ_V57M	mtrD_mosaic_2mtrR_promoter_mosaic_2	271
22GB000367.fasta	22GB000367	SAMEA117567310	PRJEB83897	25	M	Oslo	East	Sekret	Anus	2022-04-05	0,5	0,032	0,004	2	0,125	8	0,25	NA	Negativ	1580	1	18	4457	RESISTANT	NOT_FOUND	RESISTANT	RESISTANT	INTERMEDIATE	RESISTANT	NOT_FOUND	INTERMEDIATE	folP_R228SgyrA_D95AgyrA_S91FparC_S87RpenA_G545SpenA_I312MpenA_V316TrpsJ_V57M	mtrD_mosaic_2mtrR_promoter_mosaic_2	271
22GB000370.fasta	22GB000370	SAMEA117567313	PRJEB83897	26	M	Oslo	East	Sekret	Anus	2022-04-05	1	0,016	0,016	2	0,25	16	0,25	NA	Negativ	1580	1	18	4457	RESISTANT	NOT_FOUND	RESISTANT	RESISTANT	INTERMEDIATE	RESISTANT	NOT_FOUND	INTERMEDIATE	folP_R228SgyrA_D95AgyrA_S91FparC_S87RpenA_G545SpenA_I312MpenA_V316TrpsJ_V57M	mtrD_mosaic_2mtrR_promoter_mosaic_2	271
22GB000371.fasta	22GB000371	SAMEA117567314	PRJEB83897	21	M	Vestfold og Telemark	East	Sekret	Anus	2022-04-08	1	0,032	0,004	2	0,125	8	0,25	2	Negativ	1580	1	18	4457	RESISTANT	NOT_FOUND	RESISTANT	RESISTANT	INTERMEDIATE	RESISTANT	NOT_FOUND	INTERMEDIATE	folP_R228SgyrA_D95AgyrA_S91FparC_S87RpenA_G545SpenA_I312MpenA_V316TrpsJ_V57M	mtrD_mosaic_2mtrR_promoter_mosaic_2	271
22GB000394.fasta	22GB000394	SAMEA117567323	PRJEB83897	23	M	Oslo	East	Sekret	Anus	2022-04-12	2	0,016	0,008	2	0,125	8	0,25	8	Negativ	1580	1	18	4457	RESISTANT	NOT_FOUND	RESISTANT	RESISTANT	INTERMEDIATE	RESISTANT	NOT_FOUND	INTERMEDIATE	folP_R228SgyrA_D95AgyrA_S91FparC_S87RpenA_G545SpenA_I312MpenA_V316TrpsJ_V57M	mtrD_mosaic_2mtrR_promoter_mosaic_2	271
22GB000496.fasta	22GB000496	SAMEA117567350	PRJEB83897	18	M	Vestfold og Telemark	East	Puss	Urethra	2022-05-04	2	0,064	0,016	4	0,25	8	1	4	Negativ	1580	1	18	4457	RESISTANT	NOT_FOUND	RESISTANT	RESISTANT	INTERMEDIATE	RESISTANT	NOT_FOUND	INTERMEDIATE	folP_R228SgyrA_D95AgyrA_S91FparC_S87RpenA_G545SpenA_I312MpenA_V316TrpsJ_V57M	mtrD_mosaic_2mtrR_promoter_mosaic_2	271
22GB000506.fasta	22GB000506	SAMEA117567354	PRJEB83897	36	M	Oslo	East	Sekret	Urethra	2022-05-09	0,5	0,016	0,008	2	0,25	16	0,125	8	Negativ	1580	1	18	4457	RESISTANT	NOT_FOUND	RESISTANT	RESISTANT	INTERMEDIATE	RESISTANT	NOT_FOUND	INTERMEDIATE	folP_R228SgyrA_D95AgyrA_S91FparC_S87RpenA_G545SpenA_I312MpenA_V316TrpsJ_V57M	mtrD_mosaic_2mtrR_promoter_mosaic_2	271
22GB000619.fasta	22GB000619	SAMEA117567380	PRJEB83897	38	M	Oslo	East	Sekret	Anus	2022-05-18	2	0,032	0,008	2	0,25	16	4	8	Negativ	1580	1	18	4457	RESISTANT	NOT_FOUND	RESISTANT	RESISTANT	INTERMEDIATE	RESISTANT	NOT_FOUND	INTERMEDIATE	folP_R228SgyrA_D95AgyrA_S91FparC_S87RpenA_G545SpenA_I312MpenA_V316TrpsJ_V57M	mtrD_mosaic_2mtrR_promoter_mosaic_2	271
22GB000621.fasta	22GB000621	SAMEA117567382	PRJEB83897	25	M	Oslo	East	Sekret	Anus	2022-05-20	2	0,032	0,008	16	0,25	16	0,25	8	Negativ	1580	1	18	4457	RESISTANT	NOT_FOUND	RESISTANT	RESISTANT	INTERMEDIATE	RESISTANT	NOT_FOUND	INTERMEDIATE	folP_R228SgyrA_D95AgyrA_S91FparC_S87RpenA_G545SpenA_I312MpenA_V316TrpsJ_V57M	mtrD_mosaic_2mtrR_promoter_mosaic_2	271
22GB000626.fasta	22GB000626	SAMEA117567386	PRJEB83897	28	M	Oslo	East	Sekret	Urethra	2022-05-23	0,5	0,032	0,008	2	0,25	8	0,25	4	Negativ	1580	1	18	4457	RESISTANT	NOT_FOUND	RESISTANT	RESISTANT	INTERMEDIATE	RESISTANT	NOT_FOUND	INTERMEDIATE	folP_R228SgyrA_D95AgyrA_S91FparC_S87RpenA_G545SpenA_I312MpenA_V316TrpsJ_V57M	mtrD_mosaic_2mtrR_promoter_mosaic_2	271
22GB000636.fasta	22GB000636	SAMEA117567393	PRJEB83897	30	M	Oslo	East	Sekret	Anus	2022-05-25	2	0,064	0,016	4	0,25	16	1	8	Negativ	1580	1	18	4457	RESISTANT	NOT_FOUND	RESISTANT	RESISTANT	INTERMEDIATE	RESISTANT	NOT_FOUND	INTERMEDIATE	folP_R228SgyrA_D95AgyrA_S91FparC_S87RpenA_G545SpenA_I312MpenA_V316TrpsJ_V57M	mtrD_mosaic_2mtrR_promoter_mosaic_2	271
22GB000690.fasta	22GB000690	SAMEA117567412	PRJEB83897	29	M	Oslo	East	Sekret	Anus	2022-06-01	0,25	0,016	0,008	1	0,25	8	0,125	NA	Negativ	1580	1	18	4457	RESISTANT	NOT_FOUND	RESISTANT	RESISTANT	INTERMEDIATE	RESISTANT	NOT_FOUND	INTERMEDIATE	folP_R228SgyrA_D95AgyrA_S91FparC_S87RpenA_G545SpenA_I312MpenA_V316TrpsJ_V57M	mtrD_mosaic_2mtrR_promoter_mosaic_2	271
22GB000725.fasta	22GB000725	SAMEA117567433	PRJEB83897	23	M	Oslo	East	Sekret	Hals	2022-06-15	1	0,064	0,016	4	0,125	8	1	4	Negativ	1580	1	18	4457	RESISTANT	NOT_FOUND	RESISTANT	RESISTANT	INTERMEDIATE	RESISTANT	NOT_FOUND	INTERMEDIATE	folP_R228SgyrA_D95AgyrA_S91FparC_S87RpenA_G545SpenA_I312MpenA_V316TrpsJ_V57M	mtrD_mosaic_2mtrR_promoter_mosaic_2	271
22GB000766.fasta	22GB000766	SAMEA117567450	PRJEB83897	32	M	Oslo	East	Sekret	Anus	2022-06-20	2	0,032	0,016	2	0,5	16	0,25	8	Negativ	1580	1	18	4457	RESISTANT	NOT_FOUND	RESISTANT	RESISTANT	INTERMEDIATE	RESISTANT	NOT_FOUND	INTERMEDIATE	folP_R228SgyrA_D95AgyrA_S91FparC_S87RpenA_G545SpenA_I312MpenA_V316TrpsJ_V57M	mtrD_mosaic_2mtrR_promoter_mosaic_2	271
22GB000780.fasta	22GB000780	SAMEA117567457	PRJEB83897	25	M	Troms og Finnmark	North	Sekret	Hals	2022-06-28	2	0,064	0,016	2	0,25	16	0,25	8	Negativ	1580	1	18	4457	RESISTANT	NOT_FOUND	RESISTANT	RESISTANT	INTERMEDIATE	RESISTANT	NOT_FOUND	INTERMEDIATE	folP_R228SgyrA_D95AgyrA_S91FparC_S87RpenA_G545SpenA_I312MpenA_V316TrpsJ_V57M	mtrD_mosaic_2mtrR_promoter_mosaic_2	271
22GB000826.fasta	22GB000826	SAMEA117567476	PRJEB83897	22	M	Oslo	East	Sekret	Anus	2022-07-06	2	0,064	0,016	4	0,25	16	1	8	Negativ	1580	1	18	4457	RESISTANT	NOT_FOUND	RESISTANT	RESISTANT	INTERMEDIATE	RESISTANT	NOT_FOUND	INTERMEDIATE	folP_R228SgyrA_D95AgyrA_S91FparC_S87RpenA_G545SpenA_I312MpenA_V316TrpsJ_V57M	mtrD_mosaic_2mtrR_promoter_mosaic_2	271

671389.fasta	671389	SAMEA6534562	PRJEB32435	22	F	Vestland	West	Sekret	Cervix uter	2019-08-30	0,25	0,032	0,016	0,004	0,5	16	0,5	4	Ikke påvist	[illegible]
22GB000755.fasta	22GB000755	SAMEA117567446	PRJEB83897	21	M	Viken	East	Sekret	Urethra	2022-06-22	0,5	0,032	0,016	0,008	4	16	0,5	4	Negativ	[illegible]
22GB001414.fasta	22GB001414	SAMEA117567809	PRJEB83897	22	F	Oslo	East	Sekret	Cervix uter	2022-10-24	0,5	0,016	0,008	0,008	2	32	0,125	8	Negativ	[illegible]
22GB001464.fasta	22GB001464	SAMEA117567839	PRJEB83897	23	F	Rogaland	West	Sekret	Anus	2022-11-04	0,5	0,016	0,016	0,008	2	16	0,5	4	Negativ	[illegible]
22GB001774.fasta	22GB001774	SAMEA117567968	PRJEB83897	23	F	Nordland	North	Sekret	Cervix uter	2022-12-16	0,5	0,016	0,004	0,008	2	32	0,125	4	Negativ	[illegible]
23GB000276.fasta	23GB000276	SAMEA117568078	PRJEB83897	29	F	Oslo	East	Sekret	Cervix uter	2023-01-23	0,5	0,032	0,016	0,008	2	16	0,5	NA	Negativ	[illegible]
22GB001176.fasta	22GB001176	SAMEA117567683	PRJEB83897	19	F	Viken	East	Sekret	Cervix uter	2022-09-20	0,5	0,064	0,032	0,008	1	16	0,25	8	Negativ	[illegible]
22GB000095.fasta	22GB000095	SAMEA117567216	PRJEB83897	57	M	Oslo	East	Sekret	Anus	2022-01-24	0,25	0,016	0,032	16	0,5	16	1	NA	Ikke påvist	[illegible]
20GB000384.fasta	20GB000384	SAMEA117566629	PRJEB83897	23	F	Trøndelag	North	Sekret	Urethra	2020-01-30	256	0,064	0,032	0,008	0,5	8	0,5	8	Ikke påvist	[illegible]
20GB000628.fasta	20GB000628	SAMEA117566682	PRJEB83897	21	F	Møre og Romsdal	West	Sekret	Cervix uter	2020-02-26	256	0,032	0,016	0,008	1	16	1	4	Ikke påvist	[illegible]
20GB000641.fasta	20GB000641	SAMEA117566690	PRJEB83897	21	F	Møre og Romsdal	West	Sekret	Cervix uter	2020-02-25	256	0,032	0,032	0,008	1	16	0,5	NA	Ikke påvist	[illegible]
20GB000647.fasta	20GB000647	SAMEA117566691	PRJEB83897	21	F	Trøndelag	North	Sekret	Cervix uter	2020-02-28	256	0,064	0,032	0,008	0,5	16	0,5	NA	Ikke påvist	[illegible]
676685.fasta	676685	SAMEA6534679	PRJEB32435	18	F	Vestland	West	Sekret	Cervix uter	2019-11-08	256	0,064	0,032	0,016	1	16	0,5	8	Ikke påvist	[illegible]
665357.fasta	665357	SAMEA6534289	PRJEB32435	29	M	Oslo	East	Sekret	Anus	2019-06-04	32	0,016	0,016	8	2	8	0,5	4	Ikke påvist	[illegible]
23GB001000.fasta	23GB001000	SAMEA117568410	PRJEB83897	23	M	Nordland	North	Sekret	Urethra	2023-04-24	8	0,016	0,016	8	1	16	0,5	NA	Negativ	[illegible]
22GB001300.fasta	22GB001300	SAMEA117567743	PRJEB83897	27	M	Oslo	East	Sekret	Urethra	2022-10-11	8	0,016	0,016	4	1	8	0,25	8	Negativ	[illegible]
22GB001439.fasta	22GB001439	SAMEA117567829	PRJEB83897	23	F	Oslo	East	Sekret	Vagina	2022-10-26	16	0,032	0,032	4	1	8	0,5	4	Negativ	[illegible]
22GB001605.fasta	22GB001605	SAMEA117567908	PRJEB83897	23	F	Vestland	West	Sekret	Cervix uter	2022-11-22	8	0,016	0,016	4	2	8	0,5	4	Negativ	[illegible]
664989.fasta	664989	SAMEA6534284	PRJEB32435	23	F	Nordland	North	Sekret	Cervix uter	2019-05-23	8	0,016	0,016	2	1	16	0,5	8	Ikke påvist	[illegible]
22GB001615.fasta	22GB001615	SAMEA117567911	PRJEB83897	21	F	Oslo	East	Sekret	Cervix uter	2022-11-29	4	0,064	0,032	0,008	0,5	16	0,25	4	Negativ	[illegible]
600792.fasta	600792	SAMEA5609079	PRJEB32435	21	F	Oslo	East	Sekret	Cervix uter	2017-10-04	1	0,016	0,002	0,002	0,25	8	0,5	NA	NA	[illegible]
542783.fasta	542783	SAMEA5608330	PRJEB32435	22	M	Oslo	East	Sekret	Urethra	2016-08-11	8	0,016	0,008	0,004	0,5	16	1	NA	NA	[illegible]
553651.fasta	553651	SAMEA5608422	PRJEB32435	28	M	Oslo	East	Sekret	Urethra	2016-07-05	8	0,016	0,008	0,004	0,5	16	0,5	NA	NA	[illegible]
554680.fasta	554680	SAMEA5608469	PRJEB32435	23	M	Vestfold og Telemark	East	Sekret	Urethra	2016-07-06	16	0,032	0,016	0,004	1	16	1	NA	NA	[illegible]
554689.fasta	554689	SAMEA5608471	PRJEB32435	21	F	Viken	East	Sekret	Urethra	2016-07-11	16	0,016	0,008	0,004	1	16	1	NA	NA	[illegible]
555523.fasta	555523	SAMEA5608496	PRJEB32435	31	M	Oslo	East	Sekret	Urethra	2016-09-29	8	0,016	0,008	0,004	1	16	1	NA	NA	[illegible]
624923.fasta	624923	SAMEA5609201	PRJEB32435	19	M	NA	NA	SEKRET	ANUS	2018-04-03	4	0,016	0,004	0,002	0,125	16	0,25	-	NA	[illegible]
646395.fasta	646395	SAMEA6533968	PRJEB32435	29	M	NA	NA	SEKRET	HALS	2018-11-28	NA	NA	NA	NA	NA	NA	NA	NA	NA	[illegible]
650380.fasta	650380	SAMEA6534016	PRJEB32435	34	M	Oslo	East	Sekret	Hals	2019-01-11	4	0,016	0,002	0,002	0,125	8	0,25	2	Ikke påvist	[illegible]
550339.fasta	550339	SAMEA5608353	PRJEB32435	67	M	Trøndelag	North	Sekret	Urethra	2016-10-21	0,5	0,064	0,008	8	32	16	64	NA	NA	[illegible]
576043.fasta	576043	SAMEA5608796	PRJEB32435	28	M	Vestland	West	Sekret	Urethra	2017-04-28	8	0,016	0,004	8	32	16	64	NA	NA	[illegible]
680264.fasta	680264	SAMEA117568463	PRJEB83897	40	M	Vestfold og Telemark	East	Sekret	Urethra	2019-12-10	4	0,016	0,004	1	2	8	0,5	8	Påvist	[illegible]
22GB001332.fasta	22GB001332	SAMEA117567766	PRJEB83897	39	F	Viken	East	Sekret	Vagina	2022-10-14	256	0,016	0,002	0,064	0,125	8	0,125	4	Negativ	[illegible]
634023.fasta	634023	SAMEA6533678	PRJEB32435	23	M	NA	NA	SEKRET	URETRA	2018-07-16	NA	NA	NA	NA	NA	NA	NA	NA	NA	[illegible]
592489.fasta	592489	SAMEA5608910	PRJEB32435	35	M	NA	NA	Sekret	Urethra	2017-07-12	16	0,016	0,002	4	0,5	8	1	NA	NA	[illegible]
629684.fasta	629684	SAMEA5609222	PRJEB32435	42	M	NA	NA	SEKRET	ANUS	2018-05-23	NA	NA	NA	NA	NA	NA	NA	NA	NA	[illegible]
630152.fasta	630152	SAMEA6963053	PRJEB32435	32	M	NA	NA	SEKRET	URETRA	2018-05-28	4	0,016	0,004	0,004	0,25	16	1	-	NA	[illegible]
554395.fasta	554395	SAMEA5608445	PRJEB32435	32	M	Troms og Finnmark	North	Sekret	Urethra	2016-11-21	32	0,125	0,064	32	1	16	4	NA	NA	[illegible]
553571.fasta	553571	SAMEA5608406	PRJEB32435	24	M	Oslo	East	Sekret	Anus	2016-05-18	32	0,064	0,032	32	1	16	2	NA	NA	[illegible]
553760.fasta	553760	SAMEA5608438	PRJEB32435	27	M	Oslo	East	Sekret	Urethra	2016-05-04	32	0,125	0,032	32	1	16	2	NA	NA	[illegible]
553823.fasta	553823	SAMEA5608440	PRJEB32435	23	M	Oslo	East	Sekret	Anus	2016-05-04	0,25	0,25	0,125	32	2	16	4	NA	NA	[illegible]
555561.fasta	555561	SAMEA5608499	PRJEB32435	38	M	Oslo	East	Sekret	Urethra	2016-10-04	16	0,125	0,064	32	2	16	1	NA	NA	[illegible]
563764.fasta	563764	SAMEA6533366	PRJEB32435	26	M	Oslo	East	Sekret	Anus	2016-12-20	16	0,125	0,064	32	1	16	2	NA	NA	[illegible]
571505.fasta	571505	SAMEA5608720	PRJEB32435	26	M	Oslo	East	Sekret	Anus	2017-03-16	32	0,064	0,032	32	0,5	16	2	NA	NA	[illegible]
594675.fasta	594675	SAMEA5608977	PRJEB32435	46	M	Oslo	East	Sekret	Urethra	2017-08-01	0,5	0,016	0,002	0,002	0,064	16	0,5	NA	NA	[illegible]
23GB000206.fasta	23GB000206	SAMEA117568041	PRJEB83897	34	M	Viken	East	Sekret	Hals	2023-01-12	0,5	0,016	0,002	0,002	0,032	8	0,125	NA	Negativ	[illegible]
568642.fasta	568642	SAMEA5608696	PRJEB32435	28	M	Oslo	East	Sekret	Anus	2017-02-27	0,5	0,016	0,004	0,004	0,125	16	0,5	NA	NA	[illegible]
569261.fasta	569261	SAMEA5608705	PRJEB32435	35	M	Vestfold og Telemark	East	Sekret	Ureter	2017-03-05	1	0,016	0,002	0,004	0,25	16	1	NA	NA	[illegible]
571518.fasta	571518	SAMEA5608723	PRJEB32435	66	M	Oslo	East	Sekret	Hals	2017-03-13	0,5	0,016	0,002	0,004	0,064	8	0,5	NA	NA	[illegible]
571802.fasta	571802	SAMEA5608739	PRJEB32435	21	M	Oslo	East	Sekret	Urethra	2017-03-21	0,5	0,016	0,004	0,004	0,125	16	0,5	NA	NA	[illegible]
598034.fasta	598034	SAMEA5609038	PRJEB32435	29	M	Oslo	East	Sekret	Urethra	2017-09-04	0,5	0,016	0,002	0,004	0,125	16	1	NA	NA	[illegible]
601031.fasta	601031	SAMEA5609081	PRJEB32435	19	F	Møre og Romsdal	West	Ikke angitt	Cervix uter	2017-09-30	1	0,016	0,004	8	0,125	16	32	NA	NA	[illegible]
23GB000200.fasta	23GB000200	SAMEA117568037	PRJEB83897	42	M	Oslo	East	Sekret	Urethra	2023-01-09	1	0,016	0,002	2	0,064	32	0,25	NA	Negativ	[illegible]
23GB000920.fasta	23GB000920	SAMEA117568381	PRJEB83897	22	M	Viken	East	Sekret	Hals	2023-04-17	0,5	0,016	0,016	4	0,125	16	0,5	NA	Negativ	[illegible]
20GB000675.fasta	20GB000675	SAMEA117566699	PRJEB83897	42	M	Viken	East	Sekret	Urethra	2020-03-06	0,5	0,016	0,004	0,004	0,125	8	0,5	NA	Ikke påvist	[illegible]
20GB000677.fasta	20GB000677	SAMEA117566701	PRJEB83897	44	M	Oslo	East	Sekret	Anus	2020-03-05	0,5	0,016	0,004	0,004	0,125	8	0,25	NA	Ikke påvist	[illegible]
20GB000785.fasta	20GB000785	SAMEA117566751	PRJEB83897	43	M	Oslo	East	Sekret	Urethra	2020-04-15	0,5	0,016	0,002	0,004	0,25	16	0,25	NA	Ikke påvist	[illegible]
20GB000797.fasta	20GB000797	SAMEA117566754	PRJEB83897	42	M	Oslo	East	Sekret	Urethra	2020-04-21	1	0,016	0,002	0,004	0,25	8	0,25	NA	Ikke påvist	[illegible]
23GB000773.fasta	23GB000773	SAMEA117568319	PRJEB83897	20	M	Viken	East	Sekret	Urethra	2023-03-24	0,25	0,016	0,002	0,004	0,125	16	0,125	NA	Negativ	[illegible]
642285.fasta	642285	SAMEA6533892	PRJEB32435	21	M	NA	NA	SEKRET	URETRA	2018-10-17	0,125	0,016	0,016	0,002	NA	NA	NA	NA	NA	[illegible]
644083.fasta	644083	SAMEA6533935	PRJEB32435	18	F	NA	NA	SEKRET	CERVIXUT	2018-11-06	NA	NA	NA	NA	NA	NA	NA	NA	NA	[illegible]
644434.fasta	644434	SAMEA6533937	PRJEB32435	24	M	NA	NA	SEKRET	HALS	2018-11-12	NA	NA	NA	NA	NA	NA	NA	NA	NA	[illegible]
645910.fasta	645910	SAMEA6533956	PRJEB32435	16	F	NA	NA	SEKRET	VAGINA	2018-11-23	NA	NA	NA	NA	NA	NA	NA	NA	NA	[illegible]
646926.fasta	646926	SAMEA6533980	PRJEB32435	22	F	NA	NA	SEKRET	CERVIXUT	2018-11-30	NA	NA	NA	NA	NA	NA	NA	NA	NA	[illegible]
678568.fasta	678568	SAMEA6534723	PRJEB32435	41	M	Oslo	East	Sekret	Hals	2019-11-28	0,125	0,064	0,008	0,004	0,25	8	0,25	2	Ikke påvist	[illegible]
576166.fasta	576166	SAMEA117568446	PRJEB83897	28	M	Oslo	East	Sekret	Rektum	2017-05-02	0,5	0,016	0,002	0,004	0,125	16	0,5	NA	NA	[illegible]
606596.fasta	606596	SAMEA6533392	PRJEB32435	40	M	Agder	South	Sekret	Anus	2017-11-21	0,5	0,016	0,004	0,004	0,125	16	0,5	NA	NA	[illegible]
637467.fasta	637467	SAMEA6533779	PRJEB32435	16	F	NA	NA	SEKRET	VAGINA	2018-08-27	NA	NA	NA	NA	NA	NA	NA	NA	NA	[illegible]
675040.fasta	675040	SAMEA6534649	PRJEB32435	34	M	Oslo	East	Sekret	Urethra	2019-10-18	0,5	0,016	0,002	0,008	0,25	16	0,5	4	Ikke påvist	[illegible]
20GB001109.fasta	20GB001109	SAMEA117566845	PRJEB83897	19	M	Oslo	East	Sekret	Anus	2020-07-22	0,5	0,016	0,002	0,004	0,064	8	0,25	NA	Ikke påvist	[illegible]
20GB001123.fasta	20GB001123	SAMEA117566851	PRJEB83897	28	M	Vestfold og Telemark	East	Sekret	Urethra	2020-07-30	0,5	0,016	0,016	0,004	0,125	8	0,5	NA	Ikke påvist	[illegible]
20GB001127.fasta	20GB001127	SAMEA117566852	PRJEB83897	27	M	Oslo	East	Sekret	Anus	2020-07-30	0,5	0,016	0,016	0,004	0,125	8	0,5	NA	Ikke påvist	[illegible]
22GB000593.fasta	22GB000593	SAMEA117567372	PRJEB83897	30	M	Troms og Finnmark	North	Sekret	Anus	2022-05-16	1	0,016	0,004	0,008	0,25	16	0,5	NA	Negativ	[illegible]
673788.fasta	673788	SAMEA6534626	PRJEB32435	21	M	Vestland	West	Sekret	Hals	2019-10-02	0,5	0,016	0,016	0,016	0,5	16	1	8	Ikke påvist	[illegible]
674309.fasta	674309	SAMEA6534635	PRJEB32435	22	M	Vestland	West	Sekret	Anus	2019-10-07	0,5	0,016	0,016	0,016	0,25	16	1	8	Ikke påvist	[illegible]
20GB000705.fasta	20GB000705	SAMEA117566716	PRJEB83897	29	M	Rogaland	West	Sekret	Urethra	2020-03-12	1	0,016	0,016	0,016	0,25	16	2	NA	Ikke påvist	[illegible]
553653.fasta	553653	SAMEA5608423	PRJEB32435	41	M	Viken	East	Sekret	Urethra	2016-07-06	0,5	0,016	0,008	0,008	0,25	8	0,5	NA	NA	[illegible]
575796.fasta	575796	SAMEA5608793	PRJEB32435	25	F	Nordland	North	Sekret	Cervix uter	2017-04-27	0,5	0,016	0,004	0,008	0,125	16	0,5	NA	NA	[illegible]
22GB001408.fasta	22GB001408	SAMEA117567804	PRJEB83897	46	F	Rogaland	West	Sekret	Vagina	2022-10-25	0,5	0,016	0,016	0,008	0,125	16	1	8	Negativ	[illegible]
22GB001409.fasta	22GB001409	SAMEA117567805	PRJEB83897	47	M	Rogaland	West	Sekret	Urethra	2022-10-25	0,5	0,016	0,016	0,008	0,125	8	1	8	Negativ	[illegible]
20GB000172.fasta	20GB000172	SAMEA117566569	PRJEB83897	54	M	Oslo	East	Sekret	Hals	2019-12-18	0,25	0,016	0,002	0,008	0,064	8	0,25	4	Ikke påvist	[illegible]
20GB000528.fasta	20GB000528	SAMEA117566653	PRJEB83897	19	M	Vestland	West	Sekret	Urethra	2020-02-13	1	0,016	0,016	0,004	0,25	8	1	8	Ikke påvist	[illegible]
20GB000545.fasta	20GB000545	SAMEA117566658	PRJEB83897	60	M	Rogaland	West	Sekret	Hals	2020-02-12	0,5	0,016	0,016	0,004	0,125	8	0,5	NA	Ikke påvist	[illegible]
20GB000633.fasta	20GB000633	SAMEA117566684	PRJEB83897	22	M	Oslo	East	Sekret	Anus	2020-02-26	0,5	0,016	0,004	0,004	0,125	16	0,25	NA	Ikke påvist	[illegible]
20GB000683.fasta	20GB000683	SAMEA117566707	PRJEB83897	29	M	Viken	East	Sekret	Urethra	2020-03-03	0,5	0,016	0,002	0,008	0,125	16	0,25	NA	Ikke påvist	[illegible]
20GB000730.fasta	20GB000730	SAMEA117566729	PRJEB83897	33	F	Oslo	East	Sekret	Vagina	2020-03-16	0,125	0,016	0,004	0,004	0,125	8	0,125	NA	Ikke påvist	[illegible]
20GB000758.fasta	20GB000758	SAMEA117566740	PRJEB83897	57	M	Innlandet	East	Sekret	Anus	2020-03-25	0,032	0,016	0,002	0,002	0,064	4	0,25	NA	Ikke påvist	[illegible]
20GB000806.fasta	20GB000806	SAMEA117566758	PRJEB83897	27	M	Oslo	East	Sekret	Hals	2020-04-22	0,25	0,016	0,002	0,004	0,125	8	0,125	NA	Ikke påvist	[illegible]
20GB000808.fasta	20GB000808	SAMEA117566760	PRJEB83897	38	M	Oslo	East	Sekret	Urethra	2020-04-23	0,125	0,016	0,002	0,004	0,125	16	0,125	NA	Ikke påvist	[illegible]
20GB000819.fasta	20GB000819	SAMEA117566762	PRJEB83897	35	M	Oslo	East	Sekret	Hals	2020-04-29	0,5	0,016	0,004	0,004	0,125	8	0,25	NA	Ikke påvist	[illegible]
20GB000950.fasta	20GB000950	SAMEA117566768	PRJEB83897	37	M	Oslo	East	Sekret	Hals	2020-05-15	0,5	0,016	0,016	0,004	0,25	16	1	NA	Ikke påvist	[illegible]
20GB000953.fasta	20GB000953	SAMEA117566771	PRJEB83897	29	M	Oslo	East	Sekret	Hals	2020-05-18	0,5	0,016	0,002	0,004	0,125	8	0,25	NA	Ikke påvist	[illegible]
20GB000959.fasta	20GB000959	SAMEA117566774	PRJEB83897	32	M	Vestland	West	Sekret	Hals	2020-05-22	0,5	0,016	0,016	0,004	0,064	8	1	NA	Ikke påvist	[illegible]
20GB001014.fasta	20GB001014	SAMEA117566795	PRJEB83897	22	M	Viken	East	Sekret	Anus	2020-06-15	0,5	0,016	0,002	0,004	0,125	8	0,25	NA	Ikke påvist	[illegible]
20GB001033.fasta	20GB001033	SAMEA117566804	PRJEB83897	28	M	Oslo	East	Sekret	Urethra	2020-06-22	0,25	0,016	0,002	0,004	0,125	8	0,125	NA	Ikke påvist	[illegible]
20GB001034.fasta	20GB001034	SAMEA117566805	PRJEB83897	35	M	Oslo	East	Sekret	Anus	2020-06-22	0,25	0,016	0,002	0,004	0,125	16	0,125	NA	Ikke påvist	[illegible]
20GB001161.fasta	20GB001161	SAMEA117566867	PRJEB83897	37	M	Oslo	East	Sekret	Ureter	2020-08-18	0,25	0,016	0,002	0,004	1	8	0,25	NA	Ikke påvist	[illegible]
20GB001187.fasta	20GB001187	SAMEA117566878	PRJEB83897	21	M	Oslo	East	Sekret	Hals	2020-08-24	0,25	0,016	0,002	0,004	0,125	8	0,125	NA	Ikke påvist	[illegible]
20GB001307.fasta	20GB001307	SAMEA117566940	PRJEB83897	28	M	Vestland	West	Sekret	Hals	2020-10-21	1	0,016	0,016	0,004	0,25	16	1	NA	Ikke påvist	[illegible]
20GB001323.fasta	20GB001323	SAMEA117566946	PRJEB83897	20	F	Viken	East	Sekret	Vagina	2020-10-26	0,5	0,016	0,016	0,004	0,25	16	1	NA	Ikke påvist	[illegible]
20GB001365.fasta	20GB001365	SAMEA117566965	PRJEB83897	33	M	Viken	East	Sekret	Hals	2020-11-16	0,5	0,016	0,016	0,004	0,125	16	1	NA	Ikke påvist	[illegible]
20GB001391.fasta	20GB001391	SAMEA117566978	PRJEB83897	44	M	Oslo	East	Sekret	Anus	2020-11-23	0,5	0,016	0,002	0,004	0,125	8	0,25	NA	Ikke påvist	[illegible]
20GB001393.fasta	20GB001393	SAMEA117566980	PRJEB83897	31	M	NA	NA	Sekret	Anus	2020-11-26	0,5	0,016	0,002	0,002	0,125	4	0,25	NA	Ikke påvist	[illegible]
20GB001419.fasta	20GB001419	SAMEA117566986	PRJEB83897	20	F	Møre og Romsdal	West	Sekret	Ureter	2020-12-17	0,5	0,016	0,016	0,008	0,25	0,064	0,064	NA	Ikke påvist	[illegible]
21GB000121.fasta	21GB000121	SAMEA117567005	PRJEB83897	39	M	NA	NA	Sekret	Urethra	2021-03-04	0,25	0,016	0,002	0,002	0,064	8	0,125	NA	Ikke påvist	[illegible]
21GB000191.fasta	21GB000191	SAMEA117567024	PRJEB83897	22	F	Oslo	East	Sekret	Cervix uter	2021-04-30	0,5	0,016	0,016	0,004	0,25	16	1	NA	Ikke påvist	[illegible]
21GB000331.fasta	21GB000331	SAMEA117567039	PRJEB83897	20	F	Møre og Romsdal	West	Sekret	Cervix uter	2021-06-17	0,064	0,016	0,016	0,004	0,125	16	0,5	NA	Ikke påvist	[illegible]
21GB000340.fasta	21GB000340	SAMEA117567040	PRJEB83897	25	M	Oslo	East	Sekret	Urethra	2021-06-24	0,064	0,016	0,016	0,004	0,125	16	1	NA	Ikke påvist	[illegible]
21GB000349.fasta	21GB000349	SAMEA117567042	PRJEB83897	19	M	Oslo	East	Sekret	Hals	2021-06-28	0,064	0,016	0,002	0,002	0,125	8	0,25	NA	Ikke påvist	[illegible]
21GB000358.fasta	21GB000358	SAMEA117567045	PRJEB83897	30	M	Oslo	East	Sekret	Urethra	2021-07-09	0,064	0,016	0,002	0,002	0,125	8	0,25	NA	Ikke påvist	[illegible]
21GB000499.fasta	21GB000499	SAMEA117567073	PRJEB83897	28	M	Agder	South	Sekret	Anus	2021-08-26	0,5	0,016	0,016	0,004	0,125	16	1	NA	Ikke påvist	[illegible]
21GB000515.fasta	21GB000515	SAMEA117567077	PRJEB83897	31	M	Oslo	East	Sekret	Anus	2021-09-13	0,5	0,016	0,002	0,004	0,125	8	0,25	NA	Ikke påvist	[illegible]
21GB000571.fasta	21GB000571	SAMEA117567093	PRJEB83897	27	F	Troms og Finnmark	North	Sekret	Anus	2021-09-30	0,064	0,016	0,016	0,004	0,125	16	1	NA	Ikke påvist	[illegible]
21GB000622.fasta	21GB000622	SAMEA117567109	PRJEB83897	23	F	Troms og Finnmark	North	Sekret	Cervix uter	2021-10-15	0,064	0,016	0,016	0,004	0,125	8	0,5	NA	Ikke påvist	[illegible]
22GB000003.fasta	22GB000003	SAMEA117567186	PRJEB83897	46	M	Oslo	East	Sekret	Hals	2021-12-23	0,25	0,016	0,002	0,004	0,125	8	0,25	NA	Ikke påvist	[illegible]
22GB000046.fasta	22GB000046	SAMEA117567198	PRJEB83897	40	M	Oslo	East	Sekret	Urethra	2022-01-07	0,5	0,016	0,002	0,004	0,25	8	0,25	NA	Ikke påvist	[illegible]
22GB000115.fasta	22GB000115	SAMEA117567226	PRJEB83897	26	M	Oslo	East	Sekret	Urethra	2022-02-02	0,5	0,016	0,002	0,002	0,064	8	0,125	NA	Ikke påvist	[illegible]
22GB000130.fasta	22GB000130	SAMEA117567236	PRJEB83897	19	F	Oslo	East	Sekret	Cervix uter	2022-02-08	1	0,016	0,002	0,004	0,125	8	0,25	NA	Ikke påvist	[illegible]
22GB000155.fasta	22GB000155	SAMEA117567251	PRJEB83897	38	M	Vestland	West	Sekret	Urethra	2022-02-14	1	0,016	0,016	0,004	0,125	8	1	NA	Ikke påvist	[illegible]
22GB000162.fasta	22GB000162	SAMEA117567254	PRJEB83897	22	M	Oslo	East	Sekret	Hals	2022-02-16	0,5	0,016	0,016	0,004	0,125	16	1	NA	Ikke påvist	[illegible]
22GB000372.fasta	22GB000372	SAMEA117567315	PRJEB83897	30	M	Oslo	East	Sekret	Anus	2022-04-08	0,5	0,016	0,002	0,002	0,25	8	0,25	8	Negativ	[illegible]
22GB000635.fasta	22GB000635	SAMEA117567392	PRJEB83897	25	M	Viken	East	Sekret	Ureter	2022-05-24	0,5	0,016	0,016	0,002	0,125	8	0,5	4	Negativ	[illegible]
22GB000641.fasta	22GB000641	SAMEA117567396	PRJEB83897	31	M	Oslo	East	Sekret	Hals	2022-05-25	0,125	0,016	0,016	0,002	0,125	8	0,125	4	Negativ	[illegible]
22GB000645.fasta	22GB000645	SAMEA117567400	PRJEB83897	24	M	Vestland	West	Sekret	Anus	2022-05-27	0,5	0,016	0,016	0,004	0,125	8	0,5	4	Negativ	[illegible]
22GB000689.fasta	22GB000689	SAMEA117567411	PRJEB83897	28	M	Oslo	East	Sekret	Anus	2022-06-03	0,064	0,016	0,002	0,002	0,125	8	0,064	8	Negativ	[illegible]
22GB000698.fasta	22GB000698	SAMEA117567416	PRJEB83897	33	M	Oslo	East	Sekret	Urethra	2022-06-07	0,5	0,016	0,002	0,002	0,25	8	0,125	2	Negativ	[illegible]
22GB000713.fasta	22GB000713	SAMEA117567423	PRJEB83897	21	M	Vestland	West	Urin		2022-06-13	0,5	0,016	0,016	0,004	0,125	16	0,5	4	Negativ	[illegible]
22GB000714.fasta	22GB000714	SAMEA117567424	PRJEB83897	21	M	Vestland	West	Sekret	Urethra	2022-06-15	0,5	0,016	0,016	0,004	0,125	16	1	4	Negativ	[illegible]
22GB000794.fasta	22GB000794	SAMEA117567466	PRJEB83897	33	M	Vestland	West	Sekret	Urethra	2022-06-30	0,5	0,016	0,016	0,002	0,125	8	0,5	4	Negativ	[illegible]
22GB000856.fasta	22GB000856	SAMEA117567491	PRJEB83897	34	M	Viken	East	Sekret	Urethra	2022-07-11	0,5	0,016	0,004	0,004	0,125	8	0,25	8	Negativ	[illegible]
22GB000927.fasta	22GB000927	SAMEA117567532	PRJEB83897	23	M	Viken	East	Sekret	Hals	2022-08-04	0,5	0,016	0,002	0,002	0,125	8	0,25	8	Negativ	[illegible]
22GB000930.fasta	22GB000930	SAMEA117567534	PRJEB83897	29	M	Viken	East	Sekret	Urethra	2022-08-04	0,25	0,016	0,002	0,002	0,125	8	0,125	2	Negativ	[illegible]
22GB000934.fasta	22GB000934	SAMEA117567536	PRJEB83897	31	M	Oslo	East	Sekret	Urethra	2022-08-03	0,5	0,016	0,016	0,004	0,125	8	0,5	4	Negativ	[illegible]
22GB000986.fasta	22GB000986	SAMEA117567571	PRJEB83897	18	F	Vestland	West	Sekret	Cervix uter	2022-08-16	0,5	0,016	0,016	0,004	0,125	8	0,5	4	Negativ	[illegible]
22GB001018.fasta	22GB001018	SAMEA117567594	PRJEB83897	35	M	Oslo	East	Sekret	Urethra	2022-08-22	0,25	0,016	0,004	0,004	0,25	16	0,125	8	Negativ	[illegible]
22GB001023.fasta	22GB001023	SAMEA117567595	PRJEB83897	22	M	Vestland	West	Sekret	Hals	2022-08-22	0,25	0,016	0,016	0,004	0,125	8	0,5	4	Negativ	[illegible]
22GB001034.fasta	22GB001034	SAMEA117567603	PRJEB83897	54	M	Oslo	East	Sekret	Urethra	2022-08-26	0,5	0,016	0,002	0,002	0,125	16	0,125	8	Negativ	[illegible]
22GB001131.fasta	22GB001131	SAMEA117567661	PRJEB83897	38	M	Innlandet	East	Sekret	Anus	2022-09-08	0,5	0,016	0,002	0,002	0,125	16	0,25	8	Negativ	[illegible]
22GB001158.fasta	22GB001158	SAMEA117567677	PRJEB83897	19	F	Rogaland	West	Sekret	Vagina	2022-09-14	0,5	0,016	0,016	0,002	0,25	16	0,5	4	Negativ	[illegible]
22GB001212.fasta	22GB001212	SAMEA117567701	PRJEB83897	19	M	Oslo	East	Sekret	Anus	2022-09-26	1	0,016	0,002	0,002	0,25	32	0,25	8	Negativ	[illegible]
22GB001268.fasta	22GB001268	SAMEA117567729	PRJEB83897	32	M	Vestland	West	Sekret	Urethra	2022-10-06	0,5	0,016	0,016	0,004	0,125	8	0,5	8	Negativ	[illegible]
22GB001315.fasta	22GB001315	SAMEA117567751	PRJEB83897	46	M	Vestland	West	Sekret	Anus	2022-10-11	0,25	0,016	0,016	0,002	0,125	16	0,5	8	Negativ	[illegible]
22GB001322.fasta	22GB001322	SAMEA117567757	PRJEB83897	35	M	Oslo	East	Sekret	Urethra	2022-10-12	0,25	0,016	0,002	0,002	0,064	16	0,125	4	Negativ	[illegible]
22GB001335.fasta	22GB001335	SAMEA117567767	PRJEB83897	19	M	Troms og Finnmark	North	Sekret	Urethra	2022-10-13	0,5	0,016	0,016	0,004	0,25	16	1	8	Negativ	[illegible]
22GB001364.fasta	22GB001364	SAMEA117567777	PRJEB83897	25	M	Oslo	East	Sekret	Urethra	2022-10-20	0,5	0,016	0,016	0,004	0,125	8	0,5	4	Negativ	[illegible]
22GB001399.fasta	22GB001399	SAMEA117567800	PRJEB83897	30	M	Oslo	East	Sekret	Hals	2022-10-24	0,25	0,016	0,002	0,002	0,125	16	0,125	2	Negativ	[illegible]
22GB001422.fasta	22GB001422	SAMEA117567816	PRJEB83897	28	M	Vestland	West	Sekret	Anus	2022-10-27	0,5	0,016	0,004	0,002	0,25	16	0,25	8	Negativ	[illegible]
22GB001459.fasta	22GB001459	SAMEA117567835	PRJEB83897	34	M	Oslo	East	Sekret	Urethra	2022-10-31	2	0,016	0,004	0,004	0,25	32	0,5	NA	Negativ	[illegible]
22GB001480.fasta	22GB001480	SAMEA117567851	PRJEB83897	20	M	Vestland	West	Sekret	Urethra	2022-11-02	0,5	0,016	0,016	0,004	0,25	8	0,5	4	Negativ	[illegible]
22GB001531.fasta	22GB001531	SAMEA117567873	PRJEB83897	47	M	Oslo	East	Sekret	Urethra	2022-11-14	0,5	0,016	0,002	0,004	0,125	16	0,5	8	Negativ	[illegible]
22GB001591.fasta	22GB001591	SAMEA117567900	PRJEB83897	18	F	Vestland	West	Sekret	Cervix uter	2022-11-25	0,5	0,016	0,016	0,004	0,25	8	0,5	4	Negativ	[illegible]
22GB001653.fasta	22GB001653	SAMEA117567929	PRJEB83897	33	M	Oslo	East	Sekret	Urethra	2022-12-05	0,032	0,016	0,016	0,002	0,125	16	0,5	4	Negativ	[illegible]
22GB001687.fasta	22GB001687	SAMEA117567942	PRJEB83897	33	M	Oslo	East	Sekret	Urethra	2022-12-08	0,064	0,016	0,002	0,002	0,064	16	0,25	4	Negativ	[illegible]
22GB001766.fasta	22GB001766	SAMEA117567967	PRJEB83897	47	M	Rogaland	West	Sekret	Hals	2022-12-22	0,25	0,016	0,016	0,004	0,25	16	1	8	Negativ	[illegible]
23GB000082.fasta	23GB000082	SAMEA117567992	PRJEB83897	19	M	Vestland	West	Sekret	Urethra	2022-12-19	0,5	0,016	0,016	0,004	0,25	16	0,5	NA	Negativ	[illegible]
23GB000083.fasta	23GB000083	SAMEA117567993	PRJEB83897	17	F	Vestland	West	Sekret	Cervix uter	2022-12-30	0,5	0,016	0,016	0,004	0,25	16	0,5	NA	Negativ	[illegible]
23GB000108.fasta	23GB000108	SAMEA117568000	PRJEB83897	23	F	Vestland	West	Sekret	Cervix uter	2023-01-06	0,5	0,016	0,016	0,004	0,25	8	0,5	NA	Negativ	[illegible]
23GB000189.fasta	23GB000189	SAMEA117568033	PRJEB83897	18	M	Rogaland	West	Sekret	Urethra	2023-01-05	0,5	0,016	0,016	0,004	0,25	16	1	NA	Negativ	[illegible]
23GB000312.fasta	23GB000312	SAMEA117568098	PRJEB83897	22	M	Vestland	West	Sekret	Anus	2023-01-26	0,5	0,016	0,016	0,004	0,125	16	0,5	NA	Negativ	[illegible]
23GB000334.fasta	23GB000334	SAMEA117568109	PRJEB83897	67	M	Viken	East	Sekret	Hals	2023-01-30	0,5	0,016	0,002	0,002	0,25	32	0,25	NA	Negativ	[illegible]
23GB000401.fasta	23GB000401	SAMEA117568143	PRJEB83897	23	M	Vestland	West	Sekret	Anus	2023-02-10	0,5	0,016	0,016	0,004	0,25	16	0,5	NA	Negativ	[illegible]
23GB000472.fasta	23GB000472	SAMEA117568176	PRJEB83897	46	M	Oslo	East	Sekret	Urethra	2023-02-13	0,125	0,016	0,002	0,002	0,125	16	0,125	NA	Negativ	[illegible]
23GB000526.fasta	23GB000526	SAMEA117568207	PRJEB83897	22	F	Rogaland	West	Sekret	Vagina	2023-02-24	0,5	0,016	0,016	0,004	0,25	16	1	NA	Negativ	[illegible]
23GB000588.fasta	23GB000588	SAMEA117568237	PRJEB83897	21	M	Trøndelag	North	Sekret	Hals	2023-03-03	0,5	0,016	0,016	0,004	0,25	16	1	NA	Negativ	[illegible]
23GB000609.fasta	23GB000609	SAMEA117568243	PRJEB83897	21	F	Vestland	West	Sekret	Cervix uter	2023-03-03	0,25	0,016	0,002	0,002	0,064	16	0,25	NA	Negativ	[illegible]
23GB000645.fasta	23GB000645	SAMEA117568264	PRJEB83897	23	M	Vestland	West	Sekret	Urethra	2023-03-10	0,25	0,016	0,016	0,004	0,125	16	1	NA	Negativ	[illegible]
23GB000646.fasta	23GB000646	SAMEA117568265	PRJEB83897	22	F	Vestland	West	Sekret	Cervix uter	2023-03-10	0,5	0,016	0,016	0,004	0,25	16	1	NA	Negativ	[illegible]
23GB000656.fasta	23GB000656	SAMEA117568274	PRJEB83897	26	F	Oslo	East	Sekret	Cervix uter	2023-03-07	0,5	0,016	0,002	0,004	0,125	32	0,25	NA	Negativ	[illegible]
23GB000756.fasta	23GB000756	SAMEA117568305	PRJEB83897	22	F	Nordland	North	Sekret	Cervix uter	2023-03-23	0,5	0,016	0,016	0,004	0,25	16	1	NA	Negativ	[illegible]
23GB000758.fasta	23GB000758	SAMEA117568307	PRJEB83897	21	M	Vestland	West	Sekret	Urethra	2023-03-20	0,25	0,016	0,016	0,004	0,125	16	1	NA	Negativ	[illegible]
23GB000761.fasta	23GB000761	SAMEA117568310	PRJEB83897	21	M	Vestland	West	Sekret	Hals	2023-03-22	0,5	0,016	0,016	0,004	0,25	16	1	NA	Negativ	[illegible]
23GB000762.fasta	23GB000762	SAMEA117568311	PRJEB83897	27	M	Vestland	West	Sekret	Urethra	2023-03-21	0,5	0,016	0,016	0,004	0,125	16	1	NA	Negativ	[illegible]
23GB000763.fasta	23GB000763	SAMEA117568312	PRJEB83897	18	F	Vestland	West	Sekret	Cervix uter	2023-03-24	0,5	0,016	0,016	0,004	0,125	16	0,5	NA	Negativ	[illegible]
23GB000769.fasta	23GB000769	SAMEA117568315	PRJEB83897	36	M	Oslo	East	Sekret	Urethra	2023-03-24	0,5	0,016	0,002	0,002	0,125	16	0,25	NA	Negativ	[illegible]
23GB000855.fasta	23GB000855	SAMEA117568355	PRJEB83897	19	F	Vestland	West	Sekret	Cervix uter	2023-04-05	0,5	0,016	0,016	0,004	0,25	16	0,5	NA	Negativ	[illegible]
23GB000895.fasta	23GB000895	SAMEA117568368	PRJEB83897	25	F	Vestland	West	Sekret	Cervix uter	2023-04-14	0,5	0,016	0,016	0,004	0,125	16	1	NA	Negativ	[illegible]
23GB000896.fasta	23GB000896	SAMEA117568369	PRJEB83897	23	M	Oslo	East	Sekret	Anus	2023-04-14	0,5	0,016	0,016	0,004	0,25	16	1	NA	Negativ	[illegible]
23GB000954.fasta	23GB000954	SAMEA117568392	PRJEB83897	21	F	Nordland	North	Sekret	Cervix uter	2023-04-20	0,5	0,016	0,016	0,004	0,125	16	1	NA	Negativ	[illegible]
23GB000963.fasta	23GB000963	SAMEA117568400	PRJEB83897	34	M	Rogaland	West	Sekret	Urethra	2023-04-19	0,25	0,016	0,016	0,004	0,125	16	0,5	NA	Negativ	[illegible]
23GB001008.fasta	23GB001008	SAMEA117568414	PRJEB83897	26	M	Vestland	West	Sekret	Urethra	2023-04-25	0,5	0,016	0,016	0,004	0,25	16	1	NA	Negativ	[illegible]
23GB001010.fasta	23GB001010	SAMEA117568416	PRJEB83897	20	F	Vestland	West	Sekret	Cervix uter	2023-04-25	0,5	0,016	0,016	0,004	0,25	16	1	NA	Negativ	[illegible]
23GB001018.fasta	23GB001018	SAMEA117568423	PRJEB83897	20	M	Viken	East	Sekret	Urethra	2023-04-26	0,5	0,016	0,002	0,002	0,125	32	0,5	NA	Negativ	[illegible]
23GB001047.fasta	23GB001047	SAMEA117568434	PRJEB83897	25	M	Trøndelag	North	Sekret	Urethra	2023-04-28	0,5	0,016	0,016	0,004	0,25	16	1	NA	Negativ	[illegible]
23GB001056.fasta	23GB001056	SAMEA117568435	PRJEB83897	39	M	Vestland	West	Sekret	Anus	2023-04-26	0,5	0,016	0,016	0,004	0,25	16	1	NA	Negativ	[illegible]
23GB001058.fasta	23GB001058	SAMEA117568437	PRJEB83897	22	M	Vestland	West	Sekret	Hals	2023-04-28	1	0,016	0,016	0,004	0,125	16	1	NA	Negativ	[illegible]
529779.fasta	529779	SAMEA5608256	PRJEB32435	23	M	Oslo	East	Sekret	Hals	2016-04-05	0,5	0,016	0,004	0,004	0,064	16	0,5	NA	NA	[illegible]
530801.fasta	530801	SAMEA5608263	PRJEB32435	35	F	Vestfold og Telemark	East	Sekret	Vagina	2016-04-19	0,5	0,016	0,002	0,004	0,064	16	0,5	NA	NA	[illegible]
531223.fasta	531223	SAMEA5608265	PRJEB32435	20	M	Oslo	East	Sekret	Hals	2016-04-19	0,5	0,016	0,004	0,004	0,125	16	0,5	NA	NA	[illegible]
552460.fasta	552460	SAMEA5608362	PRJEB32435	25	F	Viken	East	Sekret	Cervix uter	2016-01-06	0,5	0,016	0,004	0,004	0,25	16	0,5	NA	NA	[illegible]
552591.fasta	552591	SAMEA5608376	PRJEB32435	52	M	Vestland	West	Sekret	Urethra	2016-02-17	1	0,016	0,004	0,004	0,25	16	0,5	NA	NA	[illegible]
553615.fasta	553615	SAMEA5608413	PRJEB32435	27	M	Oslo	East	Sekret	Urethra	2016-05-31	0,5	0,016	0,008	0,004	0,25	16	0,5	NA	NA	[illegible]
554641.fasta	554641	SAMEA5608463	PRJEB32435	37	M	Oslo	East	Sekret	Anus	2016-08-12	0,5	0,016	0,008	0,004	0,125	8	0,5	NA	NA	[illegible]
555845.fasta	555845	SAMEA5608519	PRJEB32435	27	M	Oslo	East	Sekret	Anus	2016-09-09	0,5	0,016	0,008	0,004	0,125	16	1	NA	NA	[illegible]
556830.fasta	556830	SAMEA5608530	PRJEB32435	33	M	Oslo	East	Sekret	Urethra	2016-10-24	0,5	0,016	0,004	0,004	0,125	16	0,5	NA	NA	[illegible]
556835.fasta	556835	SAMEA5608532	PRJEB32435	27	M	Viken	East	Sekret	Anus	2016-11-01	0,5	0,016	0,008	0,004	0,125	16	0,5	NA	NA	[illegible]
556853.fasta	556853	SAMEA5608538	PRJEB32435	29	M	Oslo	East	Sekret	Anus	2016-11-04	0,25	0,016	0,004	0,004	0,064	16	0,5	NA	NA	[illegible]
556857.fasta	556857	SAMEA5608539	PRJEB32435	28	M	Oslo	East	Sekret	Anus	2016-11-04	0,5	0,016	0,004	0,004	0,125	16	0,5	NA	NA	[illegible]
556880.fasta	556880	SAMEA5608544	PRJEB32435	28	M	Oslo	East	Sekret	Anus	2016-11-15	0,5	0,016	0,004	0,004	0,125	16	0,5	NA	NA	[illegible]
558650.fasta	558650	SAMEA5608557	PRJEB32435	49	M	Viken	East	Sekret	Urethra	2016-12-19	0,25	0,016	0,002	0,004	0,125	16	0,5	NA	NA	[illegible]
559716.fasta	559716	SAMEA5608563	PRJEB32435	42	M	Viken	East	Sekret	Hals	2016-12-29	0,5	0,016	0,004	0,008	0,125	16	1	NA	NA	[illegible]
560546.fasta	560546	SAMEA5608566	PRJEB32435	26	M	Oslo	East	Sekret	Anus	2017-01-02	0,5	0,016	0,004	0,004	0,125	16	0,5	NA	NA	[illegible]
560642.fasta	560642	SAMEA5608570	PRJEB32435	25	M	Oslo	East	Sekret	Hals	2017-01-02	0,5	0,016	0,004	0,004	0,125	16	1	NA	NA	[illegible]
560978.fasta	560978	SAMEA5608574	PRJEB32435	25	M	Oslo	East	Sekret	Anus	2017-01-04	0,5	0,016	0,004	0,004	0,125	16	1	NA	NA	[illegible]
561732.fasta	561732	SAMEA5608581	PRJEB32435	28	M	Nordland	North	Sekret	Urethra	2016-12-31	0,5	0,016	0,004	0,004	0,125	16	1	NA	NA	[illegible]
561752.fasta	561752	SAMEA5608583	PRJEB32435	29	M	Innlandet	East	Sekret	Urethra	2017-01-12	0,5	0,016	0,004	0,004	0,125	16	0,5	NA	NA	[illegible]
561755.fasta	561755	SAMEA5608584	PRJEB32435	48	M	Oslo	East	Sekret	Urethra	2017-01-12	0,5	0,016	0,004	0,004	0,125	16	1	NA	NA	[illegible]
562694.fasta	562694	SAMEA5608597	PRJEB32435	28	F	Oslo	East	Sekret	Vagina	2017-01-16	0,5	0,016	0,004	0,004	0,125	8	0,5	NA	NA	[illegible]
563155.fasta	563155	SAMEA5608607	PRJEB32435	35	M	Oslo	East	Sekret	Anus	2017-01-17	0,5	0,016	0,004	0,004	0,125	16	1	NA	NA	[illegible]
563160.fasta	563160	SAMEA5608608	PRJEB32435	28	M	Oslo	East	Sekret	Anus	2017-01-17	0,5	0,016	0,004	0,004	0,125	16	1	NA	NA	[illegible]
563194.fasta	563194	SAMEA5608610	PRJEB32435	37	M	Oslo	East	Sekret	Urethra	2017-01-17	0,5	0,016	0,004	0,004	0,125	16	0,5	NA	NA	[illegible]
563210.fasta	563210	SAMEA5608614	PRJEB32435	25	M	Oslo	East	Sekret	Anus	2017-01-18	0,5	0,016	0,004	0,004	0,125	16	0,5	NA	NA	[illegible]
563681.fasta	563681	SAMEA6533345	PRJEB32435	46	M	Oslo	East	Sekret	Urethra	2016-11-28	0,5	0,016	0,004	0,004	0,125	16	0,5	NA	NA	[illegible]
563695.fasta	563695	SAMEA6533351	PRJEB32435	22	M	Oslo	East	Sekret	Urethra	2016-12-02	0,5	0,016	0,002	0,004	0,125	16	0,5	NA	NA	[illegible]
563704.fasta	563704	SAMEA6533353	PRJEB32435	28	M	Oslo	East	Sekret	Urethra	2016-12-02	0,5	0,016	0,002	0,004	0,064	16	0,5	NA	NA	[illegible]
563724.fasta	563724	SAMEA6533357	PRJEB32435	29	M	Oslo	East	Sekret	Anus	2016-12-06	0,5	0,016	0,008	0,004	0,25	16	0,5	NA	NA	[illegible]
563766.fasta	563766	SAMEA6533367	PRJEB32435	51	M	Oslo	East	Sekret	Anus	2016-12-21	0,5	0,016	0,004	0,004	0,25	16	1	NA	NA	[illegible]
564104.fasta	564104	SAMEA5608621	PRJEB32435	29	M	Oslo	East	Sekret	Hals	2017-01-23	0,5	0,016	0,004	0,004	0,125	16	1	NA	NA	[illegible]
564538.fasta	564538	SAMEA5608623	PRJEB32435	56	M	Oslo	East	Sekret	Urethra	2017-01-30	1	0,016	0,004	0,004	0,125	16	0,5	NA	NA	[illegible]
564548.fasta	564548	SAMEA5608625	PRJEB32435	26	M	Oslo	East	Sekret	Urethra	2017-01-31	0,5	0,016	0,008	0,004	0,25	16	0,5	NA	NA	[illegible]
564704.fasta	564704	SAMEA5608626	PRJEB32435	54	M	Vestland	West	Sekret	Urethra	2017-02-01	1	0,016	0,004	0,004	0,25	16	1	NA	NA	[illegible]
564734.fasta	564734	SAMEA5608633	PRJEB32435	46	M	Oslo	East	Sekret	Urethra	2017-01-26	0,5	0,016	0,004	0,002	0,25	16	1	NA	NA	[illegible]
565102.fasta	565102	SAMEA5608638	PRJEB32435	48	M	Viken	East	Sekret	Hals	2017-02-02	0,5	0,016	0,004	0,004	0,125	16	0,5	NA	NA	[illegible]
565162.fasta	565162	SAMEA5608640	PRJEB32435	35	M	Oslo	East	Sekret	Anus	2017-02-02	0,5	0,016	0,004	0,004	0,125	16	0,5	NA	NA	[illegible]
565166.fasta	565166	SAMEA5608641	PRJEB32435	22	M	Oslo	East	Sekret	Hals	2017-01-31	1	0,016	0,004	0,004	0,125	16	0,5	NA	NA	[illegible]
565172.fasta	565172	SAMEA5608643	PRJEB32435	35	M	Oslo	East	Sekret	Anus	2017-02-03	1	0,016	0,004	0,004	0,25	16	0,5	NA	NA	[illegible]
565707.fasta	565707	SAMEA5608649	PRJEB32435	40	M	Oslo	East	Sekret	Hals	2017-02-08	0,25	0,016	0,002	0,004	0,125	8	0,5	NA	NA	[illegible]
565709.fasta	565709	SAMEA5608650	PRJEB32435	27	M	Oslo	East	Sekret	Anus	2017-02-09	0,25	0,016	0,004	0,004	0,125	16	0,5	NA	NA	[illegible]
566485.fasta	566485	SAMEA5608659	PRJEB32435	28	M	Oslo	East	Sekret	Hals	2017-02-13	0,5	0,016	0,004	0,004	0,25	16	0,5	NA	NA	[illegible]
567326.fasta	567326	SAMEA5608675	PRJEB32435	44	M	Viken	East	Sekret	Hals	2017-02-17	0,5	0,016	0,002	0,004	0,125	16	0,5	NA	NA	[illegible]
567377.fasta	567377	SAMEA5608681	PRJEB32435	22	M	Oslo	East	Sekret	Anus	2017-02-20	0,5	0,016	0,004	0,004	0,064	16	1	NA	NA	[illegible]
567616.fasta	567616	SAMEA5608685	PRJEB32435	38	M	Oslo	East	Sekret	Anus	2017-02-22	1	0,016	0,004	0,004	0,25	16	1	NA	NA	[illegible]
568630.fasta	568630	SAMEA5608694	PRJEB32435	34	M	Oslo	East	Sekret	Hals	2017-02-27	0,5	0,016	0,002	0,004	0,125	8	0,5	NA	NA	[illegible]
568633.fasta	568633	SAMEA5608695	PRJEB32435	26	M	Oslo	East	Sekret	Hals	2017-02-27	1	0,016	0,004	0,004	0,125	16	0,5	NA	NA	[illegible]
568645.fasta	568645	SAMEA5608697	PRJEB32435	28	M	Oslo	East	Sekret	Hals	2017-02-27	0,5	0,016	0,004	0,004	0,125	16	0,5	NA	NA	[illegible]
568828.fasta	568828	SAMEA5608700	PRJEB32435	25	M	Oslo	East	Sekret	Hals	2017-02-28	0,5	0,016	0,004	0,004	0,125	16	1	NA	NA	[illegible]
569227.fasta	569227	SAMEA5608704	PRJEB32435	35	M	Oslo	East	Sekret	Anus	2017-03-02	0,5	0,016	0,004	0,004	0,125	16	1	NA	NA	[illegible]
569456.fasta	569456	SAMEA5608710	PRJEB32435	41	M	Oslo	East	Sekret	Ureter	2017-03-09	0,5	0,016	0,004	0,004	0,125	8	1	NA	NA	[illegible]
571516.fasta	571516	SAMEA5608722	PRJEB32435	33	M	Oslo	East	Sekret	Anus	2017-03-16	1	0,016	0,002	0,004	0,125	16	0,5	NA	NA	[illegible]
571535.fasta	571535	SAMEA5608726	PRJEB32435	34	M	Oslo	East	Sekret	Urethra	2017-03-17	0,5	0,016	0,004	0,004	0,25	8	0,5	NA	NA	[illegible]
571541.fasta	571541	SAMEA5608727	PRJEB32435	27	M	Oslo	East	Sekret	Anus	2017-03-13	0,5	0,016	0,004	0,004	0,125	16	0,5	NA	NA	[illegible]
571551.fasta	571551	SAMEA5608732	PRJEB32435	43	M	Oslo	East	Sekret	Anus	2017-03-13	0,5	0,016	0,004	0,004	0,25	16	1	NA	NA	[illegible]
571559.fasta	571559	SAMEA5608736	PRJEB32435	23	M	Oslo	East	Sekret	Hals	2017-03-13	0,5	0,016	0,004	0,008	0,25	16	1	NA	NA	[illegible]
572276.fasta	572276	SAMEA5608740	PRJEB32435	54	M	Oslo	East	Sekret	Urethra	2017-03-24	0,5	0,016	0,004	0,004	0,125	16	0,5	NA	NA	[illegible]
573136.fasta	573136	SAMEA5608750	PRJEB32435	25	M	Viken	East	Sekret	Hals	2017-03-30	1	0,016	0,004	0,004	0,25	16	1	NA	NA	[illegible]
573321.fasta	573321	SAMEA5608752	PRJEB32435	46	M	Oslo	East	Sekret	Urethra	2017-04-03	0,5	0,016	0,002	0,004	0,125	16	0,5	NA	NA	[illegible]
573567.fasta	573567	SAMEA5608762	PRJEB32435	24	M	Trøndelag	North		Hals	2017-04-03	0,5	0,016	0,002	0,004	0,125	16	0,5	NA	NA	[illegible]
574847.fasta	574847	SAMEA5608778	PRJEB32435	38	M	Oslo	East	Sekret	Hals	2017-04-20	0,5	0,016	0,002	0,004	0,125	16	1	NA	NA	[illegible]
574850.fasta	574850	SAMEA5608779	PRJEB32435	41	M	Viken	East	Sekret	Hals	2017-04-20	0,5	0,016	0,002	0,004	0,125	16	0,5	NA	NA	[illegible]
574859.fasta	574859	SAMEA5608781	PRJEB32435	51	M	Oslo	East	Sekret	Hals	2017-04-19	0,5	0,016	0,004	0,004	0,25	16	1	NA	NA	[illegible]
575772.fasta	575772	SAMEA5608785	PRJEB32435	39	M	Oslo	East	Sekret	Anus	2017-04-24	0,5	0,016	0,002	0,004	0,064	16	1	NA	NA	[illegible]
575775.fasta	575775	SAMEA5608786	PRJEB32435	42	M	Oslo	East	Sekret	Anus	2017-04-24	0,5	0,016	0,002	0,004	0,125	16	0,5	NA	NA	[illegible]
575784.fasta	575784	SAMEA5608788	PRJEB32435	44	M	Vestfold og Telemark	East	Sekret	Anus	2017-04-26	0,5	0,016	0,002	0,004	0,125	16	1	NA	NA	[illegible]
575788.fasta	575788	SAMEA5608790	PRJEB32435	29	M	Oslo	East	Sekret	Anus	2017-04-28	0,5	0,016	0,004	0,004	0,25	16	1	NA	NA	[illegible]
577003.fasta	577003	SAMEA5608803	PRJEB32435	57	M	Oslo	East	Sekret	Urethra	2017-05-08	0,25	0,016	0,002	0,002	0,064	8	0,25	NA	NA	[illegible]
577128.fasta	577128	SAMEA5608809	PRJEB32435	44	M	Viken	East	Sekret	Urethra	2017-05-09	0,5	0,016	0,002	0,004	0,125	8	0,5	NA	NA	[illegible]
577223.fasta	577223	SAMEA5608810	PRJEB32435	38	M	Oslo	East	Sekret	Anus	2017-05-12	0,5	0,016	0,002	0,004	0,125	16	1	NA	NA	[illegible]
577551.fasta	577551	SAMEA5608818	PRJEB32435	34	M	Oslo	East	Sekret	Urethra	2017-05-15	0,5	0,016	0,002	0,004	0,25	16	0,5	NA	NA	[illegible]
577555.fasta	577555	SAMEA5608819	PRJEB32435	26	F	Oslo	East	Sekret	Hals	2017-05-15	0,5	0,016	0,002	0,004	0,25	16	1	NA	NA	[illegible]
577883.fasta	577883	SAMEA5608822	PRJEB32435	24	M	Viken	East	Sekret	Anus	2017-05-12	0,5	0,016	0,002	0,004	0,125	16	1	NA	NA	[illegible]
577889.fasta	577889	SAMEA5608823	PRJEB32435	29	M	Vestland	West	Sekret	Hals	2017-05-12	0,25	0,016	0,002	0,004	0,125	16	0,5	NA	NA	[illegible]
578297.fasta	578297	SAMEA5608835	PRJEB32435	20	M	Viken	East	Sekret	Hals	2017-05-24	0,25	0,016	0,002	0,004	0,125	16	0,5	NA	NA	[illegible]
578697.fasta	578697	SAMEA5608837	PRJEB32435	31	M	Oslo	East	Sekret	Anus	2017-05-26	0,5	0,016	0,002	0,004	0,125	16	0,5	NA	NA	[illegible]
579028.fasta	579028	SAMEA5608840	PRJEB32435	27	M	Oslo	East	Sekret	Anus	2017-05-31	0,5	0,016	0,002	0,002	0,125	16	0,5	NA	NA	[illegible]
579030.fasta	579030	SAMEA5608841	PRJEB32435	22	M	Viken	East	Sekret	Hals	2017-05-29	0,5	0,016	0,002	0,002	0,125	16	0,5	NA	NA	[illegible]
579034.fasta	579034	SAMEA5608843	PRJEB32435	36	M	Oslo	East	Sekret	Anus	2017-05-29	0,5	0,016	0,002	0,004	0,25	16	0,5	NA	NA	[illegible]
580400.fasta	580400	SAMEA5608852	PRJEB32435	34	M	Rogaland	West	Sekret	Urethra	2017-06-09	0,5	0,016	0,004	0,004	0,125	16	0,5	NA	NA	[illegible]
590462.fasta	590462	SAMEA5608868	PRJEB32435	21	M	Viken	East	Sekret	Anus	2017-06-16	0,25	0,016	0,004	0,008	0,125	16	0,5	NA	NA	[illegible]
591434.fasta	591434	SAMEA5608885	PRJEB32435	32	M	Oslo	East	Sekret	Anus	2017-06-30	0,25	0,016	0,004	0,004	0,125	16	0,5	NA	NA	[illegible]
592487.fasta	592487	SAMEA5608909	PRJEB32435	28	M	Oslo	East	Sekret	Urethra	2017-07-10	0,5	0,016	0,004	0,004	0,25	16	0,5	NA	NA	[illegible]
592622.fasta	592622	SAMEA5608916	PRJEB32435	48	M	Oslo	East	Sekret	Urethra	2017-07-14	0,5	0,016	0,002	0,004	0,25	8	0,5	NA	NA	[illegible]
592628.fasta	592628	SAMEA5608920	PRJEB32435	49	M	Oslo	East	Sekret		2017-07-14	0,5	0,016	0,002	0,004	0,125	16	1	NA	NA	[illegible]
592900.fasta	592900	SAMEA5608924	PRJEB32435	26	M	Oslo	East	Sekret	Anus	2017-07-17	0,5	0,016	0,002	0,004	0,125	16	1	NA	NA	[illegible]
592915.fasta	592915	SAMEA5608929	PRJEB32435	25	M	Oslo	East	Sekret	Urethra	2017-07-17	0,5	0,016	0,004	0,004	0,25	16	0,5	NA	NA	[illegible]
593235.fasta	593235	SAMEA5608938	PRJEB32435	24	M	Oslo	East	Sekret	Hals	2017-07-21	0,5	0,016	0,002	0,004	0,125	16	1	NA	NA	[illegible]
593785.fasta	593785	SAMEA5608954	PRJEB32435	56	F	NA	NA	Sekret	Urethra	2017-07-26	0,5	0,016	0,004	0,004	0,064	8	1	NA	NA	[illegible]
593788.fasta	593788	SAMEA5608955	PRJEB32435	57	M	Møre og Romsdal	West	Sekret	Urethra	2017-07-26	0,25	0,016	0,002	0,004	0,125	16	1	NA	NA	[illegible]
593973.fasta	593973	SAMEA5608959	PRJEB32435	28	M	Oslo	East	Sekret	Anus	2017-07-24	0,5	0,016	0,002	0,002	0,125	16	0,5	NA	NA	[illegible]
594138.fasta	594138	SAMEA5608966	PRJEB32435	30	F	Oslo	East	Puss	Abdomen	2017-07-28	0,5	0,016	0,002	0,004	0,125	16	1	NA	NA	[illegible]
594498.fasta	594498	SAMEA5608974	PRJEB32435	30	M	Viken	East	Sekret	Anus	2017-08-02	0,5	0,016	0,002	0,002	0,064	8	0,5	NA	NA	[illegible]
595638.fasta	595638	SAMEA5608989	PRJEB32435	18	M	Viken	East	Sekret	Anus	2017-08-07	0,5	0,016	0,004	0,004	0,125	16	0,5	NA	NA	[illegible]
598786.fasta	598786	SAMEA5609047	PRJEB32435	61	M	Oslo	East	Sekret	Urethra	2017-09-13	0,5	0,016	0,002	0,004	0,064	8	0,5	NA	NA	[illegible]
599991.fasta	599991	SAMEA5609062	PRJEB32435	27	M	Oslo	East	Sekret	Anus	2017-09-25	0,25	0,016	0,002	0,002	0,064	8	0,5	NA	NA	[illegible]
600743.fasta	600743	SAMEA5609077	PRJEB32435	41	M	Oslo	East	Sekret	Anus	2017-10-02	0,5	0,016	0,002	0,004	0,125	16	1	NA	NA	[illegible]
602142.fasta	602142	SAMEA5609102	PRJEB32435	36	M	Oslo	East	Sekret	Hals	2017-10-16	0,5	0,016	0,004	0,004	0,125	16	0,5	NA	NA	[illegible]
603155.fasta	603155	SAMEA5609109	PRJEB32435	24	M	Oslo	East	Sekret		2017-10-23	0,25	0,016	0,002	0,004	0,125	8	0,5	NA	NA	[illegible]
605704.fasta	605704	SAMEA5609140	PRJEB32435	36	M	Oslo	East	Sekret	Anus	2017-11-14	0,25	0,016	0,002	0,004	0,064	16	0,5	NA	NA	[illegible]
612594.fasta	612594	SAMEA6533435	PRJEB32435	35	M	NA	NA	Sekret	Anus	2018-01-10	0,5	0,016	0,002	0,004	0,125	16	0,5	NA	NA	[illegible]
612678.fasta	612678	SAMEA6533437	PRJEB32435	49	M	Vestland	West	Sekret	Anus	2018-01-11	0,5	0,016	0,002	0,004	0,125	16	1	NA	NA	[illegible]
613857.fasta	613857	SAMEA6533445	PRJEB32435	33	M	Oslo	East	Sekret	Urethra	2018-01-18	0,25	0,016	0,002	0,002	0,064	8	0,25	NA	NA	[illegible]
617846.fasta	617846	SAMEA6533468	PRJEB32435	22	M	Agder	South	Sekret	Rektum	2018-02-09	1	0,016	0,002	0,004	0,125	16	0,5	NA	NA	[illegible]
617919.fasta	617919	SAMEA6963047	PRJEB32435	43	M	Vestfold og Telemark	East	Sekret	Urethra	2018-02-13	0,5	0,016	0,004	0,004	0,25	16	0,5	NA	NA	[illegible]
618203.fasta	618203	SAMEA6533470	PRJEB32435	43	M	Agder	South	Sekret	Anus	2018-02-09	0,5	0,016	0,004	0,004	0,125	16	0,5	NA	NA	[illegible]
618400.fasta	618400	SAMEA6963048	PRJEB32435	26	M	Oslo	East	Sekret	Urethra	2018-02-12	1	0,016	0,004	0,004	0,25	16	1	NA	NA	[illegible]
618606.fasta	618606	SAMEA6533476	PRJEB32435	27	M	Oslo	East	Sekret	Anus	2018-02-14	1	0,016	0,004	0,004	0,5	8	1	NA	NA	[illegible]
623226.fasta	623226	SAMEA6533508	PRJEB32435	32	M	NA	NA	SEKRET	URETRA	2018-03-23	NA	NA	NA	NA	NA	NA	NA	NA	NA	[illegible]
623273.fasta	623273	SAMEA6533509	PRJEB32435	36	M	NA	NA	SEKRET	URETRA	2018-03-20	NA	NA	NA	NA	NA	NA	NA	NA	NA	[illegible]
625367.fasta	625367	SAMEA6533530	PRJEB32435	44	M	NA	NA	SEKRET	ANUS	2018-04-09	0,25	0,016	0,002	0,004	0,125	16	0,5	-	NA	[illegible]
625953.fasta	625953	SAMEA6533540	PRJEB32435	29	M	NA	NA	BKULTUR	ANUS	2018-04-11	0,5	0,016	0,004	0,002	0,5	8	0,5	-	NA	[illegible]
629717.fasta	629717	SAMEA6533595	PRJEB32435	50	M	NA	NA	SEKRET	URETRA	2018-05-22	NA	NA	NA	NA	NA	NA	NA	NA	NA	[illegible]
630156.fasta	630156	SAMEA6963055	PRJEB32435	64	M	NA	NA	SEKRET	ANUS	2018-05-28	1	0,016	0,002	0,004	0,125	16	0,5	-	NA	[illegible]
630948.fasta	630948	SAMEA6533612	PRJEB32435	25	M	NA	NA	SEKRET	ANUS	2018-06-07	NA	NA	NA	NA	NA	NA	NA	NA	NA	[illegible]
631112.fasta	631112	SAMEA6533617	PRJEB32435	44	M	NA	NA	SEKRET	HALS	2018-06-06	NA	NA	NA	NA	NA	NA	NA	NA	NA	[illegible]
632735.fasta	632735	SAMEA6533636	PRJEB32435	25	M	NA	NA	SEKRET	HALS	2018-06-25	NA	NA	NA	NA	NA	NA	NA	NA	NA	[illegible]
632817.fasta	632817	SAMEA6533638	PRJEB32435	53	M	NA	NA	SEKRET	ANUS	2018-06-29	NA	NA	NA	NA	NA	NA	NA	NA	NA	[illegible]
633178.fasta	633178	SAMEA6533653	PRJEB32435	23	M	NA	NA	SEKRET	URETRA	2018-07-04	NA	NA	NA	NA	NA	NA	NA	NA	NA	[illegible]
633563.fasta	633563	SAMEA6533658	PRJEB32435	31	M	NA	NA	SEKRET	URETRA	2018-07-05	NA	NA	NA	NA	NA	NA	NA	NA	NA	[illegible]
633593.fasta	633593	SAMEA6533665	PRJEB32435	26	M	NA	NA	SEKRET	URETRA	2018-07-04	NA	NA	NA	NA	NA	NA	NA	NA	NA	[illegible]
634188.fasta	634188	SAMEA6533683	PRJEB32435	20	M	NA	NA	SEKRET	URETRA	2018-07-11	NA	NA	NA	NA	NA	NA	NA	NA	NA	[illegible]
634406.fasta	634406	SAMEA6533701	PRJEB32435	27	M	NA	NA	SEKRET	HALS	2018-07-19	NA	NA	NA	NA	NA	NA	NA	NA	NA	[illegible]
637429.fasta	637429	SAMEA6533776	PRJEB32435	19	M	NA	NA	SEKRET	ANUS	2018-08-27	NA	NA	NA	NA	NA	NA	NA	NA	NA	[illegible]
637522.fasta	637522	SAMEA6533782	PRJEB32435	39	M	NA	NA	SEKRET	ANUS	2018-08-29	NA	NA	NA	NA	NA	NA	NA	NA	NA	[illegible]
637613.fasta	637613	SAMEA6533787	PRJEB32435	27	M	NA	NA	SEKRET	HALS	2018-08-30	NA	NA	NA	NA	NA	NA	NA	NA	NA	[illegible]
638274.fasta	638274	SAMEA6533809	PRJEB32435	20	M	NA	NA	SEKRET	URETRA	2018-09-06	0,75	0,016	0,016	0,002	NA	NA	NA	NA	NA	[illegible]
639447.fasta	639447	SAMEA6533832	PRJEB32435	22	M	NA	NA	SEKRET	URETRA	2018-09-17	0,5	0,016	0,016	0,002	NA	NA	NA	NA	NA	[illegible]
639450.fasta	639450	SAMEA6533833	PRJEB32435	24	F	NA	NA	SEKRET	CERVIXUT	2018-09-20	0,75	0,016	0,016	0,002	NA	NA	NA	NA	NA	[illegible]
641799.fasta	641799	SAMEA6533884	PRJEB32435	72	M	NA	NA	SEKRET	HALS	2018-10-15	0,75	0,016	0,016	0,002	NA	NA	NA	NA	NA	[illegible]
642299.fasta	642299	SAMEA6533893	PRJEB32435	25	M	NA	NA	SEKRET	HALS	2018-10-19	1	0,016	0,016	0,003	NA	NA	NA	NA	NA	[illegible]
643829.fasta	643829	SAMEA6533931	PRJEB32435	52	M	NA	NA	SEKRET	HALS	2018-11-07	NA	NA	NA	NA	NA	NA	NA	NA	NA	[illegible]
645929.fasta	645929	SAMEA6533958	PRJEB32435	31	M	NA	NA	SEKRET	URETRA	2018-11-23	NA	NA	NA	NA	NA	NA	NA	NA	NA	[illegible]
660436.fasta	660436	SAMEA6534167	PRJEB32435	63	M	Viken	East	Sekret	Anus	2019-04-08	0,5	0,016	0,002	0,004	0,125	16	0,5	Ikke utført	Ikke påvist	[illegible]
662312.fasta	662312	SAMEA6534201	PRJEB32435	45	M	Oslo	East	Sekret	Urethra	2019-04-29	0,5	0,016	0,002	0,002	0,125	16	0,25	4	Ikke utført	[illegible]
667832.fasta	667832	SAMEA6534439	PRJEB32435	23	M	Oslo	East	Sekret	Anus	2019-07-10	0,5	0,016	0,004	0,004	0,25	16	0,25	4	Ikke påvist	[illegible]
668211.fasta	668211	SAMEA6534458	PRJEB32435	25	M	Oslo	East	Sekret	Urethra	2019-07-15	1	0,016	0,002	0,004	0,25	16	0,5	4	Ikke påvist	[illegible]
670827.fasta	670827	SAMEA6534540	PRJEB32435	33	M	Oslo	East	Sekret	Anus	2019-08-20	1	0,016	0,002	0,004	0,25	8	1	8	Ikke påvist	[illegible]
675999.fasta	675999	SAMEA6963077	PRJEB32435	25	M	Viken	East	Sekret	Urethra	2019-10-28	1	0,016	0,004	0,004	0,25	16	0,5	8	Ikke påvist	[illegible]
676105.fasta	676105	SAMEA6534663	PRJEB32435	75	M	Viken	East	Sekret	Urethra	2019-11-02	0,032	0,016	0,016	0,002	0,064	8	0,5	8	Ikke påvist	[illegible]
676261.fasta	676261	SAMEA6534665	PRJEB32435	38	M	Oslo	East	Sekret	Urethra	2019-10-31	0,25	0,016	0,004	0,004	0,064	8	0,5	8	Ikke påvist	[illegible]
677499.fasta	677499	SAMEA6534701	PRJEB32435	30	M	Oslo	East	Sekret	Anus	2019-11-15	0,25	0,016	0,004	0,008	0,125	0,032	0,5	4	Ikke påvist	[illegible]
678132.fasta	678132	SAMEA6534710	PRJEB32435	24	M	Oslo	East	Bakterieku	Hals	2019-11-18	0,25	0,016	0,004	0,004	0,125	8	0,25	8	Ikke påvist	[illegible]
678566.fasta	678566	SAMEA6534722	PRJEB32435	27	M	Oslo	East	Sekret	Anus	2019-11-29	0,125	0,016	0,008	0,002	0,125	16	0,064	8	Ikke påvist	[illegible]
678674.fasta	678674	SAMEA6534726	PRJEB32435	27	M	Viken	East	Sekret	Urethra	2019-11-28	0,5	0,016	0,002	0,004	0,125	8	0,5	8	Ikke påvist	[illegible]
678910.fasta	678910	SAMEA6534737	PRJEB32435	24	M	Viken	East	Sekret	Urethra	2019-11-28	0,25	0,016	0,008	0,004	0,25	8	0,25	8	Ikke påvist	[illegible]
680041.fasta	680041	SAMEA117568459	PRJEB83897	33	M	Viken	East	Sekret	Urethra	2019-12-09	0,5	0,016	0,004	0,004	0,064	8	0,5	8	Ikke påvist	[illegible]
547917.fasta	547917	SAMEA5608345	PRJEB32435	23	M	Viken	East	Sekret	Urethra	2016-09-22	1	0,016	0,008	0,008	0,25	16	1	NA	NA	[illegible]
570009.fasta	570009	SAMEA5608715	PRJEB32435	22	M	Oslo	East	Sekret	Anus	2017-03-10	0,5	0,016	0,002	0,004	0,125	16	1	NA	NA	[illegible]
603442.fasta	603442	SAMEA5609116	PRJEB32435	30	M	Agder	South	Sekret	Urethra	2017-10-24	0,5	0,016	0,002	0,004	0,25	16	0,5	NA	NA	[illegible]
21GB000508.fasta	21GB000508	SAMEA117567075	PRJEB83897	32	M	Oslo	East	Sekret	Anus	2021-09-10	1	0,016	0,002	0,004	0,125	8	0,25	NA	Ikke påvist	[illegible]
21GB000550.fasta	21GB000550	SAMEA117567089	PRJEB83897	37	M	Oslo	East	Sekret	Anus	2021-09-21	0,25	0,016	0,002	0,002	0,064	4	0,25	NA	Ikke påvist	[illegible]
21GB000592.fasta	21GB000592	SAMEA117567098	PRJEB83897	22	M	Troms og Finnmark	North	Sekret	Anus	2021-10-05	0,25	0,016	0,004	0,002	0,125	4	0,25	NA	Ikke påvist	[illegible]
21GB000598.fasta	21GB000598	SAMEA117567102	PRJEB83897	23	F	Oslo	East	Sekret	Urethra	2021-10-07	0,5	0,016	0,002	0,004	0,125	8	0,125	NA	Ikke påvist	[illegible]

21GB000606.fasta	21GB000606	SAMEA117567104	PRJEB83897	30	M	Oslo	East	Sekret	Hals	2021-10-08	0,5	0,016	0,002	0,002	0,125	8	0,25	NA	Ikke påvist	15987	1	18	2620	NOT_FOUND	NOT_FOUND	NOT_FOUND	NOT_FOUND	INTERMEDIATE	RESISTANT	NOT_FOUND	INTERMEDIATE	folP_R228SmtrR_A39TpenA_ins346DrpsJ_V57M		0	322
21GB000607.fasta	21GB000607	SAMEA117567105	PRJEB83897	22	M	Oslo	East	Sekret	Urethra	2021-10-11	1	0,016	0,002	0,002	0,125	8	0,25	NA	Ikke påvist	15987	1	18	2620	NOT_FOUND	NOT_FOUND	NOT_FOUND	NOT_FOUND	INTERMEDIATE	RESISTANT	NOT_FOUND	INTERMEDIATE	folP_R228SmtrR_A39TpenA_ins346DrpsJ_V57M		0	322
21GB000755.fasta	21GB000755	SAMEA117567140	PRJEB83897	30	M	Oslo	East	Sekret	Anus	2021-11-23	0,5	0,016	0,002	0,004	0,064	8	0,125	NA	Ikke påvist	15987	1	18	2620	NOT_FOUND	NOT_FOUND	NOT_FOUND	NOT_FOUND	INTERMEDIATE	RESISTANT	NOT_FOUND	INTERMEDIATE	folP_R228SmtrR_A39TpenA_ins346DrpsJ_V57M		0	322
21GB000767.fasta	21GB000767	SAMEA117567144	PRJEB83897	31	M	Oslo	East	Sekret	Anus	2021-11-26	0,5	0,016	0,002	0,004	0,125	16	0,25	NA	Ikke påvist	15987	1	18	2620	NOT_FOUND	NOT_FOUND	NOT_FOUND	NOT_FOUND	INTERMEDIATE	RESISTANT	NOT_FOUND	INTERMEDIATE	folP_R228SmtrR_A39TpenA_ins346DrpsJ_V57M		0	322
21GB000798.fasta	21GB000798	SAMEA117567152	PRJEB83897	25	M	Oslo	East	Sekret	Urethra	2021-12-01	0,25	0,016	0,002	0,002	0,125	8	0,125	NA	Ikke påvist	15987	1	18	2620	NOT_FOUND	NOT_FOUND	NOT_FOUND	NOT_FOUND	INTERMEDIATE	RESISTANT	NOT_FOUND	INTERMEDIATE	folP_R228SmtrR_A39TpenA_ins346DrpsJ_V57M		0	322
21GB000826.fasta	21GB000826	SAMEA117567163	PRJEB83897	38	M	Oslo	East	Sekret	Urethra	2021-12-10	0,25	0,016	0,002	0,002	0,125	8	0,125	NA	Ikke påvist	15987	1	18	2620	NOT_FOUND	NOT_FOUND	NOT_FOUND	NOT_FOUND	INTERMEDIATE	RESISTANT	NOT_FOUND	INTERMEDIATE	folP_R228SmtrR_A39TpenA_ins346DrpsJ_V57M		0	322
21GB000827.fasta	21GB000827	SAMEA117567164	PRJEB83897	29	M	Oslo	East	Sekret	Anus	2021-12-10	0,25	0,016	0,002	0,002	0,125	8	0,125	NA	Ikke påvist	15987	1	18	2620	NOT_FOUND	NOT_FOUND	NOT_FOUND	NOT_FOUND	INTERMEDIATE	RESISTANT	NOT_FOUND	INTERMEDIATE	folP_R228SmtrR_A39TpenA_ins346DrpsJ_V57M		0	322
21GB000828.fasta	21GB000828	SAMEA117567165	PRJEB83897	40	M	Oslo	East	Sekret	Hals	2021-12-10	0,5	0,016	0,002	0,002	0,064	8	0,002	NA	Ikke påvist	15987	1	18	2620	NOT_FOUND	NOT_FOUND	NOT_FOUND	NOT_FOUND	INTERMEDIATE	RESISTANT	NOT_FOUND	INTERMEDIATE	folP_R228SmtrR_A39TpenA_ins346DrpsJ_V57M		0	322
22GB000628.fasta	22GB000628	SAMEA117567388	PRJEB83897	30	M	Agder	South	Sekret	Hals	2022-05-20	0,5	0,016	0,016	0,004	0,064	16	1	4	Negativ	15987	1	18	2620	NOT_FOUND	NOT_FOUND	NOT_FOUND	NOT_FOUND	INTERMEDIATE	RESISTANT	NOT_FOUND	INTERMEDIATE	folP_R228SmtrR_A39TpenA_ins346DrpsJ_V57M		0	322
616689.fasta	616689	SAMEA6533459	PRJEB32435	35	M	Oslo	East	Ikke angitt		2018-02-05	0,5	0,016	0,004	0,004	0,5	16	0,5	NA	NA	8134	1	18	63	NOT_FOUND	NOT_FOUND	NOT_FOUND	NOT_FOUND	INTERMEDIATE	RESISTANT	NOT_FOUND	INTERMEDIATE	folP_R228SmtrR_A39TpenA_ins346DrpsJ_V57M		0	322
669947.fasta	669947	SAMEA6534509	PRJEB32435	24	M	Oslo	East	Sekret	Urethra	2019-08-07	0,5	0,016	0,004	0,004	0,25	16	0,5	4	Ikke påvist	8134	1	18	63	NOT_FOUND	NOT_FOUND	NOT_FOUND	NOT_FOUND	INTERMEDIATE	RESISTANT	NOT_FOUND	INTERMEDIATE	folP_R228SmtrR_A39TpenA_ins346DrpsJ_V57M		0	322
22GB001549.fasta	22GB001549	SAMEA117567880	PRJEB83897	39	M	Rogaland	West	Sekret	Anus	2022-11-15	2	0,032	0,016	8	0,125	16	0,5	8	Negativ	-	1	18	f053dbfd249	NOT_FOUND	NOT_FOUND	NOT_FOUND	RESISTANT	NOT_FOUND	RESISTANT	NOT_FOUND	INTERMEDIATE	folP_R228SgyrA_D95AgyrA_S91FmtrR_A39TparC_S87RrpsJ_V57M		0	257
671256.fasta	671256	SAMEA6534559	PRJEB32435	33	M	Agder	South	Sekret	Anus	2019-08-13	0,5	0,016	0,004	0,004	0,125	16	0,5	4	Ikke påvist	-	1	18	b9212dbb309	NOT_FOUND	NOT_FOUND	NOT_FOUND	NOT_FOUND	INTERMEDIATE	NOT_FOUND	NOT_FOUND	INTERMEDIATE	mtrR_A39TpenA_ins346DrpsJ_V57M		0	256
555814.fasta	555814	SAMEA5608509	PRJEB32435	63	M	Oslo	East	Sekret	Urethra	2016-08-29	2	0,016	0,008	0,004	0,125	16	1	NA	NA	9363	1	18	301	NOT_FOUND	NOT_FOUND	NOT_FOUND	NOT_FOUND	INTERMEDIATE	RESISTANT	NOT_FOUND	INTERMEDIATE	folP_R228SmtrR_A39TpenA_ins346DrpsJ_V57M		0	228
555841.fasta	555841	SAMEA5608518	PRJEB32435	32	M	Oslo	East	Sekret	Urethra	2016-09-07	1	0,016	0,004	0,004	0,125	8	0,5	NA	NA	9363	1	18	301	NOT_FOUND	NOT_FOUND	NOT_FOUND	NOT_FOUND	INTERMEDIATE	RESISTANT	NOT_FOUND	INTERMEDIATE	folP_R228SmtrR_A39TpenA_ins346DrpsJ_V57M		0	228
552894.fasta	552894	SAMEA5608401	PRJEB32435	32	M	Oslo	East	Sekret	Urethra	2016-03-16	0,032	0,016	0,004	0,004	0,25	8	0,5	NA	NA	11428	1	18	959	NOT_FOUND	NOT_FOUND	NOT_FOUND	NOT_FOUND	INTERMEDIATE	RESISTANT	NOT_FOUND	INTERMEDIATE	folP_R228SmtrR_G45DpenA_ins346DrpsJ_V57M		0	226
22GB000981.fasta	22GB000981	SAMEA117567567	PRJEB83897	55	M	Oslo	East	Abscessinn	Anus	2022-08-16	0,5	0,016	0,002	0,064	0,25	16	0,125	4	Negativ	11428	1	18	73457f4c96e	NOT_FOUND	NOT_FOUND	NOT_FOUND	RESISTANT	INTERMEDIATE	RESISTANT	NOT_FOUND	INTERMEDIATE	folP_R228SgyrA_D95AgyrA_S91FmtrR_A39TpenA_ins346DrpsJ_V57M		0	213
22GB001008.fasta	22GB001008	SAMEA117567586	PRJEB83897	55	M	Oslo	East	Sekret	Anus	2022-08-19	0,5	0,016	0,002	0,064	0,125	8	0,25	8	Negativ	11428	1	18	73457f4c96e	NOT_FOUND	NOT_FOUND	NOT_FOUND	RESISTANT	INTERMEDIATE	RESISTANT	NOT_FOUND	INTERMEDIATE	folP_R228SgyrA_D95AgyrA_S91FmtrR_A39TpenA_ins346DrpsJ_V57M		0	213
655344.fasta	655344	SAMEA6534097	PRJEB32435	29	M	Viken	East	Sekret	Urethra	2019-02-25	0,5	0,016	0,002	0,5	0,125	8	0,25	4	Ikke påvist	11428	1	18	e6005f5be7d	NOT_FOUND	NOT_FOUND	NOT_FOUND	RESISTANT	INTERMEDIATE	RESISTANT	NOT_FOUND	INTERMEDIATE	folP_R228SgyrA_D95GgyrA_S91FmtrR_A39TparC_D86NpenA_ins346DrpsJ_V57M		0	213
601695.fasta	601695	SAMEA5609092	PRJEB32435	53	M	Viken	East	Sekret	Urethra	2017-10-09	1	0,016	0,004	0,004	0,125	16	1	NA	NA	11428	1	18	63	NOT_FOUND	NOT_FOUND	NOT_FOUND	NOT_FOUND	INTERMEDIATE	RESISTANT	NOT_FOUND	INTERMEDIATE	folP_R228SmtrR_A39TpenA_ins346DrpsJ_V57M		0	213
626883.fasta	626883	SAMEA6963056	PRJEB32435	53	M	NA	NA	SEKRET	URETRA	2018-04-18	1	0,016	0,002	0,004	0,125	16	0,5	-	NA	11428	1	18	63	NOT_FOUND	NOT_FOUND	NOT_FOUND	NOT_FOUND	INTERMEDIATE	RESISTANT	NOT_FOUND	INTERMEDIATE	folP_R228SmtrR_A39TpenA_ins346DrpsJ_V57M		0	213
611749.fasta	611749	SAMEA6533426	PRJEB32435	35	M	NA	NA	Bakterieku	Urethra	2018-01-02	1	0,016	0,002	0,004	0,125	16	1	NA	NA	11428	1	18	246	NOT_FOUND	NOT_FOUND	NOT_FOUND	NOT_FOUND	INTERMEDIATE	RESISTANT	NOT_FOUND	RESISTANT	folP_R228SmtrR_promoter_a-57delpenA_ins346DrpsJ_V57M		0	211
634204.fasta	634204	SAMEA6533689	PRJEB32435	36	M	NA	NA	SEKRET	URETRA	2018-07-16	NA	NA	NA	NA	NA	NA	NA	NA	NA	11428	1	18	246	NOT_FOUND	NOT_FOUND	NOT_FOUND	NOT_FOUND	INTERMEDIATE	RESISTANT	NOT_FOUND	RESISTANT	folP_R228SmtrR_promoter_a-57delpenA_ins346DrpsJ_V57M		0	211
637737.fasta	637737	SAMEA6533789	PRJEB32435	58	M	NA	NA	SEKRET	ANUS	2018-08-30	NA	NA	NA	NA	NA	NA	NA	NA	NA	7827	6	28	28d0085856d	NOT_FOUND	NOT_FOUND	RESISTANT	RESISTANT	INTERMEDIATE	RESISTANT	NOT_FOUND	RESISTANT	folP_R228SgyrA_D95GgyrA_S91FmtrR_A39TmtrR_G45DparC_D86NpenA_G545SpenA_I312MpenA_V316TponA1_L4		0	196
662558.fasta	662558	SAMEA6534208	PRJEB32435	31	M	Oslo	East	Sekret	Hals	2019-05-02	0,125	0,125	0,016	1	0,5	16	1	4	Ikke påvist	7827	6	28	3720	NOT_FOUND	NOT_FOUND	RESISTANT	RESISTANT	INTERMEDIATE	RESISTANT	NOT_FOUND	INTERMEDIATE	folP_R228SgyrA_D95GgyrA_S91FmtrR_A39TparC_D86NpenA_G545SpenA_I312MpenA_V316TponA1_L421PrpsJ_V57		0	196
22GB000135.fasta	22GB000135	SAMEA117567241	PRJEB83897	22	M	Viken	East	Sekret	Urethra	2022-02-11	0,064	0,016	0,016	0,004	0,064	8	0,25	NA	Ikke påvist	1587	5	33	2206	NOT_FOUND	NOT_FOUND	RESISTANT	RESISTANT	RESISTANT	RESISTANT	NOT_FOUND	RESISTANT	folP_R228SgyrA_D95AgyrA_S91FmtrR_disruptedparC_D86NpenA_G545SpenA_I312MpenA_V316TrpsJ_V57M	blaTEMtetM		195
22GB001219.fasta	22GB001219	SAMEA117567706	PRJEB83897	24	M	Vestland	West	Sekret	Urethra	2022-09-27	2	0,016	0,016	0,004	0,25	16	1	8	Negativ	9363	1	18	1819	NOT_FOUND	NOT_FOUND	NOT_FOUND	RESISTANT	INTERMEDIATE	RESISTANT	NOT_FOUND	NOT_FOUND	folP_R228SmtrR_A39TparC_S87RpenA_ins346D		0	169
22GB000964.fasta	22GB000964	SAMEA117567553	PRJEB83897	21	M	Viken	East	Sekret	Urethra	2022-08-11	1	0,016	0,016	0,004	0,25	16	0,5	8	Negativ	9363	1	18	1819	NOT_FOUND	NOT_FOUND	NOT_FOUND	RESISTANT	INTERMEDIATE	RESISTANT	NOT_FOUND	INTERMEDIATE	folP_R228SmtrR_A39TparC_S87RpenA_ins346DrpsJ_V57M		0	169
[illegible]																																			

23GB001024.fasta	23GB001024	SAMEA117568429	PRJEB83897	73	M	Oslo	East	Sekret	Urethra	2023-04-28	0,064	0,016	0,002	1	8	16	16	NA	Positiv	11365	8	31	c3e2957cd5f4	NOT_FOUND	NOT_FOUND	NOT_FOUND	RESISTANT	INTERMEDIATE	RESISTANT
521175.fasta	521175	SAMEA5608237	PRJEB32435	39	F	Troms og Finnmark	North	Sekret	Cervix uter	2016-02-01	0,25	0,064	0,016	4	0,25	8	16	NA	NA	7822	12	24	957	NOT_FOUND	NOT_FOUND	NOT_FOUND	RESISTANT	INTERMEDIATE	RESISTANT
529017.fasta	529017	SAMEA5608247	PRJEB32435	36	M	Rogaland	West	Sekret	Urethra	2016-04-08	0,125	0,064	0,032	32	0,5	8	64	NA	NA	7823	12	24	557	NOT_FOUND	NOT_FOUND	NOT_FOUND	RESISTANT	INTERMEDIATE	RESISTANT
22GB001067.fasta	22GB001067	SAMEA117567623	PRJEB83897	28	F	Vestland	West	Sekret	Cervix uter	2022-08-29	0,25	0,125	0,064	32	1	16	64	8	Negativ	8123	6	28	507	NOT_FOUND	NOT_FOUND	RESISTANT	RESISTANT	RESISTANT	RESISTANT
22GB001148.fasta	22GB001148	SAMEA117567669	PRJEB83897	45	M	Viken	East	Sekret	Urethra	2022-09-12	0,032	0,016	0,004	1	0,25	32	4	8	Negativ	1893	Other	16	f7295caed16	NOT_FOUND	NOT_FOUND	NOT_FOUND	RESISTANT	INTERMEDIATE	RESISTANT
538150.fasta	538150	SAMEA5608306	PRJEB32435	18	M	Oslo	East	Ikke angitt		2016-06-22	0,125	0,016	0,004	2	0,25	16	32	NA	NA	1893	Other	16	1054	NOT_FOUND	NOT_FOUND	NOT_FOUND	RESISTANT	INTERMEDIATE	RESISTANT
552145.fasta	552145	SAMEA5608361	PRJEB32435	20	M	Viken	East	Sekret	Urethra	2016-11-01	0,032	0,016	0,002	4	0,125	16	8	NA	NA	1893	Other	16	1205	NOT_FOUND	NOT_FOUND	NOT_FOUND	RESISTANT	INTERMEDIATE	RESISTANT
564717.fasta	564717	SAMEA5608628	PRJEB32435	17	F	Oslo	East	Sekret	Cervix uter	2017-01-27	0,125	0,016	0,004	4	0,25	16	16	NA	NA	1893	Other	16	1054	NOT_FOUND	NOT_FOUND	NOT_FOUND	RESISTANT	INTERMEDIATE	RESISTANT
564719.fasta	564719	SAMEA5608629	PRJEB32435	16	M	Oslo	East	Sekret	Urethra	2017-01-26	0,125	0,016	0,004	2	0,25	16	32	NA	NA	1893	Other	16	1054	NOT_FOUND	NOT_FOUND	NOT_FOUND	RESISTANT	INTERMEDIATE	RESISTANT
626009.fasta	626009	SAMEA6533545	PRJEB32435	42	M	NA	NA	SEKRET	URETRA	2018-04-12	NA	NA	NA	NA	NA	NA	NA	NA	NA	14113	Other	16	e3481a50d3c	NOT_FOUND	NOT_FOUND	NOT_FOUND	RESISTANT	INTERMEDIATE	RESISTANT
552872.fasta	552872	SAMEA5608395	PRJEB32435	23	M	Vestland	West	Sekret	Anus	2016-03-08	0,25	0,016	0,008	0,004	0,5	16	16	NA	NA	10932	Other	36	414	NOT_FOUND	NOT_FOUND	NOT_FOUND	NOT_FOUND	INTERMEDIATE	RESISTANT
640322.fasta	640322	SAMEA6533838	PRJEB32435	32	M	NA	NA	SEKRET	URETRA	2018-09-14	0,19	0,016	0,016	4	NA	NA	NA	NA	NA	8392	Other	34	3714	NOT_FOUND	NOT_FOUND	NOT_FOUND	RESISTANT	NOT_FOUND	NOT_FOUND
542364.fasta	542364	SAMEA5608326	PRJEB32435	45	M	Vestfold og Telemark	East	Sekret	Urethra	2016-08-11	0,125	0,016	0,008	2	8	16	32	NA	NA	14337	5	33	719	NOT_FOUND	NOT_FOUND	NOT_FOUND	RESISTANT	RESISTANT	RESISTANT
597652.fasta	597652	SAMEA5609034	PRJEB32435	32	M	Innlandet	East	Sekret	Urethra	2017-09-05	0,064	0,016	0,002	2	32	8	8	NA	NA	1597	9	27	2670	NOT_FOUND	NOT_FOUND	NOT_FOUND	RESISTANT	RESISTANT	RESISTANT
598593.fasta	598593	SAMEA5609042	PRJEB32435	32	M	Innlandet	East	Sekret	Urethra	2017-09-11	0,032	0,016	0,004	2	32	8	16	NA	NA	1597	9	27	2670	NOT_FOUND	NOT_FOUND	NOT_FOUND	RESISTANT	RESISTANT	RESISTANT
575779.fasta	575779	SAMEA5608787	PRJEB32435	30	M	Oslo	East	Sekret	Anus	2017-04-25	0,25	0,032	0,016	32	8	16	64	NA	NA	1588	8	31	2646	NOT_FOUND	NOT_FOUND	NOT_FOUND	RESISTANT	RESISTANT	RESISTANT
610748.fasta	610748	SAMEA5609167	PRJEB32435	53	M	NA	NA	Sekret	Urethra	2017-12-21	0,064	0,016	0,002	4	8	16	16	NA	NA	8156	Other	35	f686c0af5120	NOT_FOUND	NOT_FOUND	NOT_FOUND	RESISTANT	RESISTANT	RESISTANT
539460.fasta	539460	SAMEA5608313	PRJEB32435	70	M	Vestland	West	Sekret		2016-01-06	0,125	0,016	0,004	32	4	16	32	NA	NA	1579	Other	40	2589	NOT_FOUND	NOT_FOUND	NOT_FOUND	RESISTANT	RESISTANT	RESISTANT
21GB000732.fasta	21GB000732	SAMEA117567134	PRJEB83897	28	M	Vestland	West	Sekret	Urethra	2021-11-17	0,125	0,016	0,016	32	1	16	16	NA	Påvist	1588	8	31	d3af71b55d0	NOT_FOUND	NOT_FOUND	NOT_FOUND	RESISTANT	RESISTANT	RESISTANT
21GB000754.fasta	21GB000754	SAMEA117567139	PRJEB83897	38	M	Oslo	East	Sekret	Urethra	2021-11-22	0,125	0,016	0,002	4	1	16	8	NA	Påvist	1588	8	31	d3af71b55d0	NOT_FOUND	NOT_FOUND	NOT_FOUND	RESISTANT	RESISTANT	RESISTANT
23GB001022.fasta	23GB001022	SAMEA117568427	PRJEB83897	61	F	Oslo	East	Sekret	Cervix uter	2023-04-28	0,064	0,016	0,002	1	16	32	16	NA	Positiv	11365	8	31	c3e2957cd5f4	NOT_FOUND	NOT_FOUND	NOT_FOUND	RESISTANT	RESISTANT	RESISTANT
574853.fasta	574853	SAMEA5608780	PRJEB32435	26	M	Oslo	East	Sekret	Urethra	2017-04-20	0,25	0,032	0,016	1	2	16	16	NA	NA	1583	12	24	539	NOT_FOUND	NOT_FOUND	NOT_FOUND	RESISTANT	RESISTANT	RESISTANT
553619.fasta	553619	SAMEA5608415	PRJEB32435	34	M	Oslo	East	Sekret	Urethra	2016-06-14	0,032	0,016	0,004	4	1	8	16	NA	NA	7827	8	31	2600	NOT_FOUND	NOT_FOUND	NOT_FOUND	RESISTANT	RESISTANT	RESISTANT
553637.fasta	553637	SAMEA5608420	PRJEB32435	28	F	Oslo	East	Sekret	Cervix uter	2016-06-29	0,032	0,016	0,004	2	2	8	8	NA	NA	7827	8	31	2600	NOT_FOUND	NOT_FOUND	NOT_FOUND	RESISTANT	RESISTANT	RESISTANT
536928.fasta	536928	SAMEA5608299	PRJEB32435	51	M	Vestland	West	Sekret	Urethra	2016-06-10	0,25	0,016	0,008	1	32	16	32	NA	NA	7823	12	24	2590	NOT_FOUND	NOT_FOUND	NOT_FOUND	RESISTANT	RESISTANT	RESISTANT
562571.fasta	562571	SAMEA5608594	PRJEB32435	29	F	Trøndelag	North	Sekret	Cervix uter	2017-01-18	0,25	0,016	0,008	8	32	16	32	NA	NA	8123	9	27	625	NOT_FOUND	NOT_FOUND	NOT_FOUND	RESISTANT	RESISTANT	RESISTANT
20GB001387.fasta	20GB001387	SAMEA117566976	PRJEB83897	28	M	Troms og Finnmark	North	Sekret	Urethra	2020-11-26	0,125	0,016	0,016	8	32	16	32	NA	Påvist	1903	Other	26	6bc19ccb27d	NOT_FOUND	NOT_FOUND	NOT_FOUND	RESISTANT	RESISTANT	RESISTANT
21GB000132.fasta	21GB000132	SAMEA117567009	PRJEB83897	20	M	Oslo	East	Sekret	Urethra	2021-03-15	0,125	0,016	0,016	8	16	16	32	NA	Påvist	1903	Other	26	467a2be2cfa1	NOT_FOUND	NOT_FOUND	NOT_FOUND	RESISTANT	RESISTANT	RESISTANT
637108.fasta	637108	SAMEA6533770	PRJEB32435	58	M	NA	NA	SEKRET	URETRA	2018-08-24	NA	NA	NA	NA	NA	NA	NA	NA	NA	1903	Other	26	5b2121a325c	NOT_FOUND	NOT_FOUND	NOT_FOUND	RESISTANT	RESISTANT	RESISTANT
666561.fasta	666561	SAMEA6534316	PRJEB32435	18	M	Rogaland	West	Sekret	Urethra	2019-06-20	0,25	0,016	0,004	1	16	16	16	4	Påvist	1583	5	33	f31b903e4db	NOT_FOUND	NOT_FOUND	NOT_FOUND	RESISTANT	RESISTANT	RESISTANT
666563.fasta	666563	SAMEA6534317	PRJEB32435	18	F	Rogaland	West	Sekret	Urethra	2019-06-19	0,5	0,016	0,008	2	8	16	32	4	Påvist	1583	5	33	f31b903e4db	NOT_FOUND	NOT_FOUND	NOT_FOUND	RESISTANT	RESISTANT	RESISTANT
577543.fasta	577543	SAMEA5608816	PRJEB32435	44	F	Oslo	East	Sekret	Cervix uter	2017-05-12	0,125	0,016	0,002	2	8	8	16	NA	NA	13943	Other	26	1879	NOT_FOUND	NOT_FOUND	NOT_FOUND	RESISTANT	RESISTANT	RESISTANT
535164.fasta	535164	SAMEA5608288	PRJEB32435	32	F	Innlandet	East	Sekret	Vagina	2016-05-25	0,125	0,016	0,016	2	32	16	32	NA	NA	1925	9	27	2582	NOT_FOUND	NOT_FOUND	NOT_FOUND	RESISTANT	RESISTANT	NOT_FOUND
553821.fasta	553821	SAMEA5608439	PRJEB32435	33	M	Oslo	East	Sekret	Urethra	2016-05-06	0,25	0,016	0,008	4	32	16	16	NA	NA	1925	9	27	2582	NOT_FOUND	NOT_FOUND	NOT_FOUND	RESISTANT	RESISTANT	NOT_FOUND
591881.fasta	591881	SAMEA5608891	PRJEB32435	58	M	Vestland	West	Sekret	Urethra	2017-07-05	0,125	0,016	0,004	8	32	16	32	NA	NA	1925	9	27	2649	NOT_FOUND	NOT_FOUND	NOT_FOUND	RESISTANT	RESISTANT	NOT_FOUND
592330.fasta	592330	SAMEA5608898	PRJEB32435	58	M	Vestland	West	Sekret	Urethra	2017-07-13	0,125	0,016	0,002	4	32	16	8	NA	NA	1925	9	27	2649	NOT_FOUND	NOT_FOUND	NOT_FOUND	RESISTANT	RESISTANT	NOT_FOUND
594683.fasta	594683	SAMEA5608981	PRJEB32435	42	M	Vestland	West	Sekret	Urethra	2017-08-04	0,125	0,016	0,002	4	8	16	16	NA	NA	1925	9	27	2649	NOT_FOUND	NOT_FOUND	NOT_FOUND	RESISTANT	RESISTANT	NOT_FOUND
21GB000717.fasta	21GB000717	SAMEA117567129	PRJEB83897	38	M	Oslo	East	Sekret	Urethra	2021-11-08	0,125	0,016	0,016	32	1	16	16	NA	Påvist	1588	8	31	d3af71b55d0	NOT_FOUND	NOT_FOUND	NOT_FOUND	RESISTANT	RESISTANT	RESISTANT
561473.fasta	561473	SAMEA5608580	PRJEB32435	33	M	Vestland	West	Sekret	Urethra	2017-01-10	0,25	0,016	0,008	4	32	8	0,5	NA	NA	8143	9	27	2614	NOT_FOUND	NOT_FOUND	NOT_FOUND	RESISTANT	RESISTANT	RESISTANT
580685.fasta	580685	SAMEA5608860	PRJEB32435	58	M	Oslo	East	Sekret	Urethra	2017-06-12	0,125	0,016	0,016	8	8	16	1	NA	NA	1588	8	31	871	NOT_FOUND	NOT_FOUND	NOT_FOUND	RESISTANT	RESISTANT	NOT_FOUND
652600.fasta	652600	SAMEA6534052	PRJEB32435	26	M	Viken	East	Sekret	Urethra	2019-02-01	0,5	0,016	0,016	0,5	1	16	1	8	Ikke påvist	10314	2	10	2730	NOT_FOUND	NOT_FOUND	NOT_FOUND	RESISTANT	RESISTANT	RESISTANT
666682.fasta	666682	SAMEA6534325	PRJEB32435	41	M	Oslo	East	Sekret	Urethra	2019-06-19	0,5	0,016	0,016	0,5	2	16	0,5	8	Ikke påvist	10314	2	10	2730	NOT_FOUND	NOT_FOUND	NOT_FOUND	RESISTANT	RESISTANT	RESISTANT
667169.fasta	667169	SAMEA6534338	PRJEB32435	21	M	Oslo	East	Sekret	Urethra	2019-06-27	0,5	0,016	0,032	16	4	16	1	8	Ikke påvist	10314	2	10	2730	NOT_FOUND	NOT_FOUND	NOT_FOUND	RESISTANT	RESISTANT	RESISTANT
672391.fasta	672391	SAMEA6534589	PRJEB32435	24	M	Oslo	East	Sekret	Anus	2019-09-13	0,5	0,016	0,016	1	2	16	1	8	Ikke påvist	10314	2	10	2730	NOT_FOUND	NOT_FOUND	NOT_FOUND	RESISTANT	RESISTANT	RESISTANT
20GB000246.fasta	20GB000246	SAMEA117566590	PRJEB83897	59	M	Vestland	West	Sekret	Anus	2020-01-14	0,5	0,032	0,032	4	0,5	16	0,5	8	Ikke påvist	14564	2	10	3636	NOT_FOUND	NOT_FOUND	NOT_FOUND	RESISTANT	INTERMEDIATE	RESISTANT
20GB000618.fasta	20GB000618	SAMEA117566680	PRJEB83897	38	M	Oslo	East	Sekret	Urethra	2020-02-17	0,25	0,016	0,008	2	0,25	8	0,25	NA	Ikke påvist	10314	2	10	1615	NOT_FOUND	NOT_FOUND	NOT_FOUND	RESISTANT	INTERMEDIATE	RESISTANT
20GB000518.fasta	20GB000518	SAMEA117566649	PRJEB83897	27	M	Oslo	East	Sekret	Hals	2020-02-10	0,25	0,032	0,032	16	2	16	2	NA	Ikke påvist	10314	2	10	1816	NOT_FOUND	NOT_FOUND	NOT_FOUND	RESISTANT	RESISTANT	RESISTANT
21GB000647.fasta	21GB000647	SAMEA117567115	PRJEB83897	31	M	Rogaland	West	Sekret	Anus	2021-10-25	0,5	0,032	0,032	32	1	16	2	NA	Ikke påvist	7822	2	10	1816	NOT_FOUND	NOT_FOUND	NOT_FOUND	RESISTANT	RESISTANT	RESISTANT
592026.fasta	592026	SAMEA5608896	PRJEB32435	39	M	Viken	East	Sekret	Hals	2017-07-03	0,5	0,016	0,032	32	2	16	4	NA	NA	10314	2	10	1816	NOT_FOUND	NOT_FOUND	NOT_FOUND	RESISTANT	RESISTANT	RESISTANT
595714.fasta	595714	SAMEA5608996	PRJEB32435	26	M	Oslo	East	Sekret	Urethra	2017-08-10	0,5	0,016	0,016	32	1	16	2	NA	NA	10314	2	10	1816	NOT_FOUND	NOT_FOUND	NOT_FOUND	RESISTANT	RESISTANT	RESISTANT

[illegible]

20GB001131.fasta	20GB001131	SAMEA117566853	PRJEB83897	39	F	Oslo	East	Sekret	Cervix uter	2020-08-03	0,125	0,032	0,016	0,5	0,125	16	16	NA	Ikke påvist	1599	12	24	1340	[illegible]
20GB001133.fasta	20GB001133	SAMEA117566854	PRJEB83897	60	M	Oslo	East	Sekret	Urethra	2020-08-03	0,064	0,032	0,008	0,5	0,125	16	8	NA	Ikke påvist	1599	12	24	1340	[illegible]
20GB001140.fasta	20GB001140	SAMEA117566857	PRJEB83897	18	M	Viken	East	Sekret	Anus	2020-08-06	0,064	0,032	0,008	0,5	0,125	8	4	NA	Ikke påvist	1599	12	24	1340	[illegible]
20GB001160.fasta	20GB001160	SAMEA117566866	PRJEB83897	39	M	Oslo	East	Sekret	Anus	2020-08-17	0,125	0,016	0,008	0,25	0,125	8	8	NA	Ikke påvist	1599	12	24	1340	[illegible]
20GB001168.fasta	20GB001168	SAMEA117566871	PRJEB83897	27	M	Oslo	East	Sekret	Anus	2020-08-19	0,064	0,016	0,008	1	0,125	16	8	NA	Ikke påvist	1583	12	24	1340	[illegible]
20GB001172.fasta	20GB001172	SAMEA117566875	PRJEB83897	49	M	Oslo	East	Sekret	Hals	2020-08-20	0,064	0,016	0,008	0,5	0,125	8	8	NA	Ikke påvist	1599	12	24	1340	[illegible]
20GB001189.fasta	20GB001189	SAMEA117566879	PRJEB83897	41	M	Oslo	East	Sekret	Anus	2020-08-24	0,064	0,016	0,008	0,5	0,125	16	8	NA	Ikke påvist	1599	12	24	1340	[illegible]
20GB001218.fasta	20GB001218	SAMEA117566895	PRJEB83897	28	M	Oslo	East	Sekret	Anus	2020-09-07	0,125	0,016	0,008	0,25	0,125	16	8	NA	Ikke påvist	1599	12	24	1340	[illegible]
22GB000454.fasta	22GB000454	SAMEA117567337	PRJEB83897	32	M	Oslo	East	Sekret	Anus	2022-04-29	0,125	0,016	0,008	0,125	0,125	8	8	4	Negativ	7365	12	24	539	[illegible]
22GB001693.fasta	22GB001693	SAMEA117567948	PRJEB83897	21	M	Vestland	West	Sekret	Anus	2022-12-08	0,125	0,016	0,016	2	0,125	8	8	4	Negativ	1583	12	24	539	[illegible]
23GB000481.fasta	23GB000481	SAMEA117568185	PRJEB83897	31	M	Oslo	East	Sekret	Urethra	2023-02-18	0,125	0,016	0,008	0,25	1	32	8	NA	Positiv	1583	12	24	1340	[illegible]
612676.fasta	612676	SAMEA6533436	PRJEB32435	25	M	Møre og Romsdal	West	Sekret	Anus	2018-01-11	0,25	0,016	0,008	1	2	16	32	NA	NA	1583	12	24	539	[illegible]
656361.fasta	656361	SAMEA6534109	PRJEB32435	23	F	Oslo	East	Sekret	Cervix uter	2019-03-01	0,25	0,032	0,008	0,25	32	16	8	4	Ikke påvist	1583	12	24	1340	[illegible]
658107.fasta	658107	SAMEA6534134	PRJEB32435	39	M	Oslo	East	Sekret	Urethra	2019-03-20	0,125	0,016	0,016	0,5	0,5	32	16	NA	NA	1583	12	24	1340	[illegible]
659715.fasta	659715	SAMEA6534159	PRJEB32435	29	M	Viken	East	Sekret	Urethra	2019-04-02	0,5	0,032	0,008	1	0,125	16	16	Ikke utført	Ikke påvist	1583	12	24	1340	[illegible]
660428.fasta	660428	SAMEA6534166	PRJEB32435	31	M	Oslo	East	Sekret	Urethra	2019-04-08	0,5	0,032	0,008	0,5	0,125	16	16	Ikke utført	Ikke påvist	1583	12	24	1340	[illegible]
662310.fasta	662310	SAMEA6534200	PRJEB32435	41	M	Oslo	East	Sekret	Anus	2019-04-29	0,25	0,016	0,004	0,5	0,125	16	16	4	Ikke påvist	1583	12	24	1340	[illegible]
664775.fasta	664775	SAMEA6534280	PRJEB32435	25	M	Viken	East	Sekret	Urethra	2019-05-27	0,25	0,032	0,004	0,5	0,125	16	16	4	Ikke påvist	1583	12	24	1340	[illegible]
666961.fasta	666961	SAMEA6534332	PRJEB32435	30	M	Oslo	East	Sekret	Anus	2019-06-24	0,25	0,032	0,016	0,5	2	32	16	8	Påvist	1583	12	24	1340	[illegible]
667414.fasta	667414	SAMEA6534349	PRJEB32435	23	M	Oslo	East	Sekret	Urethra	2019-07-01	NA	0,032	0,016	1	0,25	32	32	NA	Ikke påvist	1583	12	24	539	[illegible]
668549.fasta	668549	SAMEA6534471	PRJEB32435	34	M	Oslo	East	Sekret	Anus	2019-07-22	0,25	0,032	0,008	0,5	0,25	16	16	8	Ikke påvist	1583	12	24	1340	[illegible]
668638.fasta	668638	SAMEA6534476	PRJEB32435	34	M	Viken	East	Sekret	Anus	2019-07-25	0,5	0,032	0,008	0,5	0,25	16	16	4	Ikke påvist	1583	12	24	1340	[illegible]
670527.fasta	670527	SAMEA6534528	PRJEB32435	46	M	Viken	East	Sekret	Urethra	2019-08-13	0,25	0,032	0,008	0,5	NA	16	16	4	Påvist	1583	12	24	1340	[illegible]
674271.fasta	674271	SAMEA6534634	PRJEB32435	33	M	Oslo	East	Sekret	Hals	2019-10-07	0,125	0,032	0,008	1	0,125	16	16	4	Ikke påvist	1583	12	24	1340	[illegible]
676263.fasta	676263	SAMEA6534666	PRJEB32435	33	M	Viken	East	Sekret	Urethra	2019-11-01	0,064	0,032	0,016	0,5	0,125	8	16	4	Ikke påvist	1583	12	24	1340	[illegible]
677141.fasta	677141	SAMEA6534688	PRJEB32435	28	M	Oslo	East	Sekret	Anus	2019-11-12	0,125	0,032	0,016	1	0,125	32	16	4	Ikke påvist	1583	12	24	539	[illegible]
20GB000742.fasta	20GB000742	SAMEA117566735	PRJEB83897	39	M	Viken	East	Sekret	Anus	2020-03-20	0,125	0,125	0,016	0,5	0,25	16	32	NA	Ikke påvist	1583	12	24	4536	[illegible]
22GB001691.fasta	22GB001691	SAMEA117567946	PRJEB83897	31	M	Viken	East	Sekret	Urethra	2022-12-07	0,25	0,016	0,016	1	0,5	8	16	8	Negativ	7823	12	24	538daa784db	[illegible]
22GB000039.fasta	22GB000039	SAMEA117567195	PRJEB83897	41	M	Rogaland	West		Urethra	2022-01-06	0,125	0,016	0,016	0,5	0,25	8	16	NA	Ikke påvist	1588	8	31	433	[illegible]
546001.fasta	546001	SAMEA117568445	PRJEB83897	49	M	Møre og Romsdal	West	Sekret	Urethra	2016-09-11	0,125	0,032	0,016	1	0,25	16	32	NA	NA	8776	8	31	2592	[illegible]
636532.fasta	636532	SAMEA6533746	PRJEB32435	32	M	NA	NA	SEKRET	URETRA	2018-08-14	NA	NA	NA	NA	NA	NA	NA	NA	NA	1588	8	31	5435	[illegible]
664485.fasta	664485	SAMEA6534260	PRJEB32435	21	M	Viken	East	Sekret	Hals	2019-05-23	0,25	0,064	0,016	1	0,25	16	32	8	Ikke påvist	1583	12	24	2881	[illegible]
669211.fasta	669211	SAMEA6534498	PRJEB32435	25	M	Oslo	East	Sekret	Anus	2019-08-01	0,25	0,016	0,002	0,5	0,5	16	16	4	Ikke påvist	1583	12	24	2881	[illegible]
680081.fasta	680081	SAMEA117568460	PRJEB83897	59	M	Møre og Romsdal	West	Sekret	Urethra	2019-12-08	0,064	0,016	0,002	0,25	0,125	8	16	4	Ikke påvist	12894	8	31	2638	[illegible]
20GB001006.fasta	20GB001006	SAMEA117566790	PRJEB83897	55	M	Oslo	East	Sekret	Anus	2020-06-11	0,5	0,064	0,004	0,5	0,125	4	8	NA	Ikke påvist	15219	8	31	4535	[illegible]
553599.fasta	553599	SAMEA5608410	PRJEB32435	21	M	Oslo	East	Sekret	Urethra	2016-05-28	0,25	0,064	0,032	32	1	16	256	NA	NA	1588	8	31	1302	[illegible]
553724.fasta	553724	SAMEA5608428	PRJEB32435	23	M	Oslo	East	Sekret	Urethra	2016-04-04	0,25	0,032	0,064	32	1	8	256	NA	NA	1588	8	31	1302	[illegible]
679709.fasta	679709	SAMEA117568457	PRJEB83897	39	M	Vestfold og Telemark	East	Sekret		2019-12-03	0,125	0,016	0,016	2	0,125	4	8	4	Ikke påvist	7822	12	24	1883	[illegible]
607277.fasta	607277	SAMEA6533397	PRJEB32435	38	M	Viken	East	Sekret	Anus	2017-11-27	0,5	0,016	0,008	8	0,125	16	32	NA	NA	7822	12	24	3321	[illegible]
518242.fasta	518242	SAMEA5608225	PRJEB32435	64	M	Troms og Finnmark	North	Sekret	Urethra	2016-01-22	0,5	0,032	0,008	4	0,125	16	16	NA	NA	7822	12	24	957	[illegible]
526504.fasta	526504	SAMEA5608246	PRJEB32435	25	M	Rogaland	West			2016-03-10	0,25	0,016	0,004	4	0,25	16	16	NA	NA	7822	12	24	416	[illegible]
530060.fasta	530060	SAMEA5608260	PRJEB32435	42	M	Rogaland	West	Sekret	Anus	2016-04-14	0,125	0,016	0,002	4	0,125	8	16	NA	NA	9900	12	24	416	[illegible]
552866.fasta	552866	SAMEA5608393	PRJEB32435	29	M	Oslo	East	Sekret	Urethra	2016-03-10	0,25	0,016	0,008	4	0,5	16	16	NA	NA	7822	12	24	416	[illegible]
603932.fasta	603932	SAMEA5609123	PRJEB32435	35	M	Oslo	East	Sekret	Urethra	2017-10-25	0,5	0,016	0,004	4	0,25	16	32	NA	NA	7822	12	24	71	[illegible]
668215.fasta	668215	SAMEA6534460	PRJEB32435	19	M	Oslo	East	Sekret	Urethra	2019-07-15	0,5	0,016	0,002	2	0,5	8	16	4	Ikke påvist	[illegible]	[illegible]	[illegible]	[illegible]	[illegible]
605803.fasta	605803	SAMEA5609143	PRJEB32435	27	M	Rogaland	West	Sekret	Urethra	2017-11-15	0,125	0,016	0,004	2	0,25	8	32	NA	NA	[illegible]	[illegible]	[illegible]	[illegible]	[illegible]
573718.fasta	573718	SAMEA5608764	PRJEB32435	23	M	Rogaland	West	Sekret	Hals	2017-04-07	0,125	0,016	0,002	2	0,125	16	8	NA	NA	[illegible]	[illegible]	[illegible]	[illegible]	[illegible]
563771.fasta	563771	SAMEA6533369	PRJEB32435	48	M	Oslo	East	Sekret	Urethra	2016-12-23	0,064	0,016	0,004	8	0,25	16	32	NA	NA	[illegible]	[illegible]	[illegible]	[illegible]	[illegible]
608375.fasta	608375	SAMEA6533409	PRJEB32435	45	M	Vestland	West	Sekret	Urethra	2017-12-08	0,064	0,016	0,004	4	0,125	4	8	NA	NA	[illegible]	[illegible]	[illegible]	[illegible]	[illegible]
20GB000335.fasta	20GB000335	SAMEA117566615	PRJEB83897	36	M	Vestfold og Telemark	East	Sekret	Urethra	2020-01-23	0,125	0,016	0,016	2	0,25	8	16	8	Ikke påvist	[illegible]	[illegible]	[illegible]	[illegible]	[illegible]
552837.fasta	552837	SAMEA5608386	PRJEB32435	38	M	Viken	East	Sekret	Urethra	2016-02-29	0,25	0,016	0,004	4	0,25	8	16	NA	NA	[illegible]	[illegible]	[illegible]	[illegible]	[illegible]
23GB001012.fasta	23GB001012	SAMEA117568418	PRJEB83897	44	M	Oslo	East	Sekret	Anus	2023-04-25	0,125	0,032	0,008	0,5	0,25	16	8	NA	Negativ	[illegible]	[illegible]	[illegible]	[illegible]	[illegible]
22GB000883.fasta	22GB000883	SAMEA117567506	PRJEB83897	26	M	Oslo	East	Sekret	Urethra	2022-07-20	0,125	0,016	0,004	0,5	0,25	16	4	8	Negativ	[illegible]	[illegible]	[illegible]	[illegible]	[illegible]
554565.fasta	554565	SAMEA5608451	PRJEB32435	30	M	Oslo	East	Sekret	Urethra	2016-08-09	0,25	0,016	0,008	2	0,25	16	16	NA	NA	[illegible]	[illegible]	[illegible]	[illegible]	[illegible]
20GB000769.fasta	20GB000769	SAMEA117566745	PRJEB83897	67	M	Oslo	East	Sekret	Urethra	2020-04-06	0,5	0,016	0,032	32	2	8	64	NA	Ikke påvist	[illegible]	[illegible]	[illegible]	[illegible]	[illegible]
20GB000165.fasta	20GB000165	SAMEA117566562	PRJEB83897	35	M	Oslo	East	Sekret	Hals	2019-12-16	0,125	0,016	0,008	0,004	0,125	16	16	16	Ikke påvist	[illegible]	[illegible]	[illegible]	[illegible]	[illegible]
560870.fasta	560870	SAMEA5608572	PRJEB32435	21	M	Agder	South	Sekret	Anus	2017-01-04	0,25	0,016	0,004	0,008	0,25	16	64	NA	NA	[illegible]	[illegible]	[illegible]	[illegible]	[illegible]
564102.fasta	564102	SAMEA5608620	PRJEB32435	36	M	Vestfold og Telemark	East	Sekret	Urethra	2017-01-26	0,125	0,016	0,004	0,004	0,125	16	32	NA	NA	[illegible]	[illegible]	[illegible]	[illegible]	[illegible]
571533.fasta	571533	SAMEA5608725	PRJEB32435	26	M	Oslo	East	Sekret	Hals	2017-03-13	0,125	0,016	0,004	0,008	0,25	8	16	NA	NA	[illegible]	[illegible]	[illegible]	[illegible]	[illegible]
571547.fasta	571547	SAMEA5608730	PRJEB32435	36	M	Oslo	East	Sekret	Urethra	2017-03-22	0,125	0,016	0,002	0,004	0,125	8	32	NA	NA	[illegible]	[illegible]	[illegible]	[illegible]	[illegible]
554816.fasta	554816	SAMEA5608486	PRJEB32435	38	M	Viken	East	Sekret	Urethra	2016-08-02	0,25	0,016	0,008	0,004	0,5	16	16	NA	NA	[illegible]	[illegible]	[illegible]	[illegible]	[illegible]
677260.fasta	677260	SAMEA6534695	PRJEB32435	29	M	Oslo	East	Sekret	Urethra	2019-11-12	0,125	0,016	0,016	0,004	0,25	8	16	16	Ikke påvist	[illegible]	[illegible]	[illegible]	[illegible]	[illegible]

[illegible]

666905.fasta	666905	SAMEA6534327	PRJEB32435	29	M	Vestland	West	Sekret	Anus	2019-06-25	0,25	0,016	
673517.fasta	673517	SAMEA6534609	PRJEB32435	23	M	Oslo	East	Sekret	Urethra	2019-09-26	0,064	0,016	
673751.fasta	673751	SAMEA6534624	PRJEB32435	25	M	Viken	East	Sekret	Urethra	2019-09-30	0,125	0,016	
676677.fasta	676677	SAMEA6534677	PRJEB32435	53	M	Vestland	West	Sekret	Anus	2019-11-05	0,25	0,016	
646803.fasta	646803	SAMEA6533976	PRJEB32435	44	M	NA	NA	SEKRET	URETRA	2018-12-03	NA	NA	NA
580458.fasta	580458	SAMEA5608855	PRJEB32435	20	M	Oslo	East	Urin		2017-06-07	0,5	0,125	
22GB000168.fasta	22GB000168	SAMEA117567255	PRJEB83897	34	M	Innlandet	East	Sekret	Urethra	2022-02-18	0,5	0,016	
537175.fasta	537175	SAMEA5608303	PRJEB32435	20	M	Viken	East	Sekret	Anus	2016-06-16	1	0,016	
20GB000460.fasta	20GB000460	SAMEA117566640	PRJEB83897	26	M	Oslo	East	Sekret	Hals	2020-02-06	0,125	0,032	
20GB001091.fasta	20GB001091	SAMEA117566835	PRJEB83897	25	M	Oslo	East	Sekret	Anus	2020-07-20	0,125	0,016	
574506.fasta	574506	SAMEA5608774	PRJEB32435	24	M	Nordland	North	Ikke angitt		2017-01-11	0,25	0,016	
577537.fasta	577537	SAMEA5608815	PRJEB32435	18	F	Nordland	North	Sekret	Rektum	2017-05-09	0,25	0,016	
20GB001386.fasta	20GB001386	SAMEA117566975	PRJEB83897	28	M	Troms og Finnmark	North	Sekret	Hals	2020-11-26	0,25	0,064	
562568.fasta	562568	SAMEA5608593	PRJEB32435	26	M	Trøndelag	North	Sekret	Urethra	2017-01-12	0,25	0,064	
20GB001245.fasta	20GB001245	SAMEA117566909	PRJEB83897	27	F	Trøndelag	North	Sekret	Cervix uter	2020-09-22	1	0,25	
21GB000096.fasta	21GB000096	SAMEA117566999	PRJEB83897	26	F	Trøndelag	North	Sekret	Cervix uter	2021-02-05	1	0,25	
616681.fasta	616681	SAMEA6533457	PRJEB32435	51	F	Oslo	East	Sekret	Cervix uter	2018-02-05	0,25	0,032	
518657.fasta	518657	SAMEA5608230	PRJEB32435	22	F	Innlandet	East	Sekret	Vagina	2016-01-18	0,064	0,032	
555585.fasta	555585	SAMEA5608502	PRJEB32435	43	M	Viken	East	Sekret	Anus	2016-10-11	0,25	0,064	
592586.fasta	592586	SAMEA5608912	PRJEB32435	27	M	Rogaland	West	Sekret	Urethra	2017-07-13	0,25	0,032	
595643.fasta	595643	SAMEA5608991	PRJEB32435	40	M	Oslo	East	Sekret	Urethra	2017-08-07	0,125	0,016	
618493.fasta	618493	SAMEA6533473	PRJEB32435	49	F	Trøndelag	North	Sekret	Vagina	2018-02-13	0,5	0,032	
20GB000173.fasta	20GB000173	SAMEA117566570	PRJEB83897	29	M	Oslo	East	Sekret	Anus	2019-12-18	0,125	0,032	
22GB001462.fasta	22GB001462	SAMEA117567837	PRJEB83897	38	F	Vestland	West	Sekret	Cervix uter	2022-11-04	0,25	0,032	
672945.fasta	672945	SAMEA6534598	PRJEB32435	32	M	Oslo	East	Sekret	Anus	2019-09-16	0,25	0,032	
673513.fasta	673513	SAMEA6534608	PRJEB32435	53	M	Oslo	East	Sekret	Urethra	2019-09-26	0,25	0,064	
674269.fasta	674269	SAMEA6534633	PRJEB32435	37	M	Oslo	East	Sekret	Anus	2019-10-07	0,125	0,032	
539526.fasta	539526	SAMEA5608314	PRJEB32435	18	F	Trøndelag	North	Sekret	Cervix uter	2016-07-11	1	0,032	
20GB000377.fasta	20GB000377	SAMEA117566626	PRJEB83897	22	F	Rogaland	West	Sekret	Vagina	2020-01-29	2	0,125	
547920.fasta	547920	SAMEA5608346	PRJEB32435	22	F	Vestfold og Telemark	East	Sekret	Urethra	2016-09-29	1	0,125	
549760.fasta	549760	SAMEA5608351	PRJEB32435	45	M	Vestfold og Telemark	East	Sekret	Urethra	2016-10-15	1	0,125	
567332.fasta	567332	SAMEA5608676	PRJEB32435	26	M	Oslo	East	Sekret	Hals	2017-02-16	0,5	0,064	
580429.fasta	580429	SAMEA5608854	PRJEB32435	24	F	Vestland	West	Sekret	Cervix uter	2017-06-07	0,064	0,032	
593792.fasta	593792	SAMEA5608956	PRJEB32435	23	F	Vestland	West	Sekret	Vagina	2017-07-28	0,032	0,032	
659762.fasta	659762	SAMEA6534161	PRJEB32435	22	M	Troms og Finnmark	North	Sekret	Urethra	2019-03-28	0,25	0,016	
632121.fasta	632121	SAMEA6533625	PRJEB32435	32	M	NA	NA	SEKRET	URETRA	2018-06-18	NA	NA	NA
532644.fasta	532644	SAMEA5608271	PRJEB32435	49	M	Rogaland	West	Sekret	Anus	2016-05-04	0,125	0,016	
549872.fasta	549872	SAMEA5608352	PRJEB32435	35	F	Oslo	East	Sekret	Cervix uter	2016-10-20	0,5	0,064	
552860.fasta	552860	SAMEA5608392	PRJEB32435	49	F	Viken	East	Sekret	Cervix uter	2016-03-08	0,25	0,032	
554737.fasta	554737	SAMEA5608474	PRJEB32435	32	M	Oslo	East	Sekret	Anus	2016-07-15	0,5	0,032	
555609.fasta	555609	SAMEA5608505	PRJEB32435	35	F	Oslo	East	Sekret	Cervix uter	2016-10-13	0,5	0,032	
555816.fasta	555816	SAMEA5608510	PRJEB32435	34	M	Oslo	East	Sekret	Urethra	2016-08-30	0,5	0,064	
556826.fasta	556826	SAMEA5608529	PRJEB32435	27	M	Oslo	East	Sekret	Urethra	2016-10-24	0,25	0,032	
563752.fasta	563752	SAMEA6533361	PRJEB32435	32	M	Oslo	East	Sekret	Urethra	2016-12-12	0,5	0,064	
564723.fasta	564723	SAMEA5608631	PRJEB32435	31	M	Oslo	East	Sekret	Urethra	2017-01-26	0,5	0,032	
565462.fasta	565462	SAMEA5608647	PRJEB32435	28	M	Oslo	East	Sekret	Urethra	2017-02-06	0,5	0,064	
566763.fasta	566763	SAMEA5608661	PRJEB32435	28	M	Innlandet	East	Sekret	Urethra	2017-02-14	0,5	0,064	
573978.fasta	573978	SAMEA5608770	PRJEB32435	23	M	Oslo	East	Sekret	Urethra	2017-04-11	0,5	0,064	
577230.fasta	577230	SAMEA5608812	PRJEB32435	19	F	Viken	East	Sekret	Cervix uter	2017-05-11	0,5	0,032	

680262.fasta	680262	SAMEA117568462	PRJEB83897	41	M	Oslo	East	Sekret	Urethra	2019-11-05	0,25	0,016	0,002	0,008	0,25	16	0,125		8	Ikke påvist	7359	4	5	1887	NOT_FOUND	NOT_FOUND	NOT_FOUND	NOT_FOUND	NOT_FOUND	NOT_FOUND	NOT_FOUND	NOT_FOUND	mtrR_A39T	0	7
22GB001365.fasta	22GB001365	SAMEA117567778	PRJEB83897	23	F	Oslo	East	Sekret	Vagina	2022-10-19	0,25	0,032	0,016	0,004	0,5	16	1		8	Negativ	11967	Other	26	1845	NOT_FOUND	NOT_FOUND	NOT_FOUND	NOT_FOUND	INTERMEDIATE	NOT_FOUND	NOT_FOUND	RESISTANT	mtrR_A39TmtrR_G45DpenA_ins346DrpsJ_V57M	0	7
580683.fasta	580683	SAMEA5608859	PRJEB32435	24	M	Oslo	East	Sekret	Urethra	2017-06-12	0,125	0,016	0,008	0,004	0,25	16	1	NA		NA	1918	Other	26	1845	NOT_FOUND	NOT_FOUND	NOT_FOUND	NOT_FOUND	INTERMEDIATE	NOT_FOUND	NOT_FOUND	RESISTANT	mtrR_A39TmtrR_G45DpenA_ins346DrpsJ_V57M	0	7
592358.fasta	592358	SAMEA5608902	PRJEB32435	27	M	Oslo	East	Sekret	Urethra	2017-07-10	0,125	0,016	0,008	0,004	0,25	16	1	NA		NA	13413	Other	26	2976	NOT_FOUND	NOT_FOUND	NOT_FOUND	NOT_FOUND	INTERMEDIATE	NOT_FOUND	NOT_FOUND	RESISTANT	mtrR_A39TmtrR_G45DpenA_ins346DrpsJ_V57M	0	7
614999.fasta	614999	SAMEA6533447	PRJEB32435	27	M	Oslo	East	Sekret	Urethra	2018-01-24	0,25	0,016	0,008	0,004	0,25	32	1	NA		NA	13413	Other	26	2976	NOT_FOUND	NOT_FOUND	NOT_FOUND	NOT_FOUND	INTERMEDIATE	NOT_FOUND	NOT_FOUND	RESISTANT	mtrR_A39TmtrR_G45DpenA_ins346DrpsJ_V57M	0	7
22GB000785.fasta	22GB000785	SAMEA117567460	PRJEB83897	34	M	Vestland	West	Sekret	Urethra	2022-06-27	0,25	0,064	0,016	0,004	0,25	8	0,25		4	Negativ	7359	4	5	2171	NOT_FOUND	NOT_FOUND	RESISTANT	NOT_FOUND	INTERMEDIATE	NOT_FOUND	NOT_FOUND	NOT_FOUND	mtrR_A39TpenA_G545SpenA_I312MpenA_V316T	0	7
22GB000786.fasta	22GB000786	SAMEA117567461	PRJEB83897	27	F	Vestland	West	Sekret	Cervix uter	2022-06-28	0,25	0,064	0,016	0,004	0,25	8	0,25		4	Negativ	7359	4	5	2171	NOT_FOUND	NOT_FOUND	RESISTANT	NOT_FOUND	INTERMEDIATE	NOT_FOUND	NOT_FOUND	NOT_FOUND	mtrR_A39TpenA_G545SpenA_I312MpenA_V316T	0	7
554569.fasta	554569	SAMEA5608453	PRJEB32435	25	M	Viken	East	Sekret	Urethra	2016-08-12	0,25	0,064	0,016	0,004	0,125	8	0,25	NA		NA	7359	4	5	2171	NOT_FOUND	NOT_FOUND	RESISTANT	NOT_FOUND	INTERMEDIATE	NOT_FOUND	NOT_FOUND	NOT_FOUND	mtrR_A39TpenA_G545SpenA_I312MpenA_V316T	0	7
555491.fasta	555491	SAMEA5608489	PRJEB32435	24	M	Oslo	East	Sekret	Urethra	2016-09-21	0,25	0,064	0,016	0,008	0,25	16	0,25	NA		NA	7359	4	5	2171	NOT_FOUND	NOT_FOUND	RESISTANT	NOT_FOUND	INTERMEDIATE	NOT_FOUND	NOT_FOUND	NOT_FOUND	mtrR_A39TpenA_G545SpenA_I312MpenA_V316T	0	7
627457.fasta	627457	SAMEA6533564	PRJEB32435	30	F	NA	NA	SEKRET	VAGINA	2018-04-24	0,25	0,064	0,008	0,004	0,25	16	0,125	-		NA	7359	4	5	2171	NOT_FOUND	NOT_FOUND	RESISTANT	NOT_FOUND	INTERMEDIATE	NOT_FOUND	NOT_FOUND	NOT_FOUND	mtrR_A39TpenA_G545SpenA_I312MpenA_V316T	0	7
638508.fasta	638508	SAMEA6533816	PRJEB32435	30	F	NA	NA	SEKRET	URETRA	2018-09-06	0,25	0,094	0,023	0,003	NA	NA	NA	NA		NA	7359	4	5	2171	NOT_FOUND	NOT_FOUND	RESISTANT	NOT_FOUND	INTERMEDIATE	NOT_FOUND	NOT_FOUND	NOT_FOUND	mtrR_A39TpenA_G545SpenA_I312MpenA_V316T	0	7
20GB000080.fasta	20GB000080	SAMEA117566528	PRJEB83897	18	F	Troms og Finnmark	North	Sekret	Cervix uter	2019-12-23	0,5	0,016	0,016	0,008	0,25	8	0,25		8	Ikke påvist	7359	4	5	231	NOT_FOUND	NOT_FOUND	NOT_FOUND	NOT_FOUND	INTERMEDIATE	NOT_FOUND	NOT_FOUND	NOT_FOUND	mtrR_A39TpenA_ins346D	0	7
20GB000096.fasta	20GB000096	SAMEA117566534	PRJEB83897	38	F	Viken	East	Sekret	Cervix uter	2019-12-16	0,25	0,016	0,016	0,004	0,25	8	0,25		8	Ikke påvist	7359	4	5	231	NOT_FOUND	NOT_FOUND	NOT_FOUND	NOT_FOUND	INTERMEDIATE	NOT_FOUND	NOT_FOUND	NOT_FOUND	mtrR_A39TpenA_ins346D	0	7
20GB000109.fasta	20GB000109	SAMEA117566539	PRJEB83897	19	M	Oslo	East	Sekret	Urethra	2020-01-03	0,25	0,016	0,016	0,008	0,25	16	0,25		8	Ikke påvist	7359	4	5	231	NOT_FOUND	NOT_FOUND	NOT_FOUND	NOT_FOUND	INTERMEDIATE	NOT_FOUND	NOT_FOUND	NOT_FOUND	mtrR_A39TpenA_ins346D	0	7
20GB000159.fasta	20GB000159	SAMEA117566556	PRJEB83897	50	M	NA	NA	Sekret	Urethra	2019-12-13	0,25	0,016	0,008	0,008	0,25	8	0,125		4	Ikke påvist	7359	4	5	231	NOT_FOUND	NOT_FOUND	NOT_FOUND	NOT_FOUND	INTERMEDIATE	NOT_FOUND	NOT_FOUND	NOT_FOUND	mtrR_A39TpenA_ins346D	0	7
20GB000189.fasta	20GB000189	SAMEA117566575	PRJEB83897	21	M	Oslo	East	Sekret	Urethra	2020-01-10	0,125	0,016	0,008	0,008	0,25	8	0,125		4	Ikke påvist	7359	4	5	231	NOT_FOUND	NOT_FOUND	NOT_FOUND	NOT_FOUND	INTERMEDIATE	NOT_FOUND	NOT_FOUND	NOT_FOUND	mtrR_A39TpenA_ins346D	0	7
20GB000193.fasta	20GB000193	SAMEA117566576	PRJEB83897	16	F	Rogaland	West	Sekret	Cervix uter	2019-12-11	0,125	0,016	0,016	0,008	0,25	16	0,25		8	Ikke påvist	7359	4	5	231	NOT_FOUND	NOT_FOUND	NOT_FOUND	NOT_FOUND	INTERMEDIATE	NOT_FOUND	NOT_FOUND	NOT_FOUND	mtrR_A39TpenA_ins346D	0	7
20GB000195.fasta	20GB000195	SAMEA117566577	PRJEB83897	26	M	Vestfold og Telemark	East	Sekret		2020-01-09	0,5	0,016	0,016	0,008	0,25	16	0,5		16	Ikke påvist	7359	4	5	231	NOT_FOUND	NOT_FOUND	NOT_FOUND	NOT_FOUND	INTERMEDIATE	NOT_FOUND	NOT_FOUND	NOT_FOUND	mtrR_A39TpenA_ins346D	0	7
20GB000231.fasta	20GB000231	SAMEA117566585	PRJEB83897	19	F	Troms og Finnmark	North	Sekret	Cervix uter	2020-01-10	0,25	0,016	0,016	0,008	0,25	16	0,25		8	Ikke påvist	7359	4	5	231	NOT_FOUND	NOT_FOUND	NOT_FOUND	NOT_FOUND	INTERMEDIATE	NOT_FOUND	NOT_FOUND	NOT_FOUND	mtrR_A39TpenA_ins346D	0	7
20GB000239.fasta	20GB000239	SAMEA117566587	PRJEB83897	35	M	Viken	East	Sekret	Urethra	2020-01-15	0,25	0,016	0,016	0,008	0,25	16	0,25		8	Ikke påvist	7359	4	5	231	NOT_FOUND	NOT_FOUND	NOT_FOUND	NOT_FOUND	INTERMEDIATE	NOT_FOUND	NOT_FOUND	NOT_FOUND	mtrR_A39TpenA_ins346D	0	7
20GB000477.fasta	20GB000477	SAMEA117566647	PRJEB83897	18	M	Oslo	East	Sekret	Urethra	2020-02-03	0,125	0,016	0,016	0,008	0,25	8	0,25		4	Ikke påvist	7359	4	5	231	NOT_FOUND	NOT_FOUND	NOT_FOUND	NOT_FOUND	INTERMEDIATE	NOT_FOUND	NOT_FOUND	NOT_FOUND	mtrR_A39TpenA_ins346D	0	7
20GB000561.fasta	20GB000561	SAMEA117566662	PRJEB83897	25	M	Vestfold og Telemark	East	Sekret	Urethra	2020-02-13	0,25	0,016	0,004	0,008	0,125	8	0,125	NA		Ikke påvist	7359	4	5	231	NOT_FOUND	NOT_FOUND	NOT_FOUND	NOT_FOUND	INTERMEDIATE	NOT_FOUND	NOT_FOUND	NOT_FOUND	mtrR_A39TpenA_ins346D	0	7
20GB000586.fasta	20GB000586	SAMEA117566671	PRJEB83897	42	M	Vestfold og Telemark	East	Sekret	Urethra	2020-02-20	0,25	0,016	0,016	0,004	0,125	8	0,25	NA		Ikke påvist	14102	4	5	58beb17225c	NOT_FOUND	NOT_FOUND	NOT_FOUND	NOT_FOUND	INTERMEDIATE	NOT_FOUND	NOT_FOUND	NOT_FOUND	mtrR_A39TpenA_ins346D	0	7
20GB000632.fasta	20GB000632	SAMEA117566683	PRJEB83897	22	F	Viken	East	Sekret	Cervix uter	2020-02-25	0,125	0,016	0,004	0,008	0,25	8	0,125	NA		Ikke påvist	7359	4	5	231	NOT_FOUND	NOT_FOUND	NOT_FOUND	NOT_FOUND	INTERMEDIATE	NOT_FOUND	NOT_FOUND	NOT_FOUND	mtrR_A39TpenA_ins346D	0	7
20GB000640.fasta	20GB000640	SAMEA117566689	PRJEB83897	17	F	Viken	East	Sekret	Cervix uter	2020-02-26	0,25	0,016	0,016	0,004	0,25	8	0,5	NA		Ikke påvist	7359	4	5	231	NOT_FOUND	NOT_FOUND	NOT_FOUND	NOT_FOUND	INTERMEDIATE	NOT_FOUND	NOT_FOUND	NOT_FOUND	mtrR_A39TpenA_ins346D	0	7
20GB000672.fasta	20GB000672	SAMEA117566698	PRJEB83897	21	M	Viken	East	Sekret	Urethra	2020-03-03	0,25	0,016	0,016	0,008	0,25	16	0,25	NA		Ikke påvist	7359	4	5	231	NOT_FOUND	NOT_FOUND	NOT_FOUND	NOT_FOUND	INTERMEDIATE	NOT_FOUND	NOT_FOUND	NOT_FOUND	mtrR_A39TpenA_ins346D	0	7
20GB000697.fasta	20GB000697	SAMEA117566714	PRJEB83897	46	M	Viken	East	Sekret	Urethra	2020-03-09	0,064	0,016	0,008	0,004	0,125	4	0,064	NA		Ikke påvist	7359	4	5	231	NOT_FOUND	NOT_FOUND	NOT_FOUND	NOT_FOUND	INTERMEDIATE	NOT_FOUND	NOT_FOUND	NOT_FOUND	mtrR_A39TpenA_ins346D	0	7
20GB000796.fasta	20GB000796	SAMEA117566753	PRJEB83897	20	M	Oslo	East	Sekret	Urethra	2020-04-21	0,25	0,016	0,004	0,004	0,125	8	0,125	NA		Ikke påvist	7359	4	5	231	NOT_FOUND	NOT_FOUND	NOT_FOUND	NOT_FOUND	INTERMEDIATE	NOT_FOUND	NOT_FOUND	NOT_FOUND	mtrR_A39TpenA_ins346D	0	7

[illegible]

641192.fasta	641192	SAMEA6533876	PRJEB32435	26	M	NA	NA	SEKRET	ANUS	2018-09-24	0,023	0,016	0,016	0,38	NA	NA	NA	NA	NA	7363	5	33	3628	NOT_FOUND	[illegible]
558652.fasta	558652	SAMEA5608558	PRJEB32435	52	M	Vestland	West	Sekret	Urethra	2016-12-21	0,125	0,016	0,008	2	32	16	32	NA	NA	13333	5	33	2610	NOT_FOUND	[illegible]
21GB000090.fasta	21GB000090	SAMEA117566996	PRJEB83897	33	M	Viken	East	Sekret	Urethra	2021-02-02	0,25	0,016	0,016	4	32	8	32	NA	Påvist	1587	5	33	23ac6850cd8	NOT_FOUND	[illegible]
519014.fasta	519014	SAMEA5608232	PRJEB32435	21	M	Vestfold og Telemark	East	Sekret	Ureter	2016-02-01	0,125	0,016	0,008	2	32	16	32	NA	NA	1587	5	33	1204	NOT_FOUND	[illegible]
606371.fasta	606371	SAMEA6533384	PRJEB32435	32	M	Vestfold og Telemark	East	Sekret	Urethra	2017-11-20	0,125	0,016	0,008	4	32	16	32	NA	NA	1587	5	33	1204	NOT_FOUND	[illegible]
671404.fasta	671404	SAMEA6534565	PRJEB32435	30	M	Rogaland	West	Sekret	Urethra	2019-08-28	0,125	0,016	0,016	4	32	16	32	4	Påvist	1587	5	33	1204	NOT_FOUND	[illegible]
673614.fasta	673614	SAMEA6534621	PRJEB32435	34	M	Trøndelag	North	Sekret	Urethra	2019-09-24	0,125	0,016	0,016	4	32	8	32	4	Påvist	1587	5	33	1204	NOT_FOUND	[illegible]
673914.fasta	673914	SAMEA6534627	PRJEB32435	22	F	Troms og Finnmark	North	Sekret	Cervix uter	2019-09-26	0,125	0,016	0,016	4	32	16	32	8	Påvist	1587	5	33	1204	NOT_FOUND	[illegible]
20GB000160.fasta	20GB000160	SAMEA117566557	PRJEB83897	29	M	Oslo	East	Sekret	Cervix uter	2019-12-13	0,064	0,016	0,004	1	32	16	4	4	Påvist	1587	5	33	719	NOT_FOUND	[illegible]
20GB000616.fasta	20GB000616	SAMEA117566679	PRJEB83897	30	M	Viken	East	Sekret	Hals	2020-02-17	0,064	0,016	0,004	0,5	2	16	4	NA	Påvist	1587	5	33	3596	NOT_FOUND	[illegible]
20GB000757.fasta	20GB000757	SAMEA117566739	PRJEB83897	45	M	Vestland	West	Sekret	Urethra	2020-03-23	0,125	0,016	0,016	2	8	16	32	NA	Påvist	1587	5	33	719	NOT_FOUND	[illegible]
20GB000798.fasta	20GB000798	SAMEA117566755	PRJEB83897	40	M	Agder	South	Sekret	Urethra	2020-02-03	0,064	0,016	0,016	1	8	16	16	NA	Påvist	1587	5	33	1203	NOT_FOUND	[illegible]
21GB000125.fasta	21GB000125	SAMEA117567007	PRJEB83897	25	M	Oslo	East	Sekret	Anus	2021-03-09	0,125	0,016	0,004	1	16	8	8	NA	Påvist	7363	5	33	719	NOT_FOUND	[illegible]
21GB000306.fasta	21GB000306	SAMEA117567033	PRJEB83897	55	M	Viken	East	Sekret	Urethra	2021-05-27	0,125	0,016	0,016	1	2	16	16	NA	Påvist	7363	5	33	1203	NOT_FOUND	[illegible]
22GB000423.fasta	22GB000423	SAMEA117567327	PRJEB83897	26	F	Vestfold og Telemark	East	Sekret	Vagina	2022-04-22	0,125	0,016	0,016	1	32	16	32	4	Positiv	1587	5	33	1139	NOT_FOUND	[illegible]
22GB000962.fasta	22GB000962	SAMEA117567551	PRJEB83897	21	M	Møre og Romsdal	West	Sekret	Urethra	2022-08-08	0,064	0,016	0,016	0,5	32	8	16	4	Positiv	7363	5	33	719	NOT_FOUND	[illegible]
22GB001341.fasta	22GB001341	SAMEA117567770	PRJEB83897	23	M	Viken	East	Sekret	Urethra	2022-10-17	0,064	0,016	0,016	1	32	16	16	8	Positiv	7363	5	33	42208cba9f2	NOT_FOUND	[illegible]
22GB001424.fasta	22GB001424	SAMEA117567818	PRJEB83897	33	M	Oslo	East	Sekret	Urethra	2022-10-26	0,125	0,016	0,004	0,25	4	16	2	4	Positiv	1587	5	33	719	NOT_FOUND	[illegible]
23GB000821.fasta	23GB000821	SAMEA117568336	PRJEB83897	34	M	Troms og Finnmark	North	Sekret	Urethra	2023-03-31	0,064	0,016	0,016	1	4	8	32	NA	Positiv	7363	5	33	719	NOT_FOUND	[illegible]
23GB000893.fasta	23GB000893	SAMEA117568366	PRJEB83897	43	M	Vestland	West	Sekret	Urethra	2023-04-14	0,125	0,016	0,016	2	32	16	16	NA	Positiv	1587	5	33	719	NOT_FOUND	[illegible]
531664.fasta	531664	SAMEA5608267	PRJEB32435	24	M	Innlandet	East	Sekret	Urethra	2016-04-22	0,064	0,016	0,008	2	32	16	16	NA	NA	1587	5	33	714	NOT_FOUND	[illegible]
534323.fasta	534323	SAMEA5608285	PRJEB32435	36	M	Innlandet	East	Sekret	Urethra	2016-05-18	0,125	0,016	0,008	2	16	16	32	NA	NA	1587	5	33	719	NOT_FOUND	[illegible]
541209.fasta	541209	SAMEA117568440	PRJEB83897	23	M	Møre og Romsdal	West	Sekret	Urethra	2016-08-02	0,064	0,016	0,004	2	4	16	16	NA	NA	8134	Other	36	1727	NOT_FOUND	[illegible]
553633.fasta	553633	SAMEA5608419	PRJEB32435	38	M	Oslo	East	Sekret	Urethra	2016-06-23	0,125	0,016	0,004	2	16	16	16	NA	NA	1587	5	33	1139	NOT_FOUND	[illegible]
554563.fasta	554563	SAMEA5608450	PRJEB32435	54	M	Viken	East	Sekret	Urethra	2016-08-09	0,125	0,016	0,008	2	32	16	32	NA	NA	1587	5	33	1139	NOT_FOUND	[illegible]
556843.fasta	556843	SAMEA5608535	PRJEB32435	36	M	Viken	East	Sekret	Urethra	2016-11-03	0,125	0,032	0,008	2	16	16	32	NA	NA	1587	5	33	719	NOT_FOUND	[illegible]
559226.fasta	559226	SAMEA5608561	PRJEB32435	33	M	Troms og Finnmark	North	Sekret	Kjønnsorga	2016-12-27	0,064	0,016	0,008	2	16	16	16	NA	NA	1587	5	33	1661	NOT_FOUND	[illegible]
559699.fasta	559699	SAMEA5608562	PRJEB32435	25	M	Viken	East	Sekret	Urethra	2016-12-29	0,064	0,016	0,008	1	16	16	32	NA	NA	1587	5	33	719	NOT_FOUND	[illegible]
573721.fasta	573721	SAMEA5608765	PRJEB32435	24	M	Rogaland	West	Sekret	Urethra	2017-04-05	0,125	0,016	0,008	2	32	16	16	NA	NA	14347	5	33	1139	NOT_FOUND	[illegible]
574772.fasta	574772	SAMEA5608776	PRJEB32435	69	M	Møre og Romsdal	West	Sekret	Urethra	2017-04-20	0,125	0,016	0,004	1	32	16	16	NA	NA	1587	5	33	eda286018fb	NOT_FOUND	[illegible]
575878.fasta	575878	SAMEA5608794	PRJEB32435	61	M	Viken	East	Sekret	Urethra	2017-04-27	0,064	0,016	0,002	1	8	16	16	NA	NA	1587	5	33	719	NOT_FOUND	[illegible]
577982.fasta	577982	SAMEA5608828	PRJEB32435	35	M	Trøndelag	North	Sekret	Urethra	2017-05-17	0,125	0,016	0,008	1	32	16	16	NA	NA	1587	5	33	1139	NOT_FOUND	[illegible]
594113.fasta	594113	SAMEA5608961	PRJEB32435	31	M	Trøndelag	North	Sekret	Urethra	2017-07-27	0,125	0,016	0,004	2	16	16	32	NA	NA	1587	5	33	719	NOT_FOUND	[illegible]
594119.fasta	594119	SAMEA5608962	PRJEB32435	19	M	Rogaland	West	Sekret	Urethra	2017-07-27	0,064	0,016	0,002	2	16	8	32	NA	NA	7363	5	33	1661	NOT_FOUND	[illegible]
594673.fasta	594673	SAMEA5608976	PRJEB32435	28	F	Trøndelag	North	Sekret	Cervix uter	2017-08-01	0,125	0,016	0,008	2	4	8	16	NA	NA	1587	5	33	719	NOT_FOUND	[illegible]
599767.fasta	599767	SAMEA5609061	PRJEB32435	42	M	Vestfold og Telemark	East	Sekret	Urethra	2017-09-21	0,064	0,016	0,016	2	8	8	32	NA	NA	13333	5	33	719	NOT_FOUND	[illegible]
601071.fasta	601071	SAMEA5609083	PRJEB32435	22	M	Oslo	East	Sekret	Urethra	2017-10-05	0,125	0,016	0,004	4	32	8	16	NA	NA	1587	5	33	719	NOT_FOUND	[illegible]
601806.fasta	601806	SAMEA5609096	PRJEB32435	72	M	Trøndelag	North	Sekret	Urethra	2017-10-12	0,064	0,016	0,004	2	8	8	8	NA	NA	1587	5	33	1139	NOT_FOUND	[illegible]
601853.fasta	601853	SAMEA5609098	PRJEB32435	30	M	Viken	East	Sekret	Urethra	2017-10-12	0,125	0,016	0,004	1	4	16	16	NA	NA	13333	5	33	719	NOT_FOUND	[illegible]
601963.fasta	601963	SAMEA5609101	PRJEB32435	30	M	Viken	East	Sekret	Urethra	2017-10-13	0,125	0,016	0,008	1	8	16	16	NA	NA	13333	5	33	719	NOT_FOUND	[illegible]
612552.fasta	612552	SAMEA6533431	PRJEB32435	56	M	Rogaland	West	Sekret	Urethra	2018-01-04	0,125	0,016	0,004	2	8	16	16	NA	NA	1587	5	33	1139	NOT_FOUND	[illegible]
620090.fasta	620090	SAMEA6963026	PRJEB32435	53	M	NA	NA	SEKRET	URETRA	2018-02-27	NA	NA	NA	NA	NA	NA	NA	NA	NA	1587	5	33	1139	NOT_FOUND	[illegible]
621124.fasta	621124	SAMEA6533485	PRJEB32435	51	F	NA	NA	SEKRET	VAGINA	2018-03-07	NA	NA	NA	NA	NA	NA	NA	NA	NA	1587	5	33	719	NOT_FOUND	[illegible]
628085.fasta	628085	SAMEA5609219	PRJEB32435	49	M	NA	NA	SEKRET	URETRA	2018-04-30	0,125	0,016	0,002	1	32	16	8	+	NA	1587	5	33	1661	NOT_FOUND	[illegible]
634017.fasta	634017	SAMEA6533677	PRJEB32435	53	M	NA	NA	SEKRET	FORHUD	2018-07-12	NA	NA	NA	NA	NA	NA	NA	NA	NA	1587	5	33	1661	NOT_FOUND	[illegible]
635329.fasta	635329	SAMEA6533718	PRJEB32435	21	F	NA	NA	SEKRET	VAGINA	2018-08-01	NA	NA	NA	NA	NA	NA	NA	NA	NA	1587	5	33	1661	NOT_FOUND	[illegible]
640715.fasta	640715	SAMEA6533855	PRJEB32435	41	M	NA	NA	SEKRET	URETRA	2018-10-04	0,032	0,016	0,016	0,75	NA	NA	NA	NA	NA	13474	5	33	17b66eb7841	NOT_FOUND	[illegible]
641165.fasta	641165	SAMEA6533868	PRJEB32435	50	M	NA	NA	SEKRET	URETRA	2018-10-01	0,094	0,016	0,016	1	NA	NA	NA	NA	NA	1587	5	33	1139	NOT_FOUND	[illegible]
642409.fasta	642409	SAMEA6533899	PRJEB32435	38	M	NA	NA	SEKRET	URETRA	2018-10-23	0,016	0,016	0,016	1,5	NA	NA	NA	NA	NA	1587	5	33	1203	NOT_FOUND	[illegible]
651677.fasta	651677	SAMEA6534041	PRJEB32435	33	M	Nordland	North	Sekret	Urethra	2019-01-18	0,064	0,016	0,004	0,5	2	16	8	NA	Påvist	13333	5	33	1203	NOT_FOUND	[illegible]
655077.fasta	655077	SAMEA6534087	PRJEB32435	68	M	Viken	East	Sekret	Urethra	2019-02-20	0,064	0,016	0,004	1	16	16	8	Ikke utført	Påvist	14370	5	33	719	NOT_FOUND	[illegible]
658115.fasta	658115	SAMEA6534135	PRJEB32435	66	M	Agder	South	Sekret	Urethra	2019-03-19	0,125	0,016	0,004	2	32	16	16	NA	NA	1587	5	33	1139	NOT_FOUND	[illegible]
663557.fasta	663557	SAMEA6534237	PRJEB32435	28	M	Oslo	East	Sekret	Urethra	2019-05-13	0,125	0,016	0,002	1	32	16	16	4	Påvist	1587	5	33	719	NOT_FOUND	[illegible]
664985.fasta	664985	SAMEA6534283	PRJEB32435	39	M	Nordland	North	Sekret	Urethra	2019-05-28	0,125	0,016	0,008	1	32	16	8	4	Påvist	13333	5	33	714	NOT_FOUND	[illegible]
665366.fasta	665366	SAMEA6534292	PRJEB32435	23	M	Oslo	East	Sekret	Urethra	2019-06-03	0,25	0,016	0,004	1	32	16	8	4	Påvist	1587	5	33	719	NOT_FOUND	[illegible]
667743.fasta	667743	SAMEA6534436	PRJEB32435	20	F	Oslo	East	Sekret	Cervix uter	2019-07-08	0,125	0,016	0,004	1	32	16	16	4	Påvist	1587	5	33	719	NOT_FOUND	[illegible]
668205.fasta	668205	SAMEA6534455	PRJEB32435	31	M	Trøndelag	North	Sekret	Hals	2019-07-19	2	0,016	0,004	32	32	16	1	4	Påvist	1587	5	33	719	NOT_FOUND	[illegible]
668415.fasta	668415	SAMEA6534467	PRJEB32435	26	M	Vestfold og Telemark	East	Sekret	Urethra	2019-07-19	0,25	0,016	0,002	1	32	16	16	4	Påvist	1587	5	33	719	NOT_FOUND	[illegible]
668551.fasta	668551	SAMEA6534472	PRJEB32435	22	F	Oslo	East	Sekret	Cervix uter	2019-07-22	0,125	0,016	0,004	1	16	16	8	4	Påvist	1587	5	33	719	NOT_FOUND	[illegible]
668968.fasta	668968	SAMEA6534485	PRJEB32435	32	M	Viken	East	Sekret	Urethra	2019-07-30	0,125	0,016	0,002	2	16	16	16	4	Påvist	1587	5	33	719	NOT_FOUND	[illegible]
669104.fasta	669104	SAMEA6534490	PRJEB32435	33	M	Rogaland	West	Sekret	Urethra	2019-08-01	0,125	0,016	0,004	2	32	16	16	4	Påvist	1587	5	33	1661	NOT_FOUND	[illegible]
669106.fasta	669106	SAMEA6534491	PRJEB32435	24	M	Vestland	West	Sekret	Urethra	2019-07-29	0,125	0,016	0,004	2	32	16	16	4	Påvist	1587	5	33	719	NOT_FOUND	[illegible]
669372.fasta	669372	SAMEA6534500	PRJEB32435	30	F	Vestfold og Telemark	East	Sekret	Kjønnsorga	2019-08-01	0,064	0,016	0,004	1	16	16	16	4	Påvist	1587	5	33	719	NOT_FOUND	[illegible]
669566.fasta	669566	SAMEA6534503	PRJEB32435	23	M	Viken	East	Ikke angitt		2019-08-06	0,125	0,016	0,008	2	32	32	16	8	Påvist	1587	5	33	719	NOT_FOUND	[illegible]
670474.fasta	670474	SAMEA6534526	PRJEB32435	21	F	Viken	East	Sekret	Vagina	2019-08-15	0,125	0,016	0,008	4	32	16	16	4	Påvist	1587	5	33	719	NOT_FOUND	[illegible]
670668.fasta	670668	SAMEA6534531	PRJEB32435	35	F	Oslo	East	Sekret	Hals	2019-08-20	0,125	0,016	0,008	2	16	16	16	4	Påvist	1587	5	33	719	NOT_FOUND	[illegible]
673962.fasta	673962	SAMEA6534629	PRJEB32435	20	M	Oslo	East	Sekret	Urethra	2019-10-04	0,064	0,016	0,002	1	32	8	4	4	Påvist	1587	5	33	719	NOT_FOUND	[illegible]
23GB000400.fasta	23GB000400	SAMEA117568142	PRJEB83897	33	M	Vestland	West	Sekret	Urethra	2023-02-09	0,064	0,016	0,016	1	8	8	0,5	NA	Positiv	6722	5	33	719	NOT_FOUND	[illegible]
23GB000581.fasta	23GB000581	SAMEA117568233	PRJEB83897	33	M	Vestland	West	Sekret	Urethra	2023-03-02	0,064	0,016	0,016	0,5	8	16	0,5	NA	Positiv	6722	5	33	719	NOT_FOUND	[illegible]
560980.fasta	560980	SAMEA5608575	PRJEB32435	47	M	NA	NA	Sekret	Urethra	2017-01-05	0,125	0,016	0,008	4	16	16	16	NA	NA	-	5	33	1139	NOT_FOUND	[illegible]
22GB001588.fasta	22GB001588	SAMEA117567897	PRJEB83897	54	M	Nordland	North	Sekret	Kjønnsorga	2022-11-22	0,032	0,016	0,016	2	32	8	16	2	Positiv	12982	8	31	1933	NOT_FOUND	[illegible]
23GB000143.fasta	23GB000143	SAMEA117568017	PRJEB83897	38	M	Rogaland	West	Sekret	Urethra	2023-01-05	0,032	0,016	0,016	4	32	16	16	NA	Positiv	1596	8	31	3348	NOT_FOUND	[illegible]
642541.fasta	642541	SAMEA6533910	PRJEB32435	60	M	NA	NA	SEKRET	URETRA	2018-10-23	0,094	0,016	0,016	3	NA	NA	NA	NA	NA	11178	8	31	3676	NOT_FOUND	[illegible]
23GB000361.fasta	23GB000361	SAMEA117568129	PRJEB83897	19	F	Oslo	East	Sekret	Vagina	2023-02-02	0,032	0,016	0,016	2	2	16	16	NA	Positiv	1598	8	31	9057852b682	NOT_FOUND	[illegible]
609602.fasta	609602	SAMEA5609150	PRJEB32435	53	M	Vestland	West	Sekret	Urethra	2017-12-14	0,125	0,016	0,004	8	32	16	32	NA	NA	8776	Other	36	2729	NOT_FOUND	[illegible]
651047.fasta	651047	SAMEA6534032	PRJEB32435	22	M	Rogaland	West	Sekret	Urethra	2019-01-14	0,125	0,016	0,004	4	32	16	32	NA	Påvist	8776	Other	36	2729	NOT_FOUND	[illegible]
20GB000067.fasta	20GB000067	SAMEA117566525	PRJEB83897	27	M	Oslo	East	Sekret	Urethra	2019-12-19	0,125	0,016	0,016	2	32	8	32	8	Påvist	1587	5	33	1658	NOT_FOUND	[illegible]
22GB000056.fasta	22GB000056	SAMEA117567203	PRJEB83897	50	F	Vestland	West	Sekret	Cervix uter	2022-01-06	0,25	0,016	0,016	2	16	8	16	NA	Påvist	1587	5	33	1658	NOT_FOUND	[illegible]
22GB000338.fasta	22GB000338	SAMEA117567306	PRJEB83897	25	M	Vestfold og Telemark	East	Sekret	Urethra	2022-04-01	0,064	0,016	0,004	1	8	16	8	NA	Positiv	1587	5	33	1658	NOT_FOUND	[illegible]
22GB000492.fasta	22GB000492	SAMEA117567349	PRJEB83897	33	M	Oslo	East	Sekret	Urethra	2022-05-04	0,125	0,016	0,016	2	4	16	32	4	Positiv	1587	5	33	1658	NOT_FOUND	[illegible]
22GB000586.fasta	22GB000586	SAMEA117567365	PRJEB83897	36	M	Viken	East	Sekret	Urethra	2022-05-13	0,064	0,016	0,008	0,5	4	16	1	8	Positiv	1587	5	33	1658	NOT_FOUND	[illegible]
22GB000957.fasta	22GB000957	SAMEA117567548	PRJEB83897	44	M	Oslo	East	Sekret	Urethra	2022-08-09	0,25	0,016	0,004	1	8	16	16	8	Positiv	1587	5	33	1658	NOT_FOUND	[illegible]
22GB001521.fasta	22GB001521	SAMEA117567865	PRJEB83897	19	F	Oslo	East	Sekret	Vagina	2022-11-11	0,125	0,016	0,004	1	4	32	8	4	Positiv	13333	5	33	ae9e1860fcbf	NOT_FOUND	[illegible]
22GB001698.fasta	22GB001698	SAMEA117567951	PRJEB83897	19	F	Oslo	East	Sekret	Vagina	2022-12-08	0,125	0,016	0,004	0,5	4	32	8	8	Positiv	13333	5	33	ae9e1860fcbf	NOT_FOUND	[illegible]
23GB000651.fasta	23GB000651	SAMEA117568270	PRJEB83897	34	M	Oslo	East	Sekret	Hals	2023-03-08	0,125	0,016	0,004	1	8	32	8	NA	Positiv	1587	5	33	1658	NOT_FOUND	[illegible]
[illegible]																									

563746.fasta	563746	SAMEA6533358	PRJEB32435	30	M	Oslo	East	Sekret	Anus	2016-12-08	0,25	0,016	0,004	2	0,25	16	0,5	NA	NA	8143	9	27	426	NOT_FOUND	NOT_FOUND	NOT_FOUND	RESISTANT	INTERMEDIATE	RESISTANT	NOT_FOUND	INTERMEDIATE	folP_R228SgyrA_D95AgyrA_S91FmtrR_disruptedparC_D86NpenA_ins346DrpsJ_V57M	0	6
563898.fasta	563898	SAMEA5608617	PRJEB32435	27	M	Agder	South	Sekret	Urethra	2017-01-26	0,5	0,016	0,004	2	0,25	16	0,5	NA	NA	11968	9	27	426	NOT_FOUND	NOT_FOUND	NOT_FOUND	RESISTANT	INTERMEDIATE	RESISTANT	NOT_FOUND	INTERMEDIATE	folP_R228SgyrA_D95AgyrA_S91FmtrR_disruptedparC_D86NpenA_ins346DrpsJ_V57M	0	6
565760.fasta	565760	SAMEA5608653	PRJEB32435	29	M	Agder	South	Sekret	Urethra	2017-02-01	0,5	0,016	0,004	2	0,25	16	0,5	NA	NA	11968	9	27	426	NOT_FOUND	NOT_FOUND	NOT_FOUND	RESISTANT	INTERMEDIATE	RESISTANT	NOT_FOUND	INTERMEDIATE	folP_R228SgyrA_D95AgyrA_S91FmtrR_disruptedparC_D86NpenA_ins346DrpsJ_V57M	0	6
600737.fasta	600737	SAMEA5609076	PRJEB32435	22	F	Vestfold og Telemark	East	Urin		2017-09-28	1	0,016	0,004	2	0,25	16	1	NA	NA	8143	9	27	426	NOT_FOUND	NOT_FOUND	NOT_FOUND	RESISTANT	INTERMEDIATE	RESISTANT	NOT_FOUND	INTERMEDIATE	folP_R228SgyrA_D95AgyrA_S91FmtrR_disruptedparC_D86NpenA_ins346DrpsJ_V57M	0	6
605002.fasta	605002	SAMEA6533380	PRJEB32435	33	M	Oslo	East	Sekret	Urethra	2017-11-03	0,5	0,016	0,004	4	0,25	16	0,5	NA	NA	8143	9	27	426	NOT_FOUND	NOT_FOUND	NOT_FOUND	RESISTANT	INTERMEDIATE	RESISTANT	NOT_FOUND	INTERMEDIATE	folP_R228SgyrA_D95AgyrA_S91FmtrR_disruptedparC_D86NpenA_ins346DrpsJ_V57M	0	6
605206.fasta	605206	SAMEA5609136	PRJEB32435	52	M	Oslo	East	Sekret	Anus	2017-11-06	0,5	0,016	0,002	2	0,25	16	0,5	NA	NA	8143	9	27	426	NOT_FOUND	NOT_FOUND	NOT_FOUND	RESISTANT	INTERMEDIATE	RESISTANT	NOT_FOUND	INTERMEDIATE	folP_R228SgyrA_D95AgyrA_S91FmtrR_disruptedparC_D86NpenA_ins346DrpsJ_V57M	0	6
621071.fasta	621071	SAMEA6533484	PRJEB32435	25	M	NA	NA	SEKRET	ANUS	2018-02-27	0,5	0,016	0,004	1	0,5	16	1	-	NA	8143	9	27	426	NOT_FOUND	NOT_FOUND	NOT_FOUND	RESISTANT	INTERMEDIATE	RESISTANT	NOT_FOUND	INTERMEDIATE	folP_R228SgyrA_D95AgyrA_S91FmtrR_disruptedparC_D86NpenA_ins346DrpsJ_V57M	0	6
627777.fasta	627777	SAMEA6533567	PRJEB32435	26	M	NA	NA	SEKRET	ANUS	2018-05-04	NA	NA	NA	NA	NA	NA	NA	NA	NA	8143	9	27	426	NOT_FOUND	NOT_FOUND	NOT_FOUND	RESISTANT	INTERMEDIATE	RESISTANT	NOT_FOUND	INTERMEDIATE	folP_R228SgyrA_D95AgyrA_S91FmtrR_disruptedparC_D86NpenA_ins346DrpsJ_V57M	0	6
635637.fasta	635637	SAMEA6533725	PRJEB32435	46	M	NA	NA	SEKRET	ANUS	2018-08-07	NA	NA	NA	NA	NA	NA	NA	NA	NA	13142	9	27	2601	NOT_FOUND	NOT_FOUND	NOT_FOUND	RESISTANT	INTERMEDIATE	RESISTANT	NOT_FOUND	INTERMEDIATE	folP_R228SgyrA_D95AgyrA_S91FmtrR_disruptedparC_D86NpenA_ins346DrpsJ_V57M	0	6
635923.fasta	635923	SAMEA6533732	PRJEB32435	23	M	NA	NA	SEKRET	URETRA	2018-08-10	NA	NA	NA	NA	NA	NA	NA	NA	NA	13142	9	27	2601	NOT_FOUND	NOT_FOUND	NOT_FOUND	RESISTANT	INTERMEDIATE	RESISTANT	NOT_FOUND	INTERMEDIATE	folP_R228SgyrA_D95AgyrA_S91FmtrR_disruptedparC_D86NpenA_ins346DrpsJ_V57M	0	6
645553.fasta	645553	SAMEA6533954	PRJEB32435	38	M	NA	NA	SEKRET	URETRA	2018-11-22	NA	NA	NA	NA	NA	NA	NA	NA	NA	14107	9	27	5c2114ded4e	NOT_FOUND	NOT_FOUND	NOT_FOUND	RESISTANT	INTERMEDIATE	RESISTANT	NOT_FOUND	INTERMEDIATE	folP_R228SgyrA_D95AgyrA_S91FmtrR_disruptedparC_D86NpenA_ins346DrpsJ_V57M	0	6
662000.fasta	662000	SAMEA6534191	PRJEB32435	21	M	Viken	East	Sekret	Anus	2019-04-25	0,25	0,016	0,004	1	0,25	16	0,5	NA	NA	8143	9	27	426	NOT_FOUND	NOT_FOUND	NOT_FOUND	RESISTANT	INTERMEDIATE	RESISTANT	NOT_FOUND	INTERMEDIATE	folP_R228SgyrA_D95AgyrA_S91FmtrR_disruptedparC_D86NpenA_ins346DrpsJ_V57M	0	6
662488.fasta	662488	SAMEA6534205	PRJEB32435	21	M	Viken	East	Sekret	Anus	2019-04-26	0,5	0,016	0,002	1	0,25	16	0,25	Ikke utført	Ikke påvist	8143	9	27	426	NOT_FOUND	NOT_FOUND	NOT_FOUND	RESISTANT	INTERMEDIATE	RESISTANT	NOT_FOUND	INTERMEDIATE	folP_R228SgyrA_D95AgyrA_S91FmtrR_disruptedparC_D86NpenA_ins346DrpsJ_V57M	0	6
663555.fasta	663555	SAMEA6534236	PRJEB32435	26	M	Oslo	East	Sekret	Hals	2019-05-13	0,5	0,016	0,002	1	0,25	16	0,5	4	Ikke påvist	13142	9	27	2601	NOT_FOUND	NOT_FOUND	NOT_FOUND	RESISTANT	INTERMEDIATE	RESISTANT	NOT_FOUND	INTERMEDIATE	folP_R228SgyrA_D95AgyrA_S91FmtrR_disruptedparC_D86NpenA_ins346DrpsJ_V57M	0	6
671406.fasta	671406	SAMEA6534566	PRJEB32435	53	M	Rogaland	West	Sekret	Urethra	2019-08-29	0,5	0,016	0,016	2	32	16	0,5	4	Påvist	8143	9	27	e352f9d48afe	NOT_FOUND	NOT_FOUND	NOT_FOUND	RESISTANT	INTERMEDIATE	RESISTANT	NOT_FOUND	INTERMEDIATE	folP_R228SgyrA_D95AgyrA_S91FmtrR_disruptedparC_D86NpenA_ins346DrpsJ_V57M	0	6
22GB001095.fasta	22GB001095	SAMEA117567643	PRJEB83897	68	M	Trøndelag	North	Sekret	Urethra	2022-09-02	0,25	0,016	0,016	2	0,032	16	0,5	8	Negativ	11968	9	27	d7ee1a01363	NOT_FOUND	NOT_FOUND	NOT_FOUND	RESISTANT	INTERMEDIATE	RESISTANT	NOT_FOUND	INTERMEDIATE	folP_R228SgyrA_D95AgyrA_S91FmtrR_disruptedparC_D86NrpsJ_V57M	0	6
671187.fasta	671187	SAMEA6534554	PRJEB32435	39	M	Oslo	East	Sekret	Hals	2019-08-27	0,5	0,016	0,004	2	0,5	16	0,5	8	Ikke påvist	8143	9	27	b170b9aa9c1	NOT_FOUND	NOT_FOUND	NOT_FOUND	RESISTANT	INTERMEDIATE	RESISTANT	NOT_FOUND	INTERMEDIATE	folP_R228SgyrA_D95AgyrA_S91FmtrR_disruptedparC_D86NrpsJ_V57M	0	6
20GB001303.fasta	20GB001303	SAMEA117566936	PRJEB83897	42	M	Oslo	East	Sekret	Urethra	2020-10-19	0,5	0,016	0,002	4	0,25	16	0,5	NA	NA	8143	9	27	2982	NOT_FOUND	NOT_FOUND	NOT_FOUND	RESISTANT	INTERMEDIATE	RESISTANT	NOT_FOUND	INTERMEDIATE	folP_R228SgyrA_D95AgyrA_S91FmtrR_disruptedparC_S87RpenA_ins346DponA1_L421PrpsJ_V57M	0	6
664765.fasta	664765	SAMEA6534276	PRJEB32435	31	M	Oslo	East	Sekret	Anus	2019-05-27	0,5	0,032	0,016	8	0,5	16	1	16	Ikke påvist	8143	9	27	2982	NOT_FOUND	NOT_FOUND	NOT_FOUND	RESISTANT	INTERMEDIATE	RESISTANT	NOT_FOUND	INTERMEDIATE	folP_R228SgyrA_D95AgyrA_S91FmtrR_disruptedparC_S87RpenA_ins346DponA1_L421PrpsJ_V57M	0	6
20GB000759.fasta	20GB000759	SAMEA117566741	PRJEB83897	70	M	Oslo	East	Sekret		2020-03-24	0,25	0,016	0,016	4	0,25	16	1	NA	Ikke påvist	13526	9	27	436	NOT_FOUND	NOT_FOUND	NOT_FOUND	RESISTANT	INTERMEDIATE	RESISTANT	NOT_FOUND	INTERMEDIATE	folP_R228SgyrA_D95AgyrA_S91FmtrR_disruptedparC_S87RpenA_ins346DrpsJ_V57M	0	6
21GB000691.fasta	21GB000691	SAMEA117567121	PRJEB83897	28	F	Oslo	East	Sekret	Cervix uter	2021-11-04	0,5	0,016	0,002	2	0,125	16	0,25	NA	Ikke påvist	13526	9	27	436	NOT_FOUND	NOT_FOUND	NOT_FOUND	RESISTANT	INTERMEDIATE	RESISTANT	NOT_FOUND	INTERMEDIATE	folP_R228SgyrA_D95AgyrA_S91FmtrR_disruptedparC_S87RpenA_ins346DrpsJ_V57M	0	6
554590.fasta	554590	SAMEA5608456	PRJEB32435	69	M	Oslo	East	Sekret	Urethra	2016-08-15	0,25	0,016	0,002	4	0,125	16	0,5	NA	NA	8143	9	27	436	NOT_FOUND	NOT_FOUND	NOT_FOUND	RESISTANT	INTERMEDIATE	RESISTANT	NOT_FOUND	INTERMEDIATE	folP_R228SgyrA_D95AgyrA_S91FmtrR_disruptedparC_S87RpenA_ins346DrpsJ_V57M	0	6
555832.fasta	555832	SAMEA5608516	PRJEB32435	28	M	Oslo	East	Sekret	Anus	2016-09-05	0,5	0,016	0,004	8	0,125	16	0,5	NA	NA	8143	9	27	436	NOT_FOUND	NOT_FOUND	NOT_FOUND	RESISTANT	INTERMEDIATE	RESISTANT	NOT_FOUND	INTERMEDIATE	folP_R228SgyrA_D95AgyrA_S91FmtrR_disruptedparC_S87RpenA_ins346DrpsJ_V57M	0	6
594502.fasta	594502	SAMEA5608975	PRJEB32435	30	M	Innlandet	East	Sekret	Urethra	2017-07-26	0,25	0,016	0,002	4	0,125	8	0,5	NA	NA	7371	9	27	1350	NOT_FOUND	NOT_FOUND	NOT_FOUND	RESISTANT	INTERMEDIATE	RESISTANT	NOT_FOUND	INTERMEDIATE	folP_R228SgyrA_D95AgyrA_S91FmtrR_disruptedparC_S87RpenA_ins346DrpsJ_V57M	0	6
606714.fasta	606714	SAMEA6533394	PRJEB32435	37	M	Oslo	East	Sekret	Urethra	2017-11-20	0,25	0,016	0,002	4	0,25	16	0,5	NA	NA	8143	9	27	436	NOT_FOUND	NOT_FOUND	NOT_FOUND	RESISTANT	INTERMEDIATE	RESISTANT	NOT_FOUND	INTERMEDIATE	folP_R228SgyrA_D95AgyrA_S91FmtrR_disruptedparC_S87RpenA_ins346DrpsJ_V57M	0	6
609084.fasta	609084	SAMEA6533414	PRJEB32435	41	M	Oslo	East	Sekret	Urethra	2017-12-11	0,5	0,016	0,002	4	0,25	16	0,5	NA	NA	8143	9	27	436	NOT_FOUND	NOT_FOUND	NOT_FOUND	RESISTANT	INTERMEDIATE	RESISTANT	NOT_FOUND	INTERMEDIATE	folP_R228SgyrA_D95AgyrA_S91FmtrR_disruptedparC_S87RpenA_ins346DrpsJ_V57M	0	6
610102.fasta	610102	SAMEA5609158	PRJEB32435	37	M	Agder	South	Sekret	Hals	2017-12-20	0,5	0,016	0,002	4	0,25	16	0,5	NA	NA	8143	9	27	436	NOT_FOUND	NOT_FOUND	NOT_FOUND	RESISTANT	INTERMEDIATE	RESISTANT	NOT_FOUND	INTERMEDIATE	folP_R228SgyrA_D95AgyrA_S91FmtrR_disruptedparC_S87RpenA_ins346DrpsJ_V57M	0	6
615958.fasta	615958	SAMEA6963040	PRJEB32435	22	M	Viken	East	Sekret	Anus	2018-01-30	0,5	0,016	0,002	4	0,25	16	0,5	NA	NA	8143	9	27	436	NOT_FOUND	NOT_FOUND	NOT_FOUND	RESISTANT	INTERMEDIATE	RESISTANT	NOT_FOUND	INTERMEDIATE	folP_R228SgyrA_D95AgyrA_S91FmtrR_disruptedparC_S87RpenA_ins346DrpsJ_V57M	0	6
630842.fasta	630842	SAMEA6533608	PRJEB32435	34	M	NA	NA	SEKRET	ANUS	2018-06-01	NA	NA	NA	NA	NA	NA	NA	NA	NA	8143	9	27	436	NOT_FOUND	NOT_FOUND	NOT_FOUND	RESISTANT	INTERMEDIATE	RESISTANT	NOT_FOUND	INTERMEDIATE	folP_R228SgyrA_D95AgyrA_S91FmtrR_disruptedparC_S87RpenA_ins346DrpsJ_V57M	0	6
642366.fasta	642366	SAMEA6533898	PRJEB32435	24	M	NA	NA	SEKRET	URETRA	2018-10-22	0,38	0,016	0,016	2	NA	NA	NA	NA	NA	13526	9	27	436	NOT_FOUND	NOT_FOUND	NOT_FOUND	RESISTANT	INTERMEDIATE	RESISTANT	NOT_FOUND	INTERMEDIATE	folP_R228SgyrA_D95AgyrA_S91FmtrR_disruptedparC_S87RpenA_ins346DrpsJ_V57M	0	6
22GB001290.fasta	22GB001290	SAMEA117567739	PRJEB83897	56	M	Oslo	East	Sekret	Urethra	2022-10-07	256	0,016	0,002	0,064	0,25	16	0,25	4	Negativ	-	Other	4	01660adcd05	NOT_FOUND	NOT_FOUND	NOT_FOUND	RESISTANT	INTERMEDIATE	RESISTANT	NOT_FOUND	INTERMEDIATE	folP_R228SgyrA_D95AgyrA_S91FmtrR_disruptedpenA_ins346DrplD_G70DrpsJ_V57M	0	6
22GB000884.fasta	22GB000884	SAMEA117567507	PRJEB83897	36	M	Oslo	East	Sekret	Anus	2022-07-20	0,5	0,032	0,032	1	1	16	0,5	4	Negativ	7827	6	28	4541	NOT_FOUND	NOT_FOUND	NOT_FOUND	RESISTANT	INTERMEDIATE	RESISTANT	NOT_FOUND	RESISTANT	folP_R228SgyrA_D95AgyrA_S91FmtrR_G45DmtrR_promoter_a-57delparC_D86NpenA_A501VpenA_ins346DpenA_P5	0	6
23GB000827.fasta	23GB000827	SAMEA117568339	PRJEB83897	29	M	NA	NA	Sekret	Hals	2023-03-30	0,25	0,016	0,008	8	0,25	32	1	NA	Negativ	7827	6	28	2281	NOT_FOUND	NOT_FOUND	NOT_FOUND	RESISTANT	INTERMEDIATE	RESISTANT	NOT_FOUND	RESISTANT	folP_R228SgyrA_D95AgyrA_S91FmtrR_G45DmtrR_promoter_a-57delparC_D86NpenA_A501VpenA_ins346DpenA_P5	0	6
625372.fasta	625372	SAMEA6533531	PRJEB32435	48	M	NA	NA	SEKRET	REKTUM	2018-04-09	NA	NA	NA	NA	NA	NA	NA	NA	NA	7827	6	28	1975	NOT_FOUND	NOT_FOUND	NOT_FOUND	RESISTANT	INTERMEDIATE	RESISTANT	NOT_FOUND	RESISTANT	folP_R228SgyrA_D95AgyrA_S91FmtrR_G45DmtrR_promoter_a-57delparC_D86NpenA_A501VpenA_ins346DpenA_P5	0	6
625970.fasta	625970	SAMEA6533544	PRJEB32435	37	M	NA	NA	SEKRET	URETRA	2018-04-10	0,25	0,064	0,016	1	0,25	8	1	-	NA	7827	6	28	1975	NOT_FOUND	NOT_FOUND	NOT_FOUND	RESISTANT	INTERMEDIATE	RESISTANT	NOT_FOUND	RESISTANT	folP_R228SgyrA_D95AgyrA_S91FmtrR_G45DmtrR_promoter_a-57delparC_D86NpenA_A501VpenA_ins346DpenA_P5	0	6
632824.fasta	632824	SAMEA6533641	PRJEB32435	30	M	NA	NA	SEKRET	URETRA	2018-06-28	NA	NA	NA	NA	NA	NA	NA	NA	NA	7827	6	28	1975	NOT_FOUND	NOT_FOUND	NOT_FOUND	RESISTANT	INTERMEDIATE	RESISTANT	NOT_FOUND	RESISTANT	folP_R228SgyrA_D95AgyrA_S91FmtrR_G45DmtrR_promoter_a-57delparC_D86NpenA_A501VpenA_ins346DpenA_P5	0	6
633599.fasta	633599	SAMEA6533669	PRJEB32435	60	M	NA	NA	SEKRET	ANUS	2018-07-05	NA	NA	NA	NA	NA	NA	NA	NA	NA	7827	6	28	1975	NOT_FOUND	NOT_FOUND	NOT_FOUND	RESISTANT	INTERMEDIATE	RESISTANT	NOT_FOUND	RESISTANT	folP_R228SgyrA_D95AgyrA_S91FmtrR_G45DmtrR_promoter_a-57delparC_D86NpenA_A501VpenA_ins346DpenA_P5	0	6
21GB000765.fasta	21GB000765	SAMEA117567142	PRJEB83897	31	M	Rogaland	West	Sekret	Urethra	2021-11-25	0,5	0,016	0,016	4	0,5	16	1	NA	Ikke påvist	10314	2	10	3971	NOT_FOUND	NOT_FOUND	NOT_FOUND	RESISTANT	INTERMEDIATE	RESISTANT	NOT_FOUND	RESISTANT	folP_R228SgyrA_D95AgyrA_S91FmtrR_promoter_a-57delparC_S87RpenA_G542SpenA_ins346DponA1_L421PrpsJ_V5	0	6
22GB000024.fasta	22GB000024	SAMEA117567190	PRJEB83897	16	F	Rogaland	West	Sekret	Vagina	2021-12-31	0,5	0,016	0,016	8	0,5	16	1	NA	Ikke påvist	10314	2	10	3971	NOT_FOUND	NOT_FOUND	NOT_FOUND	RESISTANT	INTERMEDIATE	RESISTANT	NOT_FOUND	RESISTANT	folP_R228SgyrA_D95AgyrA_S91FmtrR_promoter_a-57delparC_S87RpenA_G542SpenA_ins346DponA1_L421PrpsJ_V5	0	6
22GB000134.fasta	22GB000134	SAMEA117567240	PRJEB83897	24	F	Rogaland	West	Sekret	Vagina	2022-02-09	0,5	0,016	0,016	4	0,5	16	1	NA	Ikke påvist	10314	2	10	3971	NOT_FOUND	NOT_FOUND	NOT_FOUND	RESISTANT	INTERMEDIATE	RESISTANT	NOT_FOUND	RESISTANT	folP_R228SgyrA_D95AgyrA_S91FmtrR_promoter_a-57delparC_S87RpenA_G542SpenA_ins346DponA1_L421PrpsJ_V5	0	6
22GB000136.fasta	22GB000136	SAMEA117567242	PRJEB83897	30	M	Rogaland	West	Sekret	Anus	2022-02-10	0,5	0,016	0,016	8	0,5	16	1	NA	Ikke påvist	10314	2	10	3971	NOT_FOUND	NOT_FOUND	NOT_FOUND	RESISTANT	INTERMEDIATE	RESISTANT	NOT_FOUND	RESISTANT	folP_R228SgyrA_D95AgyrA_S91FmtrR_promoter_a-57delparC_S87RpenA_G542SpenA_ins346DponA1_L421PrpsJ_V5	0	6
22GB001316.fasta	22GB001316	SAMEA117567752	PRJEB83897	27	F	Rogaland	West	Sekret	Cervix uter	2022-10-11	0,5	0,016	0,016	4	0,5	16	1	8	Negativ	10314	2	10	3971	NOT_FOUND	NOT_FOUND	NOT_FOUND	RESISTANT	INTERMEDIATE	RESISTANT	NOT_FOUND	RESISTANT	folP_R228SgyrA_D95AgyrA_S91FmtrR_promoter_a-57delparC_S87RpenA_G542SpenA_ins346DponA1_L421PrpsJ_V5	0	6
667924.fasta	667924	SAMEA6534443	PRJEB32435	36	M	Oslo	East	Sekret	Anus	2019-07-12	0,5	0,016	0,008	2	0,5	16	0,5	4	Ikke påvist	7822	2	10	2883	NOT_FOUND	NOT_FOUND	NOT_FOUND	RESISTANT	INTERMEDIATE	RESISTANT	NOT_FOUND	RESISTANT	folP_R228SgyrA_D95AgyrA_S91FmtrR_promoter_a-57delparC_S87RpenA_G542SpenA_ins346DponA1_L421PrpsJ_V5	0	6
552874.fasta	552874	SAMEA5608396	PRJEB32435	32	M	Oslo	East	Sekret	Urethra	2016-03-08	0,25	0,5	0,125	32	4	16	2	NA	NA	1901	11	11	1392	NOT_FOUND	NOT_FOUND	RESISTANT	RESISTANT	RESISTANT	RESISTANT	NOT_FOUND	RESISTANT	folP_R228SgyrA_D95AgyrA_S91FmtrR_promoter_a-57delparC_S87RpenA_G545SpenA_I312MpenA_V316TponA1_L4	0	6
536036.fasta	536036	SAMEA5608291	PRJEB32435	58	M	Troms og Finnmark	North	Sekret		2016-06-02	1	0,125	0,064	16	2	16	4	NA	NA	1901	11	11	507	NOT_FOUND	NOT_FOUND	RESISTANT	RESISTANT	RESISTANT	RESISTANT	NOT_FOUND	RESISTANT	folP_R228SgyrA_D95AgyrA_S91FmtrR_promoter_a-57delparC_S87RpenA_G545SpenA_I312MpenA_V316TponA1_L4	0	6
566760.fasta	566760	SAMEA5608660	PRJEB32435	33	M	Viken	East	Sekret	Urethra	2017-02-14	0,5	0,125	0,064	8	1	16	2	NA	NA	1901	11	11	507	NOT_FOUND	NOT_FOUND	RESISTANT	RESISTANT	RESISTANT	RESISTANT	NOT_FOUND	RESISTANT	folP_R228SgyrA_D95AgyrA_S91FmtrR_promoter_a-57delparC_S87RpenA_G545SpenA_I312MpenA_V316TponA1_L4	0	6
663048.fasta	663048	SAMEA6534220	PRJEB32435	30	M	Nordland	North	Sekret	Urethra	2019-05-09	0,25	0,064	0,016	16	0,5	32	2	Ikke utført	Ikke påvist	1901	11	11	507	NOT_FOUND	NOT_FOUND	RESISTANT	RESISTANT	RESISTANT	RESISTANT	NOT_FOUND	RESISTANT	folP_R228SgyrA_D95AgyrA_S91FmtrR_promoter_a-57delparC_S87RpenA_G545SpenA_I312MpenA_V316TponA1_L4	0	6
673918.fasta	673918	SAMEA6534628	PRJEB32435	33	M	Vestland	West	Sekret	Urethra	2019-10-04	0,25	0,125	0,064	8	1	16	2	8	Ikke påvist	1901	11	11	507	NOT_FOUND	NOT_FOUND	RESISTANT	RESISTANT	RESISTANT	RESISTANT	NOT_FOUND	RESISTANT	folP_R228SgyrA_D95AgyrA_S91FmtrR_promoter_a-57delparC_S87RpenA_G545SpenA_I312MpenA_V316TponA1_L4	0	6
22GB000719.fasta	22GB000719	SAMEA117567427	PRJEB83897	22	M	Oslo	East	Sekret	Urethra	2022-06-13	0,125	0,016	0,002	1	0,25	8	0,125	4	Negativ	14610	3	12	5323	NOT_FOUND	NOT_FOUND	NOT_FOUND	RESISTANT	INTERMEDIATE	RESISTANT	NOT_FOUND	INTERMEDIATE	folP_R228SgyrA_D95AgyrA_S91FmtrR_promoter_a-57delparC_S87RpenA_ins346DponA1_L421P	0	6
22GB000906.fasta	22GB000906	SAMEA117567519	PRJEB83897	24	M	Trøndelag	North	Sekret	Urethra	2022-07-27	0,5	0,016	0,016	2	0,25	16	0,25	4	Negativ	14610	3	12	5323	NOT_FOUND	NOT_FOUND	NOT_FOUND	RESISTANT	INTERMEDIATE	RESISTANT	NOT_FOUND	INTERMEDIATE	folP_R228SgyrA_D95AgyrA_S91FmtrR_promoter_a-57delparC_S87RpenA_ins346DponA1_L421P	0	6
22GB001068.fasta	22GB001068	SAMEA117567624	PRJEB83897	23	M	Vestland	West	Sekret	Urethra	2022-08-29	0,125	0,016	0,016	2	0,25	16	0,25	4	Negativ	14610	3	12	5323	NOT_FOUND	NOT_FOUND	NOT_FOUND	RESISTANT	INTERMEDIATE	RESISTANT	NOT_FOUND	INTERMEDIATE	folP_R228SgyrA_D95AgyrA_S91FmtrR_promoter_a-57delparC_S87RpenA_ins346DponA1_L421P	0	6
21GB000309.fasta	21GB000309	SAMEA117567034	PRJEB83897	49	M	Viken	East	Sekret	Urethra	2021-05-24	1	0,016	0,016	4	0,5	8	1	NA	Ikke påvist	9903	2	10	c07a6ac5322(	NOT_FOUND	NOT_FOUND	NOT_FOUND	RESISTANT	INTERMEDIATE	RESISTANT	NOT_FOUND	RESISTANT	folP_R228SgyrA_D95AgyrA_S91FmtrR_promoter_a-57delparC_S87RpenA_ins346DponA1_L421PrpsJ_V57M	0	6
21GB000823.fasta	21GB000823	SAMEA117567162	PRJEB83897	28	M	Vestland	West	Sekret	Urethra	2021-12-10	0,5	0,016	0,016	0,016	0,25	8	0,25	NA	Ikke påvist	8156	3	12	8c87cf23370t	NOT_FOUND	NOT_FOUND	NOT_FOUND	RESISTANT	INTERMEDIATE	RESISTANT	NOT_FOUND	INTERMEDIATE	folP_R228SgyrA_D95AmtrR_promoter_a-57delpenA_ins346DponA1_L421P	0	6
22GB000045.fasta	22GB000045	SAMEA117567197	PRJEB83897	32	M	Oslo	East	Sekret	Anus	2022-01-03	0,25	0,016	0,002	0,016	0,125	8	0,125	NA	Ikke påvist	8156	3	12	8c87cf23370t	NOT_FOUND	NOT_FOUND	NOT_FOUND	RESISTANT	INTERMEDIATE	RESISTANT	NOT_FOUND	INTERMEDIATE	folP_R228SgyrA_D95AmtrR_promoter_a-57delpenA_ins346DponA1_L421P	0	6
22GB000682.fasta	22GB000682	SAMEA117567407	PRJEB83897	29	M	Trøndelag	North	Sekret	Ureter	2022-06-02	0,5	0,016	0,016	0,016	0,125	16	0,25	8	Negativ	8156	3	12	8c87cf23370t	NOT_FOUND	NOT_FOUND	NOT_FOUND	RESISTANT	INTERMEDIATE	RESISTANT	NOT_FOUND	INTERMEDIATE	folP_R228SgyrA_D95AmtrR_promoter_a-57delpenA_ins346DponA1_L421P	0	6
22GB001132.fasta	22GB001132	SAMEA117567662	PRJEB83897	31	M	Oslo	East	Sekret	Hals	2022-09-07	0,5	0,016	0,002	0,008	0,125	16	0,064	4	Negativ	8156	3	12	8c87cf23370t	NOT_FOUND	NOT_FOUND	NOT_FOUND	RESISTANT	INTERMEDIATE	RESISTANT	NOT_FOUND	INTERMEDIATE	folP_R228SgyrA_D95AmtrR_promoter_a-57delpenA_ins346DponA1_L421P	0	6
23GB000113.fasta	23GB000113	SAMEA117568005	PRJEB83897	30	M	Oslo	East	Sekret	Anus	2023-01-06	0,5	0,016	0,016	0,032	0,25	8	0,25	NA	Negativ	8156	3	12	2f6215f0453a	NOT_FOUND	NOT_FOUND	NOT_FOUND	RESISTANT	INTERMEDIATE	RESISTANT	NOT_FOUND	INTERMEDIATE	folP_R228SgyrA_D95AmtrR_promoter_a-57delpenA_ins346DponA1_L421P	0	6
20GB000444.fasta	20GB000444	SAMEA117566633	PRJEB83897	32	M	Oslo	East	Sekret	Urethra	2020-02-03	0,125	0,032	0,032	2	0,5	8	0,25	NA	Ikke påvist	1901	11	11	2654	NOT_FOUND	NOT_FOUND	NOT_FOUND	RESISTANT	INTERMEDIATE	RESISTANT	NOT_FOUND	INTERMEDIATE	folP_R228SgyrA_D95GgyrA_S91FmtrR_A39TparC_S87RpenA_G542SpenA_ins346DponA1_L421PrpsJ_V57M	0	6
515710.fasta	515710	SAMEA5608217	PRJEB32435	52	F	Oslo	East	Sekret	Vagina	2016-01-05	0,5	0,032	0,016	4	1	8	1	NA	NA	1901	11	11	1332	NOT_FOUND	NOT_FOUND	NOT_FOUND	RESISTANT	INTERMEDIATE	RESISTANT	NOT_FOUND	INTERMEDIATE	folP_R228SgyrA_D95GgyrA_S91FmtrR_A39TparC_S87RpenA_G542SpenA_ins346DponA1_L421PrpsJ_V57M	0	6
577124.fasta	577124	SAMEA5608808	PRJEB32435	29	M	Oslo	East	Sekret	Urethra	2017-05-08	0,5	0,016	0,016	4	1	8	0,5	NA	NA	1901	11	11	2654	NOT_FOUND	NOT_FOUND	NOT_FOUND	RESISTANT	INTERMEDIATE	RESISTANT	NOT_FOUND	INTERMEDIATE	folP_R228SgyrA_D95GgyrA_S91FmtrR_A39TparC_S87RpenA_G542SpenA_ins346DponA1_L421PrpsJ_V57M	0	6
669902.fasta	669902	SAMEA6534507	PRJEB32435	22	M	Trøndelag	North	Sekret	Urethra	2019-08-12	0,25	0,016	0,008	0,5	0,5	16	0,5	4	Ikke påvist	14787	11	11	2654	NOT_FOUND	NOT_FOUND	NOT_FOUND	RESISTANT	INTERMEDIATE	RESISTANT	NOT_FOUND	INTERMEDIATE	folP_R228SgyrA_D95GgyrA_S91FmtrR_A39TparC_S87RpenA_G542SpenA_ins346DponA1_L421PrpsJ_V57M	0	6
670858.fasta	670858	SAMEA6534545	PRJEB32435	21	F	Trøndelag	North	Sekret	Cervix uter	2019-08-21	0,5	0,016	0,008	4	0,5	16	1	8	Ikke påvist	14787	11	11	2654	NOT_FOUND	NOT_FOUND	NOT_FOUND	RESISTANT	INTERMEDIATE	RESISTANT	NOT_FOUND	INTERMEDIATE	folP_R228SgyrA_D95GgyrA_S91FmtrR_A39TparC_S87RpenA_G542SpenA_ins346DponA1_L421PrpsJ_V57M	0	6

20GB000738.fasta	20GB000738	SAMEA117566733	PRJEB83897	42	M	Viken	East	Puss	Urethra	2020-03-18	0,5	0,125	0,032	4	1	16	1	NA	Ikke påvist	1901	11	11	303	NOT_FOUND	NOT_FOUND	RESISTANT	RESISTANT	INTERMEDIATE	RESISTANT	NOT_FOUND	RESISTANT	folP_R228SgyrA_D95GgyrA_S91FmtrR_promoter_a-57delparC_S87RpenA_G545SpenA_I312MpenA_V316TponA1_L4	0	6
20GB000750.fasta	20GB000750	SAMEA117566736	PRJEB83897	42	M	Viken	East	Sekret	Urethra	2020-03-23	0,5	0,125	0,032	4	1	16	1	NA	Ikke påvist	1901	11	11	303	NOT_FOUND	NOT_FOUND	RESISTANT	RESISTANT	INTERMEDIATE	RESISTANT	NOT_FOUND	RESISTANT	folP_R228SgyrA_D95GgyrA_S91FmtrR_promoter_a-57delparC_S87RpenA_G545SpenA_I312MpenA_V316TponA1_L4	0	6
620084.fasta	620084	SAMEA6963024	PRJEB32435	36	M	NA	NA	SEKRET	URETRA	2018-02-26	NA	NA	NA	NA	NA	NA	NA	NA	NA	8137	11	11	250	NOT_FOUND	NOT_FOUND	RESISTANT	RESISTANT	INTERMEDIATE	RESISTANT	NOT_FOUND	RESISTANT	folP_R228SgyrA_D95GgyrA_S91FmtrR_promoter_a-57delparC_S87RpenA_G545SpenA_I312MpenA_V316TponA1_L4	0	6
623295.fasta	623295	SAMEA6533510	PRJEB32435	26	F	NA	NA	SEKRET	CERVIXUT	2018-03-20	NA	NA	NA	NA	NA	NA	NA	NA	NA	8137	11	11	250	NOT_FOUND	NOT_FOUND	RESISTANT	RESISTANT	INTERMEDIATE	RESISTANT	NOT_FOUND	RESISTANT	folP_R228SgyrA_D95GgyrA_S91FmtrR_promoter_a-57delparC_S87RpenA_G545SpenA_I312MpenA_V316TponA1_L4	0	6
655081.fasta	655081	SAMEA6534088	PRJEB32435	20	F	Trøndelag	North	Sekret	Cervix uter	2019-02-21	0,25	0,125	0,032	4	1	16	0,5	NA	NA	1901	11	11	250	NOT_FOUND	NOT_FOUND	RESISTANT	RESISTANT	INTERMEDIATE	RESISTANT	NOT_FOUND	RESISTANT	folP_R228SgyrA_D95GgyrA_S91FmtrR_promoter_a-57delparC_S87RpenA_G545SpenA_I312MpenA_V316TponA1_L4	0	6
606473.fasta	606473	SAMEA6533388	PRJEB32435	27	M	Vestland	West	Sekret	Urethra	2017-11-22	0,25	0,032	0,016	8	1	8	1	NA	NA	11969	Other	22	d4794871cc2	NOT_FOUND	NOT_FOUND	NOT_FOUND	RESISTANT	INTERMEDIATE	RESISTANT	NOT_FOUND	INTERMEDIATE	folP_R228SgyrA_D95GgyrA_S91FmtrR_promoter_a-57delparC_S87RpenA_ins346DpenA_P551SporB1b_G120K	0	6
678700.fasta	678700	SAMEA6534733	PRJEB32435	35	M	Vestfold og Telemark	East	Sekret	Urethra	2019-11-22	0,25	0,016	0,004	8	0,25	8	0,5	8	Ikke påvist	7365	12	24	2714	NOT_FOUND	NOT_FOUND	NOT_FOUND	RESISTANT	INTERMEDIATE	RESISTANT	NOT_FOUND	RESISTANT	folP_R228SgyrA_D95NgyrA_S91FmtrR_G45DmtrR_promoter_a-57delparC_S87RpenA_ins346DponA1_L421PrpsJ_V57	0	6
20GB000570.fasta	20GB000570	SAMEA117566664	PRJEB83897	21	F	Troms og Finnmark	North	Sekret	Cervix uter	2020-02-17	0,125	0,125	0,032	32	1	8	0,5	4	Ikke påvist	7363	Other	13	348	NOT_FOUND	NOT_FOUND	RESISTANT	RESISTANT	RESISTANT	RESISTANT	NOT_FOUND	INTERMEDIATE	folP_R228SgyrA_D95NgyrA_S91FparC_S87RparC_S88PpenA_G545SpenA_I312MpenA_V316TponA1_L421PporB1b_A1	0	6
20GB000783.fasta	20GB000783	SAMEA117566749	PRJEB83897	47	M	Oslo	East	Sekret	Kjønnsorga	2020-04-01	0,125	0,25	0,032	32	1	16	1	NA	Ikke påvist	7363	Other	13	1236	NOT_FOUND	NOT_FOUND	RESISTANT	RESISTANT	INTERMEDIATE	RESISTANT	NOT_FOUND	INTERMEDIATE	folP_R228SgyrA_D95NgyrA_S91FparC_S87RparC_S88PpenA_G545SpenA_I312MpenA_V316TponA1_L421PrpsJ_V57M	0	6
20GB001147.fasta	20GB001147	SAMEA117566859	PRJEB83897	20	M	Oslo	East	Sekret	Urethra	2020-08-12	0,25	0,25	0,032	32	1	8	1	NA	Ikke påvist	7363	Other	13	710	NOT_FOUND	NOT_FOUND	RESISTANT	RESISTANT	INTERMEDIATE	RESISTANT	NOT_FOUND	INTERMEDIATE	folP_R228SgyrA_D95NgyrA_S91FparC_S87RparC_S88PpenA_G545SpenA_I312MpenA_V316TponA1_L421PrpsJ_V57M	0	6
21GB000351.fasta	21GB000351	SAMEA117567043	PRJEB83897	52	F	Vestland	West	Sekret	Cervix uter	2021-06-29	0,125	0,25	0,032	32	1	16	0,5	NA	Ikke påvist	7363	Other	13	1236	NOT_FOUND	NOT_FOUND	RESISTANT	RESISTANT	INTERMEDIATE	RESISTANT	NOT_FOUND	INTERMEDIATE	folP_R228SgyrA_D95NgyrA_S91FparC_S87RparC_S88PpenA_G545SpenA_I312MpenA_V316TponA1_L421PrpsJ_V57M	0	6
21GB000837.fasta	21GB000837	SAMEA117567172	PRJEB83897	23	M	Oslo	East	Sekret	Urethra	2021-12-14	0,25	0,25	0,064	16	0,5	16	1	NA	Ikke påvist	7363	Other	13	1236	NOT_FOUND	NOT_FOUND	RESISTANT	RESISTANT	INTERMEDIATE	RESISTANT	NOT_FOUND	INTERMEDIATE	folP_R228SgyrA_D95NgyrA_S91FparC_S87RparC_S88PpenA_G545SpenA_I312MpenA_V316TponA1_L421PrpsJ_V57M	0	6
21GB000847.fasta	21GB000847	SAMEA117567174	PRJEB83897	35	M	Oslo	East	Sekret	Anus	2021-12-14	0,125	0,064	0,008	4	0,5	8	4	NA	Ikke påvist	7363	Other	13	1236	NOT_FOUND	NOT_FOUND	RESISTANT	RESISTANT	INTERMEDIATE	RESISTANT	NOT_FOUND	INTERMEDIATE	folP_R228SgyrA_D95NgyrA_S91FparC_S87RparC_S88PpenA_G545SpenA_I312MpenA_V316TponA1_L421PrpsJ_V57M	0	6
537170.fasta	537170	SAMEA5608302	PRJEB32435	17	F	Viken	East	Sekret	Vagina	2016-06-16	0,064	0,25	0,032	32	0,5	8	0,5	NA	NA	7363	Other	13	710	NOT_FOUND	NOT_FOUND	RESISTANT	RESISTANT	INTERMEDIATE	RESISTANT	NOT_FOUND	INTERMEDIATE	folP_R228SgyrA_D95NgyrA_S91FparC_S87RparC_S88PpenA_G545SpenA_I312MpenA_V316TponA1_L421PrpsJ_V57M	0	6
537844.fasta	537844	SAMEA5608304	PRJEB32435	19	F	Viken	East	Sekret	Vagina	2016-06-20	0,064	0,125	0,032	16	0,5	8	0,5	NA	NA	7363	Other	13	710	NOT_FOUND	NOT_FOUND	RESISTANT	RESISTANT	INTERMEDIATE	RESISTANT	NOT_FOUND	INTERMEDIATE	folP_R228SgyrA_D95NgyrA_S91FparC_S87RparC_S88PpenA_G545SpenA_I312MpenA_V316TponA1_L421PrpsJ_V57M	0	6
542910.fasta	542910	SAMEA5608332	PRJEB32435	17	F	Viken	East	Sekret		2016-08-17	0,064	0,125	0,032	32	0,5	16	1	NA	NA	7363	Other	13	710	NOT_FOUND	NOT_FOUND	RESISTANT	RESISTANT	INTERMEDIATE	RESISTANT	NOT_FOUND	INTERMEDIATE	folP_R228SgyrA_D95NgyrA_S91FparC_S87RparC_S88PpenA_G545SpenA_I312MpenA_V316TponA1_L421PrpsJ_V57M	0	6
553566.fasta	553566	SAMEA5608405	PRJEB32435	20	M	Viken	East	Sekret	Urethra	2016-05-13	0,032	0,125	0,016	32	0,25	8	0,25	NA	NA	7363	Other	13	710	NOT_FOUND	NOT_FOUND	RESISTANT	RESISTANT	INTERMEDIATE	RESISTANT	NOT_FOUND	INTERMEDIATE	folP_R228SgyrA_D95NgyrA_S91FparC_S87RparC_S88PpenA_G545SpenA_I312MpenA_V316TponA1_L421PrpsJ_V57M	0	6
553730.fasta	553730	SAMEA5608430	PRJEB32435	18	M	Innlandet	East	Sekret	Urethra	2016-04-07	0,064	0,125	0,016	32	0,25	8	0,5	NA	NA	7363	Other	13	710	NOT_FOUND	NOT_FOUND	RESISTANT	RESISTANT	INTERMEDIATE	RESISTANT	NOT_FOUND	INTERMEDIATE	folP_R228SgyrA_D95NgyrA_S91FparC_S87RparC_S88PpenA_G545SpenA_I312MpenA_V316TponA1_L421PrpsJ_V57M	0	6
554739.fasta	554739	SAMEA5608475	PRJEB32435	20	M	Viken	East	Sekret	Urethra	2016-07-18	0,064	0,125	0,016	16	0,25	8	0,5	NA	NA	7363	Other	13	710	NOT_FOUND	NOT_FOUND	RESISTANT	RESISTANT	INTERMEDIATE	RESISTANT	NOT_FOUND	INTERMEDIATE	folP_R228SgyrA_D95NgyrA_S91FparC_S87RparC_S88PpenA_G545SpenA_I312MpenA_V316TponA1_L421PrpsJ_V57M	0	6
555509.fasta	555509	SAMEA5608495	PRJEB32435	19	F	Viken	East	Sekret	Vagina	2016-09-27	0,032	0,25	0,032	16	0,25	8	0,25	NA	NA	7363	Other	13	710	NOT_FOUND	NOT_FOUND	RESISTANT	RESISTANT	INTERMEDIATE	RESISTANT	NOT_FOUND	INTERMEDIATE	folP_R228SgyrA_D95NgyrA_S91FparC_S87RparC_S88PpenA_G545SpenA_I312MpenA_V316TponA1_L421PrpsJ_V57M	0	6
563915.fasta	563915	SAMEA5608619	PRJEB32435	32	M	Agder	South	Puss	Urethra	2017-01-24	0,25	0,25	0,032	16	0,5	8	0,5	NA	NA	14341	Other	13	710	NOT_FOUND	NOT_FOUND	RESISTANT	RESISTANT	INTERMEDIATE	RESISTANT	NOT_FOUND	INTERMEDIATE	folP_R228SgyrA_D95NgyrA_S91FparC_S87RparC_S88PpenA_G545SpenA_I312MpenA_V316TponA1_L421PrpsJ_V57M	0	6
640474.fasta	640474	SAMEA6533847	PRJEB32435	38	M	NA	NA	SEKRET	URETRA	2018-09-25	0,19	0,064	0,016	12	NA	NA	NA	NA	NA	7363	Other	13	710	NOT_FOUND	NOT_FOUND	RESISTANT	RESISTANT	INTERMEDIATE	RESISTANT	NOT_FOUND	INTERMEDIATE	folP_R228SgyrA_D95NgyrA_S91FparC_S87RparC_S88PpenA_G545SpenA_I312MpenA_V316TponA1_L421PrpsJ_V57M	0	6
23GB000031.fasta	23GB000031	SAMEA117567977	PRJEB83897	28	M	Viken	East	Sekret	Urethra	2022-12-22	0,5	0,016	0,008	0,016	0,25	32	2	NA	Negativ	1599	10	6	65aa8693ab2	NOT_FOUND	NOT_FOUND	NOT_FOUND	NOT_FOUND	RESISTANT	RESISTANT	NOT_FOUND	RESISTANT	folP_R228SmtrR_A39TmtrR_promoter_a-57delpenA_ins346DporB1b_A121DporB1b_G120KrpsJ_V57M	0	6
23GB000413.fasta	23GB000413	SAMEA117568148	PRJEB83897	21	M	Oslo	East	Sekret	Urethra	2023-02-08	0,5	0,016	0,016	0,016	0,25	16	2	NA	Negativ	1599	10	6	65aa8693ab2	NOT_FOUND	NOT_FOUND	NOT_FOUND	NOT_FOUND	RESISTANT	RESISTANT	NOT_FOUND	RESISTANT	folP_R228SmtrR_A39TmtrR_promoter_a-57delpenA_ins346DporB1b_A121DporB1b_G120KrpsJ_V57M	0	6
23GB000693.fasta	23GB000693	SAMEA117568281	PRJEB83897	38	M	Oslo	East	Sekret	Anus	2023-03-13	0,25	0,016	0,002	0,008	0,25	32	0,5	NA	Negativ	1599	10	6	65aa8693ab2	NOT_FOUND	NOT_FOUND	NOT_FOUND	NOT_FOUND	RESISTANT	RESISTANT	NOT_FOUND	RESISTANT	folP_R228SmtrR_A39TmtrR_promoter_a-57delpenA_ins346DporB1b_A121DporB1b_G120KrpsJ_V57M	0	6
23GB000705.fasta	23GB000705	SAMEA117568287	PRJEB83897	19	M	Oslo	East	Sekret	Hals	2023-03-16	0,25	0,016	0,008	0,016	0,25	32	1	NA	Negativ	1599	10	6	65aa8693ab2	NOT_FOUND	NOT_FOUND	NOT_FOUND	NOT_FOUND	RESISTANT	RESISTANT	NOT_FOUND	RESISTANT	folP_R228SmtrR_A39TmtrR_promoter_a-57delpenA_ins346DporB1b_A121DporB1b_G120KrpsJ_V57M	0	6
590785.fasta	590785	SAMEA5608870	PRJEB32435	28	M	Oslo	East	Sekret	Urethra	2017-06-22	0,25	0,016	0,008	4	1	8	0,5	NA	NA	1901	11	11	2654	NOT_FOUND	NOT_FOUND	NOT_FOUND	RESISTANT	INTERMEDIATE	RESISTANT	NOT_FOUND	INTERMEDIATE	folP_R228SmtrR_A39TparC_S87RpenA_G542SpenA_ins346DponA1_L421PrpsJ_V57M	0	6
664262.fasta	664262	SAMEA6534250	PRJEB32435	33	M	Oslo	East	Sekret		2019-05-20	0,25	0,016	0,004	0,004	0,25	16	0,25	4	Ikke påvist	8156	3	12	4344	NOT_FOUND	NOT_FOUND	NOT_FOUND	NOT_FOUND	INTERMEDIATE	RESISTANT	NOT_FOUND	NOT_FOUND	folP_R228SmtrR_A39TpenA_ins346DponA1_L421P	0	6
22GB000983.fasta	22GB000983	SAMEA117567568	PRJEB83897	23	M	Oslo	East	Sekret	Urethra	2022-08-17	0,125	0,016	0,016	0,004	0,25	16	0,5	8	Negativ	1584	3	12	5382	NOT_FOUND	NOT_FOUND	NOT_FOUND	NOT_FOUND	INTERMEDIATE	RESISTANT	NOT_FOUND	INTERMEDIATE	folP_R228SmtrR_A39TpenA_ins346DponA1_L421PrpsJ_V57M	0	6
22GB001062.fasta	22GB001062	SAMEA117567620	PRJEB83897	52	M	Viken	East	Sekret	Anus	2022-08-29	0,25	0,016	0,016	0,002	0,25	16	0,5	8	Negativ	1584	3	12	5382	NOT_FOUND	NOT_FOUND	NOT_FOUND	NOT_FOUND	INTERMEDIATE	RESISTANT	NOT_FOUND	INTERMEDIATE	folP_R228SmtrR_A39TpenA_ins346DponA1_L421PrpsJ_V57M	0	6
530615.fasta	530615	SAMEA5608262	PRJEB32435	27	M	Troms og Finnmark	North	Sekret	Ureter	2016-03-10	0,125	0,016	0,002	0,002	0,125	8	0,5	NA	NA	11999	1	18	245	NOT_FOUND	NOT_FOUND	NOT_FOUND	NOT_FOUND	INTERMEDIATE	RESISTANT	NOT_FOUND	INTERMEDIATE	folP_R228SmtrR_A39TpenA_ins346DponA1_L421PrpsJ_V57M	0	6
553718.fasta	553718	SAMEA5608426	PRJEB32435	22	F	Viken	East	Sekret	Cervix uter	2016-03-31	0,25	0,016	0,004	0,004	0,125	8	1	NA	NA	11999	1	18	245	NOT_FOUND	NOT_FOUND	NOT_FOUND	NOT_FOUND	INTERMEDIATE	RESISTANT	NOT_FOUND	INTERMEDIATE	folP_R228SmtrR_A39TpenA_ins346DponA1_L421PrpsJ_V57M	0	6
553832.fasta	553832	SAMEA5608443	PRJEB32435	22	M	Oslo	East	Sekret	Urethra	2016-05-09	0,25	0,016	0,016	0,004	0,25	16	1	NA	NA	11999	1	18	245	NOT_FOUND	NOT_FOUND	NOT_FOUND	NOT_FOUND	INTERMEDIATE	RESISTANT	NOT_FOUND	INTERMEDIATE	folP_R228SmtrR_A39TpenA_ins346DponA1_L421PrpsJ_V57M	0	6
592483.fasta	592483	SAMEA5608907	PRJEB32435	23	M	Viken	East	Sekret	Hals	2017-07-11	0,25	0,016	0,004	0,004	0,25	16	1	NA	NA	11999	1	18	245	NOT_FOUND	NOT_FOUND	NOT_FOUND	NOT_FOUND	INTERMEDIATE	RESISTANT	NOT_FOUND	INTERMEDIATE	folP_R228SmtrR_A39TpenA_ins346DponA1_L421PrpsJ_V57M	0	6
593794.fasta	593794	SAMEA5608957	PRJEB32435	14	M	Møre og Romsdal	West	Sekret	Anus	2017-07-27	0,25	0,016	0,002	0,002	0,25	8	0,5	NA	NA	11999	1	18	245	NOT_FOUND	NOT_FOUND	NOT_FOUND	NOT_FOUND	INTERMEDIATE	RESISTANT	NOT_FOUND	INTERMEDIATE	folP_R228SmtrR_A39TpenA_ins346DponA1_L421PrpsJ_V57M	0	6
635424.fasta	635424	SAMEA6533721	PRJEB32435	30	M	NA	NA	SEKRET	URETRA	2018-07-31	NA	NA	NA	NA	NA	NA	NA	NA	NA	1580	1	18	245	NOT_FOUND	NOT_FOUND	NOT_FOUND	NOT_FOUND	INTERMEDIATE	RESISTANT	NOT_FOUND	INTERMEDIATE	folP_R228SmtrR_A39TpenA_ins346DponA1_L421PrpsJ_V57M	0	6
20GB000108.fasta	20GB000108	SAMEA117566538	PRJEB83897	34	M	Oslo	East	Puss	Rektum	2020-01-06	0,5	0,016	0,016	0,016	0,5	16	2	8	Ikke påvist	1580	1	18	883	NOT_FOUND	NOT_FOUND	NOT_FOUND	NOT_FOUND	RESISTANT	RESISTANT	NOT_FOUND	INTERMEDIATE	folP_R228SmtrR_A39TpenA_ins346DporB1b_A121DporB1b_G120KrpsJ_V57M	0	6
20GB000162.fasta	20GB000162	SAMEA117566559	PRJEB83897	28	M	Oslo	East	Sekret	Anus	2019-12-11	0,25	0,016	0,008	0,008	0,25	16	1	8	Ikke påvist	1580	1	18	883	NOT_FOUND	NOT_FOUND	NOT_FOUND	NOT_FOUND	RESISTANT	RESISTANT	NOT_FOUND	INTERMEDIATE	folP_R228SmtrR_A39TpenA_ins346DporB1b_A121DporB1b_G120KrpsJ_V57M	0	6
20GB000291.fasta	20GB000291	SAMEA117566598	PRJEB83897	31	M	Troms og Finnmark	North	Sekret	Hals	2020-01-20	0,25	0,016	0,016	0,016	0,5	16	2	8	Ikke påvist	1580	1	18	883	NOT_FOUND	NOT_FOUND	NOT_FOUND	NOT_FOUND	RESISTANT	RESISTANT	NOT_FOUND	INTERMEDIATE	folP_R228SmtrR_A39TpenA_ins346DporB1b_A121DporB1b_G120KrpsJ_V57M	0	6
20GB000462.fasta	20GB000462	SAMEA117566642	PRJEB83897	23	M	Oslo	East	Sekret	Anus	2020-02-06	0,25	0,016	0,016	0,008	0,25	16	0,5	NA	Ikke påvist	1580	1	18	883	NOT_FOUND	NOT_FOUND	NOT_FOUND	NOT_FOUND	RESISTANT	RESISTANT	NOT_FOUND	INTERMEDIATE	folP_R228SmtrR_A39TpenA_ins346DporB1b_A121DporB1b_G120KrpsJ_V57M	0	6
20GB000589.fasta	20GB000589	SAMEA117566674	PRJEB83897	23	M	Viken	East	Sekret	Anus	2020-02-20	0,25	0,016	0,016	0,008	0,25	16	0,5	NA	Ikke påvist	1580	1	18	883	NOT_FOUND	NOT_FOUND	NOT_FOUND	NOT_FOUND	RESISTANT	RESISTANT	NOT_FOUND	INTERMEDIATE	folP_R228SmtrR_A39TpenA_ins346DporB1b_A121DporB1b_G120KrpsJ_V57M	0	6
20GB000892.fasta	20GB000892	SAMEA117566767	PRJEB83897	35	M	Oslo	East	Sekret		2020-05-06	0,25	0,016	0,004	0,008	0,5	8	0,5	NA	Ikke påvist	1580	1	18	883	NOT_FOUND	NOT_FOUND	NOT_FOUND	NOT_FOUND	RESISTANT	RESISTANT	NOT_FOUND	INTERMEDIATE	folP_R228SmtrR_A39TpenA_ins346DporB1b_A121DporB1b_G120KrpsJ_V57M	0	6
20GB001151.fasta	20GB001151	SAMEA117566862	PRJEB83897	36	M	Troms og Finnmark	North	Sekret	Urethra	2020-08-12	0,25	0,016	0,016	0,016	0,25	16	1	NA	Ikke påvist	1580	1	18	883	NOT_FOUND	NOT_FOUND	NOT_FOUND	NOT_FOUND	RESISTANT	RESISTANT	NOT_FOUND	INTERMEDIATE	folP_R228SmtrR_A39TpenA_ins346DporB1b_A121DporB1b_G120KrpsJ_V57M	0	6
20GB001302.fasta	20GB001302	SAMEA117566935	PRJEB83897	20	M	Nordland	North	Sekret	Urethra	2020-10-20	0,125	0,016	0,016	0,008	0,25	8	4	NA	Ikke påvist	1580	1	18	883	NOT_FOUND	NOT_FOUND	NOT_FOUND	NOT_FOUND	RESISTANT	RESISTANT	NOT_FOUND	INTERMEDIATE	folP_R228SmtrR_A39TpenA_ins346DporB1b_A121DporB1b_G120KrpsJ_V57M	0	6
20GB001320.fasta	20GB001320	SAMEA117566945	PRJEB83897	30	M	Oslo	East	Sekret	Anus	2020-10-26	0,25	0,016	0,002	0,008	0,125	8	0,5	NA	Ikke påvist	1580	1	18	883	NOT_FOUND	NOT_FOUND	NOT_FOUND	NOT_FOUND	RESISTANT	RESISTANT	NOT_FOUND	INTERMEDIATE	folP_R228SmtrR_A39TpenA_ins346DporB1b_A121DporB1b_G120KrpsJ_V57M	0	6
20GB001343.fasta	20GB001343	SAMEA117566952	PRJEB83897	30	M	Oslo	East	Sekret	Urethra	2020-11-05	0,25	0,016	0,004	0,008	0,25	16	0,5	NA	Ikke påvist	1580	1	18	883	NOT_FOUND	NOT_FOUND	NOT_FOUND	NOT_FOUND	RESISTANT	RESISTANT	NOT_FOUND	INTERMEDIATE	folP_R228SmtrR_A39TpenA_ins346DporB1b_A121DporB1b_G120KrpsJ_V57M	0	6
20GB001348.fasta	20GB001348	SAMEA117566955	PRJEB83897	31	M	Oslo	East	Sekret	Anus	2020-11-06	0,25	0,016	0,016	0,008	0,25	16	1	NA	Ikke påvist	1580	1	18	883	NOT_FOUND	NOT_FOUND	NOT_FOUND	NOT_FOUND	RESISTANT	RESISTANT	NOT_FOUND	INTERMEDIATE	folP_R228SmtrR_A39TpenA_ins346DporB1b_A121DporB1b_G120KrpsJ_V57M	0	6
20GB001353.fasta	20GB001353	SAMEA117566959	PRJEB83897	33	M	Oslo	East	Sekret	Anus	2020-11-09	0,25	0,016	0,004	0,016	0,25	16	0,5	NA	Ikke påvist	1580	1	18	883	NOT_FOUND	NOT_FOUND	NOT_FOUND	NOT_FOUND	RESISTANT	RESISTANT	NOT_FOUND	INTERMEDIATE	folP_R228SmtrR_A39TpenA_ins346DporB1b_A121DporB1b_G120KrpsJ_V57M	0	6
20GB001390.fasta	20GB001390	SAMEA117566977	PRJEB83897	28	M	Oslo	East	Sekret	Anus	2020-11-23	0,25	0,016	0,004	0,008	0,25	16	0,5	NA	Ikke påvist	1580	1	18	883	NOT_FOUND	NOT_FOUND	NOT_FOUND	NOT_FOUND	RESISTANT	RESISTANT	NOT_FOUND	INTERMEDIATE	folP_R228SmtrR_A39TpenA_ins346DporB1b_A121DporB1b_G120KrpsJ_V57M	0	6
22GB001102.fasta	22GB001102	SAMEA117567647	PRJEB83897	43	M	Oslo	East	Sekret	Anus	2022-09-05	0,25	0,016	0,016	0,016	0,25	16	1	8	Negativ	1599	10	6	3074	NOT_FOUND	NOT_FOUND	NOT_FOUND	NOT_FOUND	RESISTANT	RESISTANT	NOT_FOUND	INTERMEDIATE	folP_R228SmtrR_A39TpenA_ins346DporB1b_A121DporB1b_G120KrpsJ_V57M	0	6
22GB001242.fasta	22GB001242	SAMEA117567717	PRJEB83897	46	M	Oslo	East	Sekret	Anus	2022-09-30	0,064	0,016	0,002	0,004	0,125	32	0,5	8	Negativ	1599	10	6	3074	NOT_FOUND	NOT_FOUND	NOT_FOUND	NOT_FOUND	RESISTANT	RESISTANT	NOT_FOUND	INTERMEDIATE	folP_R228SmtrR_A39TpenA_ins346DporB1b_A121DporB1b_G120KrpsJ_V57M	0	6
22GB001254.fasta	22GB001254	SAMEA117567720	PRJEB83897	36	M	Oslo	East	Sekret	Urethra	2022-10-03	0,125	0,016	0,004	0,008	0,125	32	0,5	8	Negativ	1599	10	6	3074	NOT_FOUND	NOT_FOUND	NOT_FOUND	NOT_FOUND	RESISTANT	RESISTANT	NOT_FOUND	INTERMEDIATE	folP_R228SmtrR_A39TpenA_ins346DporB1b_A121DporB1b_G120KrpsJ_V57M	0	6
22GB001394.fasta	22GB001394	SAMEA117567797	PRJEB83897	38	M	Viken	East	Sekret	Anus	2022-10-24	0,25	0,016	0,016	0,008	0,25	16	2	8	Negativ	1599	10	6	3074	NOT_FOUND	NOT_FOUND	NOT_FOUND	NOT_FOUND	RESISTANT	RESISTANT	NOT_FOUND	INTERMEDIATE	folP_R228SmtrR_A39TpenA_ins346DporB1b_A121DporB1b_G120KrpsJ_V57M	0	6
676448.fasta	676448	SAMEA117568452	PRJEB83897	22	F	Trøndelag	North	Sekret	Cervix uter	2019-11-01	0,125	0,016	0,016	16	0,25	16	8	8	Ikke påvist	1580	1	18	883	NOT_FOUND	NOT_FOUND	NOT_FOUND	NOT_FOUND	RESISTANT	RESISTANT	NOT_FOUND	INTERMEDIATE	folP_R228SmtrR_A39TpenA_ins346DporB1b_A121DporB1b_G120KrpsJ_V57M	0	6
676791.fasta	676791	SAMEA6534682	PRJEB32435	30	M	Oslo	East	Sekret	Anus	2019-11-06	0,25	0,016	0,008	0,008	0,5	16	0,5	8	Ikke påvist	1580	1	18	883	NOT_FOUND	NOT_FOUND	NOT_FOUND	NOT_FOUND	RESISTANT	RESISTANT	NOT_FOUND	INTERMEDIATE	folP_R228SmtrR_A39TpenA_ins346DporB1b_A121DporB1b_G120KrpsJ_V57M	0	6
677778.fasta	677778	SAMEA6534705	PRJEB32435	30	M	Oslo	East	Sekret	Anus	2019-11-18	0,125	0,016	0,008	0,008	0,25	8	0,5	8	Ikke påvist	1580	1	18	883	NOT_FOUND	NOT_FOUND	NOT_FOUND	NOT_FOUND	RESISTANT	RESISTANT	NOT_FOUND	INTERMEDIATE	folP_R228SmtrR_A39TpenA_ins346DporB1b_A121DporB1b_G120KrpsJ_V57M	0	6
677904.fasta	677904	SAMEA6534706	PRJEB32435	52	M	Oslo	East	Sekret	Urethra	2019-11-19	0,25	0,016	0,016	0,016	0,25	8	1	8	Ikke påvist	1580	1	18	883	NOT_FOUND	NOT_FOUND	NOT_FOUND	NOT_FOUND	RESISTANT	RESISTANT	NOT_FOUND	INTERMEDIATE	folP_R228SmtrR_A39TpenA_ins346DporB1b_A121DporB1b_G120KrpsJ_V57M	0	6
678696.fasta	678696	SAMEA6534732	PRJEB32435	25	M	Trøndelag	North	Sekret	Hals	2019-11-26	0,25	0,016	0,008	0,008	0,25	8	1	8	Ikke påvist	1580	1	18	883	NOT_FOUND	NOT_FOUND	NOT_FOUND	NOT_FOUND	RESISTANT	RESISTANT	NOT_FOUND	INTERMEDIATE	folP_R228SmtrR_A39TpenA_ins346DporB1b_A121DporB1b_G120KrpsJ_V57M	0	6
679276.fasta	679276	SAMEA6534740	PRJEB32435	34	M	Oslo	East	Sekret	Anus	2019-12-02	0,25	0,016	0,008	0,016	0,25	16	0,5	4	Ikke påvist	1580	1	18	883	NOT_FOUND	NOT_FOUND	NOT_FOUND	NOT_FOUND	RESISTANT	RESISTANT	NOT_FOUND	INTERMEDIATE	folP_R228SmtrR_A39TpenA_ins346DporB1b_A121DporB1b_G120KrpsJ_V57M	0	6
679702.fasta	679702	SAMEA117568456	PRJEB83897	23	M	Oslo	East	Sekret		2019-12-04	0,125	0,016	0,008	0,008	0,25	16	0,25	8	Ikke påvist	1580	1	18	883	NOT_FOUND	NOT_FOUND	NOT_FOUND	NOT_FOUND	RESISTANT	RESISTANT	NOT_FOUND	INTERMEDIATE	folP_R228SmtrR_A39TpenA_ins346DporB1b_A121DporB1b_G120KrpsJ_V57M	0	6
20GB000301.fasta	20GB000301	SAMEA117566606	PRJEB83897	27	M	Viken	East	Sekret	Urethra	2020-01-23	0,25	0,016	0,004	0,008	0,25	16	0,5	NA	Ikke påvist	1599	10	6	520	NOT_FOUND	NOT_FOUND	NOT_FOUND	NOT_FOUND	INTERMEDIATE	RESISTANT	NOT_FOUND	INTERMEDIATE	folP_R228SmtrR_A39TpenA_ins346DrpsJ_V57M	0	6
20GB000388.fasta	20GB000388	SAMEA117566630	PRJEB83897	36	M	Oslo	East	Sekret	Anus	2020-01-31	0,125	0,016	0,008	0,008	0,25	16	0,5	NA	Ikke påvist	1599	10	6	520	NOT_FOUND	NOT_FOUND	NOT_FOUND	NOT_FOUND	INTERMEDIATE	RESISTANT	NOT_FOUND	INTERMEDIATE	folP_R228SmtrR_A39TpenA_ins346DrpsJ_V57M	0	6
20GB000976.fasta	20GB000976	SAMEA117566782	PRJEB83897	41	M	Viken	East	Sekret	Rektum	2020-06-02	0,25	0,016	0,016	0,004	0,25	16	1	NA	Ikke påvist	1599	10	6	520	NOT_FOUND	NOT_FOUND	NOT_FOUND	NOT_FOUND	INTERMEDIATE	RESISTANT	NOT_FOUND	INTERMEDIATE	folP_R228SmtrR_A39TpenA_ins346DrpsJ_V57M	0	6
22GB001029.fasta	22GB001029	SAMEA117567598	PRJEB83897	30	M	Oslo	East	Sekret	Anus	2022-08-24	0,5	0,016	0,004	0,002	0,125	16	0,25	8	Negativ	1599	10	6	520	NOT_FOUND	NOT_FOUND	NOT_FOUND	NOT_FOUND	INTERMEDIATE	RESISTANT	NOT_FOUND	INTERMEDIATE	folP_R228SmtrR_A39TpenA_ins346DrpsJ_V57M	0	6
22GB001177.fasta	22GB001177	SAMEA117567684	PRJEB83897	21	M	Vestland	West	Sekret	Urethra	2022-09-19	0,25	0,016	0,016	0,004	0,125	16	32	16	Negativ	1599	10	6	520	NOT_FOUND	NOT_FOUND	NOT_FOUND	NOT_FOUND	INTERMEDIATE	RESISTANT	NOT_FOUND	INTERMEDIATE	folP_R228SmtrR_A39TpenA_ins346DrpsJ_V57M	0	6
22GB001244.fasta	22GB001244	SAMEA117567719	PRJEB83897	23	M	Viken	East	Sekret	Urethra	2022-09-29	0,125	0,016	0,002	0,002	0,125	16	0,125	4	Negativ	1580	1	18	4597	NOT_FOUND	NOT_FOUND	NOT_FOUND	NOT_FOUND	INTERMEDIATE	RESISTANT	NOT_FOUND	INTERMEDIATE	folP_R228SmtrR_A39TpenA_ins346DrpsJ_V57M	0	6
22GB001388.fasta	22GB001388	SAMEA117567796	PRJEB83897	53	M	Viken	East	Sekret	Anus	2022-10-19	0,125	0,016	0,002	0,002	0,125	16	0,25	4	Negativ	1580	1	18	4597	NOT_FOUND	NOT_FOUND	NOT_FOUND	NOT_FOUND	INTERMEDIATE	RESISTANT	NOT_FOUND	INTERMEDIATE	folP_R228SmtrR_A39TpenA_ins346DrpsJ_V57M	0	6
22GB001633.fasta	22GB001633	SAMEA117567920	PRJEB83897	23	M	Oslo	East	Sekret	Urethra	2022-12-02	0,5	0,016	0,016	0,004	0,25	16	1	8	Negativ	-	1	18	63	NOT_FOUND	NOT_FOUND	NOT_FOUND	NOT_FOUND	INTERMEDIATE	RESISTANT	NOT_FOUND	INTERMEDIATE	folP_R228SmtrR_A39TpenA_ins346DrpsJ_V57M	0	6
22GB001681.fasta	22GB001681	SAMEA117567936	PRJEB83897	18	F	Oslo	East	Sekret	Vagina	2022-12-07	0,125	0,016	0,004	0,004	0,5	32	0,5	8	Negativ	-	1	18	63	NOT_FOUND	NOT_FOUND	NOT_FOUND	NOT_FOUND	INTERMEDIATE	RESISTANT	NOT_FOUND	INTERMEDIATE	folP_R228SmtrR_A39TpenA_ins346DrpsJ_V57M	0	6
22GB001682.fasta	22GB001682	SAMEA117567937	PRJEB83897	18	F	Oslo	East	Sekret	Cervix uter	2022-12-08	0,064	0,016	0,002	0,004	0,5	32	0,5	8	Negativ	-	1	18	63	NOT_FOUND	NOT_FOUND	NOT_FOUND	NOT_FOUND	INTERMEDIATE	RESISTANT	NOT_FOUND	INTERMEDIATE	folP_R228SmtrR_A39TpenA_ins346DrpsJ_V57M	0	6
23GB000040.fasta	23GB000040	SAMEA117567978	PRJEB83897	22	M	Oslo	East	Sekret	Urethra	2022-12-27	0,5	0,016	0,016	0,004	0,5	16	1	NA	Negativ	-	1	18	63	NOT_FOUND	NOT_FOUND	NOT_FOUND	NOT_FOUND	INTERMEDIATE	RESISTANT	NOT_FOUND	INTERMEDIATE	folP_R228SmtrR_A39TpenA_ins346DrpsJ_V57M	0	6
23GB000302.fasta	23GB000302	SAMEA117568091	PRJEB83897	24	F	Nordland	North	Sekret	Vagina	2023-01-27	0,25	0,016	0,016	0,004	0,25	16	0,5	NA	Negativ	-	1	18	63	NOT_FOUND	NOT_FOUND	NOT_FOUND	NOT_FOUND	INTERMEDIATE	RESISTANT	NOT_FOUND	INTERMEDIATE	folP_R228SmtrR_A39TpenA_ins346DrpsJ_V57M	0	6
23GB000703.fasta	23GB000703	SAMEA117568285	PRJEB83897	21	F	Vestfold og Telemark	East	Sekret	Cervix uter	2023-03-15	0,5	0,016	0,004	0,004	0,5	32	0,25	NA	Negativ	-	1	18	63	NOT_FOUND	NOT_FOUND	NOT_FOUND	NOT_FOUND	INTERMEDIATE	RESISTANT	NOT_FOUND	INTERMEDIATE	folP_R228SmtrR_A39TpenA_ins346DrpsJ_V57M	0	6
23GB000847.fasta	23GB000847	SAMEA117568351	PRJEB83897	24	F	Nordland	North	Sekret	Cervix uter	2023-04-04	0,25	0,016	0,016	0,004	0,25	16	0,5	NA	Negativ	-	1	18	63	NOT_FOUND	NOT_FOUND	NOT_FOUND	NOT_FOUND	INTERMEDIATE	RESISTANT	NOT_FOUND	INTERMEDIATE	folP_R228SmtrR_A39TpenA_ins346DrpsJ_V57M	0	6
554797.fasta	554797	SAMEA5608480	PRJEB32435	25	M	Innlandet	East	Sekret	Anus	2016-07-29	0,125	0,016	0,008	0,004	0,25	8	0,5	NA	NA	1893	Other	22	1958	NOT_FOUND	NOT_FOUND	NOT_FOUND	NOT_FOUND	INTERMEDIATE	RESISTANT	NOT_FOUND	INTERMEDIATE	folP_R228SmtrR_A39TpenA_ins346DrpsJ_V57M	0	6
556567.fasta	556567	SAMEA5608528	PRJEB32435	23	M	Nordland	North	Sekret		2016-12-07	0,25	0,016	0,004	0,004	0,25	16	0,5	NA	NA	1599	10	6	520	NOT_FOUND	NOT_FOUND	NOT_FOUND	NOT_FOUND	INTERMEDIATE	RESISTANT	NOT_FOUND	INTERMEDIATE	folP_R228SmtrR_A39TpenA_ins346DrpsJ_V57M	0	6
562691.fasta	562691	SAMEA5608596	PRJEB32435	37	M	Oslo	East	Sekret	Urethra	2017-01-17	0,25	0,016	0,004	0,004	0,25	16	1	NA	NA	1599	10	6	520	NOT_FOUND	NOT_FOUND	NOT_FOUND	NOT_FOUND	INTERMEDIATE	RESISTANT	NOT_FOUND	INTERMEDIATE	folP_R228SmtrR_A39TpenA_ins346DrpsJ_V57M	0	6
566481.fasta	566481	SAMEA5608657	PRJEB32435	34	M	Viken	East	Sekret	Urethra	2017-02-13	0,25	0,016	0,004	0,004	0,125	16	1	NA	NA	1599	10	6	520	NOT_FOUND	NOT_FOUND	NOT_FOUND	NOT_FOUND	INTERMEDIATE	RESISTANT	NOT_FOUND	INTERMEDIATE	folP_R228SmtrR_A39TpenA_ins346DrpsJ_V57M	0	6
567063.fasta	567063	SAMEA5608667	PRJEB32435	32	M	Oslo	East	Sekret	Hals	2017-02-16	0,25	0,016	0,008	0,004	0,125	16	0,5	NA	NA	1599	10	6	520	NOT_FOUND	NOT_FOUND	NOT_FOUND	NOT_FOUND	INTERMEDIATE	RESISTANT	NOT_FOUND	INTERMEDIATE	folP_R228SmtrR_A39TpenA_ins346DrpsJ_V57M	0	6
567623.fasta	567623	SAMEA5608686	PRJEB32435	32	M	Oslo	East	Sekret	Urethra	2017-02-23	0,25	0,016	0,004	0,004	0,25	16	1	NA	NA	1599	10	6	520	NOT_FOUND	NOT_FOUND	NOT_FOUND	NOT_FOUND	INTERMEDIATE	RESISTANT	NOT_FOUND	INTERMEDIATE	folP_R228SmtrR_A39TpenA_ins346DrpsJ_V57M	0	6
568626.fasta	568626	SAMEA5608693	PRJEB32435	47	M	Oslo	East	Sekret	Urethra	2017-02-27	0,25	0,016	0,004	0,004	0,25	16	1	NA	NA	13347	10	6	520	NOT_FOUND	NOT_FOUND	NOT_FOUND	NOT_FOUND	INTERMEDIATE	RESISTANT	NOT_FOUND	INTERMEDIATE	folP_R228SmtrR_A39TpenA_ins346DrpsJ_V57M	0	6
571557.fasta	571557	SAMEA5608735	PRJEB32435	32	M	Oslo	East	Sekret	Anus	2017-03-15	0,25	0,016	0,004	0,008	0,25	16	1	NA	NA	1599	10	6	520	NOT_FOUND	NOT_FOUND	NOT_FOUND	NOT_FOUND	INTERMEDIATE	RESISTANT	NOT_FOUND	INTERMEDIATE	folP_R228SmtrR_A39TpenA_ins346DrpsJ_V57M	0	6
573089.fasta	573089	SAMEA5608748	PRJEB32435	35	M	Oslo	East	Sekret	Anus	2017-03-30	0,25	0,016	0,002	0,004	0,25	16	0,5	NA	NA	1599	10	6	520	NOT_FOUND	NOT_FOUND	NOT_FOUND	NOT_FOUND	INTERMEDIATE	RESISTANT	NOT_FOUND	INTERMEDIATE	folP_R228SmtrR_A39TpenA_ins346DrpsJ_V57M	0	6
573414.fasta	573414	SAMEA5608757	PRJEB32435	71	M	Oslo	East	Sekret		2017-04-05	0,5	0,016	0,004	0,004	0,25	16	1	NA	NA	1599	10	6	520	NOT_FOUND	NOT_FOUND	NOT_FOUND	NOT_FOUND	INTERMEDIATE	RESISTANT	NOT_FOUND	INTERMEDIATE	folP_R228SmtrR_A39TpenA_ins346DrpsJ_V57M	0	6
579032.fasta	579032	SAMEA5608842	PRJEB32435	34	M	Oslo	East	Sekret	Anus	2017-05-29	0,25	0,016	0,004	0,004	0,25	16	0,5	NA	NA	1599	10	6	520	NOT_FOUND	NOT_FOUND	NOT_FOUND	NOT_FOUND	INTERMEDIATE	RESISTANT	NOT_FOUND	INTERMEDIATE	folP_R228SmtrR_A39TpenA_ins346DrpsJ_V57M	0	6
592362.fasta	592362	SAMEA5608904	PRJEB32435	37	M	Oslo	East	Sekret	Anus	2017-07-10	0,25	0,016	0,004	0,004	0,25	16	1	NA	NA	1599	10	6	520	NOT_FOUND	NOT_FOUND	NOT_FOUND	NOT_FOUND	INTERMEDIATE	RESISTANT	NOT_FOUND	INTERMEDIATE	folP_R228SmtrR_A39TpenA_ins346DrpsJ_V57M	0	6
593245.fasta	593245	SAMEA5608943	PRJEB32435	31	M	Viken	East	Sekret	Urethra	2017-07-19	0,25	0,016	0,004	0,004	0,25	16	1	NA	NA	1599	10	6	520	NOT_FOUND	NOT_FOUND	NOT_FOUND	NOT_FOUND	INTERMEDIATE	RESISTANT	NOT_FOUND	INTERMEDIATE	folP_R228SmtrR_A39TpenA_ins346DrpsJ_V57M	0	6
600167.fasta	600167	SAMEA5609073	PRJEB32435	48	M	Oslo	East	Sekret	Anus	2017-09-25	0,25	0,016	0,004	0,004	0,25	16	1	NA	NA	1599	10	6	520	NOT_FOUND	NOT_FOUND	NOT_FOUND	NOT_FOUND	INTERMEDIATE	RESISTANT	NOT_FOUND	INTERMEDIATE	folP_R228SmtrR_A39TpenA_ins346DrpsJ_V57M	0	6
600294.fasta	600294	SAMEA5609074	PRJEB32435	63	M	Innlandet	East	Sekret	Kjønnsorga	2017-09-28	0,25	0,016	0,002	0,008	0,125	16	1	NA	NA	1599	10	6	520	NOT_FOUND	NOT_FOUND	NOT_FOUND	NOT_FOUND	INTERMEDIATE	RESISTANT	NOT_FOUND	INTERMEDIATE	folP_R228SmtrR_A39TpenA_ins346DrpsJ_V57M	0	6
605009.fasta	605009	SAMEA5609132	PRJEB32435	26	M	Rogaland	West	Sekret	Anus	2017-11-01	0,25	0,016	0,004	0,004	0,25	16	1	NA	NA	1599	10	6	520	NOT_FOUND	NOT_FOUND	NOT_FOUND	NOT_FOUND	INTERMEDIATE	RESISTANT	NOT_FOUND	INTERMEDIATE	folP_R228SmtrR_A39TpenA_ins346DrpsJ_V57M	0	6
605202.fasta	605202	SAMEA5609134	PRJEB32435	29	M	Oslo	East	Sekret	Anus	2017-11-08	0,25	0,016	0,004	0,004	0,25	16	1	NA	NA	1599	10	6	520	NOT_FOUND	NOT_FOUND	NOT_FOUND	NOT_FOUND	INTERMEDIATE	RESISTANT	NOT_FOUND	INTERMEDIATE	folP_R228SmtrR_A39TpenA_ins346DrpsJ_V57M	0	6
612311.fasta	612311	SAMEA6533430	PRJEB32435	64	M	Oslo	East	Sekret	Anus	2018-01-08	0,25	0,016	0,002	0,004	0,25	16	1	NA	NA	1599	10	6	520	NOT_FOUND	NOT_FOUND	NOT_FOUND	NOT_FOUND	INTERMEDIATE	RESISTANT	NOT_FOUND	INTERMEDIATE	folP_R228SmtrR_A39TpenA_ins346DrpsJ_V57M	0	6
617441.fasta	617441	SAMEA6533467	PRJEB32435	27	M	NA	NA	Sekret	Anus	2018-02-08	0,25	0,016	0,004	0,004	0,25	16	1	NA	NA	1599	10	6	520	NOT_FOUND	NOT_FOUND	NOT_FOUND	NOT_FOUND	INTERMEDIATE	RESISTANT	NOT_FOUND	INTERMEDIATE	folP_R228SmtrR_A39TpenA_ins346DrpsJ_V57M	0	6
619048.fasta	619048	SAMEA6963019	PRJEB32435	27	M	Rogaland	West	Sekret	Ureter	2018-02-21	0,25	0,016	0,004	0,004	0,25	16	1	NA	NA	1599	10	6	520	NOT_FOUND	NOT_FOUND	NOT_FOUND	NOT_FOUND	INTERMEDIATE	RESISTANT	NOT_FOUND	INTERMEDIATE	folP_R228SmtrR_A39TpenA_ins346DrpsJ_V57M	0	6
646005.fasta	646005	SAMEA6533961	PRJEB32435	33	M	NA	NA	SEKRET	URETRA	2018-11-30	NA	NA	NA	NA	NA	NA	NA	NA	NA	1599	10	6	520	NOT_FOUND	NOT_FOUND	NOT_FOUND	NOT_FOUND	INTERMEDIATE	RESISTANT	NOT_FOUND	INTERMEDIATE	folP_R228SmtrR_A39TpenA_ins346DrpsJ_V57M	0	6
665230.fasta	665230	SAMEA6534286	PRJEB32435	43	M	Vestland	West	Sekret	Urethra	2019-06-04	0,125	0,016	0,004	0,002	0,25	16	0,5	8	Ikke påvist	1599	10	6	520	NOT_FOUND	NOT_FOUND	NOT_FOUND	NOT_FOUND	INTERMEDIATE	RESISTANT	NOT_FOUND	INTERMEDIATE	folP_R228SmtrR_A39TpenA_ins346DrpsJ_V57M	0	6
666684.fasta	666684	SAMEA6534326	PRJEB32435	57	M	Oslo	East	Sekret	Anus	2019-06-20	0,25	0,016	0,002	0,004	0,25	16	0,5	8	Ikke påvist	1599	10	6	520	NOT_FOUND	NOT_FOUND	NOT_FOUND	NOT_FOUND	INTERMEDIATE	RESISTANT	NOT_FOUND	INTERMEDIATE	folP_R228SmtrR_A39TpenA_ins346DrpsJ_V57M	0	6
554801.fasta	554801	SAMEA5608482	PRJEB32435	21	M	Innlandet	East	Sekret	Hals	2016-07-29	0,125	0,016	0,008	0,004	0,25	8	0,5	NA	NA	1893	Other	22	c8a65ca76d1	NOT_FOUND	NOT_FOUND	NOT_FOUND	NOT_FOUND	NOT_FOUND	RESISTANT	NOT_FOUND	INTERMEDIATE	folP_R228SmtrR_A39TrpsJ_V57M	0	6
610074.fasta	610074	SAMEA5609154	PRJEB32435	38	M	Oslo	East	Sekret	Anus	2017-12-21	0,125	0,016	0,004	0,004	0,125	16	1	NA	NA	8145	Other	9	3651	NOT_FOUND	NOT_FOUND	NOT_FOUND	NOT_FOUND	INTERMEDIATE	RESISTANT	NOT_FOUND	INTERMEDIATE	folP_R228SmtrR_disruptedpenA_ins346DrpsJ_V57M	0	6
619378.fasta	619378	SAMEA6543552	PRJEB32435	43	M	Oslo	East	Sekret	Urethra	2018-02-22	0,25	0,032	0,016	2	0,5	16	1	NA	NA	7827	6	28	1225	NOT_FOUND	NOT_FOUND	NOT_FOUND	RESISTANT	INTERMEDIATE	RESISTANT	NOT_FOUND	RESISTANT	folP_R228SmtrR_G45DmtrR_promoter_a-57delparC_D86NpenA_A501VpenA_ins346DpenA_P551SponA1_L421PrpsJ_	0	6
661059.fasta	661059	SAMEA6534177	PRJEB32435	24	M	NA	NA	Sekret	Urethra	2019-04-15	Ikke utført	0,016	0,002	0,002	0,064	4	0,5	2	Ikke påvist	1583	12	24	181	NOT_FOUND	NOT_FOUND	NOT_FOUND	NOT_FOUND	INTERMEDIATE	RESISTANT	NOT_FOUND	RESISTANT	folP_R228SmtrR_G45DmtrR_promoter_a-57delpenA_ins346DponA1_L421PrpsJ_V57M	0	6
[illegible]																																		

629698.fasta	629698	SAMEA6533593	PRJEB32435	30	M	NA	NA	SEKRET	CERVIXUT	2018-05-25	NA	NA	NA	NA	NA	NA	NA	NA	NA	8156	3	12	[illegible]
629743.fasta	629743	SAMEA6533599	PRJEB32435	27	M	NA	NA	SEKRET	ANUS	2018-05-23	NA	NA	NA	NA	NA	NA	NA	NA	NA	8156	3	12	[illegible]
632719.fasta	632719	SAMEA6533635	PRJEB32435	29	M	NA	NA	SEKRET	ANUS	2018-06-26	NA	NA	NA	NA	NA	NA	NA	NA	NA	8156	3	12	[illegible]
632819.fasta	632819	SAMEA6533639	PRJEB32435	47	M	NA	NA	SEKRET	ANUS	2018-06-29	NA	NA	NA	NA	NA	NA	NA	NA	NA	8156	3	12	[illegible]
633579.fasta	633579	SAMEA6533659	PRJEB32435	29	M	NA	NA	SEKRET	ANUS	2018-07-04	NA	NA	NA	NA	NA	NA	NA	NA	NA	8156	3	12	[illegible]
634122.fasta	634122	SAMEA6533680	PRJEB32435	27	M	NA	NA	SEKRET	ANUS	2018-07-11	NA	NA	NA	NA	NA	NA	NA	NA	NA	8156	3	12	[illegible]
634200.fasta	634200	SAMEA6533687	PRJEB32435	45	M	NA	NA	SEKRET	URETRA	2018-07-13	NA	NA	NA	NA	NA	NA	NA	NA	NA	8156	3	12	[illegible]
634762.fasta	634762	SAMEA6533707	PRJEB32435	33	M	NA	NA	SEKRET	URETRA	2018-07-27	NA	NA	NA	NA	NA	NA	NA	NA	NA	8156	3	12	[illegible]
635249.fasta	635249	SAMEA6533716	PRJEB32435	26	M	NA	NA	SEKRET	URETRA	2018-07-30	NA	NA	NA	NA	NA	NA	NA	NA	NA	8156	3	12	[illegible]
636405.fasta	636405	SAMEA6533739	PRJEB32435	53	M	NA	NA	SEKRET	URETRA	2018-08-13	NA	NA	NA	NA	NA	NA	NA	NA	NA	8156	3	12	[illegible]
636548.fasta	636548	SAMEA6533754	PRJEB32435	26	M	NA	NA	SEKRET	URETRA	2018-08-08	NA	NA	NA	NA	NA	NA	NA	NA	NA	8156	3	12	[illegible]
636718.fasta	636718	SAMEA6533755	PRJEB32435	39	M	NA	NA	SEKRET	ANUS	2018-08-20	NA	NA	NA	NA	NA	NA	NA	NA	NA	8156	3	12	[illegible]
636721.fasta	636721	SAMEA6533756	PRJEB32435	24	M	NA	NA	SEKRET	URETRA	2018-08-20	NA	NA	NA	NA	NA	NA	NA	NA	NA	8156	3	12	[illegible]
636723.fasta	636723	SAMEA6533757	PRJEB32435	42	M	NA	NA	SEKRET	URETRA	2018-08-20	NA	NA	NA	NA	NA	NA	NA	NA	NA	8156	3	12	[illegible]
641183.fasta	641183	SAMEA6533873	PRJEB32435	67	M	NA	NA	SEKRET	URETRA	2018-09-26	0,064	0,016	0,016	0,002	NA	NA	NA	NA	NA	8156	3	12	[illegible]
644436.fasta	644436	SAMEA6533938	PRJEB32435	31	M	NA	NA	SEKRET	ANUS	2018-11-12	NA	NA	NA	NA	NA	NA	NA	NA	NA	8156	3	12	[illegible]
645498.fasta	645498	SAMEA6533950	PRJEB32435	24	M	NA	NA	SEKRET	URETRA	2018-11-21	NA	NA	NA	NA	NA	NA	NA	NA	NA	8156	3	12	[illegible]
646001.fasta	646001	SAMEA6533959	PRJEB32435	59	M	NA	NA	SEKRET	ANUS	2018-11-26	NA	NA	NA	NA	NA	NA	NA	NA	NA	8156	3	12	[illegible]
646007.fasta	646007	SAMEA6533962	PRJEB32435	82	M	NA	NA	SEKRET	URETRA	2018-11-30	NA	NA	NA	NA	NA	NA	NA	NA	NA	8156	3	12	[illegible]
646009.fasta	646009	SAMEA6533963	PRJEB32435	41	M	NA	NA	SEKRET	URETRA	2018-11-28	NA	NA	NA	NA	NA	NA	NA	NA	NA	8156	3	12	[illegible]
646011.fasta	646011	SAMEA6533964	PRJEB32435	67	M	NA	NA	SEKRET	URETRA	2018-11-26	NA	NA	NA	NA	NA	NA	NA	NA	NA	8156	3	12	[illegible]
647528.fasta	647528	SAMEA6533984	PRJEB32435	63	M	NA	NA	SEKRET	ANUS	2018-12-10	NA	NA	NA	NA	NA	NA	NA	NA	NA	8156	3	12	[illegible]
647719.fasta	647719	SAMEA6533990	PRJEB32435	35	M	NA	NA	SEKRET	URETRA	2018-12-12	NA	NA	NA	NA	NA	NA	NA	NA	NA	8156	3	12	[illegible]
650169.fasta	650169	SAMEA6534010	PRJEB32435	50	M	Oslo	East	Sekret	Anus	2019-01-10	0,25	0,016	0,002	0,004	0,25	16	0,125	4	Ikke påvist	8156	3	12	[illegible]
651043.fasta	651043	SAMEA6534030	PRJEB32435	39	M	Oslo	East	Sekret	Urethra	2019-01-18	0,5	0,016	0,004	0,004	0,5	16	0,125	4	Ikke påvist	8156	3	12	[illegible]
651972.fasta	651972	SAMEA6534045	PRJEB32435	27	M	Oslo	East	Sekret	Anus	2019-01-25	0,25	0,016	0,004	0,004	0,25	16	0,25	4	Ikke påvist	8156	3	12	[illegible]
652607.fasta	652607	SAMEA6534055	PRJEB32435	39	M	Oslo	East	Sekret	Anus	2019-01-28	0,25	0,016	0,002	0,004	0,125	8	0,125	4	Ikke påvist	8156	3	12	[illegible]
654143.fasta	654143	SAMEA6534073	PRJEB32435	28	M	Oslo	East	Sekret	Anus	2019-02-15	0,5	0,016	0,002	0,004	0,25	16	0,25	8	Ikke påvist	8156	3	12	[illegible]
661051.fasta	661051	SAMEA6534176	PRJEB32435	26	M	Oslo	East	Sekret	Anus	2019-04-15	Ikke utført	0,016	0,002	0,004	0,25	16	0,25	4	Ikke påvist	8156	3	12	[illegible]
663545.fasta	663545	SAMEA6534232	PRJEB32435	42	M	Viken	East	Sekret	Anus	2019-05-14	0,5	0,016	0,002	0,002	0,25	8	0,125	4	Ikke påvist	8156	3	12	[illegible]
667177.fasta	667177	SAMEA6534342	PRJEB32435	38	M	Oslo	East	Sekret	Anus	2019-06-26	0,5	0,016	0,004	0,004	0,5	16	0,125	4	Ikke påvist	8156	3	12	[illegible]
667179.fasta	667179	SAMEA6534343	PRJEB32435	28	M	Vestland	West	Sekret	Anus	2019-06-25	1	0,016	0,002	0,004	0,25	16	0,25	4	Ikke påvist	8156	3	12	[illegible]
667412.fasta	667412	SAMEA6534348	PRJEB32435	62	M	Viken	East	Sekret	Anus	2019-07-02	NA	0,016	0,002	0,004	0,125	8	0,25	NA	Ikke påvist	8156	3	12	[illegible]
668351.fasta	668351	SAMEA6534464	PRJEB32435	30	M	Rogaland	West	Ikke angitt		2019-07-19	0,125	0,016	0,004	0,004	0,25	16	0,25	4	Ikke påvist	8156	3	12	[illegible]
669209.fasta	669209	SAMEA6534497	PRJEB32435	50	M	Oslo	East	Sekret	Urethra	2019-08-01	0,25	0,016	0,002	0,004	0,25	16	0,25	4	Ikke påvist	8156	3	12	[illegible]
670259.fasta	670259	SAMEA6534520	PRJEB32435	33	M	Nordland	North	Sekret	Urethra	2019-08-09	0,25	0,016	0,004	0,004	0,25	16	0,25	4	Ikke påvist	8156	3	12	[illegible]
670811.fasta	670811	SAMEA6534532	PRJEB32435	31	M	Oslo	East	Sekret	Urethra	2019-08-21	0,5	0,016	0,002	0,004	0,125	8	0,125	4	Ikke påvist	11182	3	12	[illegible]
670819.fasta	670819	SAMEA6534536	PRJEB32435	22	M	Viken	East	Sekret	Anus	2019-08-22	0,25	0,016	0,002	0,004	0,125	8	0,25	4	Ikke påvist	8156	3	12	[illegible]
670831.fasta	670831	SAMEA6534542	PRJEB32435	24	M	Viken	East	Sekret	Hals	2019-08-20	0,5	0,016	0,002	0,004	0,25	16	0,25	8	Ikke påvist	8156	3	12	[illegible]
670833.fasta	670833	SAMEA6534543	PRJEB32435	27	M	Oslo	East	Sekret	Anus	2019-08-20	0,5	0,016	0,004	0,004	0,5	16	0,5	8	Ikke påvist	1583	3	12	[illegible]
670835.fasta	670835	SAMEA6534544	PRJEB32435	21	M	NA	NA	Sekret	Hals	2019-08-22	0,5	0,016	0,002	0,004	0,25	8	0,25	4	Ikke påvist	8156	3	12	[illegible]
671733.fasta	671733	SAMEA6534581	PRJEB32435	40	M	Oslo	East	Sekret	Urethra	2019-09-02	1	0,016	0,002	0,004	0,5	16	0,25	8	Ikke påvist	8156	3	12	[illegible]
672099.fasta	672099	SAMEA6534587	PRJEB32435	22	M	Viken	East		Rektum	2019-08-29	0,25	0,016	0,016	0,004	0,25	16	0,25	8	Ikke påvist	8156	3	12	[illegible]
672938.fasta	672938	SAMEA6534596	PRJEB32435	54	M	Oslo	East	Sekret	Anus	2019-09-17	0,25	0,016	0,002	0,004	0,25	16	0,25	4	Ikke påvist	8156	3	12	[illegible]
672948.fasta	672948	SAMEA6534599	PRJEB32435	43	M	Oslo	East	Sekret	Hals	2019-09-16	0,25	0,016	0,002	0,004	0,125	16	0,125	4	Ikke påvist	8156	3	12	[illegible]
673121.fasta	673121	SAMEA6534602	PRJEB32435	25	M	Oslo	East	Sekret	Urethra	2019-09-18	0,5	0,016	0,002	0,004	0,25	16	0,125	8	Ikke påvist	8156	3	12	[illegible]
673125.fasta	673125	SAMEA6534604	PRJEB32435	27	M	Oslo	East	Sekret	Anus	2019-09-18	0,25	0,016	0,002	0,004	0,125	8	0,125	4	Ikke påvist	8156	3	12	[illegible]
673521.fasta	673521	SAMEA6534611	PRJEB32435	60	M	Oslo	East	Sekret	Urethra	2019-09-25	0,25	0,016	0,002	0,004	0,125	16	0,125	4	Ikke påvist	8156	3	12	[illegible]
673530.fasta	673530	SAMEA6534614	PRJEB32435	27	M	Viken	East	Sekret	Urethra	2019-09-26	0,25	0,016	0,002	0,004	0,125	16	0,125	4	Ikke påvist	8156	3	12	[illegible]
673532.fasta	673532	SAMEA6534615	PRJEB32435	35	M	Viken	East	Sekret	Hals	2019-09-23	0,125	0,016	0,002	0,004	0,125	8	0,125	4	Ikke påvist	8156	3	12	[illegible]
674261.fasta	674261	SAMEA6534630	PRJEB32435	31	M	Oslo	East	Sekret	Anus	2019-10-07	0,25	0,016	0,004	0,004	0,5	16	0,25	4	Ikke påvist	8156	3	12	[illegible]
674934.fasta	674934	SAMEA6534643	PRJEB32435	26	M	Oslo	East	Sekret	Urethra	2019-10-18	0,125	0,016	0,002	0,008	0,125	8	0,064	2	Ikke påvist	8156	3	12	[illegible]
674944.fasta	674944	SAMEA6534648	PRJEB32435	53	M	Oslo	East	Sekret	Anus	2019-10-16	0,25	0,016	0,002	0,004	0,25	16	0,125	4	Ikke påvist	8156	3	12	[illegible]
675789.fasta	675789	SAMEA6534654	PRJEB32435	22	M	Nordland	North	Sekret	Urethra	2019-10-24	0,25	0,016	0,016	0,004	0,5	8	0,25	8	Ikke påvist	14610	3	12	[illegible]
675997.fasta	675997	SAMEA6534659	PRJEB32435	39	M	Oslo	East	Sekret	Anus	2019-10-28	0,5	0,016	0,004	0,004	0,25	16	0,25	8	Ikke påvist	8156	3	12	[illegible]
676265.fasta	676265	SAMEA6534667	PRJEB32435	38	M	Oslo	East	Sekret	Urethra	2019-11-01	0,25	0,016	0,004	0,004	0,25	16	0,25	4	Ikke påvist	8156	3	12	[illegible]
676267.fasta	676267	SAMEA6534668	PRJEB32435	19	M	Oslo	East	Sekret	Anus	2019-10-30	0,125	0,016	0,004	0,002	0,25	16	0,125	4	Ikke påvist	8156	3	12	[illegible]
676338.fasta	676338	SAMEA6534671	PRJEB32435	20	F	Viken	East	Sekret	Vagina	2019-10-30	0,25	0,016	0,004	0,008	0,125	16	0,125	4	Ikke påvist	14610	3	12	[illegible]
676810.fasta	676810	SAMEA6534684	PRJEB32435	19	M	Oslo	East	Sekret	Hals	2019-11-06	0,25	0,016	0,002	0,004	0,125	8	0,125	8	Ikke påvist	8156	3	12	[illegible]
676999.fasta	676999	SAMEA6534686	PRJEB32435	25	M	Trøndelag	North	Sekret	Urethra	2019-11-09	0,5	0,016	0,016	0,004	0,064	8	0,25	8	Ikke påvist	8156	3	12	[illegible]
677362.fasta	677362	SAMEA6534697	PRJEB32435	41	M	Oslo	East	Sekret	Urethra	2019-11-15	0,25	0,016	0,004	0,004	0,25	16	0,25	8	Ikke påvist	8156	3	12	[illegible]
678134.fasta	678134	SAMEA6534711	PRJEB32435	42	M	Oslo	East	Sekret	Urethra	2019-11-19	0,25	0,016	0,004	0,004	0,25	16	0,25	4	Ikke påvist	8156	3	12	[illegible]
678137.fasta	678137	SAMEA6534712	PRJEB32435	51	M	Oslo	East	Sekret	Urethra	2019-11-21	0,125	0,016	0,004	0,004	0,25	16	0,064	4	Ikke påvist	8156	3	12	[illegible]
678207.fasta	678207	SAMEA6534714	PRJEB32435	29	M	Vestfold og Telemark	East	Sekret	Urethra	2019-11-19	0,25	0,016	0,002	0,004	0,125	8	0,25	8	Ikke påvist	8156	3	12	[illegible]
678441.fasta	678441	SAMEA6534717	PRJEB32435	20	M	Oslo	East	Sekret	Urethra	2019-11-26	0,25	0,016	0,004	0,004	0,25	16	0,25	4	Ikke påvist	8156	3	12	[illegible]
678570.fasta	678570	SAMEA6534724	PRJEB32435	23	M	Oslo	East	Sekret	Urethra	2019-11-28	0,25	0,016	0,008	0,008	0,25	8	0,125	4	Ikke påvist	15184	3	12	[illegible]
678912.fasta	678912	SAMEA6534738	PRJEB32435	42	M	Viken	East	Sekret	Anus	2019-11-29	0,25	0,016	0,008	0,008	0,25	16	0,125	4	Ikke påvist	8156	3	12	[illegible]
608857.fasta	608857	SAMEA6533413	PRJEB32435	32	M	Trøndelag	North	Sekret	Urethra	2017-12-05	0,5	0,016	0,004	0,016	0,25	16	0,5	NA	NA	8156	3	12	[illegible]
609610.fasta	609610	SAMEA5609151	PRJEB32435	34	M	Trøndelag	North	Sekret	Anus	2017-12-08	0,5	0,016	0,002	0,008	0,25	16	0,5	NA	NA	8156	3	12	[illegible]
20GB001229.fasta	20GB001229	SAMEA117566902	PRJEB83897	41	M	Nordland	North	Sekret	Urethra	2020-09-15	0,064	0,016	0,016	0,004	0,25	16	0,5	NA	Ikke påvist	8156	3	12	[illegible]
564721.fasta	564721	SAMEA5608630	PRJEB32435	34	M	Møre og Romsdal	West	Sekret	Urethra	2017-01-27	0,5	0,016	0,002	0,004	0,125	8	0,25	NA	NA	8156	3	12	[illegible]
601487.fasta	601487	SAMEA5609088	PRJEB32435	30	M	Oslo	East	Sekret	Urethra	2017-10-09	1	0,016	0,004	0,004	0,25	16	0,25	NA	NA	8156	3	12	[illegible]
615955.fasta	615955	SAMEA6533452	PRJEB32435	30	M	Oslo	East	Sekret	Urethra	2018-01-30	1	0,016	0,004	0,004	0,25	16	0,25	NA	NA	8156	3	12	[illegible]
20GB000225.fasta	20GB000225	SAMEA117566579	PRJEB83897	26	M	Oslo	East	Sekret	Urethra	2020-01-14	0,25	0,016	0,008	0,004	0,5	16	0,5	4	Ikke påvist	13536	3	12	[illegible]
21GB000366.fasta	21GB000366	SAMEA117567047	PRJEB83897	57	M	Oslo	East	Sekret	Urethra	2021-07-15	0,5	0,016	0,002	0,002	0,25	8	0,25	NA	Ikke påvist	15823	3	12	[illegible]
23GB000244.fasta	23GB000244	SAMEA117568061	PRJEB83897	27	M	Oslo	East	Sekret	Urethra	2023-01-18	0,125	0,016	0,004	0,002	0,25	8	0,25	NA	Negativ	1584	3	12	[illegible]
553751.fasta	553751	SAMEA5608435	PRJEB32435	53	M	Oslo	East	Sekret	Urethra	2016-04-25	0,5	0,016	0,008	0,004	0,25	16	0,5	NA	NA	11986	3	12	[illegible]
554799.fasta	554799	SAMEA5608481	PRJEB32435	51	M	Oslo	East	Sekret	Urethra	2016-07-29	0,5	0,016	0,004	0,004	0,25	8	1	NA	NA	13334	3	12	[illegible]
645053.fasta	645053	SAMEA6533946	PRJEB32435	33	F	NA	NA	SEKRET	CERVIXUT	2018-11-19	NA	NA	NA	NA	NA	NA	NA	NA	NA	13536	3	12	[illegible]
560982.fasta	560982	SAMEA5608576	PRJEB32435	22	M	Oslo	East	Sekret	Anus	2017-01-02	0,25	0,016	0,004	0,008	0,125	16	1	NA	NA	1584	Other	9	[illegible]
579424.fasta	579424	SAMEA5608848	PRJEB32435	24	F	Oslo	East	Sekret	Cervix uter	2017-06-02	0,125	0,016	0,004	0,004	0,5	16	0,25	NA	NA	10931	Other	32	[illegible]
554741.fasta	554741	SAMEA5608476	PRJEB32435	45	M	Oslo	East			2016-07-22	0,125	0,016	0,004	0,004	0,125	8	0,5	NA	NA	10622	Other	9	[illegible]
592898.fasta	592898	SAMEA5608923	PRJEB32435	40	M	Oslo	East	Sekret	Hals	2017-07-17	0,125	0,016	0,002	0,004	0,125	16	0,5	NA	NA	10622	Other	9	[illegible]
593781.fasta	593781	SAMEA5608953	PRJEB32435	23	M	Oslo	East	Sekret	Urethra	2017-07-11	0,5	0,016	0,002	0,002	2	8	0,5	NA	NA	12502	Other	9	[illegible]
605533.fasta	605533	SAMEA5609137	PRJEB32435	23	M	Vestland	West	Sekret	Urethra	2017-11-13	0,125	0,016	0,002	0,004	0,064	16	0,5	NA	NA	10622	Other	9	[illegible]
670002.fasta	670002	SAMEA117568449	PRJEB83897	33	F	NA	NA	Sekret	Hals	2019-08-08	0,25	0,016	0,002	0,004	0,125	16	0,5	4	Ikke påvist	10622	Other	9	[illegible]
677366.fasta	677366	SAMEA6534699	PRJEB32435	27	M	Oslo	East	Sekret	Anus	2019-11-15	0,125	0,016	0,004	0,004	1	8	0,5	4	Påvist	10317	Other	9	[illegible]
20GB001101.fasta	20GB001101	SAMEA117566838	PRJEB83897	40	M	Rogaland	West	Sekret	Urethra	2020-07-24	0,25	0,016	0,016	0,008	0,25	16	1	NA	Ikke påvist	1584	Other	9	[illegible]
20GB001102.fasta	20GB001102	SAMEA117566839	PRJEB83897	30	M	Rogaland	West	Sekret	Anus	2020-07-24	0,5	0,016	0,016	0,008	0,25	16	1	NA	Ikke påvist	1584	Other	9	[illegible]
20GB001210.fasta	20GB001210	SAMEA117566891	PRJEB83897	49	M	Rogaland	West	Sekret	Urethra	2020-09-04	0,25	0,016	0,016	0,008	0,25	16	1	NA	Ikke påvist	1584	Other	9	[illegible]
20GB001356.fasta	20GB001356	SAMEA117566962	PRJEB83897	24	M	Rogaland	West	Sekret	Urethra	2020-11-11	0,25	0,016	0,016	0,008	0,25	16	1	NA	Ikke påvist	1584	Other	9	[illegible]
21GB000180.fasta	21GB000180	SAMEA117567020	PRJEB83897	29	M	Vestfold og Telemark	East	Sekret	Urethra	2021-04-17	0,25	0,016	0,016	0,008	0,25	16	1	NA	Ikke påvist	1584	Other	9	[illegible]
21GB000220.fasta	21GB000220	SAMEA117567028	PRJEB83897	31	M	Agder	South	Sekret	Urethra	2021-05-05	0,125	0,016	0,016	0,008	0,25	16	0,5	NA	Ikke påvist	1584	Other	9	[illegible]
21GB000221.fasta	21GB000221	SAMEA117567029	PRJEB83897	29	M	Agder	South	Sekret	Anus	2021-05-05	0,25	0,016	0,016	0,004	0,25	16	1	NA	Ikke påvist	1584	Other	9	[illegible]
21GB000259.fasta	21GB000259	SAMEA117567032	PRJEB83897	34	M	Agder	South	Sekret	Anus	2021-05-12	0,125	0,016	0,016	0,008	0,25	32	1	NA	Ikke påvist	1584	Other	9	[illegible]
21GB000320.fasta	21GB000320	SAMEA117567035	PRJEB83897	41	F	Rogaland	West	Sekret	Cervix uter	2021-06-09	0,25	0,016	0,016	0,004	0,25	16	1	NA	Ikke påvist	1584	Other	9	[illegible]
21GB000322.fasta	21GB000322	SAMEA117567036	PRJEB83897	50	M	Rogaland	West	Sekret	Urethra	2021-06-10	0,25	0,016	0,016	0,002	0,25	16	0,5	NA	Ikke påvist	1584	Other	9	[illegible]
21GB000325.fasta	21GB000325	SAMEA117567037	PRJEB83897	22	M	Rogaland	West	Sekret	Anus	2021-06-15	0,25	0,016	0,016	0,008	0,25	16	1	NA	Ikke påvist	1584	Other	9	[illegible]
21GB000378.fasta	21GB000378	SAMEA117567051	PRJEB83897	35	M	Vestland	West	Sekret	Urethra	2021-07-23	0,25	0,016	0,016	0,004	0,25	32	0,5	NA	Ikke påvist	1584	Other	9	[illegible]
21GB000399.fasta	21GB000399	SAMEA117567052	PRJEB83897	26	F	Rogaland	West	Sekret	Hals	2021-07-26	0,25	0,016	0,016	0,004	0,125	16	1	NA	Ikke påvist	1584	Other	9	[illegible]
22GB000040.fasta	22GB000040	SAMEA117567196	PRJEB83897	32	M	Rogaland	West	Sekret	Urethra	2022-01-05	0,25	0,016	0,016	0,004	0,125	16	0,5	NA	Ikke påvist	1584	Other	9	[illegible]
22GB000049.fasta	22GB000049	SAMEA117567201	PRJEB83897	31	F	Rogaland	West	Sekret	Cervix uter	2022-01-06	0,25	0,016	0,016	0,004	0,125	16	1	NA	Ikke påvist	1584	Other	9	[illegible]
515634.fasta	515634	SAMEA5608214	PRJEB32435	27	M	Nordland	North	Sekret	Urethra	2016-01-04	0,25	0,016	0,008	0,004	0,5	16	0,5	NA	NA	8122	Other	25	[illegible]
516308.fasta	516308	SAMEA5608219	PRJEB32435	22	M	Oslo	East	Sekret	Urethra	2016-01-07	0,25	0,016	0,004	0,004	0,25	16	0,5	NA	NA	8122	Other	25	[illegible]
516932.fasta	516932	SAMEA5608221	PRJEB32435	19	M	Oslo	East	Sekret	Urethra	2016-01-14	0,25	0,016	0,008	0,004	0,25	8	0,5	NA	NA	14333	Other	25	[illegible]
517052.fasta	517052	SAMEA5608222	PRJEB32435	15	F	Viken	East	Sekret	Glandula B	2016-01-19	0,064	0,25	0,016	32	0,25	8	0,5	NA	NA	8122	Other	25	[illegible]
518244.fasta	518244	SAMEA5608226	PRJEB32435	16	M	Viken	East	Sekret	Hals	2016-01-12	0,25	0,016	0,004	0,004	0,25	16	0,5	NA	NA	8122	Other	25	[illegible]
530056.fasta	530056	SAMEA5608259	PRJEB32435	36	M	Rogaland	West	Sekret	Urethra	2016-04-14	0,125	0,016	0,004	0,004	0,125	8	0,5	NA	NA	8122	Other	25	[illegible]
549528.fasta	549528	SAMEA5608350	PRJEB32435	23	M	Oslo	East	Sekret	Anus	2016-10-12	0,25	0,016	0,008	0,008	0,25	16	1	NA	NA	8122	Other	25	[illegible]
552468.fasta	552468	SAMEA5608363	PRJEB32435	15	M	Oslo	East	Sekret	Urethra	2016-01-07	0,25	0,016	0,008	0,008	0,5	16	0,5	NA	NA	8122	Other	25	[illegible]
552524.fasta	552524	SAMEA5608368	PRJEB32435	33	M	Vestland	West	Sekret	Urethra	2016-02-02	0,25	0,016	0,008	0,004	0,5	16	0,5	NA	NA	8122	Other	25	[illegible]
552526.fasta	552526	SAMEA5608369	PRJEB32435	22	F	Troms og Finnmark	North	Sekret	Cervix uter	2016-02-03	0,25	0,016	0,008	0,004	0,5	16	0,5	NA	NA	8122	Other	25	[illegible]
552553.fasta	552553	SAMEA5608370	PRJEB32435	30	M	Oslo	East	Sekret	Anus	2016-02-03	0,25	0,016	0,008	0,004	0,5	16	0,5	NA	NA	8122	Other	25	[illegible]
552606.fasta	552606	SAMEA5608379	PRJEB32435	21	M	Vestfold og Telemark	East	Sekret	Urethra	2016-02-24	0,125	0,016	0,008	0,004	0,25	8	0,5	NA	NA	8122	Other	25	[illegible]
552659.fasta	552659	SAMEA5608381	PRJEB32435	38	M	Oslo	East	Sekret	Urethra	2016-02-19	0,25	0,016	0,008	0,004	0,25	16	1	NA	NA	8122	Other	25	[illegible]
552828.fasta	552828	SAMEA5608383	PRJEB32435	23	M	Oslo	East	Sekret	Anus	2016-02-25	0,25	0,016	0,008	0,004	0,25	16	0,5	NA	NA	8122	Other	25	[illegible]
552852.fasta	552852	SAMEA5608388	PRJEB32435	26	M	Oslo	East	Sekret	Hals	2016-03-01	0,125	0,016	0,008	0,004	0,25	16	0,5	NA	NA	8122	Other	25	[illegible]
552891.fasta	552891	SAMEA5608400	PRJEB32435	20	F	Oslo	East	Sekret	Cervix uter	2016-03-18	0,125	0,016	0,008	0,004	0,25	8	0,5	NA	NA	8122	Other	25	[illegible]
552910.fasta	552910	SAMEA5608403	PRJEB32435	20	M	Viken	East	Ikke angitt		2016-11-08	0,25	0,016	0,008	0,004	0,25	32	1	NA	NA	1584	Other	9	[illegible]
553617.fasta	553617	SAMEA5608414	PRJEB32435	51	M	Viken	East	Sekret	Anus	2016-06-03	0,25	0,016	0,004	0,004	0,25	16	0,5	NA	NA	1584	Other	9	[illegible]
553623.fasta	553623	SAMEA5608417	PRJEB32435	41	M	Oslo	East	Sekret	Urethra	2016-06-20	0,25	0,016	0,004	0,004	0,25	16	1	NA	NA	1584	Other	9	[illegible]
553714.fasta	553714	SAMEA5608425	PRJEB32435	25	M	Oslo	East	Sekret	Urethra	2016-03-23	0,25	0,016	0,008	0,004	0,25	16	0,5	NA	NA	8122	Other	25	[illegible]
553744.fasta	553744	SAMEA5608434	PRJEB32435	20	M	Oslo	East	Sekret	Anus	2016-04-21	0,25	0,032	0,016	0,004	0,25	16	0,5	NA	NA	8122	Other	25	[illegible]
554584.fasta	554584	SAMEA5608454	PRJEB32435	32	M	Oslo	East	Sekret	Anus	2016-08-15	0,25	0,016	0,008	0,004	0,25	16	0,5	NA	NA	8122	Other	25	[illegible]
554791.fasta	554791	SAMEA5608477	PRJEB32435	33	M	Oslo	East	Sekret	Urethra	2016-07-22	0,25	0,016	0,008	0,004	0,25	16	0,5	NA	NA	8122	Other	25	[illegible]
554804.fasta	554804	SAMEA5608483	PRJEB32435	30	M	Oslo	East	Sekret	Urethra	2016-08-01	0,25	0,016	0,008	0,004	0,25	16	0,5	NA	NA	8122	Other	25	[illegible]
555559.fasta	555559	SAMEA5608498	PRJEB32435	26	M	Viken	East	Sekret	Urethra	2016-10-03	0,25	0,016	0,008	0,008	0,25	16	0,5	NA	NA	1584	Other	9	[illegible]
555572.fasta	555572	SAMEA5608501	PRJEB32435	34	M	Oslo	East	Sekret	Anus	2016-10-11	0,25	0,016	0,004	0,008	0,25	16	1	NA	NA	1584	Other	9	[illegible]
555607.fasta	555607	SAMEA5608504	PRJEB32435	25	M	Oslo	East	Sekret	Urethra	2016-10-11	0,25	0,016	0,008	0,008	0,25	16	0,5	NA	NA	8122	Other	25	[illegible]
555617.fasta	555617	SAMEA5608507	PRJEB32435	30	M	Oslo	East	Sekret	Urethra	2016-10-17	0,25	0,016	0,004	0,008	0,25	16	1	NA	NA	1584	Other	9	[illegible]
555820.fasta	555820	SAMEA5608512	PRJEB32435	30	M	Viken	East	Sekret	Urethra	2016-08-31	0,25	0,016	0,008	0,004	0,25	8	1	NA	NA	8122	Other	25	[illegible]
555855.fasta	555855	SAMEA5608520	PRJEB32435	27	M	Oslo	East	Sekret	Anus	2016-09-09	0,25	0,016	0,008	0,004	0,25	16	1	NA	NA	8122	Other	25	[illegible]
555897.fasta	555897	SAMEA5608524	PRJEB32435	40	M	Oslo	East	Sekret	Urethra	2016-09-16	0,25	0,016	0,008	0,008	0,25	16	1	NA	NA	1584	Other	9	[illegible]
556850.fasta	556850	SAMEA5608537	PRJEB32435	27	M	Oslo	East	Sekret	Anus	2016-11-04	0,25	0,016	0,008	0,004	0,25	16	0,5	NA	NA	8122	Other	25	[illegible]
556876.fasta	556876	SAMEA5608543	PRJEB32435	20	M	Viken	East	Sekret	Urethra	2016-11-14	0,125	0,016	0,004	0,008	0,125	16	0,25	NA	NA	1584	Other	9	[illegible]
557753.fasta	557753	SAMEA5608555	PRJEB32435	32	M	Vestland	West	Sårsekret	Anus	2016-12-12	0,25	0,016	0,008	0,008	0,25	32	1	NA	NA	1584	Other	9	[illegible]
563689.fasta	563689	SAMEA6533347	PRJEB32435	22	M	Oslo	East	Sekret	Anus	2016-11-28	0,25	0,016	0,004	0,004	0,25	16	0,5	NA	NA	1584	Other	9	[illegible]
563719.fasta	563719	SAMEA6533356	PRJEB32435	26	M	Nordland	North	Sekret	Urethra	2016-12-05	0,25	0,016	0,004	0,008	0,25	16	0,5	NA	NA	1584	Other	9	[illegible]
565460.fasta	565460	SAMEA5608646	PRJEB32435	35	M	Oslo	East	Sekret	Anus	2017-02-06	0,25	0,016	0,004	0,008	0,25	16	0,5	NA	NA	1584	Other	9	[illegible]
566483.fasta	566483	SAMEA5608658	PRJEB32435	22	M	Troms og Finnmark	North	Sekret	Anus	2017-02-13	0,25	0,016	0,004	0,004	0,25	16	0,5	NA	NA	1584	Other	9	[illegible]
576568.fasta	576568	SAMEA5608802	PRJEB32435	26	M	Oslo	East	Sekret	Rektum	2017-05-05	0,25	0,016	0,004	0,004	0,25	8	0,5	NA	NA	14350	Other	9	[illegible]
577023.fasta	577023	SAMEA5608806	PRJEB32435	37	M	Oslo	East	Sekret	Anus	2017-05-08	0,25	0,016	0,004	0,004	0,25	16	0,5	NA	NA	1584	Other	9	[illegible]
625543.fasta	625543	SAMEA6533536	PRJEB32435	29	F	NA	NA	SEKRET	CERVIXUT	2018-04-10	NA	NA	NA	NA	NA	NA	NA	NA	NA	8122	Other	25	[illegible]
626896.fasta	626896	SAMEA6533557	PRJEB32435	32	F	NA	NA	SEKRET	ANUS	2018-04-17	0,25	0,032	0,016	0,004	0,5	16	0,5	-	NA	8122	Other	25	[illegible]
641487.fasta	641487	SAMEA6533882	PRJEB32435	26	M	NA	NA	PUSS	URETRA	2018-10-11	0,25	0,016	0,016	0,003	NA	NA	NA	NA	NA	1584	Other	9	[illegible]
666909.fasta	666909	SAMEA6534329	PRJEB32435	22	M	Rogaland	West	Sekret	Urethra	2019-06-25	0,125	0,016	0,004	0,002	0,25	16	0,5	8	Ikke påvist	1584	Other	9	[illegible]
602144.fasta	602144	SAMEA5609103	PRJEB32435	38	M	Viken	East	Sekret	Hals	2017-10-16	0,25	0,032	0,032	2	0,5	16	1	NA	NA	7827	6	28	[illegible]
619248.fasta	619248	SAMEA6963022	PRJEB32435	22	F	Vestland	West	Sekret	Cervix uter	2018-02-20	0,5	0,125	0,032	32	2	16	2	NA	NA	1901	11	11	[illegible]
20GB001354.fasta	20GB001354	SAMEA117566960	PRJEB83897	26	F	Vestland	West	Sekret	Cervix uter	2020-11-10	0,25	0,016	0,016	0,004	0,032	16	0,25	NA	Ikke påvist	15664	Other	34	[illegible]
576450.fasta	576450	SAMEA5608799	PRJEB32435	18	F	Vestland	West	Sekret	Cervix uter	2017-05-05	0,125	0,004	0,016	0,125	0,125	16	0,5	NA	NA	13411	Other	9	[illegible]
580147.fasta	580147	SAMEA5608851	PRJEB32435	43	M	Oslo	East	Sekret	Urethra	2017-06-06	0,25	0,016	0,004	0,004	0,25	16	1	NA	NA	1599	10	6	[illegible]
595647.fasta	595647	SAMEA5608993	PRJEB32435	54	M	Oslo	East	Sekret	Urethra	2017-08-09	0,25	0,016	0,002	0,004	0,125	16	1	NA	NA	1599	10	6	[illegible]
581036.fasta	581036	SAMEA5608863	PRJEB32435	30	M	Agder	South	Sekret	Anus	2017-06-07	0,25	0,064	0,125	16	2	16	2	NA	NA	7827	6	28	[illegible]
20GB001227.fasta	20GB001227	SAMEA117566901	PRJEB83897	19	M	Viken	East	Sekret	Urethra	2020-09-16	0,125	0,016	0,004	0,004	0,5	4	0,25	NA	Ikke påvist	6726	Other	25	[illegible]
20GB001304.fasta	20GB001304	SAMEA117566937	PRJEB83897	26	M	Oslo	East	Sekret	Urethra	2020-10-20	0,125	0,016	0,004	0,004	0,5	4	0,25	NA	Ikke påvist	6726	Other	25	[illegible]
544354.fasta	544354	SAMEA5608338	PRJEB32435	21	M	Vestfold og Telemark	East	Sekret	Urethra	2016-08-29	0,5	0,016	0,008	0,008	0,25	16	0,25	NA	NA	8156	3	12	[illegible]
563148.fasta	563148	SAMEA5608604	PRJEB32435	52	M	Oslo	East	Sekret	Anus	2017-01-19	0,5	0,016	0,004	0,004	0,25	16	0,25	NA	NA	-	3	12	[illegible]
563196.fasta	563196	SAMEA5608611	PRJEB32435	22	M	Innlandet	East	Sekret	Hals	2017-01-17	0,5	0,016	0,004	0,004	0,25	16	0,25	NA	NA	14340	3	12	[illegible]
563203.fasta	563203	SAMEA5608613	PRJEB32435	34	M	Oslo	East	Sekret	Urethra	2017-01-17	0,5	0,016	0,004	0,004	0,25	16	0,125	NA	NA	8156	3	12	[illegible]
597325.fasta	597325	SAMEA5609028	PRJEB32435	18	M	Oslo	East	Ikke angitt		2017-08-30	0,5	0,016	0,004	0,004	0,25	8	0,25	NA	NA	8156	3	12	[illegible]
601812.fasta	601812	SAMEA5609097	PRJEB32435	36	M	Oslo	East	Sekret	Urethra	2017-10-11	0,5	0,016	0,002	0,004	0,25	16	0,25	NA	NA	8156	3	12	[illegible]
601961.fasta	601961	SAMEA5609100	PRJEB32435	71	M	Oslo	East	Sekret	Urethra	2017-10-11	0,25	0,016	0,002	0,004	0,125	8	0,25	NA	NA	8156	3	12	[illegible]
21GB000170.fasta	21GB000170	SAMEA117567015	PRJEB83897	20	M	Oslo	East	Sekret	Urethra	2021-04-13	0,5	0,032	0,032	0,004	1	16	1	NA	Ikke påvist	1893	Other	22	[illegible]
22GB000447.fasta	22GB000447	SAMEA117567332	PRJEB83897	20	F	Vestland	West	Sekret	Cervix uter	2022-04-26	0,064	0,016	0,016	1	2	8	16	4	Positiv	7363	5	33	[illegible]
22GB000678.fasta	22GB000678	SAMEA117567406	PRJEB83897	25	F	Vestland	West	Sekret	Cervix uter	2022-06-06	0,125	0,016	0,016	1	8	16	16	4	Positiv	7363	5	33	[illegible]
23GB000107.fasta	23GB000107	SAMEA117567999	PRJEB83897	20	F	Viken	East	Sekret	Cervix uter	2023-01-05	0,125	0,016	0,016	2	8	8	16	NA	Positiv	7363	5	33	[illegible]
624114.fasta	624114	SAMEA6533517	PRJEB32435	59	M	NA	NA	SEKRET	URETRA	2018-03-28	NA	NA	NA	NA	NA	NA	NA	NA	NA	7363	Other	8	[illegible]
20GB000382.fasta	20GB000382	SAMEA117566628	PRJEB83897	49	M	Viken	East	Sekret	Urethra	2020-02-04	0,25	0,016	0,016	2	32	8	32	8	Påvist	7363	Other	8	[illegible]
519462.fasta	519462	SAMEA5608234	PRJEB32435	25	M	Trøndelag	North	Sekret	Urethra	2016-02-01	0,125	0,016	0,004	0,004	0,125	16	16	NA	NA	12462	Other	9	[illegible]
556862.fasta	556862	SAMEA5608541	PRJEB32435	28	M	Oslo	East	Sekret	Urethra	2016-11-11	0,125	0,016	0,002	0,004	0,125	16	16	NA	NA	12462	Other	9	[illegible]
22GB000243.fasta	22GB000243	SAMEA117567277	PRJEB83897	20	F	Vestland	West	Sekret	Cervix uter	2022-03-17	0,125	0,016	0,016	1	4	16	16	NA	Påvist	7363	5	33	[illegible]
22GB000328.fasta	22GB000328	SAMEA117567302	PRJEB83897	19	F	Vestland	West	Sekret	Cervix uter	2022-04-01	0,125	0,016	0,016	1	4	8	16	NA	Positiv	7363	5	33	[illegible]
22GB000332.fasta	22GB000332	SAMEA117567303	PRJEB83897	21	F	Rogaland	West	Sekret	Vagina	2022-03-31	0,125	0,016	0,016	1	4	8	16	4	Positiv	7363	5	33	[illegible]
22GB000475.fasta	22GB000475	SAMEA117567343	PRJEB83897	20	M	Rogaland	West	Sekret	Urethra	2022-04-28	0,125	0,016	0,016	1	2	8	8	4	Positiv	7363	5	33	[illegible]
22GB000547.fasta	22GB000547	SAMEA117567358	PRJEB83897	20	F	Rogaland	West	Sekret	Vagina	2022-05-09	0,125	0,016	0,016	1	4	8	8	4	Positiv	7363	5	33	[illegible]
22GB000553.fasta	22GB000553	SAMEA117567361	PRJEB83897	28	M	Vestland	West	Sekret	Urethra	2022-05-11	0,125	0,016	0,016	1	4	8	8	2	Positiv	7363	5	33	[illegible]
22GB000701.fasta	22GB000701	SAMEA117567418	PRJEB83897	27	M	Vestland	West	Sekret	Urethra	2022-06-10	0,125	0,016	0,016	1	4	16	16	4	Positiv	7363	5	33	[illegible]
22GB000751.fasta	22GB000751	SAMEA117567444	PRJEB83897	27	F	Vestland	West	Sekret	Cervix uter	2022-06-20	0,125	0,016	0,016	1	8	16	8	4	Positiv	7363	5	33	[illegible]
22GB000752.fasta	22GB000752	SAMEA117567445	PRJEB83897	23	F	Viken	East	Sekret	Cervix uter	2022-06-21	0,125	0,016	0,016	1	8	16	16	4	Positiv	7363	5	33	[illegible]
22GB000858.fasta	22GB000858	SAMEA117567493	PRJEB83897	18	M	Rogaland	West	Sekret	Urethra	2022-07-12	0,125	0,016	0,002	0,5	8	16	8	8	Positiv	7363	5	33	[illegible]
22GB000866.fasta	22GB000866	SAMEA117567497	PRJEB83897	18	M	Rogaland	West	Sekret	Urethra	2022-07-20	0,125	0,016	0,016	1	16	16	16	4	Positiv	7363	5	33	[illegible]
22GB000923.fasta	22GB000923	SAMEA117567530	PRJEB83897	26	M	Oslo	East	Sekret	Urethra	2022-08-03	0,125	0,016	0,016	1	16	16	8	4	Positiv	7363	5	33	[illegible]
22GB000924.fasta	22GB000924	SAMEA117567531	PRJEB83897	18	F	Vestland	West	Sekret	Øye	2022-07-31	0,125	0,016	0,016	0,5	4	8	16	4	Positiv	7363	5	33	[illegible]
22GB000993.fasta	22GB000993	SAMEA117567574	PRJEB83897	17	F	Rogaland	West	Sekret	Hals	2022-08-16	0,125	0,016	0,016	1	8	8	32	4	Positiv	7363	5	33	[illegible]
22GB001001.fasta	22GB001001	SAMEA117567580	PRJEB83897	22	F	Viken	East	Sekret	Hals	2022-08-17	0,125	0,016	0,016	1	8	8	8	4	Positiv	7363	5	33	[illegible]
22GB001004.fasta	22GB001004	SAMEA117567582	PRJEB83897	25	F	Oslo	East	Sekret	Cervix uter	2022-08-16	0,125	0,016	0,002	0,5	8	16	8	8	Positiv	7363	5	33	[illegible]
22GB001024.fasta	22GB001024	SAMEA117567596	PRJEB83897	19	M	Vestland	West	Sekret	Urethra	2022-08-23	0,064	0,016	0,016	1	8	8	8	4	Positiv	7363	5	33	[illegible]
22GB001044.fasta	22GB001044	SAMEA117567610	PRJEB83897	17	F	Vestland	West	Sekret	Cervix uter	2022-08-25	0,064	0,016	0,016	1	8	8	16	4	Positiv	7363	5	33	[illegible]
22GB001073.fasta	22GB001073	SAMEA117567627	PRJEB83897	18	M	Vestland	West	Sekret	Urethra	2022-09-01	0,125	0,016	0,016	1	8	8	16	4	Positiv	7363	5	33	[illegible]
22GB001116.fasta	22GB001116	SAMEA117567656	PRJEB83897	18	M	Rogaland	West			2022-09-07	0,125	0,016	0,016	2	8	16	16	8	Positiv	7363	5	33	[illegible]
22GB001150.fasta	22GB001150	SAMEA117567671	PRJEB83897	18	M	Vestland	West	Sekret	Urethra	2022-09-12	0,125	0,016	0,016	1	8	8	16	8	Positiv	7363	5	33	[illegible]
22GB001151.fasta	22GB001151	SAMEA117567672	PRJEB83897	17	F	Vestland	West	Sekret	Cervix uter	2022-09-14	0,125	0,016	0,016	2	8	16	16	4	Positiv	7363	5	33	[illegible]
22GB001153.fasta	22GB001153	SAMEA117567674	PRJEB83897	18	M	Vestland	West	Sekret	Urethra	2022-09-13	0,125	0,016	0,016	1	8	8	16	4	Positiv	7363	5	33	[illegible]
22GB001156.fasta	22GB001156	SAMEA117567675	PRJEB83897	18	F	Vestland	West	Sekret	Cervix uter	2022-09-15	0,125	0,016	0,016	1	8	8	16	4	Positiv	7363	5	33	[illegible]
22GB001171.fasta	22GB001171	SAMEA117567680	PRJEB83897	20	F	Vestland	West	Sekret	Øye	2022-09-16	4	0,016	0,016	0,004	0,5	16	1	4	Positiv	7363	5	33	[illegible]
22GB001189.fasta	22GB001189	SAMEA117567690	PRJEB83897	20	F	Møre og Romsdal	West	Sekret	Cervix uter	2022-09-23	0,064	0,016	0,002	0,25	4	16	8	8	Positiv	7363	5	33	[illegible]
22GB001218.fasta	22GB001218	SAMEA117567705	PRJEB83897	18	F	Vestland	West	Sekret	Cervix uter	2022-09-27	0,25	0,016	0,016	2	8	8	16	8	Positiv	7363	5	33	[illegible]
22GB001266.fasta	22GB001266	SAMEA117567727	PRJEB83897	20	F	Vestland	West	Sekret	Cervix uter	2022-10-04	0,125	0,016	0,016	2	4	16	16	4	Positiv	7363	5	33	[illegible]
22GB001274.fasta	22GB001274	SAMEA117567731	PRJEB83897	42	F	Trøndelag	North	Sekret	Cervix uter	2022-10-03	0,25	0,016	0,016	2	32	16	16	4	Positiv	7363	5	33	[illegible]
22GB001564.fasta	22GB001564	SAMEA117567886	PRJEB83897	22	F	Rogaland	West	Sekret	Vagina	2022-11-18	0,125	0,016	0,016	2	16	16	32	4	Positiv	7363	5	33	[illegible]
22GB001690.fasta	22GB001690	SAMEA117567945	PRJEB83897	18	M	Vestland	West	Sekret	Urethra	2022-12-06	0,125	0,016	0,016	1	8	8	16	8	Negativ	7363	5	33	[illegible]
22GB001720.fasta	22GB001720	SAMEA117567956	PRJEB83897	20	F	Oslo	East	Sekret	Cervix uter	2022-12-14	0,064	0,016	0,016	1	8	8	8	8	Positiv	7363	5	33	[illegible]
23GB000044.fasta	23GB000044	SAMEA117567982	PRJEB83897	17	F	Vestland	West	Sekret	Cervix uter	2022-12-22	0,125	0,016	0,016	2	8	8	16	NA	Positiv	7363	5	33	[illegible]
23GB000047.fasta	23GB000047	SAMEA117567985	PRJEB83897	18	M	Vestland	West	Sekret	Urethra	2022-12-27	0,125	0,016	0,016	2	16	8	16	NA	Positiv	7363	5	33	[illegible]
23GB000106.fasta	23GB000106	SAMEA117567998	PRJEB83897	17	F	Vestland	West	Sekret	Cervix uter	2023-01-03	0,125	0,016	0,016	1	8	8	16	NA	Positiv	7363	5	33	[illegible]
23GB000173.fasta	23GB000173	SAMEA117568022	PRJEB83897	20	F	Vestland	West	Sekret	Cervix uter	2023-01-09	0,125	0,016	0,016	1	4	8	16	NA	Positiv	7363	5	33	[illegible]
23GB000174.fasta	23GB000174	SAMEA117568023	PRJEB83897	18	F	Vestland	West	Sekret	Cervix uter	2023-01-10	0,125	0,016	0,016	1	8	8	16	NA	Positiv	7363	5	33	[illegible]
23GB000178.fasta	23GB000178	SAMEA117568027	PRJEB83897	18	M	Vestland	West	Sekret	Urethra	2023-01-11	0,125	0,016	0,016	2	8	8	16	NA	Positiv	7363	5	33	[illegible]
23GB000187.fasta	23GB000187	SAMEA117568031	PRJEB83897	18	M	Troms og Finnmark	North	Sekret	Urethra	2023-01-09	0,125	0,016	0,016	2	8	8	16	NA	Positiv	7363	5	33	[illegible]
23GB000247.fasta	23GB000247	SAMEA117568064	PRJEB83897	18	F	Vestland	West	Sekret	Cervix uter	2023-01-17	0,064	0,016	0,016	1	8	8	16	NA	Positiv	7363	5	33	[illegible]
23GB000290.fasta	23GB000290	SAMEA117568084	PRJEB83897	21	F	Vestland	West	Sekret	Cervix uter	2023-01-24	0,125	0,016	0,016	2	16	8	16	NA	Positiv	7363	5	33	[illegible]
23GB000353.fasta	23GB000353	SAMEA117568121	PRJEB83897	18	M	Vestland	West	Sekret	Urethra	2023-02-03	0,125	0,016	0,016	1	8	8	16	NA	Positiv	7363	5	33	[illegible]
23GB000362.fasta	23GB000362	SAMEA117568130	PRJEB83897	31	M	Vestfold og Telemark	East	Sekret	Urethra	2023-01-31	0,125	0,016	0,016	2	16	16	16	NA	Positiv	7363	5	33	[illegible]
23GB000377.fasta	23GB000377	SAMEA117568135	PRJEB83897	26	M	Oslo	East	Sekret	Hals	2023-01-31	0,125	0,016	0,016	1	8	8	16	NA	Positiv	7363	5	33	[illegible]
23GB000532.fasta	23GB000532	SAMEA117568212	PRJEB83897	19	M	Vestland	West	Sekret	Urethra	2023-02-23	0,125	0,016	0,016	1	4	16	32	NA	Positiv	7363	5	33	[illegible]
23GB000568.fasta	23GB000568	SAMEA117568228	PRJEB83897	18	F	Vestland	West	Sekret	Cervix uter	2023-02-28	0,125	0,016	0,016	1	8	8	16	NA	Positiv	7363	5	33	[illegible]
23GB000642.fasta	23GB000642	SAMEA117568261	PRJEB83897	19	M	Vestland	West	Sekret	Urethra	2023-03-08	0,064	0,016	0,016	1	4	16	16	NA	Positiv	7363	5	33	[illegible]
23GB000643.fasta	23GB000643	SAMEA117568262	PRJEB83897	19	M	Vestland	West	Sekret	Urethra	2023-03-09	0,064	0,016	0,016	1	8	16	32	NA	Positiv	7363	5	33	[illegible]
23GB000648.fasta	23GB000648	SAMEA117568267	PRJEB83897	17	F	Vestland	West	Sekret	Cervix uter	2023-03-10	0,064	0,016	0,016	1	8	16	16	NA	Positiv	7363	5	33	[illegible]
23GB000755.fasta	23GB000755	SAMEA117568304	PRJEB83897	17	F	Vestland	West	Sekret	Cervix uter	2023-03-21	0,125	0,016	0,016	1	2	16	16	NA	Positiv	7363	5	33	[illegible]
23GB000944.fasta	23GB000944	SAMEA117568388	PRJEB83897	20	F	Oslo	East	Sekret	Vagina	2023-04-20	0,064	0,016	0,016	2	4	8	8	NA	Positiv	7363	5	33	[illegible]
20GB001207.fasta	20GB001207	SAMEA117566888	PRJEB83897	18	F	Viken	East	Sekret	Vagina	2020-09-02	0,25	0,016	0,004	0,5	0,125	8	0,25	NA	Ikke påvist	7363	5	33	[illegible]
22GB001179.fasta	22GB001179	SAMEA117567686	PRJEB83897	30	M	Rogaland	West	Sekret	Urethra	2022-09-20	0,25	0,016	0,016	4	4	16	16	8	Positiv	7363	5	33	[illegible]
23GB000300.fasta	23GB000300	SAMEA117568089	PRJEB83897	55	M	Oslo	East	Sekret	Urethra	2023-01-26	0,125	0,016	0,016	4	8	16	16	NA	Positiv	7363	5	33	[illegible]
23GB000423.fasta	23GB000423	SAMEA117568157	PRJEB83897	44	M	Viken	East	Sekret	Urethra	2023-02-08	0,25	0,016	0,004	2	4	16	8	NA	Positiv	7363	5	33	[illegible]
595945.fasta	595945	SAMEA5609003	PRJEB32435	16	M	Viken	East	Sekret	Urethra	2017-08-15	0,125	0,016	0,004	8	2	16	16	NA	NA	7363	5	33	[illegible]
563773.fasta	563773	SAMEA6533370	PRJEB32435	37	M	Oslo	East	Sekret	Urethra	2016-12-27	0,125	0,016	0,004	2	4	16	8	NA	NA	1587	5	33	[illegible]
23GB000078.fasta	23GB000078	SAMEA117567991	PRJEB83897	25	M	Oslo	East	Sekret	Urethra	2022-12-29	0,5	0,016	0,004	2	8	16	16	NA	Positiv	10634	5	33	[illegible]
23GB000226.fasta	23GB000226	SAMEA117568053	PRJEB83897	60	M	Oslo	East	Sekret	Anus	2023-01-16	0,5	0,016	0,016	16	8	16	32	NA	Positiv	10634	5	33	[illegible]
22GB001635.fasta	22GB001635	SAMEA117567922	PRJEB83897	23	M	Oslo	East	Sekret	Anus	2022-11-30	0,25	0,016	0,016	4	8	16	16	4	Positiv	7363	5	33	[illegible]
22GB000550.fasta	22GB000550	SAMEA117567359	PRJEB83897	22	F	Vestland	West	Sekret	Cervix uter	2022-05-12	0,125	0,016	0,016	1	8	16	16	4	Positiv	7363	5	33	[illegible]
21GB000872.fasta	21GB000872	SAMEA117567182	PRJEB83897	25	M	Oslo	East	Sekret	Urethra	2021-12-20	0,064	0,016	0,002	0,004	0,016	16	0,064	NA	Ikke påvist	6962	Other	20	[illegible]
592342.fasta	592342	SAMEA5608900	PRJEB32435	30	F	Rogaland	West	Sekret	Cervix uter	2017-07-06	0,125	0,016	0,002	0,002	0,016	8	0,125	NA	NA	8135	Other	2	[illegible]
22GB000450.fasta	22GB000450	SAMEA117567335	PRJEB83897	34	M	Vestland	West	Sekret	Urethra	2022-04-28	0,125	0,016	0,016	2	0,125	16	0,5	4	Negativ	7363	Other	8	[illegible]
631504.fasta	631504	SAMEA6533622	PRJEB32435	41	M	NA	NA	SEKRET	URETRA	2018-06-09	NA	NA	NA	NA	NA	NA	NA	NA	NA	1901	11	11	[illegible]
636538.fasta	636538	SAMEA6533749	PRJEB32435	55	M	NA	NA	SEKRET	ANUS	2018-08-15	NA	NA	NA	NA	NA	NA	NA	NA	NA	1580	1	18	[illegible]
636875.fasta	636875	SAMEA6533763	PRJEB32435	34	M	NA	NA	SEKRET	URETRA	2018-08-23	NA	NA	NA	NA	NA	NA	NA	NA	NA	1580	1	18	[illegible]
20GB000098.fasta	20GB000098	SAMEA117566536	PRJEB83897	53	F	Viken	East	Sekret	Vagina	2019-12-30	0,25	0,064	0,064	4	1	16	2	8	Ikke påvist	7827	6	28	[illegible]
20GB001085.fasta	20GB001085	SAMEA117566832	PRJEB83897	28	M	Trøndelag	North	Sekret	Urethra	2020-07-15	0,5	0,016	0,032	16	1	8	2	NA	Ikke påvist	1901	11	11	[illegible]
659555.fasta	659555	SAMEA6534155	PRJEB32435	49	M	Oslo	East	Sekret	Anus	2019-04-01	0,5	0,016	0,008	8	1	16	0,5	Ikke utført	Ikke påvist	1901	11	11	[illegible]
666018.fasta	666018	SAMEA6534305	PRJEB32435	29	M	Oslo	East	Sekret	Anus	2019-06-12	0,5	0,032	0,016	8	2	16	1	4	Ikke påvist	1901	11	11	[illegible]
575409.fasta	575409	SAMEA5608783	PRJEB32435	41	M	Viken	East	Sekret	Rektum	2017-04-23	2	0,125	0,032	32	1	16	2	NA	NA	1901	11	11	[illegible]
610072.fasta	610072	SAMEA5609153	PRJEB32435	64	M	Viken	East	Sekret	Urethra	2017-12-21	1	0,032	0,008	8	0,25	8	1	NA	NA	1901	11	11	[illegible]
612554.fasta	612554	SAMEA6533432	PRJEB32435	52	M	Rogaland	West	Sekret	Urethra	2018-01-10	1	0,125	0,032	32	1	16	2	NA	NA	1901	11	11	[illegible]
612556.fasta	612556	SAMEA6533433	PRJEB32435	54	F	Rogaland	West	Sekret	Cervix uter	2018-01-09	1	0,125	0,032	32	1	16	2	NA	NA	1901	11	11	[illegible]
666558.fasta	666558	SAMEA6534315	PRJEB32435	45	M	Viken	East	Sekret	Urethra	2019-06-21	0,5	0,125	0,064	32	1	16	1	8	Ikke påvist	1901	11	11	[illegible]
667048.fasta	667048	SAMEA6534333	PRJEB32435	27	F	Oslo	East	Sekret	Hals	2019-06-21	0,5	0,125	0,064	32	1	16	2	4	Ikke påvist	1901	11	11	[illegible]
20GB000712.fasta	20GB000712	SAMEA117566722	PRJEB83897	29	M	Oslo	East	Sekret	Hals	2020-03-11	0,032	0,016	0,002	0,002	0,125	8	0,125	NA	Ikke påvist	15376	Other	26	[illegible]
628638.fasta	628638	SAMEA6533578	PRJEB32435	37	M	NA	NA	SEKRET	URETRA	2018-05-09	0,25	0,016	0,002	0,002	0,125	16	0,125	-	NA	8156	3	12	[illegible]
578273.fasta	578273	SAMEA5608830	PRJEB32435	21	M	Vestland	West	Sekret	Urethra	2017-05-18	0,125	0,016	0,004	0,004	0,064	16	0,25	NA	NA	13411	Other	9	[illegible]
534235.fasta	534235	SAMEA5608277	PRJEB32435	19	F	Vestland	West	Sekret	Cervix uter	2016-04-11	0,064	0,016	0,002	8	0,125	8	32	NA	NA	11750	Other	7	[illegible]
22GB001380.fasta	22GB001380	SAMEA117567788	PRJEB83897	17	M	Oslo	East	Sekret	Urethra	2022-10-20	0,125	0,016	0,002	0,5	0,125	8	4	2	Negativ	11750	Other	7	[illegible]
534219.fasta	534219	SAMEA5608276	PRJEB32435	20	F	Vestland	West	Sekret	Cervix uter	2016-04-24	0,032	0,016	0,002	4	0,125	8	16	NA	NA	11750	Other	7	[illegible]
668633.fasta	668633	SAMEA6534474	PRJEB32435	35	M	Oslo	East	Sekret	Urethra	2019-07-26	0,25	0,016	0,002	1	32	16	8	4	Påvist	13735	Other	7	[illegible]
22GB001265.fasta	22GB001265	SAMEA117567726	PRJEB83897	23	M	Møre og Romsdal	West	Sekret	Urethra	2022-10-07	0,125	0,016	0,016	0,004	0,016	8	0,125	8	Negativ	8135	Other	2	[illegible]
534448.fasta	534448	SAMEA5608286	PRJEB32435	33	M	Vestland	West	Sekret	Urethra	2016-05-12	0,125	0,016	0,002	0,004	0,032	16	0,5	NA	NA	13923	Other	2	[illegible]
556837.fasta	556837	SAMEA5608533	PRJEB32435	20	M	Oslo	East	Sekret	Anus	2016-10-31	0,25	0,016	0,002	0,004	0,016	8	0,064	NA	NA	8135	Other	2	[illegible]
634390.fasta	634390	SAMEA6533694	PRJEB32435	37	M	NA	NA	SEKRET	URETRA	2018-07-18	NA	NA	NA	NA	NA	NA	NA	NA	NA	8135	Other	2	[illegible]
634395.fasta	634395	SAMEA6533696	PRJEB32435	30	F	NA	NA	SEKRET	CERVIXUT	2018-07-18	NA	NA	NA	NA	NA	NA	NA	NA	NA	8135	Other	2	[illegible]
636796.fasta	636796	SAMEA6533760	PRJEB32435	23	M	NA	NA	SEKRET	URETRA	2018-08-17	NA	NA	NA	NA	NA	NA	NA	NA	NA	8135	Other	2	[illegible]
661105.fasta	661105	SAMEA6534182	PRJEB32435	30	F	Viken	East	Sekret	Cervix uter	2019-04-19	0,125	0,016	0,002	0,002	0,016	8	0,064	NA	NA	8135	Other	2	[illegible]
20GB000588.fasta	20GB000588	SAMEA117566673	PRJEB83897	49	M	Oslo	East	Sekret	Urethra	2020-02-21	0,5	0,032	0,032	1	0,5	16	0,5	NA	Ikke påvist	7827	6	28	[illegible]
597321.fasta	597321	SAMEA5609026	PRJEB32435	25	F	Vestland	West	Sekret	Cervix uter	2017-08-28	0,125	0,064	0,008	4	0,125	16	1	NA	NA	1594	Other	3	[illegible]
551147.fasta	551147	SAMEA5608355	PRJEB32435	18	F	Vestfold og Telemark	East	Sekret	Cervix uter	2016-10-25	0,064	0,016	0,002	0,002	0,008	16	8	NA	NA	8135	Other	2	[illegible]
554813.fasta	554813	SAMEA5608485	PRJEB32435	26	M	Oslo	East	Sekret	Urethra	2016-08-01	0,125	0,016	0,002	0,004	0,016	8	0,064	NA	NA	8135	Other	2	[illegible]
532439.fasta	532439	SAMEA5608270	PRJEB32435	29	F	Vestland	West	Sekret	Cervix uter	2016-05-04	0,064	0,016	0,002	0,004	0,016	16	0,125	NA	NA	13923	Other	2	[illegible]
20GB000577.fasta	20GB000577	SAMEA117566666	PRJEB83897	25	M	Rogaland	West	Sekret	Urethra	2020-02-21	0,064	0,016	0,016	0,004	0,016	8	0,125	4	Ikke påvist	8135	Other	2	[illegible]
20GB000706.fasta	20GB000706	SAMEA117566717	PRJEB83897	34	M	Oslo	East	Sekret	Urethra	2020-03-13	0,064	0,016	0,002	0,004	0,016	16	0,064	NA	Ikke påvist	15755	Other	2	[illegible]
20GB000741.fasta	20GB000741	SAMEA117566734	PRJEB83897	17	F	Vestfold og Telemark	East	Sekret	Cervix uter	2020-03-16	0,125	0,016	0,016	0,004	0,016	8	0,25	NA	Ikke påvist	15755	Other	2	[illegible]
20GB001384.fasta	20GB001384	SAMEA117566973	PRJEB83897	26	M	Rogaland	West	Sekret	Urethra	2020-11-25	0,125	0,016	0,016	0,004	0,016	8	0,125	NA	Ikke påvist	8135	Other	2	[illegible]
21GB000482.fasta	21GB000482	SAMEA117567066	PRJEB83897	18	F	Oslo	East	Sekret	Cervix uter	2021-08-31	0,25	0,016	0,016	0,004	0,016	16	0,25	NA	Ikke påvist	8135	Other	2	[illegible]
21GB000514.fasta	21GB000514	SAMEA117567076	PRJEB83897	25	F	Vestfold og Telemark	East	Sekret	Vagina	2021-09-16	0,064	0,016	0,002	0,002	0,002	4	0,064	NA	Ikke påvist	1892	Other	2	[illegible]
21GB000666.fasta	21GB000666	SAMEA117567116	PRJEB83897	21	M	Oslo	East	Sekret	Urethra	2021-10-27	0,064	0,016	0,002	0,002	0,004	4	0,016	NA	Ikke påvist	8135	Other	2	[illegible]
22GB001012.fasta	22GB001012	SAMEA117567590	PRJEB83897	39	F	Oslo	East	Sekret	Cervix uter	2022-08-17	0,125	0,016	0,002	0,002	0,008	8	0,064	4	Negativ	8135	Other	2	[illegible]
23GB000077.fasta	23GB000077	SAMEA117567990	PRJEB83897	19	M	Oslo	East	Sekret	Urethra	2022-12-29	0,125	0,016	0,002	0,002	0,008	16	0,064	8	Negativ	8135	Other	2	[illegible]
552521.fasta	552521	SAMEA5608367	PRJEB32435	26	M	Oslo	East	Sekret	Urethra	2016-01-25	0,125	0,016	0,002	0,004	0,032	16	0,125	NA	NA	8135	Other	2	[illegible]
563707.fasta	563707	SAMEA6533354	PRJEB32435	23	M	Viken	East	Sekret	Urethra	2016-12-07	0,125	0,016	0,002	0,004	0,016	8	0,125	NA	NA	13967	Other	2	[illegible]
575440.fasta	575440	SAMEA5608784	PRJEB32435	27	F	Vestfold og Telemark	East	Sekret	Urethra	2017-04-24	0,064	0,016	0,002	0,004	0,016	8	0,25	NA	NA	13923	Other	2	[illegible]
615880.fasta	615880	SAMEA6533449	PRJEB32435	21	M	Vestland	West	Sekret	Urethra	2018-01-31	0,064	0,016	0,002	0,004	0,016	8	0,25	NA	NA	13923	Other	2	[illegible]
630442.fasta	630442	SAMEA6533603	PRJEB32435	16	F	NA	NA	SEKRET	CERVIXUT	2018-05-30	NA	NA	NA	NA	NA	NA	NA	NA	NA	8135	Other	2	[illegible]
643385.fasta	643385	SAMEA6533923	PRJEB32435	58	M	NA	NA	SEKRET	URETRA	2018-11-01	0,125	0,016	0,016	0,002	NA	NA	NA	NA	NA	1892	Other	2	[illegible]
658876.fasta	658876	SAMEA6534144	PRJEB32435	40	F	Innlandet	East	Sekret	Vagina	2019-03-29	0,125	0,016	0,002	0,002	0,016	16	0,25	NA	NA	1892	Other	2	[illegible]
668986.fasta	668986	SAMEA6534486	PRJEB32435	55	M	Viken	East	Ikke angitt		2019-07-30	0,25	0,016	0,002	0,004	0,032	32	0,25	8	Ikke påvist	8135	Other	2	[illegible]
670880.fasta	670880	SAMEA6534551	PRJEB32435	51	M	Viken	East	Sekret	Annet, spe	2019-08-20	0,125	0,016	0,002	0,004	0,016	16	0,125	4	Ikke påvist	8135	Other	2	[illegible]
561760.fasta	561760	SAMEA5608585	PRJEB32435	35	M	Oslo	East	Sekret	Urethra	2017-01-12	0,5	0,016	0,002	0,008	0,25	16	0,25	NA	NA	1596	Other	1	[illegible]
593237.fasta	593237	SAMEA5608939	PRJEB32435	19	F	Viken	East	Sekret	Vagina	2017-07-21	0,25	0,016	0,002	0,004	0,25	8	0,25	NA	NA	1596	Other	1	[illegible]
610777.fasta	610777	SAMEA6533424	PRJEB32435	45	M	Oslo	East	Sekret	Urethra	2017-12-29	0,25	0,016	0,004	0,004	0,25	16	0,25	NA	NA	8156	3	12	[illegible]
677267.fasta	677267	SAMEA6534696	PRJEB32435	21	F	Trøndelag	North	Sekret	Cervix uter	2019-11-13	0,25	0,016	0,016	0,008	0,5	8	0,25	8	Ikke påvist	7359	4	5	[illegible]
20GB000170.fasta	20GB000170	SAMEA117566567	PRJEB83897	30	M	Viken	East	Sekret	Urethra	2019-12-17	0,125	0,016	0,008	0,002	0,064	4	0,125	2	Ikke påvist	11990	Other	3	[illegible]
20GB000307.fasta	20GB000307	SAMEA117566611	PRJEB83897	23	F	Vestland	West	Sekret	Cervix uter	2020-01-20	0,125	0,016	0,016	0,004	0,125	4	0,125	4	Ikke påvist	11990	Other	3	[illegible]
20GB000429.fasta	20GB000429	SAMEA117566631	PRJEB83897	20	M	Vestland	West	Sekret	Urethra	2020-01-28	0,125	0,016	0,016	0,004	0,125	4	0,25	4	Ikke påvist	11990	Other	3	[illegible]
20GB000466.fasta	20GB000466	SAMEA117566645	PRJEB83897	21	F	Oslo	East	Sekret	Cervix uter	2020-02-03	0,125	0,016	0,004	0,004	0,125	8	0,125	NA	Ikke påvist	11990	Other	3	[illegible]
20GB001284.fasta	20GB001284	SAMEA117566923	PRJEB83897	30	M	Oslo	East	Sekret	Urethra	2020-10-12	0,125	0,032	0,008	0,004	0,125	4	0,125	NA	Ikke påvist	11990	Other	3	[illegible]
569374.fasta	569374	SAMEA5608708	PRJEB32435	27	M	Møre og Romsdal	West	Sekret	Urethra	2017-03-06	0,125	0,016	0,004	0,004	0,125	16	0,125	NA	NA	1594	Other	3	[illegible]
573138.fasta	573138	SAMEA5608751	PRJEB32435	18	F	Trøndelag	North	Sekret	Cervix uter	2017-03-30	0,25	0,016	0,004	0,004	0,125	8	0,25	NA	NA	1594	Other	3	[illegible]
630436.fasta	630436	SAMEA6963064	PRJEB32435	57	M	NA	NA	SEKRET	URETRA	2018-05-30	NA	NA	NA	NA	NA	NA	NA	NA	NA	1594	Other	3	[illegible]
655671.fasta	655671	SAMEA6534100	PRJEB32435	19	F	Vestland	West	Sekret	Cervix uter	2019-02-28	0,064	0,016	0,008	0,002	0,064	4	0,125	NA	NA	11990	Other	3	[illegible]
658651.fasta	658651	SAMEA6534142	PRJEB32435	17	F	Vestland	West	Sekret	Cervix uter	2019-03-21	0,125	0,016	0,004	0,002	0,064	4	0,125	NA	NA	11990	Other	3	[illegible]
663219.fasta	663219	SAMEA6534224	PRJEB32435	18	F	Rogaland	West	Sekret	Cervix uter	2019-05-09	0,125	0,016	0,004	0,002	0,125	4	0,125	4	Ikke påvist	11990	Other	3	[illegible]
664310.fasta	664310	SAMEA6534252	PRJEB32435	22	M	Vestland	West	Sekret	Urethra	2019-05-24	0,064	0,016	0,004	0,002	0,125	8	0,064	4	Ikke påvist	11990	Other	3	[illegible]
666045.fasta	666045	SAMEA6534308	PRJEB32435	19	F	Vestland	West	Sekret	Vagina	2019-06-13	0,125	0,016	0,008	0,002	0,25	8	0,125	4	Ikke påvist	11990	Other	3	[illegible]
667432.fasta	667432	SAMEA6534354	PRJEB32435	18	F	Vestland	West	Sekret	Cervix uter	2019-07-04	0,125	0,016	0,008	0,004	0,125	4	0,25	2	Ikke påvist	11990	Other	3	[illegible]
667737.fasta	667737	SAMEA117568448	PRJEB83897	24	F	Oslo	East		Cervix uter	2019-07-08	0,125	0,016	0,004	0,004	0,125	8	0,125	2	Ikke påvist	1594	Other	3	[illegible]
670269.fasta	670269	SAMEA6534521	PRJEB32435	19	F	Vestland	West	Sekret	Cervix uter	2019-08-13	0,064	0,016	0,004	0,002	0,064	4	0,125	2	Ikke påvist	11990	Other	3	[illegible]
674481.fasta	674481	SAMEA117568451	PRJEB83897	19	M	Rogaland	West	Sekret	Urethra	2019-10-09	0,125	0,016	0,004	0,004	0,064	4	0,125	4	Ikke påvist	11990	Other	3	[illegible]
676004.fasta	676004	SAMEA6963078	PRJEB32435	40	F	Oslo	East	Ikke angitt		2019-10-28	0,25	0,016	0,008	0,004	0,125	16	0,125	4	Ikke påvist	1594	Other	3	[illegible]
678684.fasta	678684	SAMEA6534729	PRJEB32435	20	M	Vestland	West	Sekret	Urethra	2019-11-29	0,125	0,016	0,008	0,004	0,125	2	0,064	4	Ikke påvist	11990	Other	3	[illegible]
676694.fasta	676694	SAMEA6534681	PRJEB32435	41	F	Oslo	East	Sekret	Cervix uter	2019-11-05	0,25	0,016	0,016	0,004	0,125	8	16	8	Ikke påvist	1594	Other	3	[illegible]
555869.fasta	555869	SAMEA5608522	PRJEB32435	21	F	Oslo	East	Sekret	Cervix uter	2016-09-13	0,125	0,016	0,004	0,004	0,064	8	0,125	NA	NA	1594	Other	3	[illegible]
22GB001428.fasta	22GB001428	SAMEA117567821	PRJEB83897	27	F	Trøndelag	North	Sekret	Cervix uter	2022-10-14	0,5	0,016	0,016	0,004	0,25	16	0,25	4	Negativ	1596	Other	1	[illegible]
22GB001451.fasta	22GB001451	SAMEA117567832	PRJEB83897	21	M	Trøndelag	North	Sekret	Urethra	2022-10-31	0,5	0,016	0,016	0,004	0,25	16	0,25	8	Negativ	1596	Other	1	[illegible]
22GB001590.fasta	22GB001590	SAMEA117567899	PRJEB83897	23	F	Vestland	West		Cervix uter	2022-11-25	0,25	0,016	0,016	0,004	0,25	16	0,125	4	Negativ	1596	Other	1	[illegible]
22GB001601.fasta	22GB001601	SAMEA117567904	PRJEB83897	27	M	Oslo	East	Sekret	Urethra	2022-11-24	0,25	0,016	0,016	0,004	0,25	16	0,125	4	Negativ	1596	Other	1	[illegible]
23GB000049.fasta	23GB000049	SAMEA117567987	PRJEB83897	24	M	Oslo	East	Sekret	Urethra	2022-12-21	0,25	0,016	0,016	0,004	0,25	8	0,125	NA	Negativ	1596	Other	1	[illegible]
23GB000857.fasta	23GB000857	SAMEA117568357	PRJEB83897	17	F	Vestland	West	Sekret	Hals	2023-04-04	0,25	0,016	0,016	0,004	0,125	16	0,25	NA	Negativ	1596	Other	1	[illegible]
23GB000859.fasta	23GB000859	SAMEA117568359	PRJEB83897	16	M	Vestland	West	Sekret	Urethra	2023-03-31	0,25	0,016	0,016	0,004	0,125	8	0,125	NA	Negativ	1596	Other	1	[illegible]
551585.fasta	551585	SAMEA5608357	PRJEB32435	29	F	Oslo	East	Sekret	Cervix uter	2016-10-31	0,5	0,016	0,002	0,004	0,5	16	0,25	NA	NA	1596	Other	1	[illegible]
551600.fasta	551600	SAMEA5608358	PRJEB32435	21	M	Vestland	West	Sekret	Urethra	2016-11-02	0,5	0,016	0,004	0,008	0,5	16	0,25	NA	NA	1596	Other	1	[illegible]
553887.fasta	553887	SAMEA5608444	PRJEB32435	37	M	Viken	East	Sekret	Urethra	2016-11-17	0,25	0,016	0,002	0,004	0,125	8	0,25	NA	NA	1596	Other	1	[illegible]
555614.fasta	555614	SAMEA5608506	PRJEB32435	41	M	Oslo	East	Sekret	Urethra	2016-10-13	0,5	0,016	0,002	0,004	0,25	16	0,25	NA	NA	1596	Other	1	[illegible]
556846.fasta	556846	SAMEA5608536	PRJEB32435	29	F	Oslo	East	Sekret	Cervix uter	2016-11-03	0,5	0,016	0,002	0,004	0,25	16	0,125	NA	NA	1596	Other	1	[illegible]
562099.fasta	562099	SAMEA5608590	PRJEB32435	25	M	Viken	East	Sekret	Urethra	2017-01-17	0,5	0,016	0,004	0,004	0,25	16	0,125	NA	NA	1596	Other	1	[illegible]
573980.fasta	573980	SAMEA5608771	PRJEB32435	25	M	Oslo	East	Sekret	Urethra	2017-04-03	0,5	0,016	0,002	0,004	0,25	16	0,25	NA	NA	14348	Other	1	[illegible]
592626.fasta	592626	SAMEA5608919	PRJEB32435	30	M	Oslo	East	Sekret	Urethra	2017-07-13	0,5	0,016	0,002	0,004	0,25	8	0,125	NA	NA	1596	Other	1	[illegible]
593250.fasta	593250	SAMEA5608945	PRJEB32435	22	M	Oslo	East	Sekret	Urethra	2017-07-21	0,25	0,016	0,002	0,004	0,25	16	0,25	NA	NA	1596	Other	1	[illegible]
629597.fasta	629597	SAMEA6963052	PRJEB32435	20	F	NA	NA	NULL	NULL	2018-05-23	NA	NA	NA	NA	NA	NA	NA	NA	NA	1596	Other	1	[illegible]
636544.fasta	636544	SAMEA6533752	PRJEB32435	29	M	NA	NA	SEKRET	URETRA	2018-08-16	NA	NA	NA	NA	NA	NA	NA	NA	NA	1596	Other	1	[illegible]
637117.fasta	637117	SAMEA6533771	PRJEB32435	23	M	NA	NA	SEKRET	URETRA	2018-08-24	NA	NA	NA	NA	NA	NA	NA	NA	NA	1596	Other	1	[illegible]
640307.fasta	640307	SAMEA6533837	PRJEB32435	17	F	NA	NA	SEKRET	CERVIXUT	2018-09-18	0,25	0,016	0,016	0,002	NA	NA	NA	NA	NA	1596	Other	1	[illegible]
640493.fasta	640493	SAMEA6533851	PRJEB32435	24	M	NA	NA	SEKRET	URETRA	2018-09-27	0,19	0,016	0,016	0,002	NA	NA	NA	NA	NA	1596	Other	1	[illegible]
642710.fasta	642710	SAMEA6533913	PRJEB32435	28	M	NA	NA	SEKRET	URETRA	2018-10-26	0,25	0,016	0,016	0,002	NA	NA	NA	NA	NA	1596	Other	1	[illegible]
643144.fasta	643144	SAMEA6533921	PRJEB32435	21	F	NA	NA	SEKRET	CERVIXUT	2018-10-30	0,125	0,016	0,016	0,002	NA	NA	NA	NA	NA	1596	Other	1	[illegible]
643824.fasta	643824	SAMEA6533930	PRJEB32435	36	M	NA	NA	SEKRET	ANUS	2018-11-07	NA	NA	NA	NA	NA	NA	NA	NA	NA	1596	Other	1	[illegible]
646329.fasta	646329	SAMEA6533967	PRJEB32435	23	M	NA	NA	SEKRET	URETRA	2018-11-27	NA	NA	NA	NA	NA	NA	NA	NA	NA	1596	Other	1	[illegible]
646800.fasta	646800	SAMEA6533975	PRJEB32435	17	F	NA	NA	SEKRET	CERVIXUT	2018-11-26	NA	NA	NA	NA	NA	NA	NA	NA	NA	1596	Other	1	[illegible]
647735.fasta	647735	SAMEA6533991	PRJEB32435	62	M	NA	NA	SEKRET	URETRA	2018-12-14	NA	NA	NA	NA	NA	NA	NA	NA	NA	1596	Other	1	[illegible]
655075.fasta	655075	SAMEA6534086	PRJEB32435	24	F	Innlandet	East	Sekret	Cervix uter	2019-02-22	0,25	0,016	0,002	0,004	0,25	16	0,25	NA	NA	1596	Other	1	[illegible]
663543.fasta	663543	SAMEA6534231	PRJEB32435	30	M	Oslo	East	Sekret	Ureter	2019-05-14	0,25	0,016	0,002	0,002	0,125	16	0,125	4	Ikke påvist	1596	Other	1	[illegible]
667122.fasta	667122	SAMEA6534335	PRJEB32435	23	M	Agder	South	Sekret	Urethra	2019-06-27	0,5	0,016	0,002	0,004	0,25	16	0,125	4	Ikke påvist	1596	Other	1	[illegible]
677143.fasta	677143	SAMEA6534689	PRJEB32435	43	F	Viken	East	Sekret	Hals	2019-11-08	0,25	0,016	0,004	0,004	0,25	16	0,125	2	Ikke påvist	1596	Other	1	[illegible]

20GB000285.fasta	20GB000285	SAMEA117566597	PRJEB83897	22	F	Trøndelag	North	Sekret	Cervix uter	2020-01-17	0,064	0,016	0,016	0,004	0,125	16	0,25		8	Ikke påvist	1594	Other	3	1267	NOT_FOUND	NOT_FOUND	NOT_FOUND	NOT_FOUND	INTERMEDIATE	NOT_FOUND	NOT_FOUND	NOT_FOUND	penA_ins346D	0	1
20GB001288.fasta	20GB001288	SAMEA117566927	PRJEB83897	26	F	Oslo	East	Sekret	Cervix uter	2020-10-15	0,25	0,016	0,016	0,004	0,125	8	0,25	NA		Ikke påvist	11990	Other	3	962	NOT_FOUND	NOT_FOUND	NOT_FOUND	NOT_FOUND	INTERMEDIATE	NOT_FOUND	NOT_FOUND	NOT_FOUND	penA_ins346D	0	1
21GB000472.fasta	21GB000472	SAMEA117567063	PRJEB83897	20	F	Oslo	East	Sekret	Cervix uter	2021-08-24	0,125	0,016	0,002	0,004	0,064	8	0,064	NA		Ikke påvist	15957	Other	3	20	NOT_FOUND	NOT_FOUND	NOT_FOUND	NOT_FOUND	INTERMEDIATE	NOT_FOUND	NOT_FOUND	NOT_FOUND	penA_ins346D	0	1
21GB000547.fasta	21GB000547	SAMEA117567087	PRJEB83897	20	F	Oslo	East	Sekret	Cervix uter	2021-09-23	0,125	0,016	0,002	0,002	0,032	8	0,064	NA		Ikke påvist	15957	Other	3	20	NOT_FOUND	NOT_FOUND	NOT_FOUND	NOT_FOUND	INTERMEDIATE	NOT_FOUND	NOT_FOUND	NOT_FOUND	penA_ins346D	0	1
21GB000569.fasta	21GB000569	SAMEA117567092	PRJEB83897	19	M	Oslo	East	Sekret	Urethra	2021-09-30	0,125	0,016	0,004	0,004	0,032	4	0,064	NA		Ikke påvist	15957	Other	3	20	NOT_FOUND	NOT_FOUND	NOT_FOUND	NOT_FOUND	INTERMEDIATE	NOT_FOUND	NOT_FOUND	NOT_FOUND	penA_ins346D	0	1
21GB000752.fasta	21GB000752	SAMEA117567138	PRJEB83897	34	F	Oslo	East	Sekret	Urethra	2021-11-22	0,064	0,016	0,004	0,002	0,032	8	0,064	NA		Ikke påvist	11990	Other	3	20	NOT_FOUND	NOT_FOUND	NOT_FOUND	NOT_FOUND	INTERMEDIATE	NOT_FOUND	NOT_FOUND	NOT_FOUND	penA_ins346D	0	1
541996.fasta	541996	SAMEA5608324	PRJEB32435	18	M	Vestland	West	Sekret	Urethra	2016-08-09	0,064	0,016	0,008	0,004	0,125	16	0,125	NA		NA	1594	Other	3	20	NOT_FOUND	NOT_FOUND	NOT_FOUND	NOT_FOUND	INTERMEDIATE	NOT_FOUND	NOT_FOUND	NOT_FOUND	penA_ins346D	0	1
554646.fasta	554646	SAMEA5608464	PRJEB32435	27	M	Oslo	East	Sekret	Urethra	2016-08-25	0,064	0,016	0,008	0,004	0,125	8	0,125	NA		NA	1594	Other	3	1267	NOT_FOUND	NOT_FOUND	NOT_FOUND	NOT_FOUND	INTERMEDIATE	NOT_FOUND	NOT_FOUND	NOT_FOUND	penA_ins346D	0	1
577121.fasta	577121	SAMEA5608807	PRJEB32435	19	F	Viken	East	Sekret	Vagina	2017-05-11	0,125	0,016	0,002	0,004	0,064	8	0,125	NA		NA	1594	Other	3	20	NOT_FOUND	NOT_FOUND	NOT_FOUND	NOT_FOUND	INTERMEDIATE	NOT_FOUND	NOT_FOUND	NOT_FOUND	penA_ins346D	0	1
601197.fasta	601197	SAMEA5609085	PRJEB32435	16	F	Viken	East	Sekret	Cervix uter	2017-10-06	0,125	0,016	0,004	0,004	0,064	8	0,25	NA		NA	1594	Other	3	20	NOT_FOUND	NOT_FOUND	NOT_FOUND	NOT_FOUND	INTERMEDIATE	NOT_FOUND	NOT_FOUND	NOT_FOUND	penA_ins346D	0	1
615952.fasta	615952	SAMEA6963039	PRJEB32435	29	F	Oslo	East	Ikke angitt		2018-02-02	0,064	0,016	0,004	0,002	0,064	8	0,125	NA		NA	1594	Other	3	851	NOT_FOUND	NOT_FOUND	NOT_FOUND	NOT_FOUND	INTERMEDIATE	NOT_FOUND	NOT_FOUND	NOT_FOUND	penA_ins346D	0	1
616223.fasta	616223	SAMEA6533455	PRJEB32435	35	M	Oslo	East	Sekret	Urethra	2018-02-01	0,064	0,016	0,004	0,002	0,064	8	0,125	NA		NA	1594	Other	3	851	NOT_FOUND	NOT_FOUND	NOT_FOUND	NOT_FOUND	INTERMEDIATE	NOT_FOUND	NOT_FOUND	NOT_FOUND	penA_ins346D	0	1
621063.fasta	621063	SAMEA6533480	PRJEB32435	22	M	NA	NA	SEKRET	URETRA	2018-03-05	0,125	0,016	0,002	0,002	0,064	8	0,064	-		NA	1594	Other	3	20	NOT_FOUND	NOT_FOUND	NOT_FOUND	NOT_FOUND	INTERMEDIATE	NOT_FOUND	NOT_FOUND	NOT_FOUND	penA_ins346D	0	1
657791.fasta	657791	SAMEA6534127	PRJEB32435	22	M	Oslo	East	Sekret	Urethra	2019-03-18	0,125	0,016	0,002	0,002	0,125	4	0,125		2	Ikke påvist	1594	Other	3	20	NOT_FOUND	NOT_FOUND	NOT_FOUND	NOT_FOUND	INTERMEDIATE	NOT_FOUND	NOT_FOUND	NOT_FOUND	penA_ins346D	0	1
657851.fasta	657851	SAMEA6534129	PRJEB32435	21	F	Vestfold og Telemark	East	Sekret	Cervix uter	2019-03-18	0,064	0,016	0,008	0,004	0,125	8	0,125	NA		NA	1594	Other	3	20	NOT_FOUND	NOT_FOUND	NOT_FOUND	NOT_FOUND	INTERMEDIATE	NOT_FOUND	NOT_FOUND	NOT_FOUND	penA_ins346D	0	1
677170.fasta	677170	SAMEA6534693	PRJEB32435	22	F	Rogaland	West	Sekret	Cervix uter	2019-11-11	0,125	0,016	0,016	0,004	0,25	8	0,125		8	Ikke påvist	1594	Other	3	20	NOT_FOUND	NOT_FOUND	NOT_FOUND	NOT_FOUND	INTERMEDIATE	NOT_FOUND	NOT_FOUND	NOT_FOUND	penA_ins346D	0	1
678904.fasta	678904	SAMEA6534735	PRJEB32435	64	M	Viken	East	Sekret	Urethra	2019-11-29	0,125	0,016	0,016	0,004	0,125	16	0,25		8	Ikke påvist	1594	Other	3	1267	NOT_FOUND	NOT_FOUND	NOT_FOUND	NOT_FOUND	INTERMEDIATE	NOT_FOUND	NOT_FOUND	NOT_FOUND	penA_ins346D	0	1
