# Supplementary material for: Genome analysis of Neisseria gonorrhoeae in Norway, 2016–2023, reveals shifting epidemiology in the wake of the COVID-19 pandemic
Source: Microb Genom. 2025 Sep 11;11(9):001479. doi: 10.1099/mgen.0.001479 (PMC12426203; doi:10.1099/mgen.0.001479)
Supplement: Uncited Table S2. [file mgen-11-01479-s003.pdf]

displayname	date of isolation	Sample alias	Country
SAMEA2298506	1996	DO96_47-sc-1649491	Denmark
SAMEA2298507	1996	DO96_92-sc-1649492	Denmark
SAMEA2074560	1998	989000164-sc-2013-05-22T10:46:54Z-1609326	Brasil
SAMN04191718	1998	20869	Canada
SAMN04191718	1998	20869	Canada
SAMN04191719	1998	20870	Canada
SAMN04191719	1998	20870	Canada
SAMEA2076959	1998	989000018-sc-2013-06-07T10:05:08Z-1642523	China
SAMEA2080468	1998	989000022-sc-2013-06-07T10:05:19Z-1642534	Philippines
SAMEA2074564	1998	989000236-sc-2013-05-22T10:47:13Z-1609341	USA
SAMEA2074759	1999	999000071-sc-2013-05-22T10:48:16Z-1609399	Spain
SAMEA3462888	2000	00AZI_C_001-sc-2320746	USA
SAMEA3463081	2000	00AZI_T_008-sc-2320878	USA
SAMEA3463083	2000	00AZI_T_009-sc-2320880	USA
SAMEA3463085	2000	00AZI_T_010-sc-2320882	USA
SAMEA3463086	2000	00AZI_T_011-sc-2320883	USA
SAMEA3463088	2000	00AZI_T_013-sc-2320886	USA
SAMEA3463087	2000	00AZI_T_012-sc-2320885	USA
SAMEA3463090	2000	00AZI_T_015-sc-2320888	USA
SAMEA3463094	2000	00AZI_T_019-sc-2320892	USA
SAMEA7495895	2001	CCITS-99	Argentina
SAMEA3463102	2001	01AZI_T_008-sc-2320900	USA
SAMEA3463104	2001	01AZI_T_010-sc-2320902	USA
SAMEA3463108	2001	01AZI_T_014-sc-2320907	USA
SAMEA3463109	2001	01AZI_T_015-sc-2320908	USA
SAMEA3463111	2002	02AZI_T_002-sc-2320910	USA
SAMEA3463123	2002	02AZI_T_009-sc-2320925	USA
SAMEA3463124	2002	02AZI_T_010-sc-2320927	USA
SAMEA3463127	2002	02AZI_T_013-sc-2320933	USA
SAMEA3463130	2002	02AZI_T_016-sc-2320940	USA
SAMEA3463132	2002	02AZI_T_018-sc-2320944	USA
SAMN05901200	2003	44162	Netherlands
SAMN05901200	2003	44162	Netherlands
SAMEA3463142	2003	03AZI_T_001-sc-2320964	USA
SAMEA3463143	2003	03AZI_T_002-sc-2320966	USA
SAMEA3463146	2003	03AZI_T_005-sc-2320972	USA
SAMEA3463154	2003	03AZI_T_013-sc-2320988	USA
SAMEA3463155	2003	03AZI_T_014-sc-2320990	USA
SAMEA3463156	2003	03AZI_T_015-sc-2320992	USA
SAMEA3463157	2003	03AZI_T_016-sc-2320994	USA
SAMEA2157700	2004	05_590_29-sc-2013-06-19T11:52:25Z-1649072	Australia
SAMEA104347864	2004	2004-04_2	Scotland
SAMEA104347865	2004	2004-06_3	Scotland
SAMEA104347866	2004	2004-07_4	Scotland
SAMEA3463016	2004	04AZI_C_021-sc-2320812	USA
SAMEA3463162	2004	04AZI_T_002-sc-2321006	USA
SAMEA3463163	2004	04AZI_T_003-sc-2321008	USA
SAMEA3463164	2004	04AZI_T_004-sc-2321010	USA
SAMEA3463166	2004	04AZI_T_006-sc-2321014	USA
SAMEA3463167	2004	04AZI_T_007-sc-2321016	USA
SAMEA3463171	2004	04AZI_T_011-sc-2321025	USA

SAMEA3463176	2004	04AZI_T_016-sc-2321035	USA
SAMEA3463178	2004	04AZI_T_018-sc-2321040	USA
SAMEA3463179	2004	04AZI_T_019-sc-2321042	USA
SAMEA3463181	2004	04AZI_T_021-sc-2321046	USA
SAMEA3463182	2004	04AZI_T_022-sc-2321048	USA
SAMEA3463203	2004	04AZI_T_043-sc-2321091	USA
SAMEA3463205	2004	04AZI_T_045-sc-2321095	USA
SAMEA7495875	2005	CCITS-79	Argentina
SAMN04191741	2005	26629	Canada
SAMN04191741	2005	26629	Canada
SAMN04191743	2005	26891	Canada
SAMN04191743	2005	26891	Canada
SAMN04191746	2005	27046	Canada
SAMN04191746	2005	27046	Canada
SAMN04191747	2005	27455	Canada
SAMN04191747	2005	27455	Canada
SAMEA104347867	2005	2005-02_5	Scotland
SAMEA3165407	2005	05AZI_T_005-sc-2186570	USA
SAMEA3165411	2005	05AZI_T_008-sc-2186574	USA
SAMEA3165414	2005	05AZI_T_010-sc-2186577	USA
SAMEA3165417	2005	05AZI_T_012-sc-2186580	USA
SAMEA3165421	2005	05AZI_T_015-sc-2186584	USA
SAMEA3165418	2005	05AZI_T_013-sc-2186581	USA
SAMEA3165422	2005	05AZI_T_016-sc-2186585	USA
SAMEA3165423	2005	05AZI_T_017-sc-2186586	USA
SAMEA3165425	2005	05AZI_T_018-sc-2186588	USA
SAMEA3165428	2005	05AZI_T_021-sc-2186592	USA
SAMEA3165429	2005	05AZI_T_022-sc-2186593	USA
SAMEA3165434	2005	05AZI_T_027-sc-2186598	USA
SAMEA3165435	2005	05AZI_T_028-sc-2186599	USA
SAMEA3165436	2005	05AZI_T_029-sc-2186600	USA
SAMEA3165503	2005	05AZI_C_001-sc-2186668	USA
SAMEA3165513	2005	05AZI_C_011-sc-2186678	USA
SAMEA7495876	2006	CCITS-80	Argentina
SAMN04191751	2006	27924	Canada
SAMN04191751	2006	27924	Canada
SAMN04191764	2006	30616	Canada
SAMN04191764	2006	30616	Canada
SAMN04191765	2006	30617	Canada
SAMN04191765	2006	30617	Canada
SAMEA104347868	2006	2006-06_6	Scotland
SAMEA104347869	2006	2006-07_7	Scotland
SAMEA104347870	2006	2006-08_8	Scotland
SAMEA104347871	2006	2006-11_9	Scotland
SAMEA3165441	2006	06AZI_T_004-sc-2186605	USA
SAMEA3165445	2006	06AZI_T_008-sc-2186609	USA
SAMEA3165447	2006	06AZI_T_010-sc-2186611	USA
SAMN04191756	2007	29360	Canada
SAMN04191756	2007	29360	Canada
SAMN04191757	2007	29362	Canada
SAMN04191757	2007	29362	Canada
SAMEA104347863	2007	2007-12_1	Scotland

SAMEA104347872	2007	2007-02_10	Scotland
SAMEA104347873	2007	2007-03_11	Scotland
SAMEA104347874	2007	2007-03_12	Scotland
SAMEA104347875	2007	2007-08_13	Scotland
SAMEA104347876	2007	2007-08_14	Scotland
SAMEA104347955	2007	2007-07_93	United Kingdom
SAMEA104347956	2007	2007-07_94	United Kingdom
SAMEA104347957	2007	2007-08_95	United Kingdom
SAMEA104347958	2007	2007-08_96	United Kingdom
SAMEA104347959	2007	2007-06_97	United Kingdom
SAMN04624841	2007	MMMOSAM:2d7206f5-2a5b-4f28-a8f0-f3ce01aeae37	United Kingdom
SAMN04624841	2007	MMMOSAM:2d7206f5-2a5b-4f28-a8f0-f3ce01aeae37	United Kingdom
SAMN04624930	2007	MMMOSAM:a20ce48f-6b1c-4a41-a351-5eb669a2d0b3	United Kingdom
SAMN04624930	2007	MMMOSAM:a20ce48f-6b1c-4a41-a351-5eb669a2d0b3	United Kingdom
SAMN04624962	2007	MMMOSAM:cefef4c9-5bcd-42b1-8ec9-9d126305dcd8	United Kingdom
SAMN04624962	2007	MMMOSAM:cefef4c9-5bcd-42b1-8ec9-9d126305dcd8	United Kingdom
SAMN04624975	2007	MMMOSAM:e337e896-21b3-4d70-a419-78d1f13b122c	United Kingdom
SAMN04624975	2007	MMMOSAM:e337e896-21b3-4d70-a419-78d1f13b122c	United Kingdom
SAMN04624998	2007	MMMOSAM:fd29694e-4e38-459f-a234-258f34febefc	United Kingdom
SAMN04624998	2007	MMMOSAM:fd29694e-4e38-459f-a234-258f34febefc	United Kingdom
SAMEA3165451	2007	07AZI_T_002-sc-2186615	USA
SAMEA3165453	2007	07AZI_T_004-sc-2186617	USA
SAMEA3165454	2007	07AZI_T_005-sc-2186618	USA
SAMEA3165455	2007	07AZI_T_006-sc-2186619	USA
SAMEA3165456	2007	07AZI_T_007-sc-2186620	USA
SAMEA3165472	2007	07AZI_T_023-sc-2186636	USA
SAMEA7495877	2008	CCITS-81	Argentina
SAMEA7495878	2008	CCITS-82	Argentina
SAMN03339567	2008	NGSJH7	Ireland
SAMN04624847	2008	MMMOSAM:33e91e5f-acb4-4116-8d12-13489e28ab65	United Kingdom
SAMN04624847	2008	MMMOSAM:33e91e5f-acb4-4116-8d12-13489e28ab65	United Kingdom
SAMN04624933	2008	MMMOSAM:a4a54667-3cb0-4bb9-b064-166ece1ba2d1	United Kingdom
SAMN04624933	2008	MMMOSAM:a4a54667-3cb0-4bb9-b064-166ece1ba2d1	United Kingdom
SAMN04624991	2008	MMMOSAM:f893ffe6-459c-421e-b1de-4f50b62a7f78	United Kingdom
SAMN04624991	2008	MMMOSAM:f893ffe6-459c-421e-b1de-4f50b62a7f78	United Kingdom
SAMEA3165478	2008	08AZI_T_005-sc-2186642	USA
SAMEA104313223	2009	AUSMDU00005546	Australia
SAMEA104313223	2009	AUSMDU00005546	Australia
SAMEA104313224	2009	AUSMDU00005547	Australia
SAMEA104313224	2009	AUSMDU00005547	Australia
SAMEA104313225	2009	AUSMDU00005548	Australia
SAMEA104313225	2009	AUSMDU00005548	Australia
SAMEA104313226	2009	AUSMDU00005549	Australia
SAMEA104313226	2009	AUSMDU00005549	Australia
SAMEA104347877	2009	2009-06_15	United Kingdom
ERS135265	2009		USA
ERS135274	2009		USA
ERS135347	2009		USA
ERS135538	2009		USA
ERS135559	2009		USA
SAMEA1690341	2009	GCGS144-sc-2012-05-03T12:02:57Z-1410646	USA
SAMEA1690395	2009	GCGS062-sc-2012-05-03T12:01:51Z-1410564	USA

SAMEA1690395	2009	GCGS062-sc-2012-05-03T12:01:51Z-1410564	USA
SAMEA1690440	2009	GCGS073-sc-2012-05-03T12:11:32Z-1410575	USA
SAMEA1690440	2009	GCGS073-sc-2012-05-03T12:11:32Z-1410575	USA
SAMEA1690450	2009	GCGS071-sc-2012-05-03T12:01:58Z-1410573	USA
SAMEA1690450	2009	GCGS071-sc-2012-05-03T12:01:58Z-1410573	USA
SAMEA1690468	2009	GCGS010-sc-2012-05-03T12:11:11Z-1410512	USA
SAMEA1690468	2009	GCGS010-sc-2012-05-03T12:11:11Z-1410512	USA
SAMEA7495880	2010	CCITS-84	Argentina
SAMEA2076671	2010	EST_10_16-sc-2013-06-07T10:03:13Z-1642432	Estonia
SAMN04624953	2010	MMMOSAM:c16a825f-5eb8-41b3-b6c7-0d0a09057e17	United Kingdom
SAMN04624953	2010	MMMOSAM:c16a825f-5eb8-41b3-b6c7-0d0a09057e17	United Kingdom
ERS135295	2010		USA
ERS135352	2010		USA
ERS135356	2010		USA
ERS135364	2010		USA
ERS135370	2010		USA
ERS135374	2010		USA
ERS135376	2010		USA
ERS135394	2010		USA
ERS135400	2010		USA
ERS135404	2010		USA
ERS135406	2010		USA
ERS135410	2010		USA
ERS135412	2010		USA
ERS135416	2010		USA
ERS135418	2010		USA
ERS135422	2010		USA
ERS135424	2010		USA
ERS135428	2010		USA
ERS135593	2010		USA
ERS135599	2010		USA
ERS135601	2010		USA
ERS135605	2010		USA
ERS135607	2010		USA
ERS135664	2010		USA
ERS135670	2010		USA
SAMEA1677292	2010	GCGS215-sc-2012-05-03T12:03:55Z-1410717	USA
SAMEA1677292	2010	GCGS215-sc-2012-05-03T12:03:55Z-1410717	USA
SAMEA1677308	2010	GCGS209-sc-2012-05-03T12:03:50Z-1410711	USA
SAMEA1677308	2010	GCGS209-sc-2012-05-03T12:03:50Z-1410711	USA
SAMEA1677316	2010	GCGS213-sc-2012-05-03T12:03:54Z-1410715	USA
SAMEA1677316	2010	GCGS213-sc-2012-05-03T12:03:54Z-1410715	USA
SAMEA1677323	2010	GCGS207-sc-2012-05-03T12:03:49Z-1410709	USA
SAMEA1677323	2010	GCGS207-sc-2012-05-03T12:03:49Z-1410709	USA
SAMEA1677331	2010	GCGS193-sc-2012-05-03T12:12:04Z-1410695	USA
SAMEA1677331	2010	GCGS193-sc-2012-05-03T12:12:04Z-1410695	USA
SAMEA1677398	2010	GCGS217-sc-2012-05-03T12:12:11Z-1410719	USA
SAMEA1677398	2010	GCGS217-sc-2012-05-03T12:12:11Z-1410719	USA
SAMEA1677399	2010	GCGS197-sc-2012-05-03T12:03:41Z-1410699	USA
SAMEA1677399	2010	GCGS197-sc-2012-05-03T12:03:41Z-1410699	USA
SAMEA1677410	2010	GCGS223-sc-2012-05-03T12:16:37Z-1410725	USA
SAMEA1677410	2010	GCGS223-sc-2012-05-03T12:16:37Z-1410725	USA

SAMEA1677414	2010	GCGS225-sc-2012-05-03T12:04:04Z-1410727	USA
SAMEA1677414	2010	GCGS225-sc-2012-05-03T12:04:04Z-1410727	USA
SAMEA1677418	2010	GCGS221-sc-2012-05-03T12:04:00Z-1410723	USA
SAMEA1677418	2010	GCGS221-sc-2012-05-03T12:04:00Z-1410723	USA
SAMEA1677423	2010	GCGS211-sc-2012-05-03T12:12:09Z-1410713	USA
SAMEA1677423	2010	GCGS211-sc-2012-05-03T12:12:09Z-1410713	USA
SAMEA1677425	2010	GCGS219-sc-2012-05-03T12:03:59Z-1410721	USA
SAMEA1677425	2010	GCGS219-sc-2012-05-03T12:03:59Z-1410721	USA
SAMEA1677426	2010	GCGS199-sc-2012-05-03T12:12:06Z-1410701	USA
SAMEA1677426	2010	GCGS199-sc-2012-05-03T12:12:06Z-1410701	USA
SAMEA1677481	2010	GCGS203-sc-2012-05-03T12:03:45Z-1410705	USA
SAMEA1677481	2010	GCGS203-sc-2012-05-03T12:03:45Z-1410705	USA
SAMEA1677487	2010	GCGS201-sc-2012-05-03T12:03:44Z-1410703	USA
SAMEA1690321	2010	GCGS171-sc-2012-05-03T12:03:19Z-1410673	USA
SAMEA1690321	2010	GCGS171-sc-2012-05-03T12:03:19Z-1410673	USA
SAMEA1690334	2010	GCGS191-sc-2012-05-03T12:03:36Z-1410693	USA
SAMEA1690334	2010	GCGS191-sc-2012-05-03T12:03:36Z-1410693	USA
SAMEA1690355	2010	GCGS175-sc-2012-05-03T12:12:00Z-1410677	USA
SAMEA1690358	2010	GCGS149-sc-2012-05-03T12:03:02Z-1410651	USA
SAMEA1690358	2010	GCGS149-sc-2012-05-03T12:03:02Z-1410651	USA
SAMEA1690367	2010	GCGS173-sc-2012-05-03T12:03:21Z-1410675	USA
SAMEA1690367	2010	GCGS173-sc-2012-05-03T12:03:21Z-1410675	USA
SAMEA1690382	2010	GCGS169-sc-2012-05-03T12:16:32Z-1410671	USA
SAMEA1690382	2010	GCGS169-sc-2012-05-03T12:16:32Z-1410671	USA
SAMEA1690434	2010	GCGS167-sc-2012-05-03T12:03:16Z-1410669	USA
SAMEA1690434	2010	GCGS167-sc-2012-05-03T12:03:16Z-1410669	USA
SAMEA1690479	2010	GCGS153-sc-2012-05-03T12:03:05Z-1410655	USA
SAMEA1690486	2010	GCGS092-sc-2012-05-03T12:02:15Z-1410594	USA
SAMEA1690486	2010	GCGS092-sc-2012-05-03T12:02:15Z-1410594	USA
SAMEA1690505	2010	GCGS161-sc-2012-05-03T12:03:11Z-1410663	USA
SAMEA1690505	2010	GCGS161-sc-2012-05-03T12:03:11Z-1410663	USA
SAMEA3165589	2010	10AZI_C_001-sc-2186754	USA
SAMN04624435	2011	MMMOSAM:2fe87d83-6cb9-4b3c-b34a-54394787c596	United Kingdom
SAMN04624435	2011	MMMOSAM:2fe87d83-6cb9-4b3c-b34a-54394787c596	United Kingdom
SAMN04624436	2011	MMMOSAM:311d43b2-469b-4d55-9e91-e30853217b19	United Kingdom
SAMN04624436	2011	MMMOSAM:311d43b2-469b-4d55-9e91-e30853217b19	United Kingdom
SAMN04624439	2011	MMMOSAM:32bbe997-d8b2-4a15-8742-556a955c8fb2	United Kingdom
SAMN04624439	2011	MMMOSAM:32bbe997-d8b2-4a15-8742-556a955c8fb2	United Kingdom
SAMN04625158	2011	MMMOSAM:6e309435-0a5f-4ab1-9dc5-9e3d7078cd21	United Kingdom
SAMN04625158	2011	MMMOSAM:6e309435-0a5f-4ab1-9dc5-9e3d7078cd21	United Kingdom
SAMEA3165495	2011	11AZI_T_008-sc-2186659	USA
SAMEA3165619	2011	11CFX_T_002-sc-2186785	USA
SAMEA3165729	2011	11AZI_C_010-sc-2186895	USA
SAMEA3165732	2011	11AZI_C_011-sc-2186898	USA
SAMEA3165733	2011	11CFX_T_029-sc-2186899	USA
SAMEA3165734	2011	11CFX_T_031-sc-2186900	USA
SAMEA3233686	2011	11CFX_T_056-sc-2204593	USA
SAMN09273317	2012	NGRL_180505	Ireland
SAMN09273317	2012	NGRL_180505	Ireland
SAMEA2080403	2012	SI12_18-sc-2013-06-07T10:04:48Z-1642510	Slovenia
SAMEA104347960	2012	2012-07_98	United Kingdom
SAMEA104347961	2012	2012-09_99	United Kingdom

SAMN04621993	2012	MMMOSAM:740ba171-bb5b-40f2-a1a2-866a1ac974e3	United Kingdom
SAMN04621993	2012	MMMOSAM:740ba171-bb5b-40f2-a1a2-866a1ac974e3	United Kingdom
SAMN04624188	2012	MMMOSAM:01e05f84-f4bf-46c9-afe0-745e33b4edd7	United Kingdom
SAMN04624188	2012	MMMOSAM:01e05f84-f4bf-46c9-afe0-745e33b4edd7	United Kingdom
SAMN04624222	2012	MMMOSAM:2d01b767-457f-46c5-9fe4-1ffc3d041d40	United Kingdom
SAMN04624222	2012	MMMOSAM:2d01b767-457f-46c5-9fe4-1ffc3d041d40	United Kingdom
SAMN04624224	2012	MMMOSAM:300cf1b2-03de-4c86-8d58-ca8d8eb71b19	United Kingdom
SAMN04624224	2012	MMMOSAM:300cf1b2-03de-4c86-8d58-ca8d8eb71b19	United Kingdom
SAMN04624316	2012	MMMOSAM:a69b8e04-1ddb-47ca-85a5-4e19190dce68	United Kingdom
SAMN04624316	2012	MMMOSAM:a69b8e04-1ddb-47ca-85a5-4e19190dce68	United Kingdom
SAMN04624356	2012	MMMOSAM:ce242d3f-6270-4228-8e17-40549bd4b3ca	United Kingdom
SAMN04624356	2012	MMMOSAM:ce242d3f-6270-4228-8e17-40549bd4b3ca	United Kingdom
SAMN04624360	2012	MMMOSAM:d4d7e602-430d-44a4-9600-d79c673b6fc1	United Kingdom
SAMN04624360	2012	MMMOSAM:d4d7e602-430d-44a4-9600-d79c673b6fc1	United Kingdom
SAMN04624547	2012	MMMOSAM:be28fc0e-e751-4488-9464-4c193bb47819	United Kingdom
SAMN04624574	2012	MMMOSAM:df2d3bad-fb5e-44bf-a9b8-73474af35439	United Kingdom
SAMN04624574	2012	MMMOSAM:df2d3bad-fb5e-44bf-a9b8-73474af35439	United Kingdom
SAMEA4709542	2012	839b48c0-2bf6-11e6-a63e-3c4a9275d6c8	United States
SAMEA4709916	2012	b20a5d18-31b7-11e8-88eb-3c4a9275d6c8	United States
SAMEA4710136	2012	33a117f4-21eb-11e8-9200-3c4a9275d6c8	United States
SAMEA3165412	2012	12CFX_C_048-sc-2186575	USA
SAMEA3165656	2012	12AZI_T_007-sc-2186822	USA
SAMEA3165663	2012	12AZI_T_012-sc-2186829	USA
SAMEA3165665	2012	12AZI_T_013-sc-2186831	USA
SAMEA3165759	2012	12AZI_C_003-sc-2186925	USA
SAMEA7495887	2013	CCITS-91	Argentina
SAMEA104147536	2013	93958720-513b-11e7-ac63-3c4a9275d6c8	United Kingdom
SAMEA104347962	2013	2013-08_100	United Kingdom
SAMEA13008691	2013	NG010	United Kingdom
SAMEA5994590	2013	d17f4780-b62a-11e6-8d8d-3c4a9275d6c8	United Kingdom
SAMEA5994683	2013	d996a8f0-b62a-11e6-8d8d-3c4a9275d6c8	United Kingdom
SAMEA5994692	2013	d9fe4460-b62a-11e6-8d8d-3c4a9275d6c8	United Kingdom
SAMEA5994713	2013	dabbc800-b62a-11e6-8d8d-3c4a9275d6c8	United Kingdom
SAMEA5994840	2013	e99de6a0-b62a-11e6-8d8d-3c4a9275d6c8	United Kingdom
SAMEA5994843	2013	e9b34360-b62a-11e6-8d8d-3c4a9275d6c8	United Kingdom
SAMEA5994846	2013	e9beb510-b62a-11e6-8d8d-3c4a9275d6c8	United Kingdom
SAMEA5994883	2013	eae02aa0-b62a-11e6-8d8d-3c4a9275d6c8	United Kingdom
SAMEA5994897	2013	eb4c32e0-b62a-11e6-8d8d-3c4a9275d6c8	United Kingdom
SAMN04621899	2013	MMMOSAM:01cc5c52-1043-4103-bc8b-1fbb537cad1d	United Kingdom
SAMN04621899	2013	MMMOSAM:01cc5c52-1043-4103-bc8b-1fbb537cad1d	United Kingdom
SAMN04621925	2013	MMMOSAM:254f375b-f502-4bac-9527-05a75537ca1c	United Kingdom
SAMN04621925	2013	MMMOSAM:254f375b-f502-4bac-9527-05a75537ca1c	United Kingdom
SAMN04622017	2013	MMMOSAM:882504cd-7674-4de2-a880-d4deada07faa	United Kingdom
SAMN04622017	2013	MMMOSAM:882504cd-7674-4de2-a880-d4deada07faa	United Kingdom
SAMN04622045	2013	MMMOSAM:be68a1ac-311e-4e01-9c5e-76e16431f00d	United Kingdom
SAMN04622045	2013	MMMOSAM:be68a1ac-311e-4e01-9c5e-76e16431f00d	United Kingdom
SAMN04622075	2013	MMMOSAM:e4962047-ad0b-4578-ba33-70ea81e0c698	United Kingdom
SAMN04622075	2013	MMMOSAM:e4962047-ad0b-4578-ba33-70ea81e0c698	United Kingdom
SAMN04623605	2013	MMMOSAM:0f426dc2-5851-4196-addd-f27549a60ce3	United Kingdom
SAMN04623605	2013	MMMOSAM:0f426dc2-5851-4196-addd-f27549a60ce3	United Kingdom
SAMN04623763	2013	MMMOSAM:dc4221bb-e601-4c5d-8333-70494c44b6e3	United Kingdom
SAMN04623763	2013	MMMOSAM:dc4221bb-e601-4c5d-8333-70494c44b6e3	United Kingdom

SAMN04624606	2013	MMMOSAM:060c6c45-c6b1-463f-bbb0-9bfef661a4c3	United Kingdom
SAMN04624606	2013	MMMOSAM:060c6c45-c6b1-463f-bbb0-9bfef661a4c3	United Kingdom
SAMEA4709696	2013	9967e190-2bf6-11e6-a63e-3c4a9275d6c8	United States
SAMEA4709604	2013	8de91230-2bf6-11e6-a63e-3c4a9275d6c8	United States
SAMEA4710059	2013	15505a62-21eb-11e8-9200-3c4a9275d6c8	United States
SAMN03339571	2014	NGSJH11	Ireland
SAMN09273310	2014	NGRL_176561	Ireland
SAMN09273310	2014	NGRL_176561	Ireland
SAMN09273313	2014	NGRL_178526	Ireland
SAMN09273313	2014	NGRL_178526	Ireland
SAMEA104347923	2014	2014-04_61	Scotland
SAMEA104347924	2014	2014-04_62	Scotland
SAMEA104347925	2014	2014-04_63	Scotland
SAMEA104347926	2014	2014-06_64	Scotland
SAMEA104347927	2014	2014-06_65	Scotland
SAMEA104347928	2014	2014-08_66	Scotland
SAMEA104347929	2014	2014-10_67	Scotland
SAMEA104347930	2014	2014-10_68	Scotland
SAMEA104347931	2014	2014-10_69	Scotland
SAMEA104347878	2014	2014-06_16	United Kingdom
SAMEA104347879	2014	2014-11_17	United Kingdom
SAMEA5994741	2014	e27c7df0-b62a-11e6-8d8d-3c4a9275d6c8	United Kingdom
SAMEA5994972	2014	f274a070-b62a-11e6-8d8d-3c4a9275d6c8	United Kingdom
SAMN09273315	2015	NGRL_179411	Ireland
SAMN09273315	2015	NGRL_179411	Ireland
SAMN09273340	2015	NGRL_UHW6	Ireland
SAMN09273340	2015	NGRL_UHW6	Ireland
SAMEA104347904	2015	2015-02_42	Scotland
SAMEA104347905	2015	2015-12_43	Scotland
SAMEA104347932	2015	2015-06_70	Scotland
SAMEA104347880	2015	2015-01_18	United Kingdom
SAMEA104347881	2015	2015-02_19	United Kingdom
SAMEA104347882	2015	2015-02_20	United Kingdom
SAMEA104347883	2015	2015-03_21	United Kingdom
SAMEA104347884	2015	2015-03_22	United Kingdom
SAMEA104347885	2015	2015-06_23	United Kingdom
SAMEA104347886	2015	2015-06_24	United Kingdom
SAMEA104347887	2015	2015-06_25	United Kingdom
SAMEA104347888	2015	2015-07_26	United Kingdom
SAMEA104347889	2015	2015-07_27	United Kingdom
SAMEA104347890	2015	2015-08_28	United Kingdom
SAMEA104347891	2015	2015-09_29	United Kingdom
SAMEA104347892	2015	2015-09_30	United Kingdom
SAMEA104347893	2015	2015-10_31	United Kingdom
SAMEA104347894	2015	2015-09_32	United Kingdom
SAMEA104347895	2015	2015-10_33	United Kingdom
SAMEA104347896	2015	2015-10_34	United Kingdom
SAMEA104347897	2015	2015-10_35	United Kingdom
SAMEA104347898	2015	2015-11_36	United Kingdom
SAMEA104347899	2015	2015-11_37	United Kingdom
SAMEA104347900	2015	2015-12_38	United Kingdom
SAMEA104347901	2015	2015-12_39	United Kingdom

SAMEA104347902	2015	2015-12_40	United Kingdom
SAMEA104347963	2015	2015-07_101	United Kingdom
SAMEA4357103	2015	101260	United Kingdom
SAMEA4357104	2015	101261	United Kingdom
SAMEA4357105	2015	101262	United Kingdom
SAMEA4357106	2015	101263	United Kingdom
SAMEA4357108	2015	110944	United Kingdom
SAMEA4357109	2015	110947	United Kingdom
SAMEA5995067	2015	fa647cb0-b62a-11e6-8d8d-3c4a9275d6c8	United Kingdom
SAMN08331923	2015	GCWGS_1084	United States
SAMEA7495891	2016	CCITS-95	Argentina
SAMEA7495894	2016	CCITS-98	Argentina
SAMEA6595528	2016	BH02	Brazil
SAMEA6595536	2016	BH10	Brazil
SAMEA6595713	2016	BH83	Brazil
SAMN09273304	2016	NGRL_76972	Ireland
SAMN09273304	2016	NGRL_76972	Ireland
SAMN09273319	2016	NGRL_190440	Ireland
SAMN09273319	2016	NGRL_190440	Ireland
SAMEA5608330	2016	542783	Norway
SAMEA5608330	2016	542783	Norway
SAMEA5608422	2016	553651	Norway
SAMEA5608422	2016	553651	Norway
SAMEA5608469	2016	554680	Norway
SAMEA5608469	2016	554680	Norway
SAMEA5608471	2016	554689	Norway
SAMEA5608471	2016	554689	Norway
SAMEA5608496	2016	555523	Norway
SAMEA104347906	2016	2016-01_44	Scotland
SAMEA104347935	2016	2016-02_73	Scotland
SAMEA104147345	2016	7aec3430-513b-11e7-ac63-3c4a9275d6c8	United Kingdom
SAMEA104147356	2016	7b884d20-513b-11e7-ac63-3c4a9275d6c8	United Kingdom
SAMEA104147438	2016	8733f8e0-513b-11e7-ac63-3c4a9275d6c8	United Kingdom
SAMEA104347903	2016	2016-02_41	United Kingdom
SAMEA104347907	2016	2016-02_45	United Kingdom
SAMEA104347908	2016	2016-02_46	United Kingdom
SAMEA104347909	2016	2016-02_47	United Kingdom
SAMEA104347910	2016	2016-02_48	United Kingdom
SAMEA104347911	2016	2016-03_49	United Kingdom
SAMEA104347912	2016	2016-03_50	United Kingdom
SAMEA104347913	2016	2016-03_51	United Kingdom
SAMEA104347914	2016	2016-03_52	United Kingdom
SAMEA104347915	2016	2016-03_53	United Kingdom
SAMEA104347916	2016	2016-03_54	United Kingdom
SAMEA104347917	2016	2016-03_55	United Kingdom
SAMEA104347918	2016	2016-04_56	United Kingdom
SAMEA104347919	2016	2016-04_57	United Kingdom
SAMEA104347920	2016	2016-04_58	United Kingdom
SAMEA104347921	2016	2016-06_59	United Kingdom
SAMEA104347922	2016	2016-06_60	United Kingdom
SAMEA104347933	2016	2016-07_71	United Kingdom
SAMEA104347934	2016	2016-07_72	United Kingdom

SAMEA104347936	2016	2016-07_74	United Kingdom
SAMEA104347937	2016	2016-07_75	United Kingdom
SAMEA104347938	2016	2016-07_76	United Kingdom
SAMEA104347939	2016	2016-08_77	United Kingdom
SAMEA104347941	2016	2016-08_79	United Kingdom
SAMEA104347942	2016	2016-10_80	United Kingdom
SAMEA104347943	2016	2016-10_81	United Kingdom
SAMEA104347944	2016	2016-10_82	United Kingdom
SAMEA104347945	2016	2016-10_83	United Kingdom
SAMEA104347946	2016	2016-11_84	United Kingdom
SAMN05859808	2016	GCWGS_0311	United States
SAMN10253499	2016	LRRBGS_0326	United States
SAMN10253512	2016	LRRBGS_0342	United States
SAMN10253514	2016	LRRBGS_0344	United States
SAMN10253616	2016	LRRBGS_0218	United States
SAMN05859808	2016	GCWGS_0311	USA
SAMEA6124501	2016	VNM16-97	Vietnam
SAMEA6124501	2016	VNM16-97	Vietnam
SAMEA7495801	2017	CCITS-02	Argentina
SAMEA7495802	2017	CCITS-03	Argentina
SAMEA7495804	2017	CCITS-05	Argentina
SAMEA7495808	2017	CCITS-09	Argentina
SAMEA7495815	2017	CCITS-17	Argentina
SAMEA7495823	2017	CCITS-25	Argentina
SAMEA7495824	2017	CCITS-26	Argentina
SAMN10912899	2017	AUSMDU00011107	Australia
SAMN10912899	2017	AUSMDU00011107	Australia
SAMN10913326	2017	AUSMDU00011945	Australia
SAMN10913326	2017	AUSMDU00011945	Australia
SAMN10913554	2017	AUSMDU00012447	Australia
SAMN10913554	2017	AUSMDU00012447	Australia
SAMN10914583	2017	AUSMDU00014451	Australia
SAMN10914583	2017	AUSMDU00014451	Australia
SAMN10914870	2017	AUSMDU00014952	Australia
SAMN10914870	2017	AUSMDU00014952	Australia
SAMN10914929	2017	AUSMDU00014987	Australia
SAMN10914929	2017	AUSMDU00014987	Australia
SAMN10920623	2017	AUSMDU00012728	Australia
SAMN10920623	2017	AUSMDU00012728	Australia
SAMN23565762	2017	49685	Canada
SAMEA5608821	2017	577666	Norway
SAMEA5608821	2017	577666	Norway
SAMEA5608827	2017	577916	Norway
SAMEA5608827	2017	577916	Norway
SAMEA5608834	2017	578295	Norway
SAMEA5608834	2017	578295	Norway
SAMEA5608874	2017	590801	Norway
SAMEA5608874	2017	590801	Norway
SAMEA5608952	2017	593771	Norway
SAMEA5608952	2017	593771	Norway
SAMEA5609079	2017	600792	Norway
SAMEA5609079	2017	600792	Norway

SAMEA104347947	2017	2017-01_85	United Kingdom
SAMEA104347949	2017	2017-02_87	United Kingdom
SAMEA104347950	2017	2017-02_88	United Kingdom
SAMEA104347951	2017	2017-02_89	United Kingdom
SAMEA104347952	2017	2017-02_90	United Kingdom
SAMEA104347953	2017	2017-02_91	United Kingdom
SAMEA104347954	2017	2017-02_92	United Kingdom
SAMN08357525	2017	GCWGS_1447	USA
SAMN08553214	2017	GCWGS_1533	USA
SAMEA7495814	2018	CCITS-16	Argentina
SAMEA7495819	2018	CCITS-21	Argentina
SAMEA7495822	2018	CCITS-24	Argentina
SAMEA7495825	2018	CCITS-27	Argentina
SAMEA7495826	2018	CCITS-28	Argentina
SAMEA7495832	2018	CCITS-34	Argentina
SAMEA7495836	2018	CCITS-38	Argentina
SAMEA7495842	2018	CCITS-45	Argentina
SAMEA7495838	2018	CCITS-40	Argentina
SAMEA7495843	2018	CCITS-46	Argentina
SAMEA7495844	2018	CCITS-47	Argentina
SAMEA7495845	2018	CCITS-48	Argentina
SAMEA7495846	2018	CCITS-49	Argentina
SAMN23566245	2018	54512	Canada
SAMN23566250	2018	54536	Canada
SAMN23566334	2018	56151	Canada
SAMN23566374	2018	56348	Canada
SAMEA11548686	2018	NG_AMS0439	Netherlands
SAMEA11548687	2018	NG_AMS0440	Netherlands
SAMEA6533623	2018	631506	Norway
SAMEA6533702	2018	634426	Norway
SAMEA6533721	2018	635424	Norway
SAMEA6533749	2018	636538	Norway
SAMEA6533763	2018	636875	Norway
SAMEA6533972	2018	646792	Norway
SAMN08865588	2018	GCWGS_2243	USA
SAMN09664977	2018	GCWGS_2846	USA
SAMN09666457	2018	GCWGS_2927	USA
SAMN09666467	2018	GCWGS_2937	USA
SAMN09666472	2018	GCWGS_2943	USA
SAMN10148359	2018	GCWGS_3857	USA
SAMN10793560	2018	GCWGS_4792	USA
SAMN10925280	2018	GCWGS_5136	USA
SAMN11514949	2018	GCWGS_6156	USA
SAMN11554222	2018	GCWGS_5961	USA
SAMN12883891	2018	GCWGS_7299	USA
SAMEA7495851	2019	CCITS-54	Argentina
SAMEA7495854	2019	CCITS-57	Argentina
SAMEA7495855	2019	CCITS-58	Argentina
SAMEA7495856	2019	CCITS-59	Argentina
SAMEA7495859	2019	CCITS-63	Argentina
SAMEA7495864	2019	CCITS-68	Argentina
SAMEA7495866	2019	CCITS-70	Argentina

SAMEA7495869	2019	CCITS-73	Argentina
SAMEA7495870	2019	CCITS-74	Argentina
SAMEA7495873	2019	CCITS-77	Argentina
SAMEA7495874	2019	CCITS-78	Argentina
SAMN11482586	2019	LRRBGS_0064	Brazil
SAMEA7492469	2019	NG_AMS0267	Netherlands
SAMN19718164	2019	NZ19AR0452	New Zealand
SAMEA6534096	2019	655342	Norway
SAMEA6534147	2019	658888	Norway
SAMEA6534444	2019	667990	Norway
SAMEA6534449	2019	668038	Norway
SAMEA6534453	2019	668042	Norway
SAMEA6534488	2019	669061	Norway
SAMEA6534562	2019	671389	Norway
SAMEA6534577	2019	671550	Norway
SAMEA6534579	2019	671555	Norway
SAMEA6534584	2019	671770	Norway
SAMEA6534591	2019	672415	Norway
SAMEA6534594	2019	672561	Norway
SAMEA6534679	2019	676685	Norway
SAMEA6534682	2019	676791	Norway
SAMEA6534683	2019	676800	Norway
SAMN17051030	2019	HUA1941	Spain
SAMN11463677	2019	GCWGS_5999	USA
SAMN11463682	2019	GCWGS_6004	USA
SAMN11938415	2019	GCWGS_6530	USA
SAMN12684809	2019	GCWGS_7412	USA
SAMN13047881	2019	GCWGS_8778	USA
SAMN13191780	2019	GCWGS_8962	USA
SAMN13191789	2019	GCWGS_8971	USA
SAMN13191791	2019	GCWGS_8973	USA
SAMN13495300	2019	GCWGS_9053	USA
SAMN13495525	2019	GCWGS_9462	USA
SAMN13513403	2019	GCWGS_9805	USA
SAMN13839847	2019	GCWGS_9484	USA
SAMN13918417	2019	GCWGS_10138	USA
SAMN14170428	2019	GCWGS-10686	USA
SAMN14170458	2019	GCWGS-10716	USA
SAMN14170501	2019	GCWGS-11110	USA
SAMN14588957	2019	GCWGS-11725	USA
SAMN14640508	2019	GCWGS-11768	USA
SAMN14835642	2019	GCWGS_9156	USA
SAMN15468324	2020	GCWGS-12770	USA
SAMN15468328	2020	GCWGS-12774	USA
SAMN15970657	2020	GCWGS-12972	USA
SAMN16293131	2020	GCWGS-13714	USA
SAMN16423174	2020	GCWGS-13200	USA
SAMN16877352	2020	GCWGS-14102	USA
SAMN16877357	2020	GCWGS-14107	USA
SAMN17315506	2020	GCWGS-14091	USA
SAMN17833994	2020	GCWGS-14703	USA
SAMN18524931	2020	GCWGS-15286	USA

SAMN21984647	2020	GCWGS-16592	USA
SAMN21984667	2020	GCWGS-16612	USA
SAMN20867011	2021	GCWGS-16309	USA
SAMN21984552	2021	GCWGS-16382	USA
SAMN21984558	2021	GCWGS-16388	USA
SAMN21984562	2021	GCWGS-16392	USA
SAMN21984609	2021	GCWGS-16518	USA
SAMN24041290	2021	GCWGS-16919	USA
SAMN24041308	2021	GCWGS-16937	USA
SAMN24041327	2021	GCWGS-16956	USA
SAMN24041483	2021	GCWGS-17339	USA
